# Moiré materials based on M-point twisting

**DOI:** 10.1038/s41586-025-09187-5

**Published:** 2025-07-09

**Authors:** Dumitru Călugăru, Yi Jiang, Haoyu Hu, Hanqi Pi, Jiabin Yu, Maia G. Vergniory, Jie Shan, Claudia Felser, Leslie M. Schoop, Dmitri K. Efetov, Kin Fai Mak, B. Andrei Bernevig

**Affiliations:** 1 Department of Physics, Princeton University, Princeton, NJ USA; 2 Rudolf Peierls Centre for Theoretical Physics, University of Oxford, Oxford, UK; 3 Donostia International Physics Center, Donostia-San Sebastián, Spain; 4 Beijing National Laboratory for Condensed Matter Physics, Institute of Physics, Chinese Academy of Sciences, Beijing, China; 5 University of Chinese Academy of Sciences, Beijing, China; 6 Department of Physics, University of Florida, Gainesville, FL USA; 7 Département de physique et Institut quantique, Université de Sherbrooke, Sherbrooke, Quebec Canada; 8 Regroupement Québécois sur les Matériaux de Pointe (RQMP), Montréal, Quebec Canada; 9 Max Planck Institute for the Structure and Dynamics of Matter, Hamburg, Germany; 10 School of Applied and Engineering Physics, Department of Physics, Cornell University, Ithaca, NY USA; 11 Kavli Institute at Cornell for Nanoscale Science, Ithaca, NY USA; 12 Max Planck Institute for Chemical Physics of Solids, Dresden, Germany; 13 Department of Chemistry, Princeton University, Princeton, NJ USA; 14 Faculty of Physics, Ludwig Maximilian University of Munich, Munich, Germany; 15 Munich Center for Quantum Science and Technology (MCQST), Ludwig Maximilian University of Munich, Munich, Germany; 16 Ikerbasque, the Basque Foundation for Science, Bilbao, Spain

**Keywords:** Two-dimensional materials, Ferromagnetism

## Abstract

When two monolayer materials are stacked with a relative twist, an effective moiré translation symmetry emerges, leading to fundamentally different properties in the resulting heterostructure. As such, moiré materials have recently provided highly tunable platforms for exploring strongly correlated systems^1,2^. However, previous studies have focused almost exclusively on monolayers with triangular lattices and low-energy states near the Γ (refs. ^3,4^) or K (refs. ^5–9^) points of the Brillouin zone (BZ). Here we introduce a new class of moiré systems based on monolayers with triangular lattices but low-energy states at the M points of the BZ. These M-point moiré materials feature three time-reversal-preserving valleys related by threefold rotational symmetry. We propose twisted bilayers of exfoliable 1T-SnSe_2_ and 1T-ZrS_2_ as realizations of this new class. Using extensive ab initio simulations, we identify twist angles that yield flat conduction bands, provide accurate continuum models, analyse their topology and charge density and explore the platform’s rich physics. Notably, the M-point moiré Hamiltonians exhibit emergent momentum-space non-symmorphic symmetries and a kagome plane-wave lattice structure. This represents, to our knowledge, the first experimentally viable realization of projective representations of crystalline space groups in a non-magnetic system. With interactions, these systems act as six-flavour Hubbard simulators with Mott physics. Moreover, the presence of a momentum-space non-symmorphic in-plane mirror symmetry renders some of the M-point moiré Hamiltonians quasi-one-dimensional in each valley, suggesting the possibility of realizing Luttinger-liquid physics.

## Abstract (web summary)

A new class of moiré materials based on monolayers with triangular lattices and low-energy states at the M points of the Brillouin zone is introduced, demonstrating emergent momentum-space non-symmorphic symmetries, a kagome plane-wave lattice structure, and potential quasi-one-dimensionality.

## Main

Moiré heterostructures have recently emerged as versatile quantum simulators of archetypal condensed matter models^1,2^. When two identical or nearly identical monolayers are twisted, the resulting moiré modulation of the interlayer potential gives rise to an effective moiré discrete translation symmetry. In the moiré BZ, the moiré-modulated interlayer hybridization opens gaps in the folded band structure, quenching the kinetic energy of the monolayer electrons^10^. The moiré system thus enters an interaction-dominated regime, providing a tunable platform for simulating various prototypical condensed matter systems. A notable example is twisted bilayer graphene^5^, which hosts unconventional superconductors^11^ and correlated insulators^12^ near the magic angle and has recently been shown to simulate topological heavy-fermions^13,14^. Transition-metal dichalcogenide (TMD) heterobilayers can emulate the Hubbard model on a triangular lattice^7,15^, whereas twisted WTe_2_ exhibits signatures of a one-dimensional Luttinger liquid, although its theoretical description remains challenging owing to the complex monolayer band structure^16^. Beyond these examples, a growing body of theoretical and experimental work has explored other exotic phases in TMDs^17–22^. Furthermore, both integer and fractional Chern insulator states have been reported in moiré TMD^23–30^, graphene^31–33^ and graphene–boron nitride heterostructures^34–37^.

Until now, nearly all moiré heterostructures have been based on twisting monolayers with triangular lattices and low-energy states near the Γ (refs. ^3,4^) or K (refs. ^5–9^) points, leading to systems with one or two valleys (in the two-valley case, time-reversal exchanges the valley). This work introduces a new family of moiré materials by twisting monolayers with triangular lattices and low-energy states around the M point of the BZ. These M-point moiré systems feature three time-reversal-preserving valleys related by *C*_3*z*_ rotation symmetry. Building on extensive ab initio calculations, we propose (among others^38^) experimentally exfoliable twisted SnSe_2_ and ZrS_2_ as promising platforms for realizing M-point moiré heterostructures. We develop quantitative simplified models for these systems and perform a detailed analysis of the band structure, topology and charge density of the flat bands at the predicted small twist angles. We show analytically that M-point moiré Hamiltonians exhibit a new type of symmetry, termed momentum-space non-symmorphic^39–43^. In crystallography, space groups are symmorphic or non-symmorphic, depending on whether they include symmetry operations that translate the origin by a fraction of the lattice vectors. Although in real space conventional crystalline groups can feature both symmorphic and non-symmorphic operations, in momentum space, all conventional crystalline groups exhibit only symmorphic operations. M-point moiré systems are the first experimentally realizable non-magnetic systems to exhibit momentum-space non-symmorphic symmetries, all without requiring an applied magnetic field in the range of thousands of Tesla^39–41^. In a single valley, these non-symmorphic symmetries can render the system effectively one-dimensional at the single-particle level, making M-point moiré systems prime candidates for Luttinger-liquid simulators^16,44^. With all three valleys considered, they can realize a multi-orbital triangular lattice Hubbard model (H. Hu et al., to be published), in which valley-spin local moments couple differently along the three *C*_3*z*_-related directions, in a manner reminiscent of Kitaev’s honeycomb model^45^.

## M-point moiré models

**Fig. 1:**
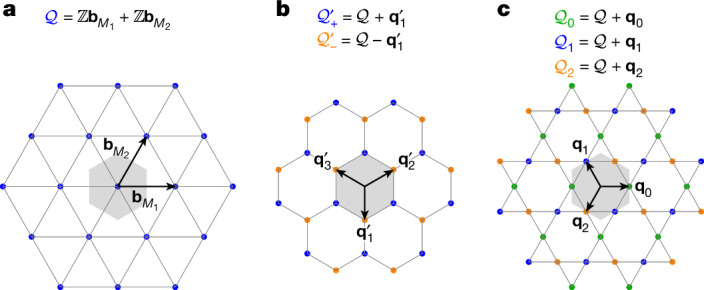
Momentum-space Q-lattices for twisted triangular lattice monolayers. **a**–**c**, The three panels correspond to the cases in which the low-energy degrees of freedom are located at the Γ (**a**), K (**b**) and M (**c**) points. In each panel, the sublattices are coloured according to the legend above each plot. The moiré BZ is shown by the grey hexagon, whereas the reciprocal moiré vectors ${{\bf{b}}}_{{M}_{1,2}}$ as well as the auxiliary vectors ${{\bf{q}}}_{1,2,3}^{{\prime} }$ and ${{\bf{q}}}_{0,1,2}$ are shown by the black arrows.

For triangular monolayer lattices, the moiré lattice is also triangular, generated by the reciprocal lattice vectors ${{\bf{b}}}_{{M}_{1}}$ and ${{\bf{b}}}_{{M}_{2}}$ (see Supplementary Information Section IV). These vectors span the moiré reciprocal lattice ${\mathcal{Q}}={\mathbb{Z}}{{\bf{b}}}_{{M}_{1}}+{\mathbb{Z}}{{\bf{b}}}_{{M}_{2}}$, as depicted in Fig. 1a. In general, the single-particle Hamiltonians of moiré systems take the form of a hopping model in momentum space. This arises because the moiré potential breaks the monolayer translation symmetry and couples momentum states that are connected by reciprocal moiré vectors. The single-particle moiré Hamiltonian can be written ${\mathcal{H}}={\sum }_{{\bf{k}},{\bf{Q}},{{\bf{Q}}}^{{\prime} },i,j}{[{h}_{{\bf{Q}},{{\bf{Q}}}^{{\prime} }}({\bf{k}})]}_{ij}{\widehat{c}}_{{\bf{k}},{\bf{Q}},i}^{\dagger }{\widehat{c}}_{{\bf{k}},{{\bf{Q}}}^{{\prime} },j}$, in which ${\widehat{c}}_{{\bf{k}},{\bf{Q}},i}^{\dagger }$ denotes the moiré plane-wave operators at moiré momentum **k**, and *i* denotes a combined index comprising orbital, spin, valley, layer or other further degrees of freedom.

When the low-energy fermions of the monolayer are located at the Γ point^3,4^, the operators ${\widehat{c}}_{{\bf{k}},{\bf{Q}},i}^{\dagger }$ carry total momentum **k** − **Q** and the **Q**-vectors lie on the triangular lattice shown in Fig. 1a. In the case of a monolayer with low-energy states located at the K point^5–7^, the moiré fermions carry an extra valley index *η* = ±, in which the moiré Hamiltonian is diagonal. The **Q**-vectors form a honeycomb lattice, as illustrated in Fig. 1b. The moiré and monolayer operators are related by ${\widehat{c}}_{{\bf{k}},{\bf{Q}},\eta ,i}^{\dagger }={\widehat{a}}_{\eta {{\bf{K}}}_{{\rm{K}}}^{l}+{\bf{k}}-{\bf{Q}},l,i}^{\dagger }$ for ${\bf{Q}}\in {{\mathcal{Q}}}_{\eta l}^{{\prime} }$, in which ${\widehat{a}}_{{\bf{p}},l,i}^{\dagger }$ represents the monolayer operators from layer *l* = ± at momentum **p** and ${{\bf{K}}}_{{\rm{K}}}^{l}$ is the K-point momentum of layer *l*.

Distinctly, in M-point moiré materials, the **Q**-vectors form a kagome lattice, as shown in Fig. 1c. To be specific, the moiré operators in layer *l*—which, for the present case, include only an extra spin *s* = *↑*,*↓* index—are related to the monolayer ones according to ${\widehat{c}}_{{\bf{k}},{\bf{Q}},s,l}^{\dagger }={\widehat{c}}_{{C}_{3z}^{\eta }{{\bf{K}}}_{{\rm{M}}}^{l}+{\bf{k}}-{\bf{Q}},s,l}^{\dagger }$, for ${\bf{Q}}\in {{\mathcal{Q}}}_{\eta +l}$, in which ${{\bf{K}}}_{{\rm{M}}}^{l}$ is the momentum of the monolayer M point. The three *C*_3*z*_-related valleys indexed by *η* = 0, 1 and 2 are implicitly encoded by the kagome sublattice to which **Q** belongs: the valley-*η* fermions are supported on the ${{\mathcal{Q}}}_{\eta \pm 1}$ sublattices (in which *η* + *l* is taken modulo 3), as derived in Supplementary Information Section IV. As we will show, the kagome **Q**-lattice leads to substantially different properties of M-point moiré materials.

## Materials realizations

**Fig. 2:**
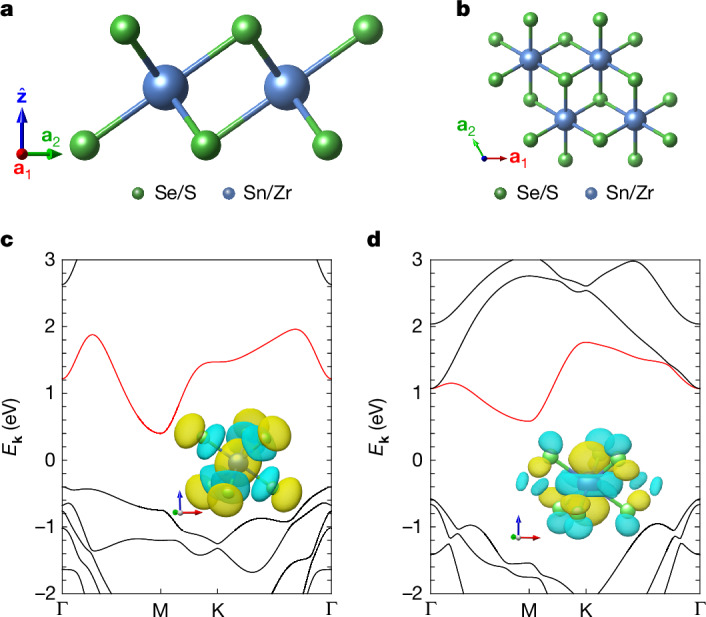
Exfoliable monolayers for M-point moiré materials. **a**,**b**, Side and top views of the crystal structures of 1T-SnSe_2_ and 1T-ZrS_2_. **c**,**d**, The ab initio band structures for SnSe_2_ and ZrS_2_, respectively. The lowest spinful conduction band, with minima at the M points, is highlighted in red, whereas the Wannier orbitals contributing to the low-energy states are shown as insets. The yellow (blue) colours correspond to the positive (negative) sign of the orbitals.

We now turn to 1T-SnSe_2_ and 1T-ZrS_2_ as experimentally exfoliable monolayers for realizing M-point moiré heterostructures (see Supplementary Information Section II). The monolayer crystal structure of both materials is shown in Fig. 2a,b and belongs to the $P\bar{3}m1{1}^{{\prime} }$ group, which is generated by translations, *C*_3*z*_ rotations, in-plane twofold rotations *C*_2*x*_, inversion ${\mathcal{I}}$ and time-reversal ${\mathcal{T}}$ symmetries. The Sn (Zr) atoms form a triangular lattice, with the Se (S) atoms being located at the other *C*_3*z*_-invariant Wyckoff positions above and below the Sn (Zr) plane. The ab initio band structures of monolayer SnSe_2_ and ZrS_2_ shown in Fig. 2c,d reveal two insulators for which the conduction band minimum is located at the M point. The first isolated Kramers-degenerate conduction band of SnSe_2_ is atomic, being spanned by an effective *s*-like molecular orbital centred on the Sn atom. For ZrS_2_, the low-energy M-point states are contributed primarily by the ${d}_{{z}^{2}}$ orbitals of Zr.

## Moiré Hamiltonians

Because the SnSe_2_ and ZrS_2_ monolayers lack twofold out-of-plane rotation symmetry (*C*_2*z*_), there are two distinct ways to stack and subsequently twist them by an angle *θ* to achieve a large-scale moiré periodicity. In the so-called AA-stacking configuration, the top (*l* = +1) and bottom (*l* = −1) layers are stacked directly on top of each other and then twisted by the layer-dependent angle $\frac{l\theta }{2}$. By contrast, for AB-stacking, the bottom layer is first rotated by 180° around the $\widehat{{\bf{z}}}$ axis, before applying the $\frac{l\theta }{2}$ twist. As discussed in Supplementary Information Section III, the two configurations have different crystalline symmetries. Although both stackings feature *C*_3*z*_ and ${\mathcal{T}}$ symmetries, they differ in the direction of the in-plane twofold rotation symmetry: the AA (AB)-stacking arrangement has *C*_2*x*_ (*C*_2*y*_) symmetry.

We perform large-scale ab initio calculations (which include relaxation effects) at commensurate twist angles 13.17° ≥ *θ* ≥ 3.89° (see Methods and Supplementary Information Sections III and VIII) and construct two types of moiré Hamiltonian model for each angle and stacking configuration according to the method outlined in Supplementary Information Section IX. The first is a numerically exact model, which accurately reproduces a large set of spinful bands (at least the first five in each valley) in both energy and wavefunction. The second is an analytical approximate continuum model capturing the dispersion and wavefunction of the first or first two (depending on the angle) lowest-energy spinful gapped bands (and, qualitatively, the higher-energy spectrum) in each valley. The comprehensive results at all angles are presented in Supplementary Information Section XI. Unlike the case of Γ-point or K-point twisting, ab initio simulations are crucial for obtaining even the correct qualitative moiré Hamiltonian. The two-centred first-monolayer harmonic approximation incorrectly predicts continuous translation symmetry along one direction (for example, along the ${C}_{3z}^{\eta }\widehat{{\bf{y}}}$ direction in valley *η*) and an overall gapless spectrum, as shown in Supplementary Information Section VI.

**Fig. 3:**
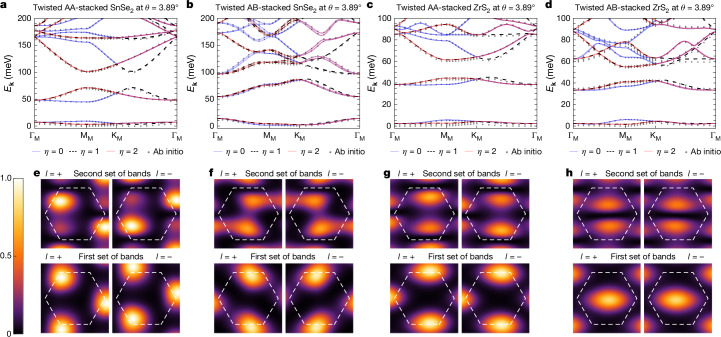
Ab initio results for M-point moiré SnSe_2_ and ZrS_2_. **a**–**d**, Band structures for AA-stacked (**a**,**c**) and AB-stacked (**b**,**d**) twisted SnSe_2_ and ZrS_2_ at the commensurate angle *θ* = 3.89°. Both the ab initio and valley-resolved continuum model band structures are shown. **e**–**h**, The layer-resolved CDD corresponding to the first and second sets of spinful bands in valley *η* = 0. The Wigner–Seitz unit cell is indicated by the dashed hexagon.

Figure 3 summarizes the ab initio results for twisted AA-stacked and AB-stacked SnSe_2_ and ZrS_2_ at low twist angle. Both stacking configurations exhibit approximate spin SU(2) symmetry (see Supplementary Information Section IX) and feature two sets of spinful gapped bands in each of the three *C*_3*z*_-related valleys, as shown in Fig. 3a–d. The lowest-energy set of bands has a narrow bandwidth of around 10 meV. The charge density distribution (CDD) for the lowest two bands in valley *η* = 0, shown in Fig. 3e–h, reveals that these moiré systems have approximate spatial symmetries beyond the exact valley-preserving *C*_2*x*_ and *C*_2*y*_ symmetries expected in the AA-stacked and AB-stacked configurations, respectively. For instance, the CDD of the first set of spinful bands in AA-stacked SnSe_2_, as well as the first two sets of bands in twisted ZrS_2_, feature an approximate twofold rotation symmetry (the second set of spinful bands in AA-stacked SnSe_2_ exhibits this symmetry to a lesser extent). In the AB-stacked configuration, the centre of the approximate *C*_2*z*_ symmetry aligns with the unit cell origin, whereas in the AA-stacked case, the effective ${\widetilde{C}}_{2z}$ rotation centre is shifted away from the unit cell origin and will be specified below. Moreover, the CDD suggests the presence of an approximate in-plane mirror symmetry, ${\widetilde{M}}_{z}$. These effective symmetries (whose origin is explained below and in Supplementary Information Section VB) prompt us to construct simplified analytical continuum models that can capture and explain these features.

**Fig. 4:**
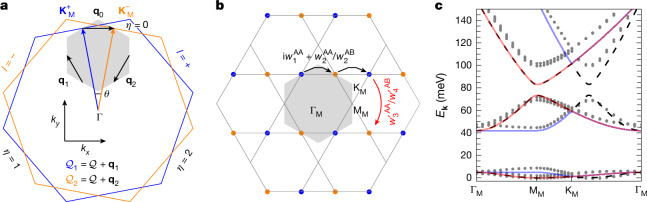
Analytical continuum M-point moiré models. **a**, Relationship between the monolayer and moiré BZs, with the coloured and grey hexagons representing the respective BZs. **b**, Generation of the ${\bf{Q}}$-lattice in the *η* = 0 valley, showing the hopping terms of the moiré potential matrix ${T}_{{\bf{Q}},{{\bf{Q}}}^{{\prime} }}$. **c**, The band structure of the simplified moiré model for AA-stacked SnSe_2_ at *θ* = 3.89°. The colour scheme matches that of Fig. 3.

In valley *η* = 0, the simplified M-point moiré Hamiltonian can be expressed as

1 $$\begin{array}{l}{[{h}_{{\bf{Q}},{{\bf{Q}}}^{{\prime} }}({\bf{k}})]}_{sl;{s}^{{\prime} }{l}^{{\prime} }}\,=\,{\delta }_{{\bf{Q}},{{\bf{Q}}}^{{\prime} }}{\delta }_{s{s}^{{\prime} }}{\delta }_{l{l}^{{\prime} }}\left[\frac{{({k}_{x}-{Q}_{x})}^{2}}{2{m}_{x}}+\frac{{({k}_{y}-{Q}_{y})}^{2}}{2{m}_{y}}\right]\\ \,\,\,+{[{T}_{{\bf{Q}},{{\bf{Q}}}^{{\prime} }}]}_{sl;{s}^{{\prime} }{l}^{{\prime} }}\,{\rm{for}}\,{{\bf{Q}}}^{({\prime} )}\in {{\mathcal{Q}}}_{{l}^{({\prime} )}},\end{array}$$

in which *m*_*x*_ and *m*_*y*_ are the anisotropic effective masses of SnSe_2_ and ZrS_2_ (see Methods). As shown in Fig. 4a,b, the moiré potential takes the form of a hopping model on two of the three sublattices of the kagome M-point **Q**-lattice. Explicitly, the simplified Hermitian moiré potential tensor exhibits spin SU(2) symmetry and includes only interlayer terms, given by ${[{T}_{{\bf{Q}},{{\bf{Q}}}^{{\prime} }}^{{\rm{AA}}}]}_{ls;(-l)s}=(\pm {\rm{i}}{w}_{1}^{{\rm{AA}}}+{w}_{2}^{{\rm{AA}}}){\delta }_{{\bf{Q}}\pm {{\bf{q}}}_{0},{{\bf{Q}}}^{{\prime} }}+{w}_{3}^{{\prime} {\rm{AA}}}{\delta }_{{\bf{Q}}\pm ({{\bf{q}}}_{1}-{{\bf{q}}}_{2}),{{\bf{Q}}}^{{\prime} }}$ and ${[{T}_{{\bf{Q}},{{\bf{Q}}}^{{\prime} }}^{{\rm{AB}}}]}_{ls;(-l)s}={w}_{2}^{{\rm{AB}}}{\delta }_{{\bf{Q}}\pm {{\bf{q}}}_{0},{{\bf{Q}}}^{{\prime} }}+{w}_{4}^{{\prime} {\rm{AB}}}{\delta }_{{\bf{Q}}\pm ({{\bf{q}}}_{1}-{{\bf{q}}}_{2}),{{\bf{Q}}}^{{\prime} }}$. The interlayer hopping parameters, obtained by fitting to the ab initio band structure, are listed in Methods. The band structure of the simplified model for AA-stacked SnSe_2_ is shown in Fig. 4c, indicating excellent qualitative agreement with the ab initio results for such a small number of parameters. In the simplified models, for both SnSe_2_ and ZrS_2_, the overlap between the fitted and ab initio bands is larger than 95% (85%) with the first (second) set of spinful bands, as we show in Supplementary Information Section XI.

## Momentum-space non-symmorphic symmetries

The approximate symmetries inferred from the layer-resolved CDD of the M-point moiré Hamiltonian are exact symmetries in the simplified moiré models from equation (1) (see detailed discussion in Supplementary Information Section VI). Specifically, the centre of the effective twofold rotation symmetry ${\widetilde{C}}_{2z}$ for the AA-stacked Hamiltonian is located at $\frac{{{\bf{q}}}_{\eta }}{|{{\bf{q}}}_{\eta }{|}^{2}}\arg ({\rm{i}}{w}_{1}^{{\rm{AA}}}+{w}_{2}^{{\rm{AA}}})$ in valley *η*. By contrast, the simplified AB-stacked moiré Hamiltonian exhibits *C*_2*z*_ symmetry, with its rotation centre aligned with the origin of the moiré unit cell. Because both models are effectively spinless (owing to atomistic arguments presented in Supplementary Information Section XI) and exhibit either ${\widetilde{C}}_{2z}{\mathcal{T}}$ or ${C}_{2z}{\mathcal{T}}$ symmetry in each valley, the Berry curvature of any gapped set of bands is exactly zero. Consequently, the first two sets of bands of both the AA-stacked and AB-stacked moiré Hamiltonians are topological trivial and, hence, Wannierizable. This is also consistent (and the result of) the bands being flat and exhibiting a large (40 meV) gap from one another. However, the physics of these Hubbard (with interaction) bands is far from trivial in this system, as shown below.

Unlike the *C*_2*z*_ and ${\widetilde{C}}_{2z}$ symmetries, the effective mirror ${\widetilde{M}}_{z}$ symmetry has an unconventional action on the momentum-space moiré fermions. Specifically, ${\widetilde{M}}_{z}$ acts non-symmorphically in momentum space, with ${\widetilde{M}}_{z}{\widehat{c}}_{{\bf{k}},{\bf{Q}},s,l}^{\dagger }{\widetilde{M}}_{z}^{-1}={\widehat{c}}_{{\bf{k}}+{{\bf{q}}}_{\eta },{\bf{Q}}+{{\bf{q}}}_{\eta },s,-l}^{\dagger }$ for ${\bf{Q}}\in {{\mathcal{Q}}}_{\eta +l}$. Because ${{\bf{q}}}_{0}=\frac{{{\bf{b}}}_{{M}_{1}}}{2}$, the action of ${\widetilde{M}}_{z}$ can only be made conventional by folding the moiré BZ along ${{\bf{q}}}_{\eta }$, which would break the moiré translation symmetry. The non-symmorphic action of the ${\widetilde{M}}_{z}$ symmetry originates from the moiré fermions realizing a projective representation of the symmetry group of the system. Letting ${T}_{{{\bf{a}}}_{{M}_{1,2}}}^{{\prime} }$ denote the two moiré translation operators for valley *η* = 0 along the direct moiré lattice vectors ${{\bf{a}}}_{{M}_{1,2}}$ (with ${{\bf{a}}}_{{M}_{i}}\cdot {{\bf{b}}}_{{M}_{j}}=2\pi {\delta }_{ij}$), we find that $[{T}_{{{\bf{a}}}_{{M}_{2}}}^{{\prime} },{\widetilde{M}}_{z}]=\{{T}_{{{\bf{a}}}_{{M}_{1}}}^{{\prime} },{\widetilde{M}}_{z}\}=0$ (contrasting with a conventional mirror *M*_*z*_ symmetry, which would commute with both ${T}_{{{\bf{a}}}_{{M}_{1}}}^{{\prime} }$ and ${T}_{{{\bf{a}}}_{{M}_{2}}}^{{\prime} }$).

It is important to note that the effective ${\widetilde{M}}_{z}$ symmetry is not accidental. In the AA-stacked case, it can be shown to hold exactly for arbitrary moiré harmonics within the local-stacking approximation^46^. In the limit of vanishing twist angle (*θ* → 0), the moiré Hamiltonian can be constrained by the exact symmetries of the untwisted bilayer configuration. The inversion symmetry of the untwisted AA-stacked bilayer gives rise to the ${\widetilde{M}}_{z}$ symmetry of the moiré Hamiltonian, as shown in Supplementary Information Sections VB and VI. In the AB-stacked case, the true in-plane mirror symmetry of the untwisted bilayer leads to an effective inversion symmetry $\widetilde{{\mathcal{I}}}$ of the corresponding moiré Hamiltonian, which also acts non-symmorphically in momentum space. In the simplified AB-stacked model, the approximate *C*_2*z*_ symmetry, combined with the $\widetilde{{\mathcal{I}}}$ symmetry, leads to an ${\widetilde{M}}_{z}={C}_{2z}\widetilde{{\mathcal{I}}}$ symmetry of the system.

**Fig. 5:**
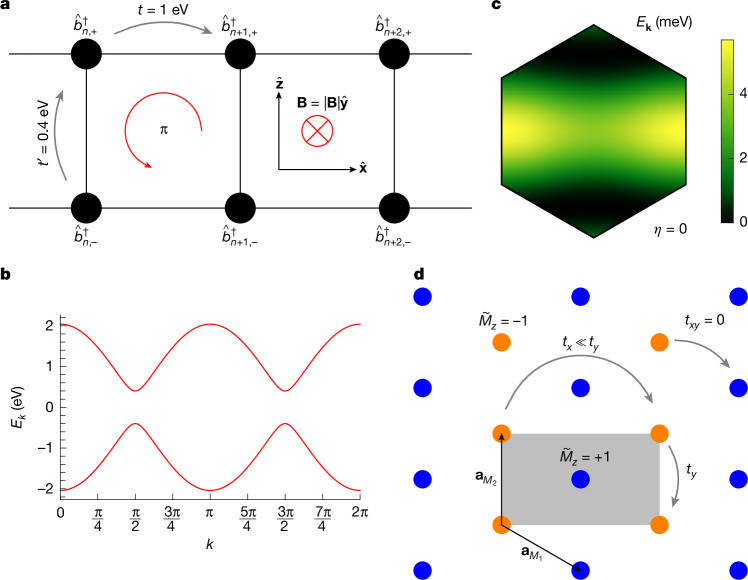
Momentum-space non-symmorphic symmetries. **a**, A ladder tight-binding model with magnetic flux that realizes the ${\widetilde{M}}_{z}$ symmetry. Fermion operators and hopping amplitudes are indicated above each site (black dots). **b**, Dispersion relation. **c**, Energy dispersion of the first band of AA-stacked SnSe_2_ at *θ* = 3.89° in the first moiré BZ. **d**, Schematic illustration of the corresponding quasi-one-dimensional character of atomic bands in M-point moiré systems for valley *η* = 0. Each Wannier orbital (dots) is coloured according to its ${\widetilde{M}}_{z}$ eigenvalue. The grey rectangle represents the rectangular unit cell of each ${\widetilde{M}}_{z}$ symmetry sector.

Projective fermion representations that realize momentum-space non-symmorphic symmetries have previously been proposed in magnetic systems^42^ or systems subjected to a large magnetic field (on the order of thousands of Tesla)^40,41,47^. M-point moiré materials provide the first experimentally viable realization of these symmetries in any (that is, magnetic or non-magnetic) system. To better understand the origin of the momentum-space non-symmorphic action of the ${\widetilde{M}}_{z}$ symmetry, we construct a simple one-dimensional tight-binding model that incorporates it. The resulting ladder model, shown in Fig. 5a, mimics the dispersion of an atomic band in the M-point moiré Hamiltonian for valley *η* = 0 along the $\widehat{{\bf{x}}}$ direction (see Supplementary Information Section VI). Each unit cell is threaded by a uniform perpendicular magnetic field, enclosing a π-flux. Because π-flux and (−π)-flux are equivalent, the model also respects time-reversal and ${\widetilde{M}}_{z}$ symmetry. In the Fourier-transformed basis ${\widehat{b}}_{k,l}^{\dagger }=\frac{1}{\sqrt{N}}{\sum }_{n}{\widehat{b}}_{n,l}^{\dagger }{{\rm{e}}}^{{\rm{i}}kn}$, the ${\widetilde{M}}_{z}$ symmetry acts non-symmorphically as ${\widetilde{M}}_{z}{\widehat{b}}_{k,l}^{\dagger }{\widetilde{M}}_{z}^{-1}={\widehat{b}}_{k+\pi ,-l}^{\dagger }$, ensuring that the spectra of the Hamiltonian at *k* and *k* + π are identical, as shown in Fig. 5b.

## Hubbard and Luttinger simulators

Within each valley, the first two sets of spinful bands in SnSe_2_ and ZrS_2_ bilayers are individually Wannierizable, with their bandwidths tunable by adjusting the twist angle. Given the excellent SU(2) symmetry, these M-point moiré systems become effective simulators of the Hubbard model when Coulomb interactions are included (H. Hu et al., to be published). However, owing to the extra valley degree of freedom, these systems go beyond the single-band U(2) Hubbard model, instead realizing a six-flavour U(2) × U(2) × U(2) Hubbard model.

Another key distinction from the standard Hubbard model can arise from the ${\widetilde{M}}_{z}$ symmetry. In real space, ${\widetilde{M}}_{z}$ does not change the position along the moiré heterostructure. As a result, the continuum moiré Hamiltonian can be made diagonal in the ${\widetilde{M}}_{z}$ basis. Because ${({T}_{{{\bf{a}}}_{{M}_{1}}}^{{\prime} })}^{2}{({T}_{{{\bf{a}}}_{{M}_{2}}}^{{\prime} })}^{-1}$ and ${T}_{{{\bf{a}}}_{{M}_{2}}}^{{\prime} }$ both commute with ${\widetilde{M}}_{z}$, each mirror sector of valley *η* = 0 will feature reduced translation symmetry specified by the rectangular lattice vectors $2{{\bf{a}}}_{{M}_{1}}-{{\bf{a}}}_{{M}_{2}}$ and ${{\bf{a}}}_{{M}_{2}}$. The ${T}_{{{\bf{a}}}_{{M}_{1}}}^{{\prime} }$ operator anticommutes with ${\widetilde{M}}_{z}$, exchanging the two mirror sectors. The Wannier orbitals of any atomic band—such as the first conduction band of AA-stacked SnSe_2_ from Fig. 5c—can therefore be split by their ${\widetilde{M}}_{z}$ eigenvalues: the orbitals of each mirror sector are displaced by ${{\bf{a}}}_{{M}_{1}}$ and form two interpenetrating rectangular lattices shown in Fig. 5d. Within each mirror sector and in valley *η* = 0, the interorbital separation is larger by a factor of $\sqrt{3}$ along the $\widehat{{\bf{x}}}$ direction compared with the $\widehat{{\bf{y}}}$ one. Provided that the Wannier orbital spread is approximately isotropic (as it happens for the first band of AA-stacked SnSe_2_ but not in the first band of twisted ZrS_2_), this will lead to reduced hopping along $\widehat{{\bf{x}}}$ compared with $\widehat{{\bf{y}}}$ (see Supplementary Information Section VI). As the tunnelling between ${\widetilde{M}}_{z}$ sectors is forbidden, the system in each valley will behave quasi-one-dimensionally, with flatter dispersion along the ${C}_{3z}^{\eta }\widehat{{\bf{x}}}$ direction, effectively emulating a Luttinger model. In the three-valley system, this quasi-one-dimensional behaviour causes the U(2) × U(2) × U(2) local moments to couple differently along three *C*_3*z*_-related directions, similar (but not identical) to the couplings of the Kitaev model^45^.

We note, however, that quasi-one-dimensionality along the ${C}_{3z}^{\eta }\widehat{{\bf{y}}}$ direction (that is, flatter dispersion along the ${C}_{3z}^{\eta }\widehat{{\bf{x}}}$ direction) is not an inherent or universal feature of M-point moiré materials. Instead, it is the presence of the effective ${\widetilde{M}}_{z}$ symmetry, not previously identified, that plays a more general role. Together with approximately isotropic Wannier orbitals for the bands, the effective ${\widetilde{M}}_{z}$ symmetry can enforce one-dimensional behaviour in the single-particle valley-projected moiré Hamiltonian. However, this symmetry is also compatible with two-dimensional physics in general (see Methods). For instance, because of the elongated Wannier orbitals, twisted ZrS_2_ exhibits excellent effective ${\widetilde{M}}_{z}$ symmetry, but its first set of conduction bands is not quasi-one-dimensional along the ${C}_{3z}^{\eta }\widehat{{\bf{y}}}$ direction.

## Discussion

We have introduced a new platform for moiré materials based on monolayers with triangular lattices, in which the low-energy states are located at the M points of the BZ. The presence of three *C*_3*z*_-related valleys makes M-point moiré materials manifestly different from pre-existing Γ-point and K-point twisted heterostructures. We have shown that M-point moiré materials can be realized in many materials^38,48^ and specifically in twisted 1T-SnSe_2_ and 1T-ZrS_2_, both of which are experimentally exfoliable. By constructing the corresponding moiré Hamiltonians, we have shown that these materials provide the first experimentally viable example of momentum-space non-symmorphic symmetry in a non-magnetic system. The projective representations of the crystallographic space groups associated with these symmetries extend beyond present theoretical frameworks^49^, opening new avenues for discovering symmetry-protected topological phases.

When electron–electron interactions are considered, twisted SnSe_2_ and ZrS_2_ bilayers can realize strongly correlated, tunable six-flavour Hubbard models. As well as exhibiting Mott physics and correlated insulating phases at integer fillings, these systems can spontaneously break the ${\widetilde{M}}_{z}$ symmetry, potentially giving rise to various stripe phases, which will be explored in future work (H. Hu et al., to be published). Notably, we find that the multivalley Wannier model for AA-stacked SnSe_2_ admits exact solutions in the strong-coupling limit, under the experimentally justified assumption of weak spin-valley U(6) symmetry breaking in the interaction Hamiltonian. At integer fillings 0 ≤ *ν* ≤ 6 of the lowest six flat bands, the corresponding ground states include classical spin liquids at *ν* = 1 and *ν* = 5, valence bond solids at *ν* = 2 and *ν* = 4 and a quantum spin liquid at *ν* = 3 (H. Hu et al., to be published). The perfect nesting at momentum **q**_*η*_ in valley *η*, enforced by the ${\widetilde{M}}_{z}$ symmetry, further enhances the potential for new correlated phases, as does the recently introduced quantum nesting condition^50^, satisfied as a result of the same symmetry. Moreover, owing to their quasi-one-dimensional nature within each valley, these materials are promising candidates for exploring Luttinger physics.

## Methods

### First-principles calculation

The ab initio calculations were performed using the Vienna ab initio Simulation Package (VASP)^51–55^ and OpenMX^56–59^. The lattice relaxation was carried out in two stages: first, the twisted structures were ‘pre-relaxed’ using a machine learning force field (MLFF) trained with NequIP^60^ and DPmoire^61^; second, a further relaxation step was conducted using VASP until the force on each atom was less than 0.01 eV Å^−1^. van der Waals interactions were included using the DFT-D2 method of Grimme^62^ for SnSe_2_ and the DFT-D3 method of Grimme et al.^63^ for ZrS_2_, based on benchmarking with bulk structures (details provided in Supplementary Information Section III). A vacuum slab larger than 15 Å was applied along the *z*-direction to eliminate any artificial layer interactions.

The exchange-correlation energy functional within the generalized gradient approximation as parameterized by Perdew et al.^64^ was used in the VASP calculations. The calculations were carried out on a 2 × 2 × 1 **k**-mesh for *θ* = 13.17° and *θ* = 9.43° and on a 1 × 1 × 1 mesh for smaller twist angles *θ*. The energy cut-off for the plane-wave basis is 288 eV (337 eV) for SnSe_2_ (ZrS_2_). Larger energy cut-offs have been tested to have negligible influence on the band structures. Furthermore, the ab initio Hamiltonians in the atomic orbital basis, used for valley projection and continuum model construction, were generated using OpenMX^56–59^. In these calculations, we used the 2019 version of optimized numerical pseudo-atomic orbitals, specifically Sn7.0-s2p2d2 and Se7.0-s2p2d2 for SnSe_2_ and Zr7.0-s2p2d2 and S7.0-s2p2d2 for ZrS_2_.

### First-principles results for twisted bilayers

AA-stacked twisted bilayers are obtained by aligning the two layers directly on top of one another and rotating them by a small relative angle *θ*. By contrast, the AB-stacked configurations are formed by rotating the layers by a relative angle of 180° − *θ*. The AA-stacked and AB-stacked configurations exhibit *P*3211′ and *P*3121′ symmetries, respectively. Both space groups include *C*_3*z*_ and ${\mathcal{T}}$ as symmetry generators, but *P*3211′ also features *C*_2*x*_, whereas *P*3121′ includes *C*_2*y*_. In this work, we primarily focus on the AA-stacked configuration of twisted SnSe_2_ and ZrS_2_, with further details as well as results for AB-stacked heterostructures provided in Supplementary Information Section III.

**Extended Data Fig. 1:**
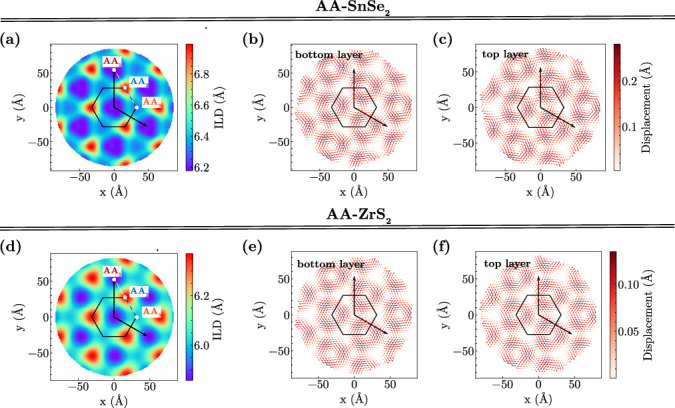
Ab initio lattice relaxation results for twisted AA-stacked SnSe_2_ and ZrS_2_ at *θ* = 3.89°. **a**–**c**, For twisted SnSe_2_: interlayer distance (ILD) (**a**); intralayer atom displacements in the bottom (**b**) and top (**c**) layers. **d**–**f**, Same as **a**–**c** but for twisted ZrS_2_. The definitions of the *C*_3*z*_-symmetric local regions labelled by AA_*i*_ (for 1 ≤ *i* ≤ 3) are given in Supplementary Information Section III.

Large-scale ab initio calculations were performed for twisted SnSe_2_ and ZrS_2_ bilayers using the methodology outlined above. The relaxed structures for SnSe_2_ and ZrS_2_ at a twist angle *θ* = 3.89° are shown in Extended Data Fig. 1, highlighting both interlayer and intralayer relaxation effects. For SnSe_2_, the interlayer distance varies by approximately 0.8 Å and the maximum intralayer displacement is about 0.3 Å. By contrast, ZrS_2_ exhibits smaller variations, with interlayer distance changes of approximately 0.6 Å and intralayer displacements up to 0.15 Å. These findings underscore the marked lattice relaxation effects in both materials, which are crucial for the energetics of the moiré potential and the resulting band dispersion.

**Extended Data Fig. 2:**
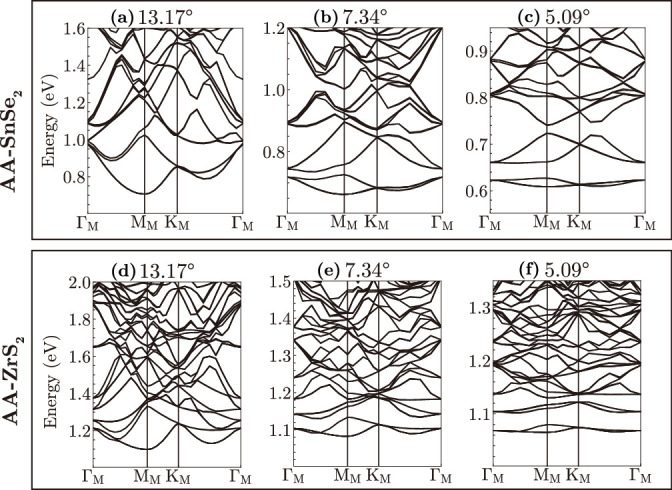
The ab initio band structures of twisted AA-stacked SnSe_2_ and ZrS_2_ bilayers for twist angles 13.17° ≥ *θ* ≥ 5.09°. The twist angle is indicated above each panel. **a**–**c**, Moiré conduction bands of twisted SnSe_2_. **d**–**f**, Band structure of twisted ZrS_2_. In both cases, the lowest group of conduction bands, isolated from other energy bands, consists of six bands originating from the three inequivalent M valleys of the monolayer.

Using the fully relaxed bilayer structures, we calculated the moiré band structures for SnSe_2_ and ZrS_2_. The band structures for *θ* = 3.89° are shown in Fig. 3, with further results for larger twist angles presented in Extended Data Fig. 2. The moiré Hamiltonian reveals one or two sets of isolated conduction bands, each consisting of six spinful bands originating from the three *C*_3*z*_-related M valleys. Each valley contributes two nearly degenerate bands owing to the approximate SU(2) symmetry. As the twist angle decreases, the moiré bands become increasingly flat, with the bandwidth of the lowest set narrowing to just several meV. Further details are provided in Supplementary Information Section III.

### Constructing faithful continuum models

**Extended Data Fig. 3:**
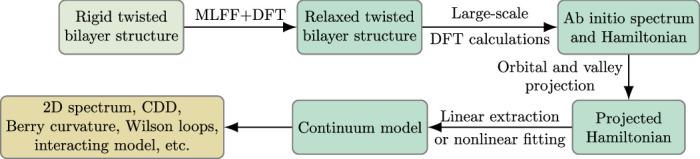
Workflow used for constructing faithful continuum models .

The workflow used in this work for constructing faithful continuum models for twisted SnSe_2_ and ZrS_2_ bilayers is summarized schematically in Extended Data Fig. 3. The process begins with a rigid twisted bilayer structure at a chosen commensurate twist angle *θ*. This rigid structure is relaxed using a combination of machine learning force field and density functional theory (DFT), as detailed in Supplementary Information Section III. The relaxed structure is then used for large-scale DFT calculations to obtain the ab initio spectrum (through VASP) and the corresponding tight-binding Kohn–Sham Hamiltonian in an atomic orbital basis (through OpenMX).

Next, the ab initio Hamiltonian is projected onto the relevant orbitals and valleys to derive a low-dimensional effective Hamiltonian at a small set of **k**-points within the moiré BZ, as described in Supplementary Information Section VIII. Simultaneously, a symmetry-based parameterization of the moiré Hamiltonian is constructed symbolically, as explained in Supplementary Information Sections IV, V and VII. The parameters of this model are determined through either linear extraction or nonlinear fitting, as detailed in Supplementary Information Section IX. The final output is an analytic, accurate and faithful continuum moiré Hamiltonian for the corresponding heterostructure.

With these continuum models, we can compute various spectral properties of the moiré system, including the band structures across the full moiré BZ, the CDD of the isolated bands, their Berry curvature, Wilson loops and interacting tight-binding models, among others. Further details are provided in Supplementary Information Section XI and H. Hu et al. (to be published). For the simple continuum models shown in equation (1) for twisted SnSe_2_ and ZrS_2_ at *θ* = 3.89°, the parameters are listed in Extended Data Table 1.

### Other M-point moiré materials

As well as 1T-SnSe_2_ (refs. ^65–69^) and 1T-ZrS_2_ (refs. ^70–72^) studied in this work, a wide range of other experimentally exfoliable monolayers offer promising platforms for M-point moiré heterostructures, as listed in ref. ^38^. These include materials with structures similar to 1T-SnSe_2_ and 1T-ZrS_2_, such as 1T-ZrSe_2_ (refs. ^71,72^), 1T-SnS_2_ (refs. ^73,74^), 1T-HfSe_2_ (ref. ^75^) and 1T-HfS_2_ (refs. ^76–78^). Also, GaTe (ref. ^79^), which has a different crystalline structure, further expands this moiré ‘universality class’.

### Emergence of quasi-one-dimensionality

**Extended Data Fig. 4:**
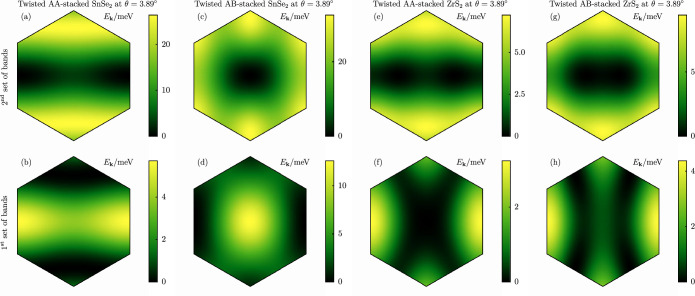
Small-angle band structures for M-point moiré SnSe_2_ and ZrS_2_. **a**–**d**, Results for SnSe_2_. **e**–**h**, Results for ZrS_2_. Each panel shows the dispersion of a gapped set of conduction bands in valley *η* = 0 across the first moiré BZ from Fig. 4b, with the band minimum set to zero for clarity. Columns represent different stacking configurations (AA-stacked and AB-stacked) and different monolayers and rows show either the first or the second set of conduction bands. For each band, the results are averaged over the two approximately SU(2)-degenerate bands.

In the main text, we showed that quasi-one-dimensional physics can emerge in M-point moiré materials. However, we also highlighted that quasi-one-dimensionality along the ${C}_{3z}^{\eta }\widehat{{\bf{y}}}$ direction, as observed in AA-stacked twisted SnSe_2_, is neither a generic nor a fundamental feature of M-point moiré systems. To further illustrate this, Extended Data Fig. 4 shows the dispersion of the first two sets of conduction bands for the four monolayer-stacking configurations considered in this work.

Starting with AA-stacked twisted SnSe_2_, Extended Data Fig. 4a,b shows that the two gapped conduction bands are nearly dispersionless along the ${C}_{3z}^{\eta }\widehat{{\bf{x}}}$ direction in valley *η*. This observation aligns with the general argument presented in the main text and arises from the combined effects of the effective ${\widetilde{M}}_{z}$ symmetry and the approximately isotropic shape of the Wannier orbitals associated with these bands. Notably, this dispersion asymmetry is opposite to what would be expected from the effective monolayer masses of SnSe_2_, as given in Extended Data Table 1: *m*_*y*_ > *m*_*x*_ would generally favour flatter dispersion along the ${C}_{3z}^{\eta }\widehat{{\bf{y}}}$ direction rather than along the ${C}_{3z}^{\eta }\widehat{{\bf{x}}}$ direction. However, in the case of AA-stacked SnSe_2_, the difference between *m*_*y*_ and *m*_*x*_ is not substantial (${m}_{y}{\not\gg}{m}_{x}$), allowing the effect of ${\widetilde{M}}_{z}$ symmetry to dominate and promote quasi-one-dimensional behaviour along the ${C}_{3z}^{\eta }\widehat{{\bf{y}}}$ direction (with flatter dispersion along the ${C}_{3z}^{\eta }\widehat{{\bf{x}}}$ direction).

In AB-stacked SnSe_2_, Extended Data Fig. 4c,d shows that the bands are not quasi-one-dimensional. This behaviour arises because of relatively larger relaxation effects, which worsen the validity of the local-stacking approximation for this monolayer and stacking configuration. Because the effective ${\widetilde{M}}_{z}$ and $\widetilde{{\mathcal{I}}}$ symmetries rely on the local-stacking approximation, this heterostructure does not exhibit strong ${\widetilde{M}}_{z}$ symmetry. Among the four heterostructures considered in this work, AB-stacked SnSe_2_ shows the smallest overlap between the ab initio wavefunctions and those computed from the fitted effective model with enforced ${\widetilde{M}}_{z}$ symmetry. Consequently, the system does not feature quasi-one-dimensional behaviour.

For the first conduction band of either AA-stacked or AB-stacked twisted ZrS_2_, shown in Extended Data Fig. 4f,h, the greatly enhanced monolayer mass asymmetry (*m*_*y*_ ≫ *m*_*x*_) or, equivalently, the real-space orbitals elongated along the ${C}_{3z}^{\eta }\widehat{{\bf{x}}}$ direction—as illustrated in Fig. 3g,h—leads to a flatter dispersion along the ${C}_{3z}^{\eta }\widehat{{\bf{y}}}$ direction for the first set of conduction bands. This occurs despite the system exhibiting excellent ${\widetilde{M}}_{z}$ symmetry, which is even stronger than that of AA-stacked SnSe_2_. For the second set of bands of twisted ZrS_2_, shown in Extended Data Fig. 4e,g, the orbitals have nearly equal spread along the ${C}_{3z}^{\eta }\widehat{{\bf{x}}}$ and ${C}_{3z}^{\eta }\widehat{{\bf{y}}}$ directions. Consequently, our argument, which incorporates both the ${\widetilde{M}}_{z}$ symmetry and the orbital shape, holds, resulting in flatter dispersion along the ${C}_{3z}^{\eta }\widehat{{\bf{x}}}$ direction.

These results demonstrate that quasi-one-dimensionality is not a generic feature of M-point moiré materials but instead depends on both symmetry and energetic considerations. The relevant symmetry is compatible with behaviours opposite to those proposed in ref. ^80^, as observed in twisted ZrS_2_. Specifically, the mass-enforced band flattening, also identified in BC_3_ (ref. ^81^), in which *m*_*y*_ ⋙ *m*_*x*_, occurs in the opposite direction to that predicted by ref. ^80^. Our momentum-space non-symmorphic ${\widetilde{M}}_{z}$ symmetry emerges as the unifying principle that reconciles these seemingly contradictory emergent behaviours. This symmetry, along with the nature of the constituent orbitals, plays a crucial role in shaping the interacting Hamiltonian, ultimately determining the emergence of one-dimensional or two-dimensional physics (H. Hu et al., to be published).

All of these findings underscore the importance of performing detailed ab initio calculations. As discussed in the main text, without such precise insights, simplified models—such as those using the two-centred first-monolayer harmonic approximation^82^—produce incorrect results. Specifically, they predict a continuous translational symmetry of the system along the ${C}_{3z}^{\eta }\widehat{{\bf{y}}}$ direction in valley *η* and an overall gapless spectrum, neither of which are observed in the four monolayer and stacking configurations analysed in this work.

### Wannier model for AA-stacked twisted SnSe_2_

For AA-stacked twisted SnSe_2_, the system develops topologically trivial, isolated conduction bands for each valley and spin. H. Hu et al. (to be published) construct the Wannier orbitals and derive the corresponding interacting models for this system. The Wannier orbitals for each valley *η*, denoted by ${\widehat{d}}_{{\bf{R}},\eta ,s}^{\dagger }$ for unit cell ${\bf{R}}\in {\mathbb{Z}}{{\bf{a}}}_{{M}_{1}}+{\mathbb{Z}}{{\bf{a}}}_{{M}_{2}}$ and spin *s*, form a triangular lattice. The positions of the Wannier orbitals within each unit cell for all three valleys are nearly identical.

The tight-binding model is expressed as

2 $${H}_{{\rm{t}}}=\sum _{{\bf{R}},\Delta {\bf{R}}}{t}_{\Delta {\bf{R}}}^{\eta }{\hat{d}}_{{\bf{R}},\eta ,s}^{\dagger }{\hat{d}}_{{\bf{R}}+\Delta {\bf{R}},\eta ,s},$$

in which, owing to the emergent ${\widetilde{M}}_{z}$ symmetry, the hopping becomes quasi-one-dimensional. The dominant hopping terms are

3 $${t}_{\Delta {\bf{R}}}^{\eta }=t{\delta }_{\Delta {\bf{R}},\pm {C}_{3z}^{\eta }{{\bf{a}}}_{{M}_{2}}}.$$

The dominant interaction term is the on-site Hubbard repulsion, which takes the form

4 $${H}_{U}=\sum _{{\bf{R}},\Delta {\bf{R}}}\frac{{U}_{\eta {\eta }^{{\prime} }}}{2}{n}_{{\bf{R}},\eta }{n}_{{\bf{R}},{\eta }^{{\prime} }},$$

in which ${n}_{{\bf{R}},\eta }={\sum }_{s}{\widehat{d}}_{{\bf{R}},\eta ,s}^{\dagger }{\widehat{d}}_{{\bf{R}},\eta ,s}$ is the density operator associated with unit cell **R** and valley *η*.

## Online content

Any methods, additional references, Nature Portfolio reporting summaries, source data, extended data, supplementary information, acknowledgements, peer review information; details of author contributions and competing interests; and statements of data and code availability are available at 10.1038/s41586-025-09187-5.

## Supplementary information

Supplementary information Supplementary Figs. 1–133, discussion (including detailed DFT results for monolayers and bilayers, derivation and symmetry analysis of moiré Hamiltonians with and without gradient terms, fitting procedures for continuum models, analytical and numerical band structure results and a comprehensive summary of effective models) and Supplementary Tables 1–26.

Supplementary files including the M-point moiré Hamiltonians are in Mathematica format.

## Figures and Tables

**Extended Data Table 1:**
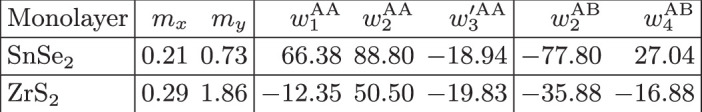
Parameters of the simple moiré models for twisted SnSe_2_ and ZrS_2_ at *θ* = 3.89°

The effective masses are given in units of the bare electron mass *m*_e_, whereas the hopping amplitudes are given in meV.

## Data Availability

All data generated in this study are included in the main text and the Supplementary Information. The continuum models for the M-point moiré materials derived in this work are available online as a supplementary file. Further data, along with any code required for reproducing the figures, are available from the authors on reasonable request.
