# Supplementary material for: Moiré materials based on M-point twisting
Source: Nature. 2025 Jul 9;643(8071):376–81. doi: 10.1038/s41586-025-09187-5 (PMC12240816; doi:10.1038/s41586-025-09187-5)
Supplement: Supplementary file 1 — Supplementary Figs. 1–133, discussion (including detailed DFT results for monolayers and bilayers, derivation and symmetry analysis of moiré Hamiltonians with and without gradient terms, fitting procedures for continuum models, analytical and numerical band structure results and a comprehensive summary of effective models) and Supplementary Tables 1–26. [file 41586_2025_9187_MOESM1_ESM.pdf]

nature portfolio

https://doi.org/10.1038/s41586-025-09187-5

Supplementary information

Moiré materials based on M-point twisting

In the format provided by the authors and unedited

Supplementary Information for "Moiré materials based on M-point twisting"

Dumitru Călugăru,[1,2,*] Yi Jiang,[3,*] Haoyu Hu,[3,*] Hanqi Pi,[3,4,*] Jiabin Yu,[5,1]
Maia G. Vergniory,[6,7,3] Jie Shan,[8,9,10] Claudia Felser,[11,†] Leslie M. Schoop,[12]
Dmitri K. Efetov,[13,14] Kin Fai Mak,[8,9,10] and B. Andrei Bernevig[1,3,15,‡]

[1]*Department of Physics, Princeton University, Princeton, New Jersey 08544, USA*
[2]*Rudolf Peierls Centre for Theoretical Physics, University of Oxford, Oxford OX1 3PU, United Kingdom*
[3]*Donostia International Physics Center, P. Manuel de Lardizabal 4, 20018 Donostia-San Sebastián, Spain*
[4]*Beijing National Laboratory for Condensed Matter Physics, and Institute of Physics, Chinese Academy of Sciences, Beijing 100190, China*
[5]*Department of Physics, University of Florida, Gainesville, FL, USA*
[6]*Département de physique et Institut quantique, Université de Sherbrooke, Sherbrooke J1K 2R1 QC, Canada*
[7]*Regroupement Québécois sur les Matériaux de Pointe (RQMP), Quebec H3T 3J7, Canada*
[8]*Max Planck Institute for the Structure and Dynamics of Matter, Hamburg, Germany*
[9]*School of Applied and Engineering Physics and Department of Physics, Cornell University, Ithaca, NY 14850, USA*
[10]*Kavli Institute at Cornell for Nanoscale Science, Ithaca, NY 14850, USA*
[11]*Max Planck Institute for Chemical Physics of Solids, Nöthnitzer Str. 40, Dresden 01187, Germany*
[12]*Department of Chemistry, Princeton University, Princeton, NJ 08540*
[13]*Faculty of Physics, Ludwig-Maximilians-University Munich, Munich 80799, Germany*
[14]*Munich Center for Quantum Science and Technology (MCQST), Ludwig-Maximilians-University Munich, Munich 80799, Germany*
[15]*IKERBASQUE, Basque Foundation for Science, Bilbao, Spain*

CONTENTS

I. Introduction to the supplementary information 4

II. First-principles results for monolayer materials 4
  A. Monolayer $SnSe_2$ 4
    1. Symmetries 5
    2. Hamiltonian for the lowest conduction band and effective $\mathbf{k}\cdot\mathbf{p}$ model at the M valley 7
  B. Monolayer ZrS_2 7

III. First-principles results for bilayer materials 8
  A. Bilayer $SnSe_2$ 9
    1. Untwisted bilayer $SnSe_2$ 9
    2. Twisted bilayer $SnSe_2$ 9
  B. Bilayer ZrS_2 13
    1. Untwisted bilayer ZrS_2 13
    2. Twisted bilayer ZrS_2 13

IV. Single-particle Bistritzer-MacDonald models for twisted $SnSe_2$ and ZrS_2 bilayers 15
  A. Deriving a BM model for twisted $SnSe_2$ and ZrS_2 16
    1. AA-stacking configuration 16
    2. AB-stacking configuration 18
  B. Constraining the interlayer hopping amplitude with symmetries 20
    1. Constraints arising from the $\theta \neq 0$ symmetries 21
    2. Constraints arising from the $\theta = 0$ symmetries 21
  C. The twisted bilayer model and its exact symmetries 22
    1. Model in momentum space 23
    2. Model in real space 26
    3. Exact symmetries of the model 26

* These authors contributed equally to this work.
† claudia.felser@cpfs.mpg.de
‡ bernevig@princeton.edu

V. Direct general derivation of the moiré potential without gradient terms 27
A. General form of the moiré potential restricted by the exact $\theta \neq 0$ symmetries of the heterostructure 28
B. General form of the moiré potential restricted by the approximate $\theta = 0$ symmetries of the heterostructure 29
1. Hamiltonian for the untwisted configuration 29
2. Hamiltonian for the twisted configuration 31
3. Constraining the moiré potential 34

VI. Additional symmetries of the first moiré harmonic model in different limits 34
A. Additional symmetries in the zero-twist limit 35
1. Momentum-space non-symmorphic symmetries in both the AA- and AB-stacking configurations 35
2. Even-odd basis of the AA-stacked moiré Hamiltonian 37
3. Toy model for momentum-space non-symmorphic symmetry 38
4. Connecting the toy model and AA-stacked moiré Hamiltonian 40
B. The C_{2z} symmetric limit 41
C. The $\mathrm{SU}(2)$ symmetric limit 41
D. Additional symmetries in the two-center first monolayer harmonic model 42
E. Additional symmetries of the simplified three-parameters models 42
1. The three-parameter AA-stacked model 42
2. The three-parameter AB-stacked model 44
F. Summary of symmetries in different limits 45

VII. Direct general derivation of the moiré potential including gradient terms 45
A. General form of the moiré Hamiltonian with gradient terms 45
B. Restricting the generalized moiré potential using the exact $\theta \neq 0$ symmetries of the heterostructure 47
C. Restricting the generalized moiré potential using the approximate $\theta = 0$ symmetries of the heterostructure 48
1. Moiré Hamiltonian in the local-stacking approximation with gradient terms 48
2. Further simplification in the zero-twist limit 50
3. Constraining the generalized moiré potential in the local-stacking approximation 51

VIII. First-principles results for twisted bilayer materials with valley projection 52
A. Löwdin orthogonalization 52
1. Obtaining the refined trial states 53
2. The Löwdin orthogonalization in a non-orthonormal basis 55
B. Obtaining the plane-wave moiré Hamiltonians from *ab initio* simulations 56
1. The Wannier projection step 56
2. The valley projection step 57
3. Alternation in the mapping of M points between the monolayer and moiré BZs 58
C. Valley-projected band structure and charge density of $SnSe_2$ 59
D. Valley-projected band structure and charge density of ZrS_2 62
E. Effects of lattice relaxation 62

IX. Obtaining continuum model from *ab initio* simulations 63
A. Notation 63
B. Linear Extraction 64
1. Low-energy restriction 64
2. Partial low-energy restriction 65
C. Nonlinear fitting 66
1. Analytical expression for the gradient of the cost function 67
D. Reducing the number of parameter 68

X. Analytical results for the moiré continuum Hamiltonian 68
A. Analytical results at high-symmetry momentum points 68
1. Γ_M point 69
2. K'_M point 70
3. M'_M point 71
4. Extending to the two-parameter AB-stacked model 72
B. Extracting parameters 72

1. Energy gaps 72
2. Wave functions 73

XI. Models fitted to *ab initio* simulations 74
A. Overview of the results 74
1. Types of models constructed 74
2. Overlaps, zero-twist symmetries, SU (2) symmetry 77
3. Berry curvature, CDD, and Wilson loops 77
B. Discussion 78
C. Parameter values 80
1. Full models 80
2. First moiré harmonic models 81
D. Numerical results 86
1. Band structures along high-symmetry lines 86
2. Spectra of the first gapped conduction bands 94
3. Wilson loops of the first gapped conduction bands along $\mathbf{b}_{M_1}$ 106
4. Wilson loops of the first gapped conduction bands along $\mathbf{b}_{M_2}$ 114

References 121

I. INTRODUCTION TO THE SUPPLEMENTARY INFORMATION

The supplementary information provides a self-contained, comprehensive, and pedagogical presentation of our results and the techniques employed in this work. To guide the reader through these materials, we offer an overview of each section below.

In Section II, we begin with detailed *ab initio* results for the 1T-$SnSe_2$ and 1T-ZrS_2 monolayers (referred to hereafter as $SnSe_2$ and ZrS_2, respectively). This section discusses the crystal structure, symmetries, and low-energy physics of these materials. We then turn to their homobilayer heterostructures in Section III, presenting first-principles results for both untwisted and twisted bilayers. Key results include band structures, lattice relaxation profiles, Wilson loops, and a comparison of different van der Waals functionals.

In Section IV, we shift to a symmetry-based analytical approach, deriving the moiré Hamiltonian for twisted $SnSe_2$ and ZrS_2 bilayers. We start by obtaining a Bistritzer-MacDonald model [1] for the twisted heterostructures, using a two-center, first-monolayer harmonic approximation for the interlayer hopping amplitude. We then introduce the notation for the low-energy moiré Hamiltonian and analyze the exact crystalline symmetries of the corresponding model. Section V generalizes these results by deriving the most general moiré potential without gradient terms (*i.e.*, terms proportional to gradients of the low-energy fermionic fields) using the exact symmetries of the twisted heterostructure. An explicit symmetry-obeying parameterization of the moiré potential up to the first moiré harmonic is also provided. Additionally, we show how the moiré potential can be further constrained by the symmetries of the untwisted heterostructure in the so-called "local-stacking approximation" [2]. The limit in which these constraints are imposed is referred to as the zero-twist limit.

Section VI explores additional effective symmetries of the moiré Hamiltonian that emerge under various physically relevant limits. A key finding is the presence of momentum-space nonsymmorphic symmetries in the zero-twist limit without gradient terms, and this section examines their implications in detail. Section VI further investigates the symmetries arising in other limits of the moiré Hamiltonian. Finally, it analyzes the symmetries of simplified two- and three-parameter models, which, as shown in Section XI, provide an excellent description of the lowest bands of the twisted heterostructures at small angles.

For completeness, Section VII derives the most general form of the moiré potential with gradient terms, and shows how these latter terms are further constrained in the zero-twist limit. The resulting generalized moiré Hamiltonian (away from the zero-twist limit) will then be used in Section XI to build numerically exact models for the first few moiré bands. Returning to *ab initio* methods, Section VIII details the extraction of valley-projected moiré Hamiltonians in the plane wave basis, starting from the corresponding first-principles Kohn-Sham Hamiltonians. This section also provides elementary results for the valley-projected Hamiltonians, including their *ab initio* band structures and charge density distribution at the smallest commensurate angle studied.

Section IX is a technical section that outlines the algorithms used to obtain continuum models from the *ab initio* Hamiltonians. We present both a linear least-squares extraction method, adapted from Refs. [3–5], as well as a nonlinear fitting one. Strategies to minimize the number of parameters in these methods are also discussed. Section X then shows how analytical band structure calculations can be performed for the M-point moiré plane-wave Hamiltonians. The strategy employed is similar to the "tripod" [1] and related [6], plane-wave truncation schemes used for twisted bilayer graphene.

Finally, building on the concepts introduced beforehand, Section XI offers a comprehensive summary of our findings. This section begins by presenting both numerically exact and simplified moiré Hamiltonian models for all monolayers, stacking configurations, as well as all the commensurate angles studied. It provides the parameter values for these models and compares their band structures, layer-resolved charge density distribution, Berry curvature, and Wilson loops, delivering a detailed and cohesive perspective on the twisted heterostructure physics investigated in this work.

II. FIRST-PRINCIPLES RESULTS FOR MONOLAYER MATERIALS

In this section, we present *ab initio* results for the monolayer materials considered in this work, namely $SnSe_2$ and ZrS_2. For each material, we discuss the crystal structure, the symmetries, and their density functional theory (DFT) band structures, and additionally provide $\mathbf{k}\cdot\mathbf{p}$ Hamiltonians around the M point of the monolayer Brillouin zone (BZ).

A. Monolayer $SnSe_2$

The crystal structure and band dispersion for single-layer $SnSe_2$ are shown in Fig. S1. Monolayer $SnSe_2$ crystallizes in the 1T structure within the symmetry group $P\bar{3}m11'$ [Shubnikov Space Group (SSG) 164.86], as shown in Figs. S1(a)

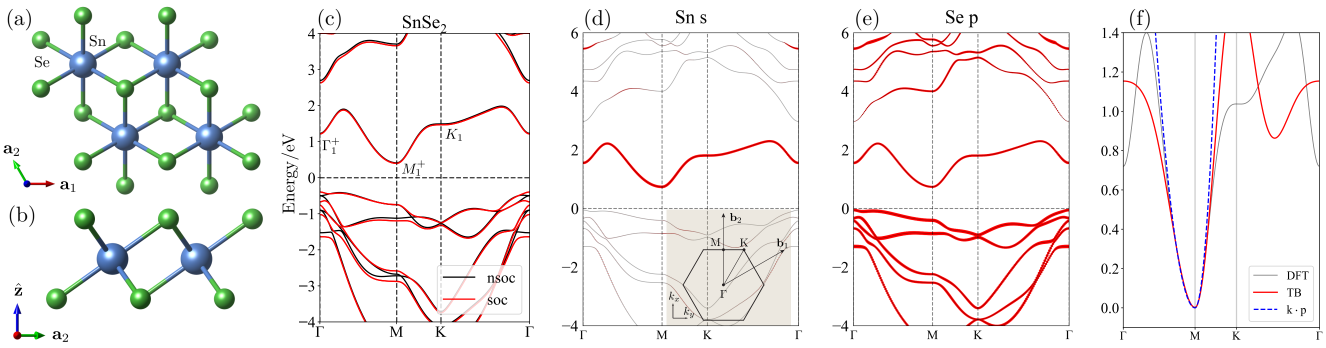

FIG. S1. *Ab initio* results for monolayer $SnSe_2$. (a) and (b) show the crystal structure of monolayer $SnSe_2$, from top and side views, respectively. (c) shows the *ab initio* band structure of monolayer $SnSe_2$, with the black (red) lines denoting the bands without (with) spin-orbital coupling (SOC). The (spinless) IRREP of the M valley is marked on the plot. (d) and (e) show the orbital weights of the s orbital of Sn and the p orbitals of Se. The inset in (d) shows the monolayer BZ, high-symmetry momenta and reciprocal lattice vectors. The bottom conduction band is isolated with the minimum at the M point, and is contributed mainly by the s orbitals of Sn and the p orbitals of Se. (f) Comparison of the dispersion from the DFT result, minimal TB model defined in Eq. (S2.15), and the $\mathbf{k}\cdot\mathbf{p}$ model from Eq. (S2.16).

and S1(b). The lattice vectors are given by

$$\mathbf{a}_1 = a\,(1,0)\,, \quad \mathbf{a}_2 = a\left(-\frac{1}{2},\frac{\sqrt{3}}{2}\right), \tag{S2.1}$$

with a being the lattice constant and the atoms being located at

$$\text{Sn}: \ (0,0,0)\,, \quad \text{Se}: \ \left(\frac{1}{3},\frac{2}{3},z\right), \left(\frac{2}{3},\frac{1}{3},-z\right). \tag{S2.2}$$

In Eq. (S2.2), the first two coordinates are written in the unit cell basis $\mathbf{a}_{1,2}$, with $a = 3.811\,\text{Å}$ and $z = 1.528\,\text{Å}$[7]. The Sn atom is located at the $1a$ Wyckoff position, which features $\bar{3}m$ site symmetry (with inversion), while the Se or S atoms are positioned at $2b$ Wyckoff position, which has $3m$ symmetry (without inversion). Within a unit cell, the two $2b$ Wyckoff positions are mapped to one another by inversion. The reciprocal lattice vectors of the single-layer system are given by

$$\mathbf{b}_1 = \frac{2\pi}{a}\left(1,\frac{1}{\sqrt{3}}\right), \quad \mathbf{b}_2 = \frac{2\pi}{a}\left(0,\frac{2}{\sqrt{3}}\right), \tag{S2.3}$$

such that $\mathbf{a}_i\cdot\mathbf{b}_j = 2\pi\delta_{ij}$, for $1 \leq i,j \leq 2$.

The band structure and corresponding orbital projections of monolayer $SnSe_2$ are shown in Figs. S1(c) to S1(e). The bottom conduction band is well-isolated and mainly contributed by the s orbital of Sn and the p orbitals of Se, all of which have negligible spin-orbital coupling (SOC). The conduction band minimum (CBM) appears at M point, and transforms as the $\bar{M}_3\bar{M}_4$ double irreducible representation (IRREP) in the spinful case.

The isolated bottom conduction band is characterized by the $\bar{\Gamma}_4\bar{\Gamma}_5$, $\bar{M}_3\bar{M}_4$, and $\bar{K}_4\bar{K}_5$ (or Γ_1^+, M_1^+, and K_1 in the spinless case) IRREPs at the three high-symmetry momenta, which correspond to the elementary band representation (EBR) [8–10] induced by ${}^1\bar{E}_g^2\bar{E}_2@1a$ (or $A_{1g}@1a$ in the spinless case). This EBR is therefore induced by a (spinful) s orbital located at the $1a$ position and, as such, allows us to construct a Wannier tight-binding model of the bottom spinful conduction band which features a single effective s orbital at the Sn site ($1a$ position). This is also confirmed by the orbital weights shown Figs. S1(d) and S1(e), which indicate that the bottom conduction band is mainly contributed by the s orbital of Sn and p orbitals of Se. The latter form molecular orbitals surrounding the Sn atoms which, from a symmetry standpoint, behave as effective s orbitals.

1. Symmetries

The symmetry group of monolayer $SnSe_2$ is generated by two-dimensional translations, time-reversal symmetry $\mathcal{T}$, inversion $\mathcal{I} = \{-1|0\,0\,0\}$, in-plane three-fold rotation $C_{3z} = \left\{3^+_{001}|0\,0\,0\right\}$, and out of plane two-fold rotation

$C_{2x} = \{2_{100}|0\,0\,0\}$. We now consider the action of these symmetries on the effective spinful s orbital located at the $1a$ Wyckoff position which spans the bottom isolated conduction band. We let $\hat{a}^\dagger_{\mathbf{R},s}$ be the electron creation operator corresponding to the effective s orbital in unit cell $\mathbf{R}$ having spin $s = \uparrow, \downarrow$ (and which is located at the unit cell origin). The corresponding momentum space operators are defined according to

$$\hat{a}^\dagger_{\mathbf{k},s} = \frac{1}{\sqrt{N}} \sum_{\mathbf{R}} \hat{a}^\dagger_{\mathbf{R},s} e^{i\mathbf{k}\cdot\mathbf{R}}. \tag{S2.4}$$

Consider g to be a certain (unitary or antiunitary) symmetry operation whose action on a real space vector $\mathbf{r}$ is given by $g\mathbf{r} = R\mathbf{r}$, where R denotes the rotation part of the transformation g. On the $\hat{a}^\dagger_{\mathbf{R},s}$ fermions, the transformation g is implemented simply as

$$g\hat{a}^\dagger_{\mathbf{R},s_1} g^{-1} = \sum_{s_2} \left[D^{\mathrm{sl}}(g)\right]_{s_2 s_1} \hat{a}^\dagger_{g\mathbf{R},s_2}, \tag{S2.5}$$

where the representation matrices $D^{\mathrm{sl}}(g)$ for the various symmetries of the model are given explicitly by

$$D^{\mathrm{sl}}(\mathcal{T}) = is_y, \quad D^{\mathrm{sl}}(C_{3z}) = e^{-\frac{\pi i}{3}s_z}, \quad D^{\mathrm{sl}}(C_{2x}) = -is_x, \quad D^{\mathrm{sl}}(\mathcal{I}) = s_0, \tag{S2.6}$$

with s_0, s_x, s_y, and s_z denoting the identity and Pauli matrices in the spin subspace. In momentum space, the action of g reads as

$$g\hat{a}^\dagger_{\mathbf{k},s_1} g^{-1} = \sum_{s_2} \left[D^{\mathrm{sl}}(g)\right]_{s_2 s_1} \hat{a}^\dagger_{g\mathbf{k},s_2} \tag{S2.7}$$

where

$$g\mathbf{k} = \begin{cases} R\mathbf{k} & \text{if } g \text{ is unitary} \\ -R\mathbf{k} & \text{if } g \text{ is antiunitary} \end{cases}. \tag{S2.8}$$

Despite the system featuring spin-orbit coupling (SOC), the isolated bottom conduction band exhibits an *effective* spin SU (2) symmetry. As we show below, such an *effective* SU (2) symmetry can always be defined for a two-band system with $\mathcal{IT}$ symmetry (but this *effective* SU (2) symmetry does not generalize to systems with a larger number of orbitals). We will also show that the breaking of the *global* SU (2) symmetry (which is defined in the original atomic orbital basis) remains weak in the case of $SnSe_2$, due to the weak SOC associated with the effective s orbital, primarily contributed by the Sn s and Se p orbitals.

First, we consider the most general Hamiltonian featuring a single spinful band

$$h(\mathbf{k}) = \sum_n h_n(\mathbf{k}) s_n, \tag{S2.9}$$

where $h_n(\mathbf{k})$ are real functions of momentum $\mathbf{k}$. The Hamiltonian has eigenvalues $h_0(\mathbf{k}) \pm \sqrt{\sum_{n=x,y,z} h_n(\mathbf{k})}$. In a system with $\mathcal{IT}$ symmetry, the Hamiltonian $h(\mathbf{k})$ obeys $h(\mathbf{k}) = s_x h^*(\mathbf{k}) s_x$, which immediately implies that $h_n(0)$ (for $n = x, y, z$) rendering all bands doubly degenerate in spin, and resulting in an *effective* SU (2) symmetry. It is important to note that this effective SU (2) symmetry is defined in the effective Wannier basis of the two-band model (*i.e.*, the basis of $h(\mathbf{k})$) and may differ from the *global* SU (2) symmetry.

To evaluate the global SU (2) symmetry breaking, we consider the projector into an isolated set of bands with wave function $|\psi_{n\mathbf{k},\alpha,s}\rangle$, where n, α, and s are the band, orbital, and spin indices, respectively

$$P_{\mathbf{k},\alpha,s,\beta,s'} = \sum_n |\psi_{n\mathbf{k},\alpha,s}\rangle \langle\psi_{n\mathbf{k},\beta,s'}|. \tag{S2.10}$$

The spin-symmetric part of the projector is defined as

$$P^{\mathrm{sym}}_{\mathbf{k},\alpha,s,\beta,s'} = \delta_{ss'} \frac{1}{2} \sum_{s''} P_{\mathbf{k},\alpha,s'',\beta,s''} \tag{S2.11}$$

while the spin-antiymmetric part is

$$P^{\mathrm{asym}}_{\mathbf{k},\alpha,s,\beta,s'} = P_{\mathbf{k},\alpha,s,\beta,s'} - P^{\mathrm{sym}}_{\mathbf{k},\alpha,s,\beta,s'} \tag{S2.12}$$

The SU (2) symmetry breaking is defined as the measure of the spin-antisymmetric part

$$\frac{1}{N}\sum_{\mathbf{k}}\frac{\|P_{\mathbf{k}}^{\mathrm{asym}}\|}{\|P_{\mathbf{k}}\|}, \tag{S2.13}$$

where $\|\ldots\|$ denotes the Frobenius norm of a matrix.

To numerically assess the global SU (2) symmetry breaking for the bottom conduction band in monolayer $SnSe_2$, we construct a Wannier tight-binding model using Sn s and Se p orbitals. From this model, we find a 9.0% SU (2) symmetry breaking for the bottom conduction band, leading to the conclusion that the *global* SU (2) symmetry is a good *approximate* symmetry for the bottom conduction band. We reiterate that the bottom conduction band has a perfect *effective* SU (2) symmetry, as discussed around Eq. (S2.9).

2. *Hamiltonian for the lowest conduction band and effective* $\mathbf{k}\cdot\mathbf{p}$ *model at the M valley*

We now build a Wannier model for the lowest conduction band. The tight-binding (TB) Hamiltonian corresponding to the model can be written as

$$\mathcal{H}^{\mathrm{sl}} = \sum_{\mathbf{k}}\left[h^{\mathrm{sl}}(\mathbf{k})\right]_{s_1 s_2}\hat{a}^{\dagger}_{\mathbf{k},s_1}\hat{a}_{\mathbf{k},s_2}. \tag{S2.14}$$

In Eq. (S2.14), the Hamiltonian matrix $h^{\mathrm{sl}}(\mathbf{k})$ can be obtained from Wannier90. The resulting Wannier function, which is also plotted as an inset in Fig. 2(c), is a molecular orbital formed by the s orbital of Sn and the p orbitals of Se and is consistent with the orbital projections in Fig. S1. Its exact expression in terms of the atomic s and p orbitals is straightforward, but beyond the scope of this work.

We also build a simplified TB Hamiltonian with the following form, constrained by $P\bar{3}m11'$ symmetries

$$\begin{aligned} h^{\mathrm{sl}}(\mathbf{k}) = \Bigg[&\epsilon_0 + 2t_1\left(\cos(k_1)+\cos(k_2)+\cos(k_1+k_2)\right) + 2t_2\left(2\cos\left(\frac{3}{2}k_1\right)\cos\left(\frac{1}{2}k_1+k_2\right)+\cos(k_1+2k_2)\right) \\ &+ 2t_3\left(\cos(2k_1)+2\cos(k_1)\cos(k_1+2k_2)\right)\Bigg]s_0, \end{aligned} \tag{S2.15}$$

where s_0 is the two-dimensional identity matrix acting on the spin subspace and $\mathbf{k} = k_1\mathbf{b}_1 + k_2\mathbf{b}_2$. The Hamiltonian matrix $h^{\mathrm{sl}}(\mathbf{k})$ includes an onsite energy ϵ_0, nearest neighbor (NN) t_1, next NN t_2, and fourth NN t_3 hoppings. By fitting to the *ab initio* dispersion, we find $\epsilon_0 = 1.589\,\mathrm{eV}$, $t_1 = 0.029\,\mathrm{eV}$, $t_2 = 0.115\,\mathrm{eV}$, and $t_3 = -0.087\,\mathrm{eV}$. The large long-range hopping is due to the extended nature of the effective s Wannier orbital. The fitted dispersion is shown in Fig. S1(f).

We then build an effective $\mathbf{k}\cdot\mathbf{p}$ Hamiltonian at the M valley. Letting $\delta\mathbf{k}$ denote the momentum deviation from the M point located at $\mathbf{K}_M = \frac{1}{2}\mathbf{b}_2$, the $\mathbf{k}\cdot\mathbf{p}$ expansion of the Hamiltonian matrix from Eq. (S2.14) around the bottom of the conduction band is given by

$$h^{\mathrm{sl}}(\mathbf{K}_M + \delta\mathbf{k}) \approx \left(\frac{\delta k_x^2}{2m_x} + \frac{\delta k_y^2}{2m_y} + \Delta\right)s_0, \tag{S2.16}$$

where m_x and m_y are mass parameters, while Δ is an energy gap parameter that will be set to zero in what follows without loss of generality. By fitting to the *ab initio* band structures, we obtain $m_x = 0.21\,m_e, m_y = 0.73\,m_e$ for $SnSe_2$ at the M valley (*i.e.*, at $\mathbf{k} = \frac{1}{2}\mathbf{b}_2$), where m_e is the electron mass. As shown in Fig. S1(c), the dispersion along the $\Gamma - \mathrm{M}$ line is controlled by m_y and is flatter compared to the dispersion along the $\mathrm{M} - \mathrm{K}$ line, due to the significantly larger value of m_y relative to m_x. A comparison of the dispersion from the DFT, the minimal TB model Eq. (S2.15), and the $\mathbf{k}\cdot\mathbf{p}$ expansion is shown in Fig. S1(f), all of which show excellent agreement near the M valley.

B. Monolayer ZrS_2

The crystal structure of monolayer ZrS_2 is the same as $1T$-$SnSe_2$ as shown in Fig. S1(a), with symmetry group $P\bar{3}m11'$. The lattice constant is $a = 3.661\,\text{Å}$ and the out-of-plane displacement of S atom is $1.441\,\text{Å}$ [11].

In Fig. S2, we show the band structure of monolayer ZrS_2. Compared with $SnSe_2$, where there exists a single (spinful) isolated conduction band above the Fermi level, ZrS_2 has three connected (spinful) conduction bands. These

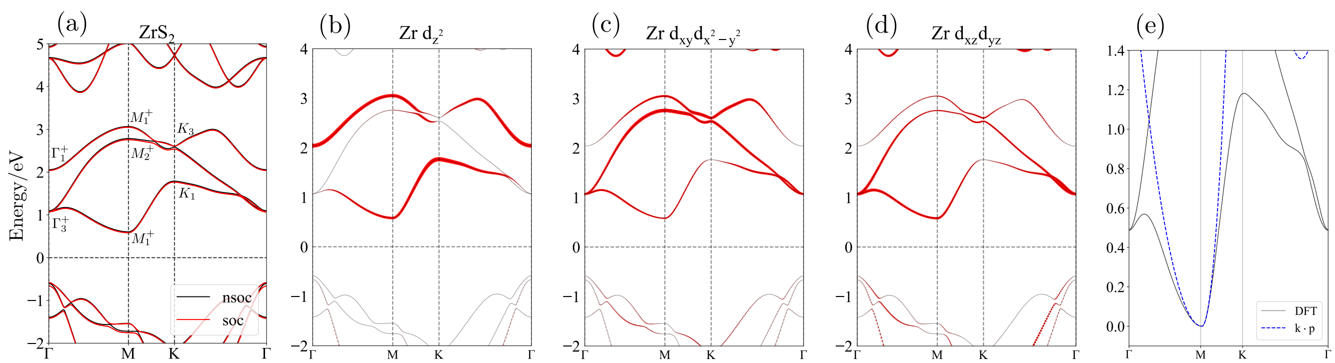

FIG. S2. *Ab initio* results for monolayer ZrS_2. In (a), we compare the bands with (red) and without SOC (black). SOC has only a minor effect on the bands. The (spinless) IRREPs for the three lowest conduction bands are shown. (b)-(d) are the Zr d orbital weights of the bottom three conduction bands. (e) is the comparison between the DFT dispersion and the $\mathbf{k}\cdot\mathbf{p}$ expansion near the M valley.

three bands are mainly contributed by the d orbitals of Zr, with the CBM at M mostly given by the d_{z^2} orbital. From the (spinless) IRREPs shown in Fig. S2(a), the three lowest conduction bands form the EBRs $A_{1g}@1a$ and $E_g@1a$. As shown by the orbital projections of Figs. S2(b) to S2(d), the CBM at M arises from the hybridization of the five d-orbitals of Zr.

To build a low-energy theory of ZrS_2, we can focus exclusively on the states near the CBM at M. We observe that the IRREP of the CBM at M is M_1^+, which is the same as in $SnSe_2$. Note that M_2^+ – the IRREP for the second lowest conduction band at M – has the opposite C_{2x} eigenvalue compared with M_1^+. We then construct an effective molecular s orbital, which we also denote by $\hat{a}^\dagger_{\mathbf{R},s}$, in analogy to the case of $SnSe_2$ discussed around Eq. (S2.4). These effective s orbitals are formed by a linear combination of the five d orbitals of Zr, as can be seen from the orbitals weights at CBM in Figs. S2(b) to S2(d). It is important to note that onsite coupling between d_{z^2} and the other four d orbitals is forbidden, as d_{z^2} form the A_{1g} IRREP while (d_{xz}, d_{yz}) and $(d_{xy}, d_{x^2-y^2})$ form the 2D E_g IRREP under the point group $\bar{3}m$ of $1a$ position. However, off-site coupling is allowed, leading to hybridization at the M point. Their EBR further supports this because both $A_{1g}@1a$ and $E_g@1a$ induce M_1^+ IRREP at M.

The effective s orbitals span the CBM at the M point. Moreover, because $\hat{a}^\dagger_{\mathbf{R},s}$ are effective orbitals located at the $1a$ Wyckoff position, they will have the same symmetry properties as the effective s orbitals defined for $SnSe_2$. As such, the effective $\mathbf{k}\cdot\mathbf{p}$ Hamiltonian describing the bottom of the conduction band of ZrS_2 takes the same form as the one of $SnSe_2$ from Eq. (S2.16). The corresponding masses are given by $m_x = 0.29\, m_e$ and $m_y = 1.86\, m_e$. A comparison of the DFT dispersion and $\mathbf{k}\cdot\mathbf{p}$ expansion is shown in Fig. S2(e), which have good agreement near the M point. The mass along the $\hat{\mathbf{y}}$-direction is significantly larger than that along the $\hat{\mathbf{x}}$-direction, indicating flatter dispersion along the M-Γ direction.

Similar to $SnSe_2$, the lowest conduction band of ZrS_2 at the M valley exhibits an approximate global spin SU (2) symmetry, despite the bands originating from the d orbitals of Zr. Using the global SU (2) symmetry-breaking metric defined in Eq. (S2.13), we find a 12.7% SU (2) symmetry breaking near the M point for the lowest conduction band. At the Γ point of the lowest conduction band, the global SU (2) symmetry breaking reaches 70.1%, primarily due to the weak SOC-induced splitting of the band crossings near Γ. However, being effectively a single band model, Hamiltonian describing the bottom of the conduction band exhibits perfect *effective* SU (2) symmetry, as discussed around Eq. (S2.9).

III. FIRST-PRINCIPLES RESULTS FOR BILAYER MATERIALS

Having presented the *ab initio* results on the monolayer materials in Section II, we now turn to the bilayer case. Specifically, for each single-layer material considered in Section II, this section discusses the symmetries and band structures of the corresponding twisted and untwisted bilayer heterostructures.

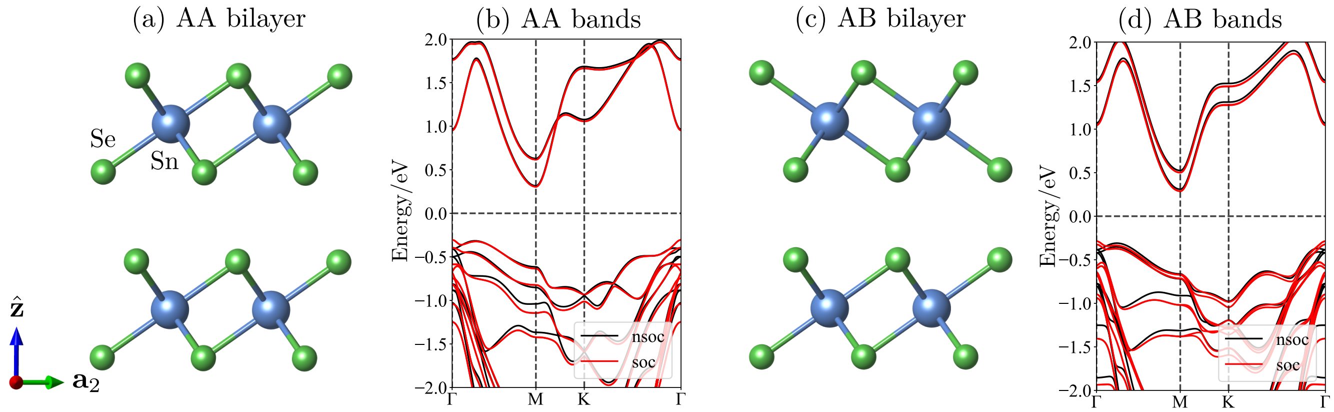

FIG. S3. Crystal and band structures of the untwisted AA- and AB-stacked bilayer $SnSe_2$. (a) illustrates the crystal structure of the AA-stacked bilayer, while (b) shows the band structure with (red lines) and without (black lines) SOC. (c) and (d) are the same as (a) and (b), but for the AB-stacked bilayer.

A. Bilayer $SnSe_2$

In this first section we show results on bilayer $SnSe_2$. Both the twisted and untwisted bilayer heterostructures feature two types of inequivalent stacking patterns, which we refer to as AA- and AB-stacked structures, and which will be defined below in both cases.

1. *Untwisted bilayer* $SnSe_2$

In the untwisted case, the crystal and band structures of the AA- and AB-stacked $SnSe_2$ bilayers are shown in Fig. S3. The symmetry group of the AA-stacked bilayer is given by $P\bar{3}m11'$ (SSG 164.86), while for the AB-stacked one, it is $P\bar{6}m21'$ (SSG 187.210). The symmetry generators in both cases comprise the C_{3z} and $\mathcal{T}$ symmetries. As seen in Figs. S3(a) and S3(c), respectively, the AA-stacked bilayer additionally features inversion symmetry $\mathcal{I}$, while the AB-stacked one is symmetric under z-directional mirror reflections henceforth denoted by M_z. The resulting $\mathcal{IT}$ symmetry of the AA-stacked bilayer enforces a Kramers degeneracy of its bands throughout the entire BZ, as shown in Fig. S3(b). The band structure of the AB-stacked bilayer from Fig. S3(d) does, however, exhibit spin splitting of the valence bands. As they are mainly contributed by the s orbitals of Sn and p orbitals of Se, the bottom four spinful conduction bands of AB-stacked $SnSe_2$ feature negligible SOC and spin splitting. The layer splitting in bilayer $SnSe_2$ is 312 meV for AA-stacking and 213 meV for AB-stacking.

Experimentally, the bulk $SnSe_2$ crystals are mainly reported in the AA-stacked phase [7, 12]. From DFT, the AA-stacked bilayer has 21.6 meV lower total energy than the AB-stacked bilayer, which means the AA-stacked bilayer is more stable thermodynamically. Nevertheless, the metastable AB-stacked configuration can still be experimentally obtained through the tear-and-stack method.

2. *Twisted bilayer* $SnSe_2$

Similarly to $MoTe_2$ [5, 14, 15] (which has a different crystal structure), but unlike graphene [1], $SnSe_2$ does not feature a C_{2z} symmetry around the unit cell origin. As a result, in the twisted bilayer arrangement, just as in the untwisted case, two inequivalent stacking configurations, which we call the AA- and AB-stacked structures, can be constructed, as illustrated in Fig. S4. We denote the two layers by l, where $l = +$ ($l = -$) corresponds to the top (bottom) layer. The two configurations can be obtained as follows:

1. In the AA-stacking configuration shown in Fig. S4(a), the two layers are first stacked *directly* on top of one another. We then rotate layer l in-plane by an angle $\frac{\theta l}{2}$ counterclockwise (with $\theta \geq 0$).

2. In the AB-stacking configuration illustrated in Fig. S4(b), the two layers are again first stacked *directly* on top of one another. However, before twisting the two layers, the bottom layer is rotated by 180° around the $\hat{\mathbf{z}}$

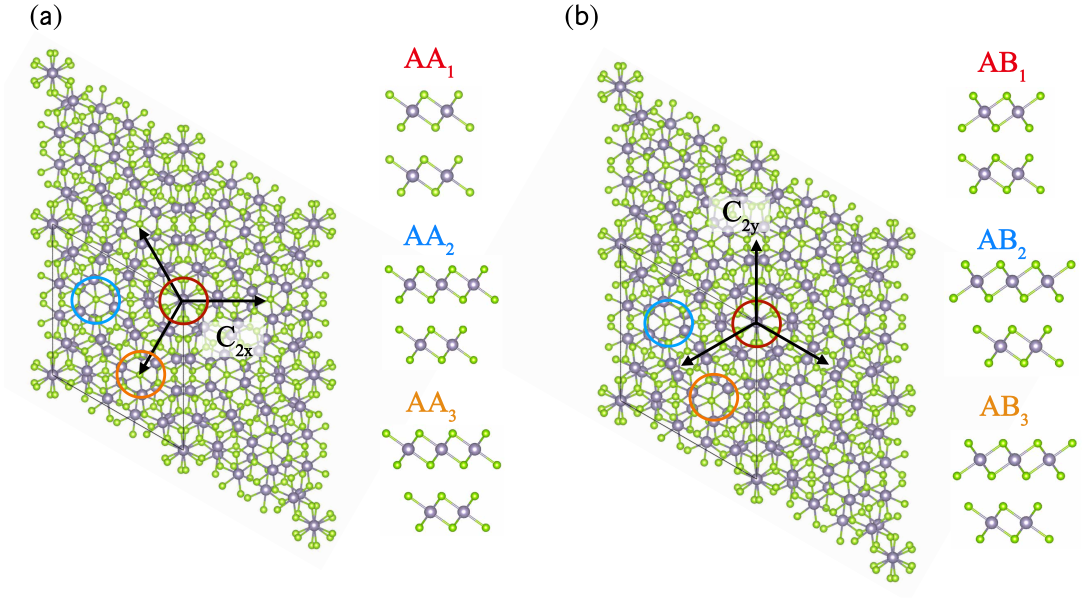

FIG. S4. The crystal structure of the twisted AA- and AB-stacked bilayer $SnSe_2$ and ZrS_2 at 13.17°. The grey spheres represent Zr/Sn atoms, while the green spheres denote Sn/S atoms. The twisted AA-stacked structure is shown in (a) and is symmetric under the $P3121'$ group (SSG 149.22), generated by the C_{3z}, C_{2x}, and $\mathcal{T}$ symmetries, In contrast, the AB-stacked structure illustrated in (b) is symmetric under the $P3211'$ group (SSG 150.26), which is generated by the C_{3z}, C_{2y}, and $\mathcal{T}$ symmetries. For each structure, we use the black arrows to denote the two-fold in-plane rotation axes. Circles of different colors highlight the C_{3z}-symmetric local regions, *i.e.*, the triangular (red) and honeycomb (blue and orange) sites. As the twist angle decreases, the three local configurations are close to the corresponding untwisted bilayer structures shown on the side, where the subscripts 1, 2, and 3 denote three types of inequivalent in-plane shifting in each stacking, as indicated in the inserted subplots. Notably, the AA_2 and AB_1 regions have neighboring Se atoms from both layers aligned at the same in-plane positions, resulting in a larger local interlayer distance (see Fig. S6 for more details).

direction, with the rotation center at the origin point (*i.e.*, the Sn atom). Only then is each layer l in-plane rotated by an angle $\frac{\theta l}{2}$ counterclockwise (with $\theta \geq 0$).

a. ***Symmetry.*** The symmetry groups of the AA- and AB-stacked twisted bilayer $SnSe_2$ are given, respectively by the $P3121'$ (SSG 149.22) and $P3211'$ (SSG 150.26) groups. The point groups of both structures are generated by the C_{3z} and in-plane two-fold symmetry C_2 operations. In the AA-stacked case, the C_2 axis is along the $\hat{\mathbf{x}}$ direction, while in the AB case, the C_2 axis points along the $\hat{\mathbf{y}}$ direction, as indicated by the black arrows in Fig. S4. We use colored circles to denote the C_{3z}-symmetric local regions, *i.e.*, the triangular (red) and honeycomb (blue and orange) sites[1]. As the twist angle decreases, these local structures become similar to the bilayer $AA/AB_{1,2,3}$-stacking configurations shown on the right side and denoted with the same color, where the subscripts 1, 2, and 3 denote three types of inequivalent in-plane shifting in each stacking. In the AA_1 and AB_1 regions, the Sn atoms in both layers are aligned directly on top of each other. In the AA_2 and AB_3 regions, the Sn atoms of the top layer are aligned with the lowermost Se atoms of the bottom layer. Lastly, in the AA_3 and AB_2 regions, the Sn atoms of the top layer are positioned above the uppermost Se atoms of the bottom layer.

b. ***Twisted band structures.*** According to the previous researches on twisted systems, geometric relaxation significantly influences the moiré bands. However, large-scale *ab initio* calculations require substantial computational resources and time. To reduce these costs, we initially pre-relax the twisted structure using a trained machine learning force field (MLFF), which is constructed using NequIP [16] and DPmoire [17]. Subsequently, we further relax the pre-relaxed structure using the Vienna *ab initio* Simulation Package (VASP) [18–22]. During the lattice relaxation process, we use the DFT-D2 method of Grimme [13] to describe the van der Waals (vdW) corrections. This method provides lattice parameters that most closely match experimental results among 21 different vdW corrections, detailed in Figs. S5(a) and S5(b).

In the fully-relaxed twisted AA and AB crystal structures, the interlayer distance (ILD) is defined as the distance between Sn atoms in different layers. As shown in Fig. S6(a), the ILD reaches its maximum in the AA_2 region of the

[1] These C_{3z}-symmetric local regions are the C_{3z}-symmetric Wyckoff position of the moiré unit cell.

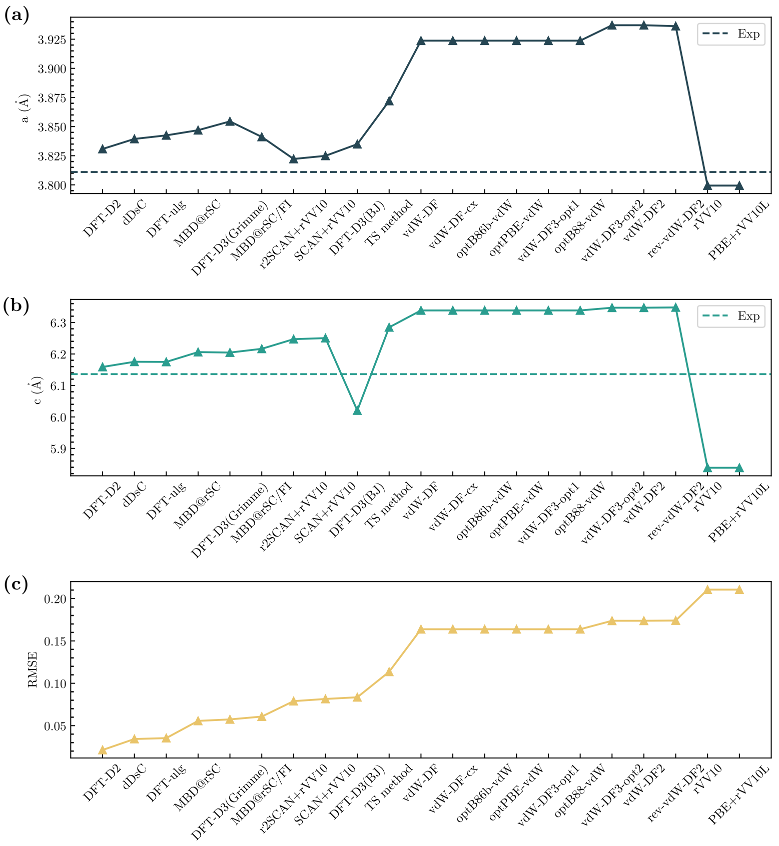

FIG. S5. Comparison of van der Waals (vdW) functionals for describing crystal structures of $SnSe_2$. (a) and (b) show the relaxed lattice constants, while (c) displays the root-mean-square error (RMSE) quantifying the discrepancy between the theoretically predicted lattice constants and the experimentally observed ones for bulk $SnSe_2$, using various vdW functionals implemented in VASP. The experimental values are depicted by dashed lines in (a) and (b), where "TS" refers to the Tkatchenko-Scheffler method. Experimental data is taken from Ref. [7]. We employ the DFT-D2 method of Grimme [13] to account for vdW interactions in $SnSe_2$, as it provides the most accurate reproduction of lattice parameters compared to experimental data.

Bandwidth (meV)	13.17°	9.43°	7.34°	6.01°	5.09°	4.41°	3.89°
AA	318	136	64	33	19	11	7
AB	324	130	61	33	20	15	12

TABLE S1. The bandwidths of the lowest set of conduction bands of twisted AA- and AB-stacked $SnSe_2$ for angles ranging from 13.17° to 3.89°.

twisted AA configuration. This maximum is attributed to the local AA_2 structure, detailed in Fig. S4(a), where the Se atoms from the top layer align directly above the Se atoms from the bottom layer, resulting in increased repulsion compared to the other local configurations. Figs. S6(b) and S6(c) illustrate the intralayer displacements of Sn atoms in the bottom and top layers, respectively, after full relaxation. The atoms rotate around the C_{3z}-symmetric centers, with rotation directions opposite for the top and bottom layers. We also note that the intralayer displacement is largest *around* the regions with large ILD. Figs. S6(d) to S6(f) display the ILD and intralayer displacements for the twisted AB configuration, which exhibit an approximately six-fold symmetric pattern, rather than just the C_{3z}-symmetric pattern observed in the AA configuration. This difference stems from the fact that the AB_2 and AB_3 regions are symmetrically equivalent under C_{2y} and share the same Wyckoff position in the twisted AB configuration. In contrast, the two regions are inequivalent in the twisted AA configuration.

The conduction bands of twisted $SnSe_2$ calculated using VASP are shown in Fig. S7, with the twist angles ranging from 13.17° to 3.89°. The upper row depicts the bands of twisted AA-stacked $SnSe_2$ originating from M valleys of the untwisted bilayer structure. In these bands, we identify one or two sets of isolated moiré bands, each comprising six spinful bands. The moiré bands within each set arise from three inequivalent C_{3z}-related M valleys, where each valley contributes two nearly degenerate bands due to approximate SU (2) symmetry. This degeneracy among different M

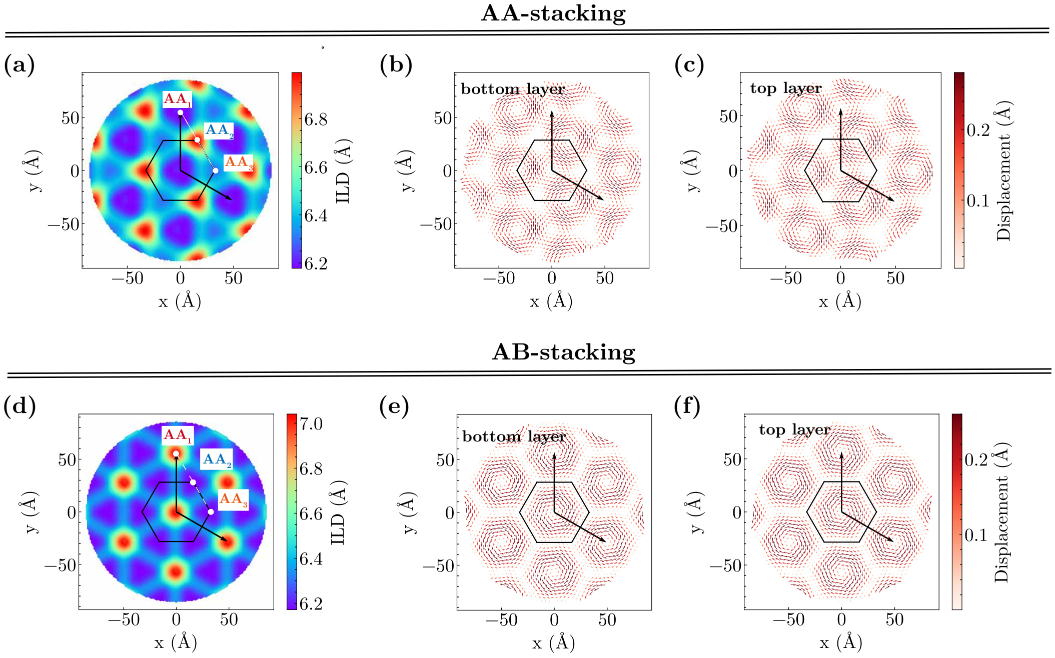

FIG. S6. Lattice relaxation results of 3.89° twisted AA- and AB-stacked $SnSe_2$. (a), (b), and (c) are interlayer distances, the intralayer displacement of the bottom and top layers for the fully-relaxed AA-stacked structure, respectively. (d), (e), and (f) are the same but for the AB-stacked structure. We note that the largest interlayer distance region is AA_2 (AB_1) in the AA-(AB-)stacked case. This agrees with the local configurations because the AA_2 and AB_1 regions have neighboring Se atoms from both layers aligned at the same in-plane positions (see Fig. S4), resulting in a larger interlayer repulsion.

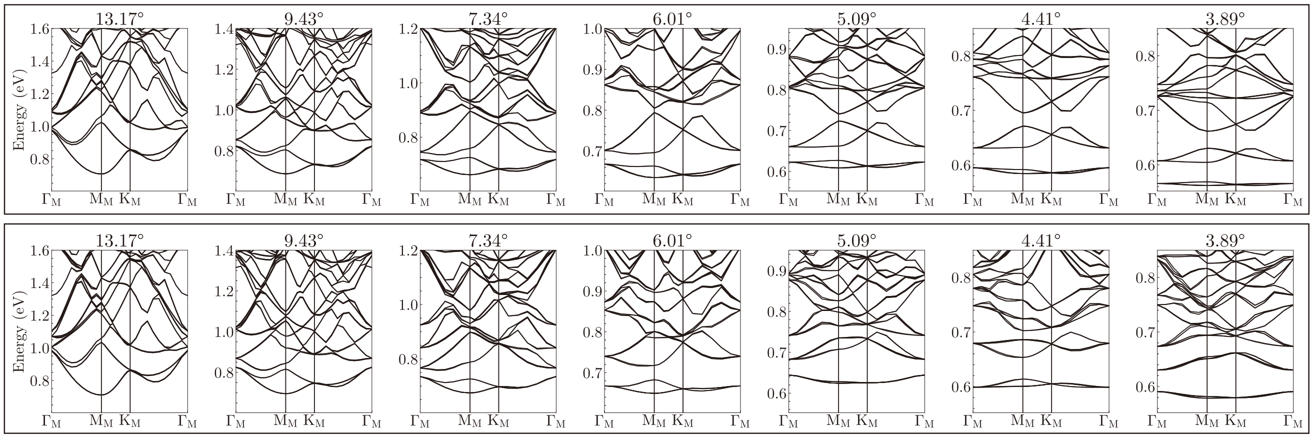

FIG. S7. The *ab initio* band structure of twisted AA-stacked (a-g) and AB-stacked (h-n) bilayer $SnSe_2$ from 13.17° to 3.89°. The lowest group of conduction bands isolated from other energy bands consists of six bands stemming from the three inequivalent M valleys of monolayer $SnSe_2$. The bandwidths of the lowest group of six bands are listed in Table S1.

valleys, protected by symmetry, will be analyzed in detail in subsequent sections. As the twist angle decreases, the moiré bands significantly flatten, with the bandwidth of the lowest set reducing to just a few meV, as detailed in Table S1. The band structure of twisted AB-stacked bilayer $SnSe_2$ shown in Figs. S7(h) to S7(n) is similar to the twisted AA-stacking case. When the twist angle decreases to 3.89°, two sets of conduction bands become isolated as shown in Figs. S7(l) to S7(n). Nonetheless, the spin splitting within the same M valley is more prominent at small angles compared to the twisted AA case (*e.g.*, the splitting in the third lowest set of bands is larger in the AB case). The bandwidth of the lowest set of valence bands from 13.17° to 3.89° are listed in Table S1.

c. ***Wilson loops.*** Finally, we compute the Wilson loops of the lowest conduction bands to explore their topology. As shown in Fig. S8, the two lowest isolated sets of conduction bands for both twisted AA and AB configurations

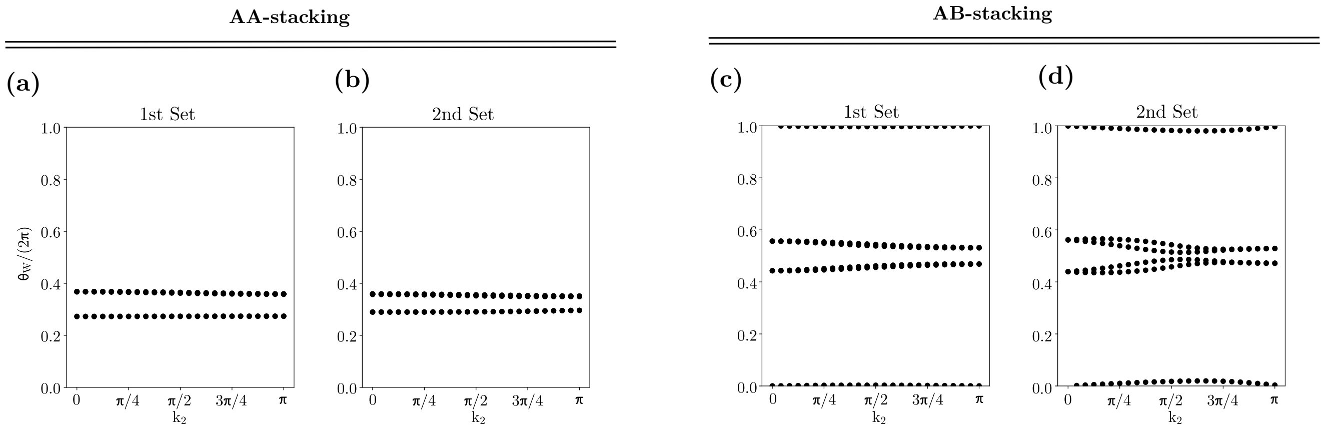

FIG. S8. The Wilson loops of twisted $SnSe_2$ at 3.89°, computed along the $\mathbf{b}_{M_1}$ direction and plotted as a function of momentum along the $\mathbf{b}_{M_2}$ direction. (a) and (b) show the results for the AA-stacked configuration, while (c) and (d) correspond to the AB-stacked configuration. The first and second lowest sets of conduction bands are displayed in (a) and (c), and (b) and (d), respectively.

exhibit no winding in their Wilson loops, indicating topologically trivial bands. However, this does not imply that the system's low-energy physics is trivial. The presence of multiple valleys and the emergence of new momentum-space non-symmorphic symmetries – discussed in detail in Sections V and VI – introduce nontrivial physical consequences not found in other systems. Note also that the Wilson loop for the AB-stacked configuration is approximately pinned at $\theta_W = 0$ and $\theta_W = \pi$. Although no *exact* symmetries enforce this behavior, it arises due to *approximate* symmetries, which will be explored in Section VI. We further discuss the implications of these approximate symmetries in Section XI.

B. Bilayer ZrS_2

This section presents the same *ab initio* results for ZrS_2 as discussed for $SnSe_2$ in Section III A. For simplicity and ease of comparison, we employ similar figure layout and only highlight the differences with $SnSe_2$.

1. *Untwisted bilayer* ZrS_2

The AA- and AB-stacked untwisted bilayer ZrS_2 have the same crystal structures as the untwisted bilayer $SnSe_2$. The corresponding band dispersions are shown in Fig. S9. The SOC opens small hybridization gaps at the band crossings but has only minor effects on the CBM at M. The layer splitting in bilayer ZrS_2 is 93 meV for the AA-stacking configuration and 222 meV for the AB-stacking one.

2. *Twisted bilayer* ZrS_2

The twisted AA- and AB-stacked bilayer ZrS_2 share similar crystal structures and the same space groups as twisted bilayer $SnSe_2$. After evaluating 21 vdW corrections, as shown in Fig. S10, we chose the DFT-D3 method by Grimme [23] for the vdW correction in ZrS_2 (*i.e.*, the third best one). This method provides lattice constants close to experimental values and is less computationally demanding than the SCAN correction. Fig. S11 illustrates the ILD and intralayer displacements of atoms in ZrS_2 after full relaxation, showing substantial similarities with $SnSe_2$, although the variations in ILD and the magnitude of intralayer displacements are about half of those in $SnSe_2$.

The *ab initio* band structures of ZrS_2 for different angles and both stacking configurations are shown in Fig. S12. The bands are qualitatively similar to the ones of $SnSe_2$, but the relevant energy scales are reduced by roughly 30% – 40%. The bandwidths of the lowest set of bands is given in Table S2 and similarly show a reduction in the energy scale compared to $SnSe_2$. Finally, in Fig. S13, we also plot the Wilson loop of the first two sets of conduction

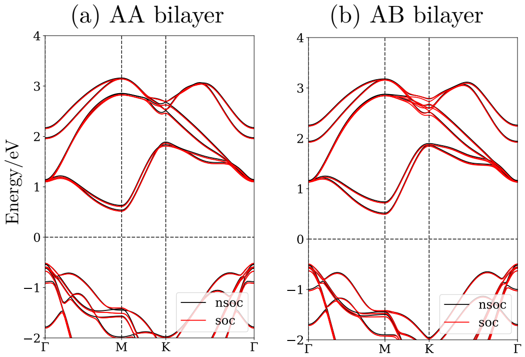

FIG. S9. Band structures of the untwisted AA- and AB-stacked bilayer ZrS_2. The DFT bands obtained with (without) SOC are shown in red (black). (a) shows the band structure for the AA-stacked bilayer, while (b) shows the same for the AB-stacked configuration.

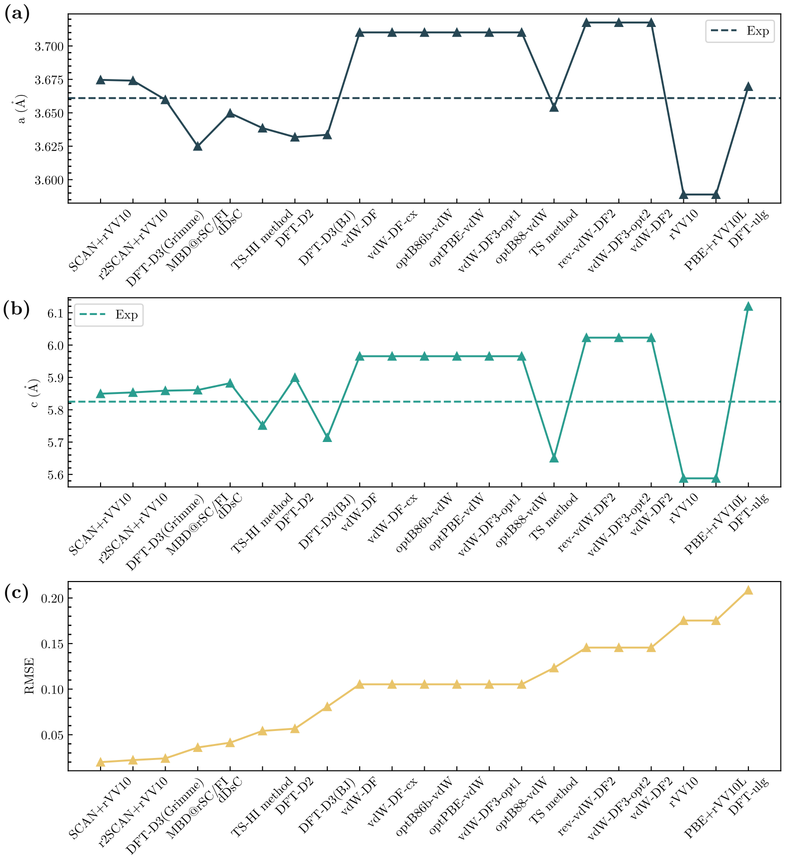

FIG. S10. Comparison of van der Waals functionals for describing the crystal structure of ZrS_2. The layout of the figure is identical to Fig. S10, but applied to ZrS_2. Experimental data is sourced from Ref. [7].

bands at 3.89°. As is the case of $SnSe_2$, there is no winding in the Wilson loop spectrum. Further results at other angles for both $SnSe_2$ and ZrS_2, as well as a discussion of the Wilson loop spectra are provided in Section XI.

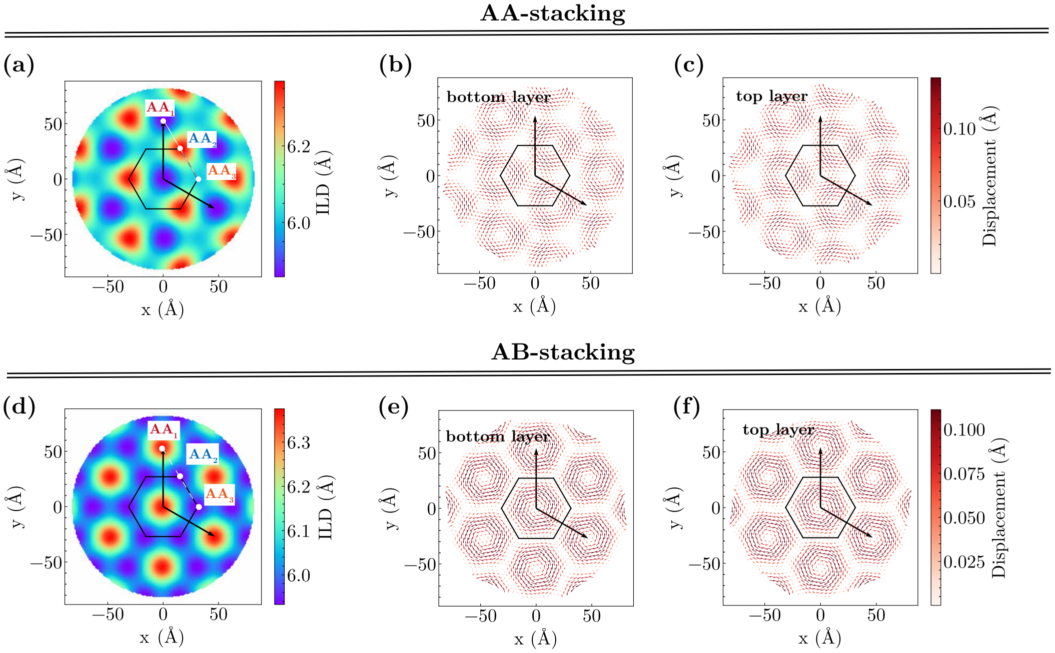

FIG. S11. Lattice relaxation results of 3.89° twisted AA and AB ZrS_2. (a), (b), and (c) are interlayer distances, the intralayer displacement of the bottom and top layers for the fully-relaxed AA stacking structure, respectively. (d), (e), and (f) are the same but for the AB stacking structure.

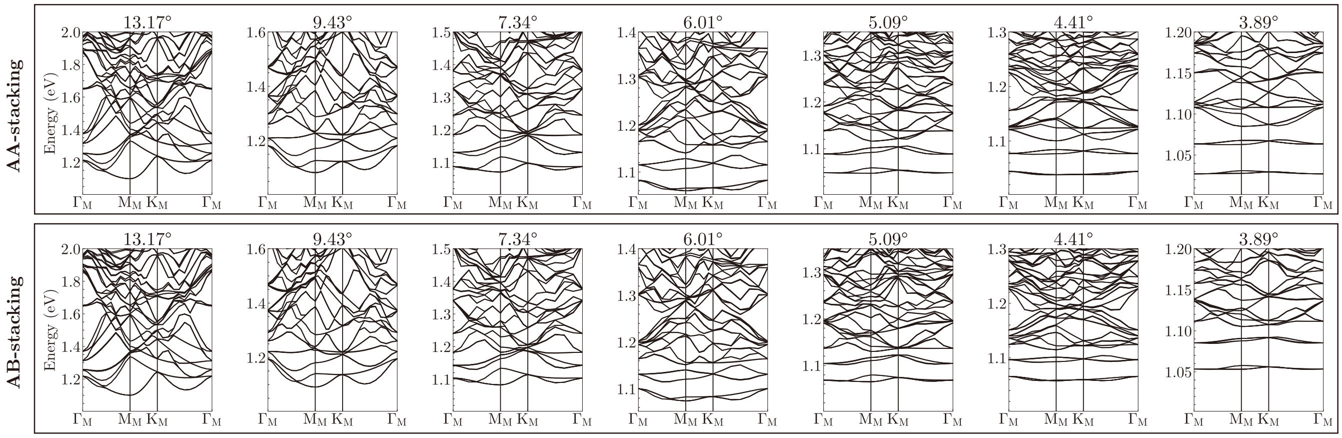

FIG. S12. The *ab initio* band structure of twisted AA-stacked (a-g) and AB-stacked (h-n) bilayer ZrS_2 from 13.17° to 3.89°. The lowest group of conduction bands isolated from other energy bands consists of six bands stemming from the three inequivalent M valleys of monolayer ZrS_2. The bandwidths of the lowest group of six bands are listed in Table S2.

IV. SINGLE-PARTICLE BISTRITZER-MACDONALD MODELS FOR TWISTED $SnSe_2$ AND ZrS_2 BILAYERS

In this section, we construct single-particle moiré heterostructure models for twisted $SnSe_2$ bilayers. We start by obtaining a Bistritzer-MacDonald (BM) [1] model using a two-center first monolayer harmonic approximation for the interlayer hopping amplitude. We constrain the form of the interlayer hopping amplitude using the symmetries of the twisted heterostructure and then further simplify the former using the symmetries of the untwisted bilayer. We then rewrite the resulting moiré Hamiltonian in the more familiar notation analogous to that used in the case of twisted bilayer graphene (TBG) by Refs. [6, 24–30] and discuss its symmetries at the general level. Importantly, this moiré Hamiltonian is defined on a kagome plane-wave lattice in momentum space.

The two-center first monolayer harmonic approximation employed in this section will be relaxed first in Section V,

Bandwidth (meV)	13.17°	9.43°	7.34°	6.01°	5.09°	4.41°	3.89°
AA	232	100	47	23	12	6	4
AB	237	105	51	26	14	8	5

TABLE S2. The bandwidths of the lowest set of conduction bands of twisted AA- and AB-stacked ZrS_2 for angles ranging from 13.17° to 3.89°.

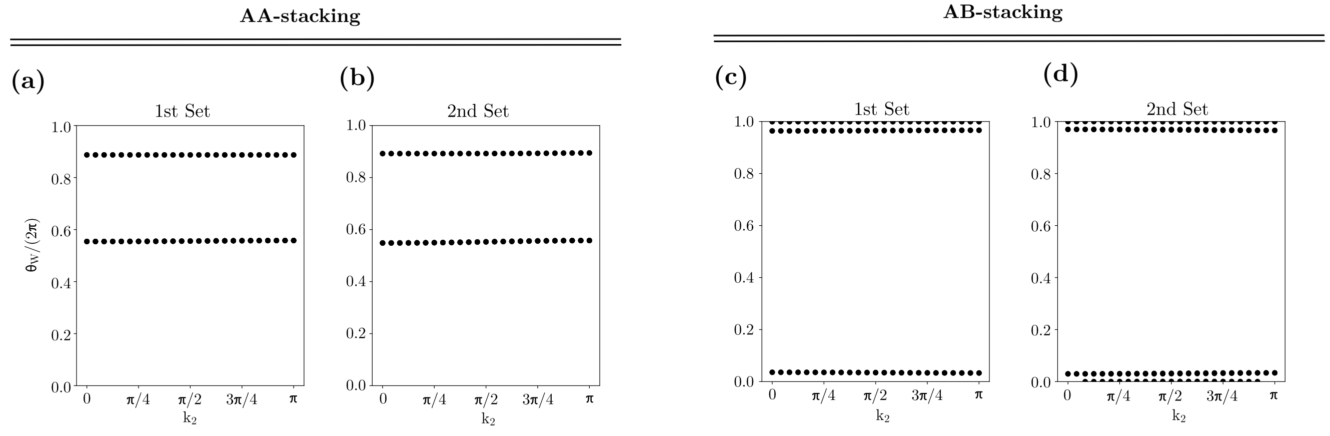

FIG. S13. The Wilson loops of twisted ZrS_2 at 3.89°. The layout of the figure is identical to Fig. S8, but applied to ZrS_2.

where we derive the moiré single-particle potential without gradient terms, and then in Section VII, where we additionally define and include gradient terms for the moiré potential. In Section VI, we also discuss the additional *approximate* symmetries of the moiré Hamiltonian arising under different physically-relevant limits, and introduce simplified models, which, as shown in Section XI, accurately describe twisted $SnSe_2$ and ZrS_2.

A. Deriving a BM model for twisted $SnSe_2$ and ZrS_2

Starting from the single-layer model of $SnSe_2$ from Section II A, we now build a BM model for the corresponding moiré heterostructures. We will consider both the AA- and AB-stacking configurations, which were introduced and discussed in Section III A 2.

1. *AA-stacking configuration*

In the context of the twisted bilayer heterostructures, we will employ the same notation as in Section II A 2 for the fermionic operators, to which we will add a layer index. As such, $\hat{a}^\dagger_{\mathbf{R},s,l}$ corresponds to the Wannier orbital of the lowest conduction band[2] and creates an electron within layer $l = \pm$ located at position $\mathcal{R}_{\theta,l}\mathbf{R}$, where the rotation matrix $\mathcal{R}_{\theta,l}$ is given by

$$\mathcal{R}_{\theta,l} = \begin{pmatrix} \cos\left(\frac{\theta l}{2}\right) & -\sin\left(\frac{\theta l}{2}\right) \\ \sin\left(\frac{\theta l}{2}\right) & \cos\left(\frac{\theta l}{2}\right) \end{pmatrix}. \tag{S4.17}$$

Correspondingly, the Fourier-transformed fermion operators for the two layers read as

$$\hat{a}^\dagger_{\mathbf{k},s,l} = \frac{1}{\sqrt{N}} \sum_{\mathbf{R}} \hat{a}^\dagger_{\mathbf{R},s,l} e^{i\mathbf{k}\cdot\mathcal{R}_{\theta,l}\mathbf{R}}. \tag{S4.18}$$

[2] In $SnSe_2$, the lowest (spinful) conduction band is gapped from the rest of the spectrum and is described by a *single* (spinful) Wannier orbital throughout the entire monolayer BZ. For ZrS_2, the lowest conduction band is part of a gapped group of three bands. However, we do not need to employ three Wannier orbitals because, at the M point of the monolayer BZ (*i.e.*, the low-energy electronic states relevant for the twisted heterostructure), there is a single (spinful) conduction band. This band is spanned (around the M point) by a Wannier orbital with symmetry properties analogous to an effective s orbital at the $1a$ Wyckoff position, similar to $SnSe_2$.

We will also find it useful to employ the Dirac bra-ket notation and define

$$|\mathbf{k}, s, l\rangle = \hat{a}^{\dagger}_{\mathbf{k},s,l} |0\rangle \quad \text{and} \quad |\mathbf{R}, s, l\rangle = \hat{a}^{\dagger}_{\mathbf{R},s,l} |0\rangle . \tag{S4.19}$$

In deriving a BM model for the twisted bilayer heterostructure, we will adapt the method of Refs. [1, 31]. We start by defining the following matrix elements

$$\left[h^{\text{bl}}_{\text{AA}} (\mathbf{k}, \mathbf{k}')\right]_{s_1 l_1; s_2 l_2} = \langle \mathbf{k}, s_1, l_1 | \mathcal{H}_{\text{AA}} | \mathbf{k}', s_2, l_2 \rangle , \tag{S4.20}$$

where $\mathcal{H}_{\text{AA}}$ is the (yet unknown) single-particle Hamiltonian operator of the system. The intralayer matrix elements can be approximated by the single-layer Hamiltonian from Eq. (S2.16) and are given by

$$\left[h^{\text{bl}}_{\text{AA}} (\mathbf{k}, \mathbf{k}')\right]_{s_1 l; s_2 l} = \delta_{\mathbf{k},\mathbf{k}'} \left[h^{\text{sl}} \left(\mathcal{R}^{-1}_{\theta,l} \mathbf{k}\right)\right]_{s_1 s_2} . \tag{S4.21}$$

This approximation ignores any intralayer moiré potential terms which could arise from relaxation effects [32], as it happens in the case of $MoTe_2$ [14, 15]. To obtain the interlayer coupling, we first transform Eq. (S4.20) in real space to afford

$$\left[h^{\text{bl}}_{\text{AA}} (\mathbf{k}, \mathbf{k}')\right]_{s_1 l; s_2 (-l)} = \frac{1}{N} \sum_{\mathbf{R},\mathbf{R}'} e^{i\mathbf{k}' \cdot \mathcal{R}_{\theta,-l} \mathbf{R}'} e^{-i\mathbf{k} \cdot \mathcal{R}_{\theta,l} \mathbf{R}} \langle \mathbf{R}, s_1, l | \mathcal{H}_{\text{AA}} | \mathbf{R}', s_2, -l \rangle . \tag{S4.22}$$

In this section, we will follow Ref. [1] and make a two-center, tight-binding approximation for the interlayer hopping amplitude, *i.e.*

$$\langle \mathbf{R}, s_1, l | \mathcal{H}_{\text{AA}} | \mathbf{R}', s_2, -l \rangle = t^{l,\text{AA}}_{s_1 s_2} \left(\mathcal{R}_{\theta,-l} \mathbf{R}' - \mathcal{R}_{\theta,l} \mathbf{R}\right) = \frac{1}{N \Omega_{\text{sl}}} \sum_{\mathbf{q},\mathbf{g}} t^{l,\text{AA}}_{s_1 s_2} (\mathbf{q} + \mathbf{g}) \, e^{-i(\mathbf{q}+\mathbf{g}) \cdot \left(\mathcal{R}_{\theta,-l} \mathbf{R}' - \mathcal{R}_{\theta,l} \mathbf{R}\right)}, \tag{S4.23}$$

where Ω_{sl} is the surface area of the single-layer material unit cell, while $t^{l,\text{AA}}_{s_1 s_2} (\mathbf{r})$ and $t^{l,\text{AA}}_{s_1 s_2} (\mathbf{q} + \mathbf{g})$ are the tunneling amplitude between two effective s Wannier orbitals located a distance $\mathbf{r}$ apart and its Fourier transformation, respectively. In Eq. (S4.23), the sum over $\mathbf{q}$ runs over the momenta of the *unrotated* BZ of the single-layer material, while $\mathbf{g}$ are the *unrotated* reciprocal lattice vectors of the single-layer material. It is worth noting, however, that due to the extended nature of the effective s-orbitals comprising the lowest two conduction bands of $SnSe_2$, the two-center approximation derived here might not furnish an accurate model for the resulting moiré Hamiltonian. The delocalized nature of the s-orbitals suggest that assisted hopping will likely take place between the layers, similarly to $MoTe_2$, where the indirect hopping processes also give rise to additional intralayer contributions beyond Eq. (S4.21). For the sake of completeness, we first will carry out a complete derivation of the moiré Hamiltonian in the two-center approximation, which will then be relaxed in Sections V and VII.

Plugging Eq. (S4.23) into Eq. (S4.22), we obtain

$$\begin{aligned}
\left[h^{\text{bl}}_{\text{AA}} (\mathbf{k}, \mathbf{k}')\right]_{s_1 l; s_2 (-l)} &= \sum_{\substack{\mathbf{q},\mathbf{g} \\ \mathbf{g}',\mathbf{g}''}} \frac{t^{l,\text{AA}}_{s_1 s_2} (\mathbf{q} + \mathbf{g})}{\Omega_{\text{sl}}} \delta_{\mathbf{q}+\mathbf{g}-\mathbf{k}', \mathcal{R}_{\theta,-l} \mathbf{g}'} \delta_{\mathbf{k}-\mathbf{q}-\mathbf{g}, \mathcal{R}_{\theta,l} \mathbf{g}''} \\
&= \sum_{\mathbf{g}',\mathbf{g}''} \frac{t^{l,\text{AA}}_{s_1 s_2} (\mathbf{k}' + \mathcal{R}_{\theta,-l} \mathbf{g}')}{\Omega_{\text{sl}}} \delta_{\mathbf{k}-\mathbf{k}', \mathcal{R}_{\theta,l} \mathbf{g}'' + \mathcal{R}_{\theta,-l} \mathbf{g}'},
\end{aligned} \tag{S4.24}$$

where the sum over $\mathbf{g}'$ and $\mathbf{g}''$ runs over the unrotated reciprocal lattice vectors of the monolayer material. The low-energy physics of the system near charge neutrality and for small twist angles $\theta \ll 1$ is dictated by the momentum states near the M points of the two layers. As a result, we can take $\mathbf{k} = C^{\eta}_{3z} \mathbf{K}^{l}_{M} + \delta\mathbf{k}$ and $\mathbf{k}' = C^{\eta'}_{3z} \mathbf{K}^{-l}_{M} + \delta\mathbf{k}'$ in Eq. (S4.24), where $0 \leq \eta, \eta' \leq 2$ index the three inequivalent valleys within each layer, $|\delta\mathbf{k}|, |\delta\mathbf{k}'| \ll |\mathbf{K}_M|$ are small momentum displacements, and we have introduced the following notation for the M points of the two layers

$$\mathbf{K}^{l}_{M} = \mathcal{R}_{\theta,l} \mathbf{K}_M . \tag{S4.25}$$

At the same time, we assume that the Fourier transformation of the interlayer tunneling amplitude decays quickly on the single-layer reciprocal lattice scale [1], such that in the sum over $\mathbf{g}'$ in Eq. (S4.24), only the leading terms need to be considered (*i.e.* the terms for which $|\mathbf{k}' + \mathcal{R}_{\theta,-l} \mathbf{g}'| = \left|\delta\mathbf{k}' + \mathcal{R}_{\theta,-l} \left(\mathbf{g}' + C^{\eta'}_{3z} \mathbf{K}_M\right)\right| \approx |\mathbf{K}_M|$). We call the resulting approximation *the two-center first monolayer harmonic approximation.* There are only two such terms, which

correspond to $\mathbf{g}' = (-1)^n C_{3z}^{\eta'} \mathbf{K}_M - C_{3z}^{\eta'} \mathbf{K}_M$ for $n = 0, 1$. At the same time, since $|\delta \mathbf{k}'| \ll |\mathbf{K}_M|$, we can assume that $t_{s_1 s_2}^{l,\mathrm{AA}} \left(\delta \mathbf{k}' + (-1)^n C_{3z}^{\eta'} \mathbf{K}_M^{-l} \right) \approx t_{s_1 s_2}^{l,\mathrm{AA}} \left((-1)^n C_{3z}^{\eta'} \mathbf{K}_M^{-l} \right) \approx t_{s_1 s_2}^{l,\mathrm{AA}} \left((-1)^n C_{3z}^{\eta'} \mathbf{K}_M \right)$. With these simplifications, Eq. (S4.24) can be rewritten as

$$
\begin{aligned}
&\left[h_{\mathrm{AA}}^{\mathrm{bl}} \left(C_{3z}^{\eta} \mathbf{K}_M^{l} + \delta \mathbf{k}, C_{3z}^{\eta'} \mathbf{K}_M^{-l} + \delta \mathbf{k}' \right) \right]_{s_1 l; s_2 (-l)} = \\
&= \sum_{n=0}^{1} \sum_{\mathbf{g}''} \frac{t_{s_1 s_2}^{l,\mathrm{AA}} \left((-1)^n C_{3z}^{\eta'} \mathbf{K}_M \right)}{\Omega_{\mathrm{sl}}} \delta_{C_{3z}^{\eta} \mathbf{K}_M^{l} + \delta \mathbf{k} - C_{3z}^{\eta'} \mathbf{K}_M^{-l} - \delta \mathbf{k}', \mathcal{R}_{\theta,l} \mathbf{g}'' + \mathcal{R}_{\theta,-l} \left[(-1)^n C_{3z}^{\eta'} \mathbf{K}_M - C_{3z}^{\eta'} \mathbf{K}_M \right]} \\
&= \sum_{n=0}^{1} \frac{t_{s_1 s_2}^{l,\mathrm{AA}} \left((-1)^n C_{3z}^{\eta} \mathbf{K}_M \right)}{\Omega_{\mathrm{sl}}} \delta_{\delta \mathbf{k} - \delta \mathbf{k}' + C_{3z}^{\eta} \left(\mathbf{K}_M^{l} - \mathbf{K}_M^{-l} \right), \left(\mathcal{R}_{\theta,l} - \mathcal{R}_{\theta,-l} \right) \left[1 - (-1)^n \right] C_{3z}^{\eta} \mathbf{K}_M} \delta_{\eta \eta'} \\
&= \sum_{n=0}^{1} \frac{t_{s_1 s_2}^{l,\mathrm{AA}} \left((-1)^n C_{3z}^{\eta} \mathbf{K}_M \right)}{\Omega_{\mathrm{sl}}} \delta_{\delta \mathbf{k} - \delta \mathbf{k}', (-1)^n \left(\mathcal{R}_{\theta,-l} - \mathcal{R}_{\theta,l} \right) C_{3z}^{\eta} \mathbf{K}_M} \delta_{\eta \eta'} .
\end{aligned}
\tag{S4.26}
$$

In going from the second to the third line of Eq. (S4.26), we have used the fact that $|\delta \mathbf{k}|, |\delta \mathbf{k}'| \ll |\mathbf{K}_M|$, which implies that the δ-function can only be non-zero if $\mathbf{g}'' = -\mathcal{R}_{\theta,-l} \left[(-1)^n C_{3z}^{\eta'} \mathbf{K}_M - C_{3z}^{\eta'} \mathbf{K}_M \right]$. Introducing the following auxiliary vectors

$$
\mathbf{q}_{\eta} = C_{3z}^{\eta} \left(\mathbf{K}_M^{-} - \mathbf{K}_M^{+} \right), \quad \text{for} \quad 0 \leq \eta \leq 2,
\tag{S4.27}
$$

as well as the rescaled hopping amplitude Fourier transformation

$$
\tilde{t}_{s_1 s_2}^{l,\mathrm{AA}} \left(\mathbf{q} + \mathbf{g} \right) = \frac{t_{s_1 s_2}^{l,\mathrm{AA}} \left(\mathbf{q} + \mathbf{g} \right)}{\Omega_{\mathrm{sl}}},
\tag{S4.28}
$$

we can rewrite the interlayer matrix elements as

$$
\left[h_{\mathrm{AA}}^{\mathrm{bl}} \left(C_{3z}^{\eta} \mathbf{K}_M^{l} + \delta \mathbf{k}, C_{3z}^{\eta'} \mathbf{K}_M^{-l} + \delta \mathbf{k}' \right) \right]_{s_1 l; s_2 (-l)} = \sum_{n=0}^{1} \tilde{t}_{s_1 s_2}^{l,\mathrm{AA}} \left((-1)^n C_{3z}^{\eta} \mathbf{K}_M \right) \delta_{\delta \mathbf{k} - \delta \mathbf{k}', l (-1)^n \mathbf{q}_{\eta}} \delta_{\eta \eta'},
\tag{S4.29}
$$

where the reader is reminded that l and s_1, s_2 refer to the layer and spin indices, respectively. In contrast to TBG, the absence of additional phase factors in Eq. (S4.29) arises from the fact that the effective s-orbitals in the monolayer material are centered at the origin of the unit cell. Furthermore, unlike TBG, within a single valley, momentum states are coupled only along a single direction (determined by $\mathbf{q}_{\eta}$ in valley η). As will be discussed in Section VI D, this leads to the first monolayer harmonic model having discrete moiré translation symmetry in only one direction and continuous translation symmetry in the other.

2. AB-stacking configuration

The derivation of the single-particle Hamiltonian for the AB-stacking configuration proceeds analogously to the one shown for the AA-stacking case. This time, however, $\hat{a}_{\mathbf{R},s,l}^{\dagger}$ will create an electron within layer l located at position $l \mathcal{R}_{\theta,l} \mathbf{R}$ (note the additional layer index specifying that the bottom layer is additionally rotated by $180°$ around the $\hat{\mathbf{z}}$ direction relative to the AA-stacking configuration). The Fourier-transformed operators for the two layers $l = \pm$ are now defined by

$$
\hat{a}_{\mathbf{k},s,l}^{\dagger} = \frac{1}{\sqrt{N}} \sum_{\mathbf{R}} \hat{a}_{\mathbf{R},s,l}^{\dagger} e^{i l \mathbf{k} \cdot \mathcal{R}_{\theta,l} \mathbf{R}}.
\tag{S4.30}
$$

In this modified momentum basis, the matrix elements of the AB-stacked configurations are defined similarly to Eq. (S4.20)

$$
\left[h_{\mathrm{AB}}^{\mathrm{bl}} \left(\mathbf{k}, \mathbf{k}' \right) \right]_{s_1 l_1; s_2 l_2} = \left\langle \mathbf{k}, s_1, l_1 \right| \mathcal{H}_{\mathrm{AB}} \left| \mathbf{k}', s_2, l_2 \right\rangle,
\tag{S4.31}
$$

but with the intralayer matrix elements being given by

$$
\left[h_{\mathrm{AB}}^{\mathrm{bl}} \left(\mathbf{k}, \mathbf{k}' \right) \right]_{s_1 l; s_2 l} = \delta_{\mathbf{k}, \mathbf{k}'} \left[h^{\mathrm{sl}} \left(l \mathcal{R}_{\theta,l}^{-1} \mathbf{k} \right) \right]_{s_1 s_2}.
\tag{S4.32}
$$

Note that because the monolayer Hamiltonian $h^{\text{sl}}(\mathbf{k})$ is even in $\mathbf{k}$, the introduction of the additional l factor compared with Eq. (S4.21) does not change the intralayer part of the moiré Hamiltonian between the AA- and AB-stacking configurations at the level of the two-center first monolayer harmonic approximation.

At the same time, the interlayer part of the Hamiltonian is given by

$$\left[h^{\text{bl}}_{\text{AB}}(\mathbf{k},\mathbf{k}')\right]_{s_1 l; s_2(-l)} = \frac{1}{N}\sum_{\mathbf{R},\mathbf{R}'} e^{-il\mathbf{k}'\cdot\mathcal{R}_{\theta,-l}\mathbf{R}'} e^{-il\mathbf{k}\cdot\mathcal{R}_{\theta,l}\mathbf{R}} \left\langle \mathbf{R}, s_1, l \middle| \mathcal{H}_{\text{AB}} \middle| \mathbf{R}', s_2, -l \right\rangle, \tag{S4.33}$$

for which the same two-center approximation as in Eq. (S4.23) gives

$$\left\langle \mathbf{R}, s_1, l \middle| \mathcal{H}_{\text{AB}} \middle| \mathbf{R}', s_2, -l \right\rangle = t^{l,\text{AB}}_{s_1 s_2}\left(-l\mathcal{R}_{\theta,-l}\mathbf{R}' - l\mathcal{R}_{\theta,l}\mathbf{R}\right) = \frac{1}{N\Omega_{\text{sl}}}\sum_{\mathbf{q},\mathbf{g}} t^{l,\text{AB}}_{s_1 s_2}(\mathbf{q}+\mathbf{g})\, e^{i(\mathbf{q}+\mathbf{g})\cdot\left(l\mathcal{R}_{\theta,-l}\mathbf{R}' + l\mathcal{R}_{\theta,l}\mathbf{R}\right)}. \tag{S4.34}$$

From Eqs. (S4.33) and (S4.34), we find that

$$\begin{aligned}
\left[h^{\text{bl}}_{\text{AB}}(\mathbf{k},\mathbf{k}')\right]_{s_1 l; s_2(-l)} &= \sum_{\substack{\mathbf{q},\mathbf{g}\\ \mathbf{g}',\mathbf{g}''}} \frac{t^{l,\text{AB}}_{s_1 s_2}(\mathbf{q}+\mathbf{g})}{\Omega_{\text{sl}}} \delta_{\mathbf{q}+\mathbf{g}-\mathbf{k}',-l\mathcal{R}_{\theta,-l}\mathbf{g}'} \delta_{\mathbf{k}-\mathbf{q}-\mathbf{g},l\mathcal{R}_{\theta,l}\mathbf{g}''} \\
&= \sum_{\mathbf{g}',\mathbf{g}''} \frac{t^{l,\text{AB}}_{s_1 s_2}(\mathbf{k}' - l\mathcal{R}_{\theta,-l}\mathbf{g}')}{\Omega_{\text{sl}}} \delta_{\mathbf{k}-\mathbf{k}',l\mathcal{R}_{\theta,l}\mathbf{g}''-l\mathcal{R}_{\theta,-l}\mathbf{g}'}.
\end{aligned} \tag{S4.35}$$

As in Section IV A 1, we take $\mathbf{k} = C^{\eta}_{3z}\mathbf{K}^{l}_{M} + \delta\mathbf{k}$ and $\mathbf{k}' = C^{\eta'}_{3z}\mathbf{K}^{-l}_{M} + \delta\mathbf{k}'$ in Eq. (S4.35), with $0 \leq \eta, \eta' \leq 2$ and assume that the Fourier transformation of the interlayer tunneling amplitude decays quickly on the single-layer reciprocal lattice scale [1]. As such, in the sum over $\mathbf{g}'$ from Eq. (S4.35), we can restrict ourselves to the leading terms for which $\left|\mathbf{k}' - l\mathcal{R}_{\theta,-l}\mathbf{g}'\right| = \left|\delta\mathbf{k}' + \mathcal{R}_{\theta,-l}\left(-l\mathbf{g}' + C^{\eta'}_{3z}\mathbf{K}_M\right)\right| \approx \left|\mathbf{K}_M\right|$. There are only two such terms, which correspond to $-l\mathbf{g}' = (-1)^n C^{\eta'}_{3z}\mathbf{K}_M - C^{\eta'}_{3z}\mathbf{K}_M$ for $n = 0, 1$. At the same time, since $|\delta\mathbf{k}'| \ll |\mathbf{K}_M|$, we can assume that $t^{l,\text{AB}}_{s_1 s_2}\left(\delta\mathbf{k}' + (-1)^n C^{\eta'}_{3z}\mathbf{K}^{-l}_M\right) \approx t^{l,\text{AB}}_{s_1 s_2}\left((-1)^n C^{\eta'}_{3z}\mathbf{K}^{-l}_M\right) \approx t^{l,\text{AB}}_{s_1 s_2}\left((-1)^n C^{\eta'}_{3z}\mathbf{K}_M\right)$. With these simplifications, Eq. (S4.35) can be rewritten as

$$\begin{aligned}
&\left[h^{\text{bl}}_{\text{AB}}\left(C^{\eta}_{3z}\mathbf{K}^{l}_{M} + \delta\mathbf{k}, C^{\eta'}_{3z}\mathbf{K}^{-l}_{M} + \delta\mathbf{k}'\right)\right]_{s_1 l; s_2(-l)} = \\
&= \sum_{n=0}^{1}\sum_{\mathbf{g}''} \frac{t^{l,\text{AB}}_{s_1 s_2}\left((-1)^n C^{\eta'}_{3z}\mathbf{K}_M\right)}{\Omega_{\text{sl}}} \delta_{C^{\eta}_{3z}\mathbf{K}^{l}_{M}+\delta\mathbf{k}-C^{\eta'}_{3z}\mathbf{K}^{-l}_{M}-\delta\mathbf{k}',l\mathcal{R}_{\theta,l}\mathbf{g}''+\mathcal{R}_{\theta,-l}\left[(-1)^n C^{\eta'}_{3z}\mathbf{K}_M - C^{\eta'}_{3z}\mathbf{K}_M\right]} \\
&= \sum_{n=0}^{1}\sum_{\mathbf{g}''} \frac{t^{l,\text{AB}}_{s_1 s_2}\left((-1)^n C^{\eta'}_{3z}\mathbf{K}_M\right)}{\Omega_{\text{sl}}} \delta_{C^{\eta}_{3z}\mathbf{K}^{l}_{M}+\delta\mathbf{k}-C^{\eta'}_{3z}\mathbf{K}^{-l}_{M}-\delta\mathbf{k}',\mathcal{R}_{\theta,l}\mathbf{g}''+\mathcal{R}_{\theta,-l}\left[(-1)^n C^{\eta'}_{3z}\mathbf{K}_M - C^{\eta'}_{3z}\mathbf{K}_M\right]},
\end{aligned} \tag{S4.36}$$

where, in the last line, we have changed the summation variable $\mathbf{g}'' \to l\mathbf{g}''$. Eq. (S4.36) is similar to the second line of Eq. (S4.26), so the derivation proceeds analogously, allowing us to conclude that

$$\left[h^{\text{bl}}_{\text{AB}}\left(C^{\eta}_{3z}\mathbf{K}^{l}_{M} + \delta\mathbf{k}, C^{\eta'}_{3z}\mathbf{K}^{-l}_{M} + \delta\mathbf{k}'\right)\right]_{s_1 l; s_2(-l)} = \sum_{n=0}^{1} \tilde{t}^{l,\text{AB}}_{s_1 s_2}\left((-1)^n C^{\eta}_{3z}\mathbf{K}_M\right) \delta_{\delta\mathbf{k}-\delta\mathbf{k}',l(-1)^n\mathbf{q}_\eta} \delta_{\eta\eta'}, \tag{S4.37}$$

where we have defined the rescaled hopping amplitude Fourier transformation

$$\tilde{t}^{l,\text{AB}}_{s_1 s_2}(\mathbf{q}+\mathbf{g}) = \frac{t^{l,\text{AB}}_{s_1 s_2}(\mathbf{q}+\mathbf{g})}{\Omega_{\text{sl}}}. \tag{S4.38}$$

Note that Eqs. (S4.29) and (S4.37) are identical in form. The difference between the interlayer Hamiltonians of the two stacking arrangements arise due to the different symmetries of the two structures, which were discussed in Section III A 2. The constraints imposed by these symmetries on the interlayer hopping amplitudes will be the focus of the following Section IV B.

B. Constraining the interlayer hopping amplitude with symmetries

Naively, the two-center interlayer hopping amplitude for both the AA- and AB-stacking configurations $\tilde{t}^{l}_{s_1 s_2}\left(\pm C^{\eta}_{3z}\mathbf{K}_M\right)$ is characterized by $2\,(\text{spin } s_1) \times 2\,(\text{spin } s_2) \times 6\,(\text{distinct momenta } \pm C^{\eta}_{3z}\mathbf{K}_M) \times 2\,(\text{layers } l) = 48$ complex parameters (or 96 real parameters), where no Hermiticity condition was imposed. However, not all of these parameters are independent. Our goal in this section is to employ the exact symmetries of the twisted bilayer system, as well as the properties of $\tilde{t}^{l}_{s_1 s_2}\left(\mathbf{q}+\mathbf{g}\right)$ to obtain the *independent* components specifying the interlayer hopping amplitude. We will also show how the symmetries of the *untwisted* bilayer arrangements discussed in Section III A 1 further provide *approximate* constraints on the interlayer hopping amplitude [33].

We start by noting that due to the Hermiticity of the bilayer Hamiltonian, one must have that $\langle \mathbf{R}, s_1, l|\mathcal{H}|\mathbf{R}', s_2, -l\rangle = \langle \mathbf{R}', s_2, -l|\mathcal{H}|\mathbf{R}, s_1, l\rangle^*$, which implies that

$$\tilde{t}^{l}_{s_1 s_2}\left(\mathbf{q}+\mathbf{g}\right) = \tilde{t}^{-l*}_{s_2 s_1}\left(\mathbf{q}+\mathbf{g}\right). \tag{S4.39}$$

Strictly speaking, for arbitrary angles and interlayer in-plane displacements (which result in an incommensurate arrangement of the two layers), our heterostructures, similar to TBG [34], do not feature any *exact* symmetries, other than time-reversal symmetry $\mathcal{T}$[3]. Nevertheless, for TBG at incommensurate angle, over length scales comparable with the moiré unit cell, but much larger than the single-layer unit cell, the C_{6z} and C_{2x} symmetries of the single-layer give rise to *emergent* (but otherwise approximate) C_{6z} and C_{2x} symmetries of the twisted bilayer arrangement. Moreover, while not even an emergent symmetry of TBG at nonzero twist angles θ, the m_y symmetry of *monolayer* graphene (corresponding to reflections across a plane perpendicular to the graphene plane) constrains the form of interlayer hopping amplitude, which is therefore only specified by two real parameters within the BM model [33].

The emergence of various symmetries in the twisted bilayer arrangement at incommensurate angles can be understood as follows. In the two-center approximation for the interlayer hopping, the $t^{l}_{s_1 s_2}\left(\mathbf{r}\right)$ function introduced in Eqs. (S4.23) and (S4.34) is independent on the twist angle θ. At the same time, within the first-harmonic approximation, the interlayer matrix elements from Eqs. (S4.29) and (S4.37) only depend on the twist angle via the auxiliary vectors $\mathbf{q}_i$ from Eq. (S4.27). As a result, one can choose a commensurate geometry for the bilayer arrangement, and use the *exact* symmetries of the system to constrain the form of the $\tilde{t}^{l}_{s_1 s_2}\left(\pm C^{\eta}_{3z}\mathbf{K}_M\right)$ tensor and determine its independent components. Finally, the parameterization of $\tilde{t}^{l}_{s_1 s_2}\left(\pm C^{\eta}_{3z}\mathbf{K}_M\right)$ determined in the symmetric commensurate arrangement *will remain* valid (within the first-harmonic approximation) for general layer displacements and small (but non-zero) twist angles. As a result, the exact symmetries of the moiré heterostructure at commensurate twist angles give rise to emerging symmetries of the system at generic incommensurate angles.

An alternative approach, which we will employ below, is to consider a generic (incommensurate) twist angle θ *without* any interlayer shift. This is consistent with the convention introduced around Eq. (S4.17), where the twisted heterostructure is generated by rotating the two layers relative to each other around the common origins of the two monolayer lattices. This configuration retains point group symmetries about the origin, which can be used to constrain the interlayer hopping amplitude.

We will now determine the properties of the interlayer hopping amplitude $\tilde{t}^{l}_{s_1 s_2}\left(\pm C^{\eta}_{3z}\mathbf{K}_M\right)$ arising from the symmetries of the single-layer material. We will consider both the symmetries of the system arising for a small, but *non-zero* twist angle, as well as the enhanced symmetries of the bilayer system *at zero twist angle.* As was discussed in Section III A, depending on the stacking arrangement, both the untwisted and twisted heterostructures will feature different symmetries, in addition to time reversal $\mathcal{T}$ symmetry:

1. For AA-stacking and $\theta \neq 0$, the system features C_{3z} and C_{2x} symmetries which give rise to the space group $P3121'$ (SSG 149.22). In the $\theta = 0$ case, the system will additionally feature $\mathcal{I}$ symmetry, such that its symmetry group is given by $P\bar{3}m11'$ (SSG 164.86).

2. For AB-stacking, the system will feature C_{3z} and C_{2y} symmetries in the $\theta \neq 0$ case, which generate the $P3211'$ group (SSG 150.26). When $\theta = 0$, the system features $P\bar{6}m21'$ symmetry (SSG 187.210), which is generated by the C_{3z}, C_{2y}, and M_z symmetries.

In what follows, we will first employ the symmetries of the $\theta \neq 0$ heterostructure to constrain the interlayer hopping amplitude. We will then use the additional symmetries arising in the $\theta = 0$ case to further constrain the interlayer tunneling. We expect that these latter constraints will hold approximately in the limit of small but nonvanishing twist angles.

---

[3] For example, imposing the Hermicity condition from Eq. (S4.39) and the time-reversal symmetry according to Eq. (S4.44) will reduce the number of real independent parameters of $\tilde{t}^{l}_{s_1 s_2}\left(\pm C^{\eta}_{3z}\mathbf{K}_M\right)$ from 96 to 24.

To this end, we let g be a symmetry of the bilayer system. As a result, $[g,\mathcal{H}]=0$ (where $\mathcal{H}$ is the Hamiltonian of the heterostructure), which implies that

$$\begin{aligned}\langle \mathbf{R}, s_1, l|\mathcal{H}|\mathbf{R}', s_2, -l\rangle &= \left\langle \mathbf{R}, s_1, l\middle|g^{-1}g\mathcal{H}\middle|\mathbf{R}', s_2, -l\right\rangle = \left\langle \mathbf{R}, s_1, l\middle|g^{-1}\mathcal{H}g\middle|\mathbf{R}', s_2, -l\right\rangle = \\ &= \sum_{s_1',s_2'} \left[D^{\mathrm{sl}}\left(g\right)\right]^*_{s_1's_1} \langle g\mathbf{R}, s_1', \epsilon_g l|\mathcal{H}|g\mathbf{R}', s_2', -\epsilon_g l\rangle^{(*)} \left[D^{\mathrm{sl}}\left(g\right)\right]_{s_2's_2}. \end{aligned} \tag{S4.40}$$

In Eq. (S4.40), $^{(*)}$ denotes a complex conjugation of the matrix elements whenever g is antiunitary, while $\epsilon_g=+1$ ($\epsilon_g=-1$) if the symmetry g preserves (exchanges) the two layers. Using the notation from Eqs. (S4.23) and (S4.34), Eq. (S4.40) imposes the following constraint on the two-center hopping amplitude function

$$t^l_{s_1s_2}\left(\mathbf{r}\right) = \sum_{s_1',s_2'} \left[D^{\mathrm{sl}}\left(g\right)\right]^*_{s_1's_1} t^{\epsilon_g l(*)}_{s_1's_2'}\left(g\mathbf{r}\right) \left[D^{\mathrm{sl}}\left(g\right)\right]_{s_2's_2}, \tag{S4.41}$$

where $\mathbf{r}=\mathcal{R}_{\theta,-l}\mathbf{R}'-\mathcal{R}_{\theta,l}\mathbf{R}$ in the AA-stacked case or $\mathbf{r}=-l\mathcal{R}_{\theta,-l}\mathbf{R}'-l\mathcal{R}_{\theta,l}\mathbf{R}$ in the AB-stacked case. This follows directly from the definitions in Eqs. (S4.23) and (S4.34). For general incommensurate twist angles, as $\mathbf{R}$ and $\mathbf{R}'$ take different discrete values in the monolayer lattice, $\mathbf{r}$ forms a dense subset of the entire two-dimensional space. As such, because $t^l_{s_1s_2}\left(\mathbf{r}\right)$ is a smooth function of $\mathbf{r}$, Eq. (S4.41) will hold for *any* two-dimensional vector $\mathbf{r}$. In momentum space, Eq. (S4.41) can therefore be written as

$$t^l_{s_1s_2}\left(\mathbf{q}+\mathbf{g}\right) = \sum_{s_1',s_2'} \left[D^{\mathrm{sl}}\left(g\right)\right]^*_{s_1's_1} t^{\epsilon_g l(*)}_{s_1's_2'}\left(g\left(\mathbf{q}+\mathbf{g}\right)\right) \left[D^{\mathrm{sl}}\left(g\right)\right]_{s_2's_2}. \tag{S4.42}$$

Section V B provides a proof that does not rely on any assumptions about the twist angles and derives the moiré potential beyond the two-center first monolayer harmonic approximation.

1. *Constraints arising from the $\theta \neq 0$ symmetries*

Explicitly, the C_{3z} and $\mathcal{T}$ symmetries, respectively, impose the following constraints on the hopping amplitude tensor for both the AA- and AB-stacking configurations

$$\tilde{t}^l_{s_1s_2}\left(\pm C^\eta_{3z}\mathbf{K}_M\right) = \sum_{s_1',s_2'} \left[D^{\mathrm{sl}}\left(C_{3z}\right)\right]^*_{s_1's_1} \tilde{t}^l_{s_1's_2'}\left(\pm C^{\eta+1}_{3z}\mathbf{K}_M\right) \left[D^{\mathrm{sl}}\left(C_{3z}\right)\right]_{s_2's_2}, \quad \text{with} \quad D^{\mathrm{sl}}\left(C_{3z}\right) = e^{-\frac{\pi i}{3}s_z}, \tag{S4.43}$$

$$\tilde{t}^l_{s_1s_2}\left(\pm C^\eta_{3z}\mathbf{K}_M\right) = \sum_{s_1',s_2'} \left[D^{\mathrm{sl}}\left(\mathcal{T}\right)\right]^*_{s_1's_1} \tilde{t}^{l*}_{s_1's_2'}\left(\mp C^{\eta}_{3z}\mathbf{K}_M\right) \left[D^{\mathrm{sl}}\left(\mathcal{T}\right)\right]_{s_2's_2}, \quad \text{with} \quad D^{\mathrm{sl}}\left(\mathcal{T}\right) = is_y. \tag{S4.44}$$

Depending on the stacking arrangement, we additionally have

$$\tilde{t}^{l,\mathrm{AA}}_{s_1s_2}\left(\pm C^\eta_{3z}\mathbf{K}_M\right) = \sum_{s_1',s_2'} \left[D^{\mathrm{sl}}\left(C_{2x}\right)\right]^*_{s_1's_1} \tilde{t}^{-l,\mathrm{AA}}_{s_1's_2'}\left(\pm C_{2x}C^{\eta}_{3z}\mathbf{K}_M\right) \left[D^{\mathrm{sl}}\left(C_{2x}\right)\right]_{s_2's_2}, \quad \text{with} \quad D^{\mathrm{sl}}\left(C_{2x}\right) = -is_x, \tag{S4.45}$$

$$\tilde{t}^{l,\mathrm{AB}}_{s_1s_2}\left(\pm C^\eta_{3z}\mathbf{K}_M\right) = \sum_{s_1',s_2'} \left[D^{\mathrm{sl}}\left(C_{2y}\right)\right]^*_{s_1's_1} \tilde{t}^{-l,\mathrm{AB}}_{s_1's_2'}\left(\pm C_{2y}C^{\eta}_{3z}\mathbf{K}_M\right) \left[D^{\mathrm{sl}}\left(C_{2y}\right)\right]_{s_2's_2}, \quad \text{with} \quad D^{\mathrm{sl}}\left(C_{2y}\right) = -is_y. \tag{S4.46}$$

Eqs. (S4.43) to (S4.46) provide exact symmetry constraints on the interlayer hopping amplitude. Apart for the C_{2y} symmetry, whose representation matrix is only given in Eq. (S4.46), the representation matrices of all the other symmetries appearing in Eqs. (S4.43) to (S4.46) are given in Eq. (S2.6) and repeated here for convenience.

2. *Constraints arising from the $\theta = 0$ symmetries*

The zero-twist heterostructure features additional symmetries, as discussed above Eq. (S4.40). These symmetries further constrain the interlayer hopping amplitude for $\theta=0$. Because $\tilde{t}^l_{s_1s_2}\left(\mathbf{q}+\mathbf{g}\right)$ is taken to be approximately angle-independent, we expect that these constraints will continue to hold approximately for small, but non-zero twist angles $\theta\neq 0$. To determine the constraints imposed by the $\theta=0$ symmetries, we can still use Eq. (S4.40) in the limit of a small vanishing incommensurate angle, for which we find

$$\tilde{t}^{l,\mathrm{AA}}_{s_1s_2}\left(\pm C^\eta_{3z}\mathbf{K}_M\right) = \sum_{s_1',s_2'} \left[D^{\mathrm{sl}}\left(\mathcal{I}\right)\right]^*_{s_1's_1} \tilde{t}^{-l,\mathrm{AA}}_{s_1's_2'}\left(\mp C^{\eta}_{3z}\mathbf{K}_M\right) \left[D^{\mathrm{sl}}\left(\mathcal{I}\right)\right]_{s_2's_2}, \quad \text{where} \quad D^{\mathrm{sl}}\left(\mathcal{I}\right) = s_0, \tag{S4.47}$$

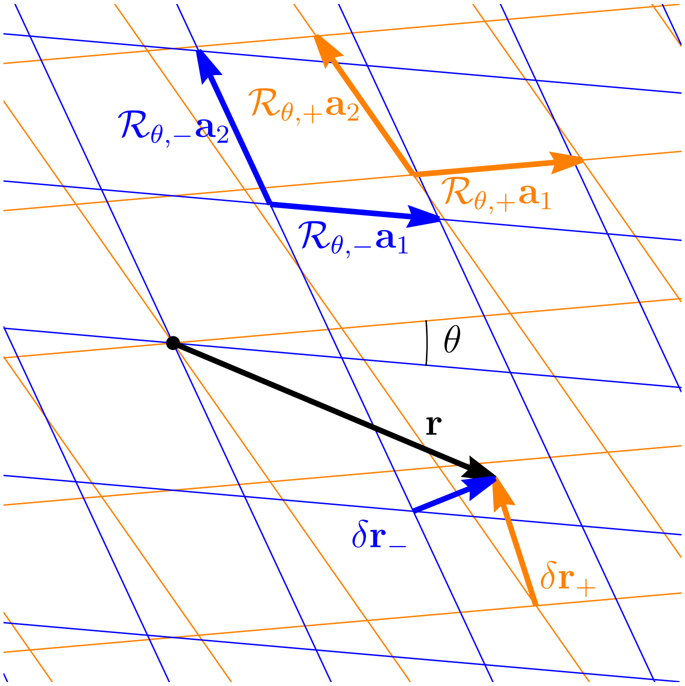

FIG. S14. Deriving the moiré translation vectors for general twist angles θ. The monolayer hexagonal lattice spanned by $\mathcal{R}_{\theta,+}\mathbf{a}_{1,2}$ ($\mathcal{R}_{\theta,-}\mathbf{a}_{1,2}$) corresponding to the top (bottom) layer is shown by the orange (blue) grid, where the rotation matrix $\mathcal{R}_{\theta,l}$ is defined in Eq. (S4.17). The two lattices are rotated relative to one another by an angle θ around the common origin, which is marked by the thick black dot. A general position $\mathbf{r}$ is displaced by $\delta\mathbf{r}_+$ ($\delta\mathbf{r}_-$) from a nearby unit cell origin of the top (bottom) layer, as defined in Eq. (S4.52). The relative displacement between the two layers at position $\mathbf{r}$ is defined and obtained around Eq. (S4.54).

$$\tilde{t}^{l,\mathrm{AB}}_{s_1 s_2}\left(\pm C^{\eta}_{3z}\mathbf{K}_M\right) = \sum_{s'_1,s'_2}\left[D^{\mathrm{sl}}\left(M_z\right)\right]^*_{s'_1 s_1}\tilde{t}^{-l,\mathrm{AB}}_{s'_1 s'_2}\left(\pm C^{\eta}_{3z}\mathbf{K}_M\right)\left[D^{\mathrm{sl}}\left(M_z\right)\right]_{s'_2 s_2}, \quad \text{where} \quad D^{\mathrm{sl}}\left(M_z\right) = -is_z. \tag{S4.48}$$

A more rigorous and complete derivation is presented in Section V B, which also extends beyond the two-center approximation.

With the exact symmetry constraints in Section IV B 1, the interlayer hopping matrices for the AA- and AB-stacking arrangements are given by

$$\tilde{t}^{l,\mathrm{AA}}\left(\pm\mathbf{K}_M\right) = \left(\pm ilw^{\mathrm{AA}}_1 + w^{\mathrm{AA}}_2\right)s_0 \pm \left(w^{\mathrm{AA}}_4 + w^{\mathrm{AA}}_6 e^{\mp il\frac{\pi}{6}}\right)s_y + \left(ilw^{\mathrm{AA}}_3 \pm w^{\mathrm{AA}}_5\right)s_z, \tag{S4.49}$$

$$\tilde{t}^{l,\mathrm{AB}}\left(\pm\mathbf{K}_M\right) = w^{\mathrm{AB}}_2 s_0 + ilw^{\mathrm{AB}}_1 s_x \pm w^{\mathrm{AB}}_3 s_y + ilw^{\mathrm{AB}}_4 s_z, \tag{S4.50}$$

where w^{AA}_i for $1 \leq i \leq 6$ and w^{AB}_i for $1 \leq i \leq 4$ are real parameters characterizing the interlayer hopping amplitudes within the two-center single-harmonic approximation. The interlayer tunneling matrices at other momenta can be directly determined from Eq. (S4.43). Finally, we note that the symmetries of the $\theta = 0$ configuration will further impose that

$$w^{\mathrm{AA}}_i = 0, \quad \text{for} \quad 3 \leq i \leq 6, \quad \text{and} \quad w^{\mathrm{AB}}_i = 0, \quad \text{for} \quad 3 \leq i \leq 4. \tag{S4.51}$$

In what follows, we will call the model corresponding to Eq. (S4.51) the *simplified* two-center first monolayer harmonic model, while the model of Eqs. (S4.49) and (S4.50) will be called the *full* two-center first monolayer harmonic model. It is worth noting that in the AA-stacking case, the $\mathcal{IT}$ symmetry of the untwisted bilayer implies SU (2) symmetry within the simplified two-center first monolayer harmonic model, since the interlayer hopping matrix becomes spin-diagonal.

C. The twisted bilayer model and its exact symmetries

From a geometric standpoint, the twisted bilayer system exhibits an effective moiré translation symmetry characterized by the moiré unit cell lattice vectors $\mathbf{a}_{M_1}$ and $\mathbf{a}_{M_2}$, which will be determined below. To derive these vectors

for a general (not necessarily commensurate) twist angle θ, we begin by examining Fig. S14, which shows the two twisted monolayer lattices superimposed.

Our goal is to determine the relative displacement $\delta\mathbf{R}(\mathbf{r})$ between the two monolayer lattices at a given position $\mathbf{r}$. In layer $l = \pm$, the position $\mathbf{r}$ is displaced from a nearby unit cell's origin of the corresponding monolayer unit cell by

$$\begin{aligned}\delta\mathbf{r}_l &\equiv \sum_{i=1}^{2} \frac{1}{2\pi} \left[(\mathcal{R}_{\theta,l}\mathbf{b}_i) \cdot \mathbf{r} \mod 2\pi \right] (\mathcal{R}_{\theta,l}\mathbf{a}_i) \\ &= \sum_{i=1}^{2} \frac{1}{2\pi} \left[\mathbf{b}_i \cdot \mathcal{R}_{\theta,l}^{-1}\mathbf{r} \mod 2\pi \right] (\mathcal{R}_{\theta,l}\mathbf{a}_i), \end{aligned} \tag{S4.52}$$

as shown in Fig. S14. We note that $\delta\mathbf{r}_l$ is only defined up to a rotated monolayer lattice vector $\mathcal{R}_{\theta,l}\mathbf{a}_i$ (for $i = 1,2$). For concreteness, we take $\delta\mathbf{r}_l$ to obey $0 \leq \delta\mathbf{r}_l \cdot (\mathcal{R}_{\theta,l}\mathbf{a}_i) < 1$ (for $i = 1,2$), but the exact convention is unimportant in what follows.

We then imagine rotating back the two monolayer lattices (*i.e.*, layer l is rotated by $-\frac{l\theta}{2}$) around the point $\mathbf{r}$. The two monolayer lattices will become aligned (*i.e.*, their lattice vectors will become parallel), but their origins will be displaced from one another by the *relative displacement.* We define this relative displacement as the local displacement of the top layer relative to the bottom layer modulo monolayer lattice vectors

$$\delta\mathbf{R}(\mathbf{r}) \equiv \sum_{i=1}^{2} \frac{1}{2\pi} \left[\left(\mathcal{R}_{\theta,-}^{-1}\delta\mathbf{r}_- - \mathcal{R}_{\theta,+}^{-1}\delta\mathbf{r}_+ \right) \cdot \mathbf{b}_i \right] \mathbf{a}_i. \tag{S4.53}$$

The relative displacement $\delta\mathbf{R}(\mathbf{r})$ is only defined up to a direct monolayer lattice vector.

A simpler form for the relative displacement can be derived using Eq. (S4.52)

$$\begin{aligned}\delta\mathbf{R}(\mathbf{r}) &= \sum_{j}^{2} \frac{1}{2\pi} \left\{ \sum_{i=1}^{2} \left[\frac{1}{2\pi}\mathbf{a}_i \left(\mathbf{b}_i \cdot \mathcal{R}_{\theta,-}^{-1}\mathbf{r} \bmod 2\pi \right) - \frac{1}{2\pi}\mathbf{a}_i \left(\mathbf{b}_i \cdot \mathcal{R}_{\theta,+}^{-1}\mathbf{r} \bmod 2\pi \right) \right] \cdot \mathbf{b}_j \right\} \mathbf{a}_j \\ &= \sum_{i=1}^{2} \frac{1}{2\pi}\mathbf{a}_i \left[\mathbf{b}_i \cdot \left(\mathcal{R}_{\theta,-}^{-1} - \mathcal{R}_{\theta,+}^{-1} \right) \mathbf{r} \bmod 2\pi \right] \\ &= \sum_{i=1}^{2} \frac{1}{2\pi}\mathbf{a}_i \left[\mathbf{b}_i \cdot \left(2\sin\left(\frac{\theta}{2}\right) \hat{\mathbf{z}} \times \mathbf{r} \right) \mod 2\pi \right] \\ &= \sum_{i=1}^{2} \frac{1}{2\pi}\mathbf{a}_i \left[\mathbf{r} \cdot \left(\mathbf{b}_i \times 2\sin\left(\frac{\theta}{2}\right) \hat{\mathbf{z}} \right) \mod 2\pi \right]. \end{aligned} \tag{S4.54}$$

The periodicity of the moiré pattern arises from the periodicity of the local displacement in $\mathbf{r}$. For any vector $\Delta\mathbf{r}$ that satisfies

$$\Delta\mathbf{r} \cdot \left(\mathbf{b}_i \times 2\sin\left(\frac{\theta}{2}\right) \hat{\mathbf{z}} \right) \in 2\pi\mathbb{Z} \quad \text{for any} \quad i = 1,2, \tag{S4.55}$$

we must have that $\delta\mathbf{R}(\mathbf{r} + \Delta\mathbf{r}) = \delta\mathbf{R}(\mathbf{r})$. The vectors $\Delta\mathbf{r}$ satisfying Eq. (S4.55) form the moiré direct lattice, which is spanned by $\mathbf{a}_{M_i}$ (with $i = 1,2$). To find $\mathbf{a}_{M_i}$, we note that Eq. (S4.55) also implies that the reciprocal moiré lattice is spanned by $\mathbf{b}_i \times 2\sin\left(\frac{\theta}{2}\right)\hat{\mathbf{z}}$, for $i = 1,2$. Using the auxiliary vectors from Eq. (S4.27), we define the reciprocal moiré lattice vectors as

$$\mathbf{b}_{M_1} = \mathbf{b}_2 \times 2\sin\left(\frac{\theta}{2}\right)\hat{\mathbf{z}} = 2\mathbf{q}_0, \qquad \mathbf{b}_{M_2} = (\mathbf{b}_2 - \mathbf{b}_1) \times 2\sin\left(\frac{\theta}{2}\right)\hat{\mathbf{z}} = -2\mathbf{q}_2, \tag{S4.56}$$

which generate the corresponding moiré reciprocal lattice $\mathcal{Q} = \mathbb{Z}\mathbf{b}_{M_1} + \mathbb{Z}\mathbf{b}_{M_2}$. At the same time, the direct moiré lattice vectors $\mathbf{a}_{M_i}$ are defined to satisfy $\mathbf{a}_{M_i} \cdot \mathbf{b}_{M_j} = 2\pi\delta_{ij}$, for $1 \leq i,j \leq 2$.

1. Model in momentum space

We are now in a position of writing down the Hamiltonian of the twisted M-point bilayer system. To this end, we introduce *three* momentum lattices, as shown in Fig. S15,

$$\mathcal{Q}_n \equiv \mathcal{Q} + \mathbf{q}_n, \quad \text{for} \quad 0 \leq n \leq 2, \tag{S4.57}$$

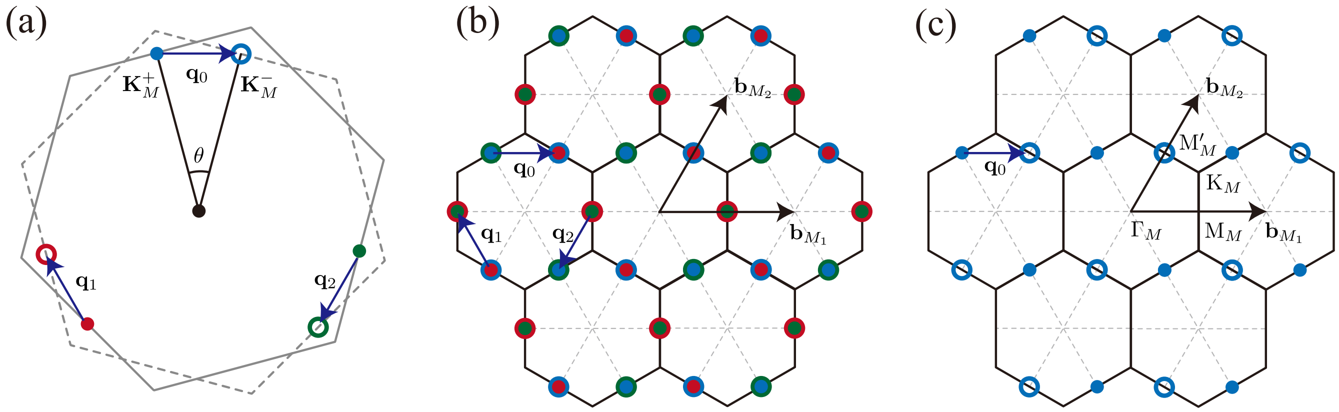

FIG. S15. The moiré BZ and momentum lattices generated by twisting the M valley. In (a), the gray continuous (dashed) hexagons correspond to the BZs of the top (bottom) layers. The M valleys of the two layers located at $\mathbf{K}_M^{\pm}$ are shown explicitly, together with the auxiliary $\mathbf{q}_\eta$ vectors defined in Eq. (S4.27). The three inequivalent M valleys are shown in solid (hollow) blue, green, and red for the top (bottom) layers. (b) shows the three momentum lattices defined in Eq. (S4.57), as well as the reciprocal moiré lattice vectors. Each lattice site corresponds to *two* M valleys from different layers. For clarity, the momentum lattice corresponding to valley $\eta = 0$ is shown in (c), where we mark the high-symmetry momentum $\Gamma = (0,0), \mathrm{M}_M = \frac{1}{2}\mathbf{b}_{M_1}, \mathrm{M}'_M = \frac{1}{2}\mathbf{b}_{M_2}, \mathrm{K}_M = \frac{1}{3}(\mathbf{b}_{M_1} + \mathbf{b}_{M_2})$.

where the $\mathbf{q}_n$ vectors were defined in Eq. (S4.27) and which together form a kagome lattice[4]

$$\mathcal{Q}_{\text{tot}} \equiv \bigcup_{n=0}^{2} \mathcal{Q}_n. \tag{S4.58}$$

For later convenience in notation, we also extend the definitions in Eqs. (S4.27) and (S4.57) beyond $0 \leq \eta \leq 2$ and $0 \leq n \leq 2$, respectively, using

$$\mathcal{Q}_n \equiv \mathcal{Q}_{n \bmod 3} \quad \text{and} \quad \mathbf{q}_n \equiv \mathbf{q}_{n \bmod 3}, \quad \text{for} \quad n \in \mathbb{Z}. \tag{S4.59}$$

For every $\mathbf{Q} \in \mathcal{Q}_{\text{tot}}$, we also associate a sublattice factor defined as

$$\zeta_{\mathbf{Q}} = n, \quad \text{for} \quad \mathbf{Q} \in \mathcal{Q}_n, \quad \text{with} \quad 0 \leq n \leq 2. \tag{S4.60}$$

Similarly to TBG [31], we define the following low-energy operators

$$\hat{c}^\dagger_{\mathbf{k},\mathbf{Q},s,l} \equiv \hat{a}^\dagger_{C_{3z}^\eta \mathbf{K}_M^l + \mathbf{k} - \mathbf{Q},s,l}, \quad \text{for} \quad \mathbf{Q} \in \mathcal{Q}_{\eta+l} \quad \text{and} \quad \mathbf{k} \in \text{MBZ}, \tag{S4.61}$$

where MBZ denotes the first moiré BZ. The definition of the $\hat{c}^\dagger_{\mathbf{k},\mathbf{Q},s,l}$ operators can be extended outside the first MBZ through the following embedding relation

$$\hat{c}^\dagger_{\mathbf{k}+\mathbf{G},\mathbf{Q},s,l} = \hat{c}^\dagger_{\mathbf{k},\mathbf{Q}-\mathbf{G},s,l}, \quad \text{for} \quad \mathbf{G} \in \mathcal{Q}. \tag{S4.62}$$

Note that the $\hat{c}^\dagger_{\mathbf{k},\mathbf{Q},s,l}$ fermions do feature a layer index l, but not a valley η index. This is because the valley η associated with $\hat{c}^\dagger_{\mathbf{k},\mathbf{Q},s,l}$ can be directly inferred from $\mathbf{Q}$ and l: $\eta = \zeta_{\mathbf{Q}} - l$[5]. More intuitively, the $\hat{c}^\dagger_{\mathbf{k},\mathbf{Q},s,l}$ from valley η are supported on the complement of lattice $\mathcal{Q}_\eta$, meaning that $\mathbf{Q} \in \mathcal{Q}_{\text{tot}}/\mathcal{Q}_\eta$.

---

[4] It is crucial to highlight the distinction between Eq. (S4.57) and the $\mathcal{Q}'_{\pm}$ lattices that emerge when twisting the K valley (such as in TBG [31] or in MoTe_2 [5, 14, 15]). As illustrated in Fig. 1, the reciprocal moiré vectors are identical in both scenarios, being given by $\mathbf{b}_{M_i}$ for $i = 1, 2$. However, the three $\mathbf{q}_n^{(\prime)} = C_{3z}^{n-1}\mathbf{q}_1^{(\prime)}$ vectors are defined differently between the two cases: $\mathbf{q}'_1 = \frac{1}{3}\left(\mathbf{b}_{M_1} - 2\mathbf{b}_{M_2}\right)$, while $\mathbf{q}_1 = \frac{1}{2}\left(-\mathbf{b}_{M_1} + \mathbf{b}_{M_2}\right)$. These differences in the lengths and orientations of the $\mathbf{q}_n^{(\prime)}$ vectors lead to the generation of a kagome lattice for M-point twisting, as opposed to a honeycomb $\mathbf{Q}$ lattice for the K-valley case.

[5] This is similar to TBG [31], where knowing the valley η and the $\mathbf{Q}$ vector of a given low-energy fermionic operator $\hat{c}^\dagger_{\mathbf{k},\mathbf{Q},\alpha,\eta,s}$ fully specifies the layer l.

Depending on the stacking arrangement, we employ Eqs. (S4.20) and (S4.31) to write the Hamiltonian of the twisted bilayer heterostructure in the two-center first monolayer harmonic approximation. For AA-stacking, we have

$$\begin{aligned}\mathcal{H}_{\mathrm{AA}} =& \sum_{\delta\mathbf{k},\delta\mathbf{k}'} \sum_{\substack{s_1,l_1,\eta_1\\ s_2,l_2,\eta_2}} \left[h^{\mathrm{bl}}_{\mathrm{AA}}\left(C_{3z}^{\eta_1}\mathbf{K}_M^{l_1}+\delta\mathbf{k}, C_{3z}^{\eta_2}\mathbf{K}_M^{l_2}+\delta\mathbf{k}'\right)\right]_{s_1l_1;s_2l_2} \hat{a}^\dagger_{C_{3z}^{\eta_1}\mathbf{K}_M^{l_1}+\delta\mathbf{k},s_1,l_1}\hat{a}_{C_{3z}^{\eta_2}\mathbf{K}_M^{l_2}+\delta\mathbf{k}',s_2,l_2}\\ =&\sum_{\delta\mathbf{k}}\sum_{s_1,s_2,\eta,l}\left[h^{\mathrm{sl}}\left(C_{3z}^{\eta}\mathbf{K}_M+\mathcal{R}_{\theta,l}^{-1}\delta\mathbf{k}\right)\right]_{s_1s_2}\hat{a}^\dagger_{C_{3z}^{\eta}\mathbf{K}_M^{l}+\delta\mathbf{k},s_1,l}\hat{a}_{C_{3z}^{\eta}\mathbf{K}_M^{l}+\delta\mathbf{k},s_2,l}\\ &+\sum_{\delta\mathbf{k}}\sum_{s_1,s_2,\eta,l}\sum_{m=0}^{1}\tilde{t}^{l,\mathrm{AA}}_{s_1s_2}\left((-1)^m C_{3z}^{\eta}\mathbf{K}_M\right)\hat{a}^\dagger_{C_{3z}^{\eta}\mathbf{K}_M^{l}+\delta\mathbf{k}+l(-1)^m\mathbf{q}_\eta,s_1,l}\hat{a}_{C_{3z}^{\eta}\mathbf{K}_M^{-l}+\delta\mathbf{k},s_2,-l},\end{aligned} \tag{S4.63}$$

where the rotation matrix $\mathcal{R}_{\theta,l}$ is defined in Eq. (S4.17). For AB-stacking, we similarly obtain

$$\begin{aligned}\mathcal{H}_{\mathrm{AB}} =&\sum_{\delta\mathbf{k}}\sum_{s_1,s_2,\eta,l}\left[h^{\mathrm{sl}}\left(lC_{3z}^{\eta}\mathbf{K}_M+l\mathcal{R}_{\theta,l}^{-1}\delta\mathbf{k}\right)\right]_{s_1s_2}\hat{a}^\dagger_{C_{3z}^{\eta}\mathbf{K}_M^{l}+\delta\mathbf{k},s_1,l}\hat{a}_{C_{3z}^{\eta}\mathbf{K}_M^{l}+\delta\mathbf{k},s_2,l}\\ &+\sum_{\delta\mathbf{k}}\sum_{s_1,s_2,\eta,l}\sum_{m=0}^{1}\tilde{t}^{l,\mathrm{AB}}_{s_1s_2}\left((-1)^m C_{3z}^{\eta}\mathbf{K}_M\right)\hat{a}^\dagger_{C_{3z}^{\eta}\mathbf{K}_M^{l}+\delta\mathbf{k}+l(-1)^m\mathbf{q}_\eta,s_1,l}\hat{a}_{C_{3z}^{\eta}\mathbf{K}_M^{-l}+\delta\mathbf{k},s_2,-l}.\end{aligned} \tag{S4.64}$$

The last term in both Eqs. (S4.63) and (S4.64) can be recast in the notation of Eq. (S4.61) as

$$\begin{aligned}&\sum_{\delta\mathbf{k}}\sum_{s_1,s_2,\eta,l}\sum_{m=0}^{1}\tilde{t}^{l}_{s_1s_2}\left((-1)^m C_{3z}^{\eta}\mathbf{K}_M\right)\hat{a}^\dagger_{C_{3z}^{\eta}\mathbf{K}_M^{l}+\delta\mathbf{k}+l(-1)^m\mathbf{q}_\eta,s_1,l}\hat{a}_{C_{3z}^{\eta}\mathbf{K}_M^{-l}+\delta\mathbf{k},s_2,-l}\\ =&\sum_{\mathbf{k},\eta,l}\sum_{\mathbf{Q}\in\mathcal{Q}_{\eta+l}}\sum_{s_1,s_2}\sum_{m=0}^{1}\tilde{t}^{-l}_{s_1s_2}\left((-1)^m C_{3z}^{\eta}\mathbf{K}_M\right)\hat{c}^\dagger_{\mathbf{k},\mathbf{Q}+l(-1)^m\mathbf{q}_\eta,s_1,-l}\hat{c}_{\mathbf{k},\mathbf{Q},s_2,l},\end{aligned} \tag{S4.65}$$

which holds for both AA- and AB-stacking and where we have made the substitution $\delta\mathbf{k}=\mathbf{k}-\mathbf{Q}$ as well as $l\to(-l)$. Note that if $\mathbf{Q}\in\mathcal{Q}_{\eta+l}$, such that $\mathbf{Q}=\mathbf{G}+\mathbf{q}_{\eta+l}$ (with $\mathbf{G}\in\mathcal{Q}$), then

$$\mathbf{Q}\pm\mathbf{q}_\eta=\mathbf{G}+\mathbf{q}_{\eta+l}+\mathbf{q}_\eta-(\mathbf{q}_\eta\mp\mathbf{q}_\eta)=[\mathbf{G}-(\mathbf{q}_\eta\mp\mathbf{q}_\eta)]-\mathbf{q}_{\eta-l}\in\mathcal{Q}_{\eta-l}. \tag{S4.66}$$

This implies that both the $\hat{c}_{\mathbf{k},\mathbf{Q},s_2,l}$ and $\hat{c}^\dagger_{\mathbf{k},\mathbf{Q}+l(-1)^m\mathbf{q}_\eta,s_1,-l}$ fermions belong to valley η. Eq. (S4.65) allows us to rewrite the Hamiltonian of the entire moiré system as

$$\mathcal{H}=\sum_{\mathbf{k}\in\mathrm{MBZ}}\sum_{s_1,s_2}\sum_{\mathbf{Q},\mathbf{Q}'\in\mathcal{Q}_{\mathrm{tot}}}\left[h_{\mathbf{Q},\mathbf{Q}'}(\mathbf{k})\right]_{s_1l_1;s_2l_2}\hat{c}^\dagger_{\mathbf{k},\mathbf{Q},s_1,l_1}\hat{c}_{\mathbf{k},\mathbf{Q}',s_2,l_2}, \tag{S4.67}$$

where the first-quantized Hamiltonian $h_{\mathbf{Q},\mathbf{Q}'}(\mathbf{k})$ is given by

$$\left[h_{\mathbf{Q},\mathbf{Q}'}(\mathbf{k})\right]_{s_1l_1;s_2l_2}=\delta_{\mathbf{Q},\mathbf{Q}'}\delta_{s_1s_2}\delta_{l_1l_2}\left(\frac{\delta k_x^2}{2m_x}+\frac{\delta k_y^2}{2m_y}\right)\Bigg|_{\delta\mathbf{k}=C_{3z}^{l_1-\zeta_{\mathbf{Q}}}\mathcal{R}_{\theta,l_1}^{-1}(\mathbf{k}-\mathbf{Q})}+\left[T_{\mathbf{Q},\mathbf{Q}'}\right]_{s_1l_1;s_2l_2}, \tag{S4.68}$$

and the momentum space lattice $\mathcal{Q}_{\mathrm{tot}}$ was defined in Eq. (S4.58). The form of the first-quantized Hamiltonian from Eq. (S4.68) is generically valid even beyond the two-center first monolayer harmonic approximation. In the case of the latter, the moiré potential term $T_{\mathbf{Q},\mathbf{Q}'}$ can be directly determined from Eqs. (S4.49) and (S4.50)

$$\left[T_{\mathbf{Q}',\mathbf{Q}}\right]_{s_1l_1;s_2l_2}=\tilde{t}^{l_1}\left((-1)^m C_{3z}^{\eta}\mathbf{K}_M\right)\delta_{\mathbf{Q}',\mathbf{Q}+l_2(-1)^m\mathbf{q}_\eta}\delta_{l_1(-l_2)},\quad\text{for}\quad\mathbf{Q}\in\mathcal{Q}_{\eta+l_2}, \tag{S4.69}$$

or explicitly for valley $\eta=0$

$$\begin{aligned}\left[T^{\mathrm{AA}}_{\mathbf{Q}',\mathbf{Q}}\right]_{s_1(-l);s_2l}=&\delta_{\mathbf{Q}',\mathbf{Q}\pm l\mathbf{q}_0}\left[\left(\mp ilw_1^{\mathrm{AA}}+w_2^{\mathrm{AA}}\right)s_0\pm\left(w_4^{\mathrm{AA}}+w_6^{\mathrm{AA}}e^{\pm il\frac{\pi}{6}}\right)s_y+\left(-ilw_3^{\mathrm{AA}}\pm w_5^{\mathrm{AA}}\right)s_z\right]_{s_1s_2}, &\text{(S4.70)}\\ \left[T^{\mathrm{AB}}_{\mathbf{Q}',\mathbf{Q}}\right]_{s_1(-l);s_2l}=&\delta_{\mathbf{Q}',\mathbf{Q}\pm l\mathbf{q}_0}\left[w_2^{\mathrm{AB}}s_0-ilw_1^{\mathrm{AB}}s_x\pm w_3^{\mathrm{AB}}s_y-ilw_4^{\mathrm{AB}}s_z\right]_{s_1s_2}, &\text{(S4.71)}\end{aligned}$$

where $\mathbf{Q}\in\mathcal{Q}_l$. The moiré potential in the other valleys can be obtained using the C_{3z} symmetry, as will be explained around Eq. (S5.91). Because the inter-valley coupling vanishes at the single-particle level, the moiré potential term *generally* (*i.e.*, even *beyond* the two-center first monolayer harmonic approximation) obeys the following property

$$\left[T_{\mathbf{Q}',\mathbf{Q}}\right]_{s_1l_1;s_2l_2}=0,\quad\text{for}\quad\mathbf{Q}\in\mathcal{Q}_{\eta_1+l_1}\quad\text{and}\quad\mathbf{Q}'\in\mathcal{Q}_{\eta_2+l_2},\quad\text{with}\quad\eta_1\neq\eta_2. \tag{S4.72}$$

2. Model in real space

It is also useful for the following discussion to recast the model obtained in Section IV C 1 from momentum to real space. For this purpose, we introduce the following real-space fermion operators

$$\hat{\psi}^\dagger_{\eta,s,l}(\mathbf{r}) = \frac{1}{\sqrt{\Omega}} \sum_{\mathbf{k}\in\mathrm{MBZ}} \sum_{\mathbf{Q}\in\mathcal{Q}_{\eta+l}} \hat{c}^\dagger_{\mathbf{k},\mathbf{Q},s,l} e^{-i(\mathbf{k}-\mathbf{Q})\cdot\mathbf{r}}, \tag{S4.73}$$

$$\hat{c}^\dagger_{\mathbf{k},\mathbf{Q},s,l} = \frac{1}{\sqrt{\Omega}} \int \mathrm{d}^2 r\, \hat{\psi}^\dagger_{\eta,s,l}(\mathbf{r}) e^{i(\mathbf{k}-\mathbf{Q})\cdot\mathbf{r}}, \quad \text{for} \quad \mathbf{Q}\in\mathcal{Q}_{\eta+l}, \tag{S4.74}$$

where Ω is the area of the moiré heterostructure. The operator $\hat{\psi}^\dagger_{\eta,s,l}(\mathbf{r})$ creates a fermion of spin s at position $\mathbf{r}$, within layer l and valley η. The real space Hamiltonian then takes the form

$$\begin{aligned}\mathcal{H} = &-\sum_{\eta,s,l} \int \mathrm{d}^2 r\, \hat{\psi}^\dagger_{\eta,s,l}(\mathbf{r}) \left(C_{3z}^{-\eta}\mathcal{R}^{-1}_{\theta,l}\nabla\right)^T \begin{pmatrix} \frac{1}{2m_x} & 0 \\ 0 & \frac{1}{2m_y} \end{pmatrix} \left(C_{3z}^{-\eta}\mathcal{R}^{-1}_{\theta,l}\nabla\right) \hat{\psi}_{\eta,s,l}(\mathbf{r}) \\ &+ \sum_{\substack{\eta,s_1,s_2\\ l_1,l_2}} \int \mathrm{d}^2 r\, V^\eta_{s_1 l_1; s_2 l_2}(\mathbf{r}) \hat{\psi}^\dagger_{\eta,s_1,l_1}(\mathbf{r}) \hat{\psi}_{\eta,s_2,l_2}(\mathbf{r}),\end{aligned} \tag{S4.75}$$

with the real-space moiré potential being given by

$$V^\eta_{s_1 l_1; s_2 l_2}(\mathbf{r}) = \sum_{\mathbf{G}\in\mathcal{Q}} \left[T_{\mathbf{q}_{\eta+l_1},\mathbf{q}_{\eta+l_2}+\mathbf{G}}\right]_{s_1 l_1; s_2 l_2} e^{i\left(\mathbf{q}_{\eta+l_2}+\mathbf{G}-\mathbf{q}_{\eta+l_1}\right)\cdot\mathbf{r}}, \tag{S4.76}$$

where we have employed Eq. (S4.59). It is easy to check that the real space moiré potential obeys the following periodicity property on the moiré lattice scale

$$V^\eta_{s_1 l_1; s_2 l_2}(\mathbf{r}+\mathbf{R}_M) = V^\eta_{s_1 l_1; s_2 l_2}(\mathbf{r}) e^{i\left(\mathbf{q}_{\eta+l_2}-\mathbf{q}_{\eta+l_1}\right)\cdot\mathbf{R}_M}, \quad \text{for any} \quad \mathbf{R}_M \in \mathbb{Z}\mathbf{a}_{M_1} + \mathbb{Z}\mathbf{a}_{M_2}, \tag{S4.77}$$

or, alternatively,

$$V^\eta_{s_1 l; s_2 l}(\mathbf{r}+\mathbf{R}_M) = V^\eta_{s_1 l; s_2 l}(\mathbf{r}), \tag{S4.78}$$

$$V^\eta_{s_1 l; s_2 (-l)}(\mathbf{r}+\mathbf{R}_M) = V^\eta_{s_1 l; s_2 (-l)}(\mathbf{r}) e^{i(2\mathbf{q}_{\eta-l}-\mathbf{q}_{\eta+l}-\mathbf{q}_{\eta-l})\cdot\mathbf{R}_M} = V^\eta_{s_1 l; s_2 (-l)}(\mathbf{r}) e^{i\mathbf{q}_\eta\cdot\mathbf{R}_M}, \tag{S4.79}$$

for any $\mathbf{R}_M \in \mathbb{Z}\mathbf{a}_{M_1} + \mathbb{Z}\mathbf{a}_{M_2}$.

3. Exact symmetries of the model

The *continuum* model features a series of *exact* symmetries depending on the stacking arrangement. The action of a symmetry transformation g on the low-energy fermions introduced in Eq. (S4.61) is given by

$$g\hat{c}^\dagger_{\mathbf{k},\mathbf{Q},s_1,l_1} g^{-1} = \sum_{s_2,l_2} \left[D(g)\right]_{s_2 l_2; s_1 l_1} \hat{c}^\dagger_{g\mathbf{k},g\mathbf{Q},s_2,l_2}. \tag{S4.80}$$

In both the AA- and AB-stacking arrangements, the system features C_{3z} and $\mathcal{T}$ symmetries whose representation matrices are given in both cases by

$$D(\mathcal{T}) = i s_y \sigma_0, \quad D(C_{3z}) = e^{-\frac{\pi i}{3} s_z} \sigma_0, \tag{S4.81}$$

where here and in what follows, we define σ_a (for $a = 0, x, y, z$) to be the identity and the three Pauli matrices acting on the layer subspace.

Additionally, in the AA-stacked case, the system features C_{2x} symmetry, while in the AB-stacked case, the system has C_{2y} symmetry. The representation matrices of these symmetries read as

$$D(C_{2x}) = -i s_x \sigma_x, \quad D(C_{2y}) = -i s_y \sigma_x. \tag{S4.82}$$

The action of these symmetries on the real space fermions defined in Eq. (S4.73) is given by

$$g\hat{\psi}^{\dagger}_{\eta_1,s_1,l_1}(\mathbf{r})\,g^{-1} = \sum_{\eta_2,s_2,l_2} [D_{\psi}(g)]_{\eta_2 s_2 l_2;\eta_1 s_1 l_1}\,\hat{\psi}^{\dagger}_{\eta_2,s_2,l_2}(g\mathbf{r})\,, \tag{S4.83}$$

with the corresponding real space representation matrices being given by

$$D_{\psi}(\mathcal{T})=i\begin{pmatrix}1&0&0\\0&1&0\\0&0&1\end{pmatrix}s_y\sigma_0,\qquad D_{\psi}(C_{3z})=\begin{pmatrix}0&0&1\\1&0&0\\0&1&0\end{pmatrix}e^{-\frac{\pi i}{3}s_z}\sigma_0,$$
$$D_{\psi}(C_{2x})=-i\begin{pmatrix}1&0&0\\0&0&1\\0&1&0\end{pmatrix}s_x\sigma_x,\quad D_{\psi}(C_{2y})=-i\begin{pmatrix}1&0&0\\0&0&1\\0&1&0\end{pmatrix}s_y\sigma_x, \tag{S4.84}$$

Finally, in both the AA- and AB-stacking cases, the model also features discrete moiré translation symmetry

$$T_{\mathbf{R}_M}\hat{c}^{\dagger}_{\mathbf{k},\mathbf{Q},s,l}T^{-1}_{\mathbf{R}_M} = \hat{c}^{\dagger}_{\mathbf{k},\mathbf{Q},s,l}e^{-i\left(C^{\eta}_{3z}\mathbf{K}^{l}_{M}+\mathbf{k}-\mathbf{Q}\right)\cdot\mathbf{R}_M}, \tag{S4.85}$$
$$T_{\mathbf{R}_M}\hat{\psi}^{\dagger}_{\eta,s,l}(\mathbf{r})\,T^{-1}_{\mathbf{R}_M} = \hat{\psi}^{\dagger}_{\eta,s,l}(\mathbf{r}+\mathbf{R}_M)\,e^{-iC^{\eta}_{3z}\mathbf{K}^{l}_{M}\cdot\mathbf{R}_M}, \tag{S4.86}$$

where $T_{\mathbf{R}_M}$ denotes the discrete moiré translation operator, which translates by the moiré lattice vector $\mathbf{R}_M \in \mathbb{Z}\mathbf{a}_{M_1} + \mathbb{Z}\mathbf{a}_{M_2}$. Eq. (S4.85) can be proved by assuming a commensurate configuration such that $\mathbf{R}_M$ is also a lattice vector of the two monolayers' Bravais lattices. By definition, the action of the translation operator $T_{\mathbf{R}_M}$ on the monolayer Wannier orbital operators is given by

$$T_{\mathbf{R}_M}\hat{a}^{\dagger}_{\mathbf{R},s,l}T^{-1}_{\mathbf{R}_M} = \hat{a}^{\dagger}_{\mathbf{R}+\mathcal{R}^{-1}_{\theta,l}\mathbf{R}_M,s,l} \quad\text{or}\quad T_{\mathbf{R}_M}\hat{a}^{\dagger}_{\mathbf{R},s,l}T^{-1}_{\mathbf{R}_M} = \hat{a}^{\dagger}_{\mathbf{R}+l\mathcal{R}^{-1}_{\theta,l}\mathbf{R}_M,s,l}, \tag{S4.87}$$

for AA- or AB-stacking, respectively. Using the Fourier transformations from Eqs. (S4.18) and (S4.30), we can determine the action of the translation operator on the momentum space operators for both AA- and AB-stacking

$$T_{\mathbf{R}_M}\hat{a}^{\dagger}_{\mathbf{k},s,l}T^{-1}_{\mathbf{R}_M} = \hat{a}^{\dagger}_{\mathbf{k},s,l}e^{-i\mathbf{k}\cdot\mathbf{R}_M}. \tag{S4.88}$$

From this, Eq. (S4.85) directly follows using the definition in Eq. (S4.61). In the continuum limit, Eq. (S4.85) holds even when the commensuration condition is relaxed.

As mentioned already in Section IV B, together with the moiré translation symmetry, the exact symmetries discussed in this section give rise to the $P3121'$ (SSG 149.22) and $P3211'$ (SSG 150.26) groups in the AA- and AB-stacked cases, respectively[6]. Additionally, we note that because the three M valleys are decoupled at the single-particle level, the system also features a $\mathrm{U}(1)\times\mathrm{U}(1)\times\mathrm{U}(1)$ valley-charge symmetry in both the AA- and AB-stacking cases. Because valley is a good quantum number, we can restrict ourselves to a single valley (which, without loss of generality, we choose to be $\eta = 0$). The single-valley system only features C_{2x} (C_{2y}) and $\mathcal{T}$ symmetries in the AA- (AB-)stacked case, which give rise to the $P21'$ (SSG 3.2) single-valley symmetry group.

V. DIRECT GENERAL DERIVATION OF THE MOIRÉ POTENTIAL WITHOUT GRADIENT TERMS

In Section IV C we have derived BM models for the twisted $SnSe_2$ and ZrS_2 and heterostructures using the two-center first monolayer harmonic approximation for the interlayer tunneling. The form of the latter was derived in Section IV B by employing the symmetries of the $\theta \neq 0$ or $\theta = 0$ heterostructures. In this section, we will employ a similar strategy to *directly* constrain the moiré potential term $T_{\mathbf{Q}',\mathbf{Q}}$ defined in Eq. (S4.68). By doing so, not only the interlayer, but also any *intralayer* moiré potential contributions (arising, for example, from lattice relaxation effects) can be constrained using symmetry considerations. The intralayer moiré potential contributions are zero in the two-center first monolayer harmonic approximation. Throughout this section, we will also assume that the moiré potential has the form given by the second row of Eq. (S4.75). The reader is referred to the following Section VII

---

[6] To avoid any ambiguities, we define the rotation axes using the Cartesian coordinate system. This is because the moiré unit cell basis vectors and the single-layer unit cell ones are different, as explained in the beginning of Section IV C.

for a more general derivation that includes gradient terms in the moiré potential (which will also be discussed and explicitly defined therein).

Similarly to Section IV B 1, we will first use the exact symmetries of the $\theta \neq 0$ heterostructure to obtain all the symmetry allowed terms (including the intralayer terms) of the moiré potential up to the first harmonic (which will be defined below). We will then consider the case of small twist angles, for which the enhanced symmetries of the $\theta = 0$ heterostructure further constrain the moiré potential. The main results of this section are general symmetry-obeying parameterizations of both the intralayer and interlayer moiré potentials.

A. General form of the moiré potential restricted by the exact $\theta \neq 0$ symmetries of the heterostructure

We begin by constraining the moiré potential using the exact symmetries of the problem arising for non-zero twist angles. For starters, the moiré potential matrix must be Hermitian

$$[T_{\mathbf{Q},\mathbf{Q}'}]_{s_1 l_1; s_2 l_2} = [T^{\dagger}_{\mathbf{Q}',\mathbf{Q}}]_{s_1 l_1; s_2 l_2}, \tag{S5.89}$$

and obey the moiré periodicity

$$T_{\mathbf{Q},\mathbf{Q}'} = T_{\mathbf{Q}+\mathbf{G},\mathbf{Q}'+\mathbf{G}}, \quad \text{for} \quad \mathbf{G} \in \mathcal{Q}. \tag{S5.90}$$

For every crystalline symmetry g of the twisted structure, whose action on the moiré fermions is given by Eq. (S4.80), the moiré potential will satisfy

$$T_{g\mathbf{Q},g\mathbf{Q}'} = D(g) T^{(*)}_{\mathbf{Q},\mathbf{Q}'} D^{\dagger}(g), \tag{S5.91}$$

where $^{(*)}$ indicates that a complex conjugation should be taken in the cases when g is antiunitary. Additionally, the $\mathrm{U}(1) \times \mathrm{U}(1) \times \mathrm{U}(1)$ valley-charge symmetry will require that the moiré potential always satisfies Eq. (S4.72).

Using the symmetry constraints of Eqs. (S4.72) and (S5.89) to (S5.91) we can construct the most general form of the moiré potential. For simplicity, we restrict ourselves to first moiré harmonic terms, *i.e.* such that

$$[T_{\mathbf{Q},\mathbf{Q}'}]_{s_1 l_1; s_2 l_2} = 0, \quad \text{for} \quad |\mathbf{Q} - \mathbf{Q}'| > |\mathbf{b}_{M_1}|. \tag{S5.92}$$

We find that compared to the two-center first monolayer harmonic approximation from Eqs. (S4.70) and (S4.71), Eqs. (S5.94) and (S5.95) contains ten additional real parameters in the AA-stacked case and twelve additional ones in the AB-stacked case. Keeping the same notation as in Eqs. (S5.94) and (S5.95) for the parameters characterizing the first monolayer harmonic terms (*i.e.*, w_i^{AA} with $1 \leq i \leq 6$ and w_i^{AB} with $1 \leq i \leq 4$), we denote the additional real parameters by

$$w_i'^{\mathrm{AA}}, \quad \text{with} \quad 1 \leq i \leq 10 \quad \text{and} \quad w_i'^{\mathrm{AB}}, \quad \text{with} \quad 1 \leq i \leq 12. \tag{S5.93}$$

Depending on the stacking configuration, the potential in the first moiré Harmonic approximation is given by

$$\begin{aligned}
\left[T^{\mathrm{AA}}_{\mathbf{q}_{-1},\mathbf{q}_{+1}-\mathbf{b}_{M_2}}\right]_{s_1(-);s_2(+)} &= \left[\left(w_2^{\mathrm{AA}} - i w_1^{\mathrm{AA}}\right) s_0 + \left(w_4^{\mathrm{AA}} + \frac{1}{2}\left(\sqrt{3}+i\right) w_6^{\mathrm{AA}}\right) s_y + \left(w_5^{\mathrm{AA}} - i w_3^{\mathrm{AA}}\right) s_z\right]_{s_1 s_2} \\
\left[T^{\mathrm{AA}}_{\mathbf{q}_{-1},\mathbf{q}_{+1}}\right]_{s_1(-);s_2(+)} &= \left[s_0 w_3'^{\mathrm{AA}} + \frac{1}{2} s_x \left(-w_8'^{\mathrm{AA}} - w_9'^{\mathrm{AA}}\right) + \frac{1}{2} i s_y \left(w_8'^{\mathrm{AA}} - w_9'^{\mathrm{AA}}\right) - i s_z w_6'^{\mathrm{AA}}\right]_{s_1 s_2} \\
\left[T^{\mathrm{AA}}_{\mathbf{q}_{-1},\mathbf{b}_{M_2}+\mathbf{q}_{-1}}\right]_{s_1(-);s_2(-)} &= \left[s_0 \left(-i w_1'^{\mathrm{AA}} + w_4'^{\mathrm{AA}} - i w_7'^{\mathrm{AA}} + w_{10}'^{\mathrm{AA}}\right)\right]_{s_1 s_2} \\
\left[T^{\mathrm{AA}}_{\mathbf{q}_{+1},\mathbf{b}_{M_2}+\mathbf{q}_{+1}}\right]_{s_1(+);s_2(+)} &= \left[s_0 \left(-i w_1'^{\mathrm{AA}} + w_4'^{\mathrm{AA}} + i w_7'^{\mathrm{AA}} - w_{10}'^{\mathrm{AA}}\right)\right]_{s_1 s_2} \\
\left[T^{\mathrm{AA}}_{\mathbf{q}_{-1},\mathbf{b}_{M_1}-2\mathbf{b}_{M_2}+\mathbf{q}_{+1}}\right]_{s_1(-);s_2(+)} &= \left[s_0 w_3'^{\mathrm{AA}} + \frac{1}{2} s_x \left(w_8'^{\mathrm{AA}} + w_9'^{\mathrm{AA}}\right) + \frac{1}{2} i s_y \left(w_8'^{\mathrm{AA}} - w_9'^{\mathrm{AA}}\right) - i s_z w_6'^{\mathrm{AA}}\right]_{s_1 s_2} \\
\left[T^{\mathrm{AA}}_{\mathbf{q}_{-1},\mathbf{b}_{M_1}-\mathbf{b}_{M_2}+\mathbf{q}_{-1}}\right]_{s_1(-);s_2(-)} &= \left[s_0 \left(-i w_1'^{\mathrm{AA}} + w_4'^{\mathrm{AA}} + i w_7'^{\mathrm{AA}} - w_{10}'^{\mathrm{AA}}\right)\right]_{s_1 s_2} \\
\left[T^{\mathrm{AA}}_{\mathbf{q}_{-1},\mathbf{b}_{M_1}-\mathbf{b}_{M_2}+\mathbf{q}_{+1}}\right]_{s_1(-);s_2(+)} &= \left[\left(w_2^{\mathrm{AA}} + i w_1^{\mathrm{AA}}\right) s_0 + \left(-w_4^{\mathrm{AA}} - \frac{1}{2}\left(\sqrt{3}-i\right) w_6^{\mathrm{AA}}\right) s_y + \left(-w_5^{\mathrm{AA}} - i w_3^{\mathrm{AA}}\right) s_z\right]_{s_1 s_2} \\
\left[T^{\mathrm{AA}}_{\mathbf{q}_{+1},\mathbf{b}_{M_1}-\mathbf{b}_{M_2}+\mathbf{q}_{+1}}\right]_{s_1(+);s_2(+)} &= \left[s_0 \left(-i w_1'^{\mathrm{AA}} + w_4'^{\mathrm{AA}} - i w_7'^{\mathrm{AA}} + w_{10}'^{\mathrm{AA}}\right)\right]_{s_1 s_2}
\end{aligned}$$

$$\begin{aligned}
\left[T^{\mathrm{AA}}_{\mathbf{q}_{-1},\mathbf{b}_{M_1}+\mathbf{q}_{-1}}\right]_{s_1(-);s_2(-)} &= \left[s_0\left(w_5'^{\mathrm{AA}} - iw_2'^{\mathrm{AA}}\right)\right]_{s_1 s_2} \\
\left[T^{\mathrm{AA}}_{\mathbf{q}_{+1},\mathbf{b}_{M_1}+\mathbf{q}_{+1}}\right]_{s_1(+);s_2(+)} &= \left[s_0\left(w_5'^{\mathrm{AA}} - iw_2'^{\mathrm{AA}}\right)\right]_{s_1 s_2},
\end{aligned} \tag{S5.94}$$

$$\begin{aligned}
\left[T^{\mathrm{AB}}_{\mathbf{q}_{-1},\mathbf{q}_{+1}-\mathbf{b}_{M_2}}\right]_{s_1(-);s_2(+)} &= \left[w_2^{\mathrm{AB}} s_0 - iw_1^{\mathrm{AB}} s_x + w_3^{\mathrm{AB}} s_y - iw_4^{\mathrm{AB}} s_z\right]_{s_1 s_2} \\
\left[T^{\mathrm{AB}}_{\mathbf{q}_{-1},\mathbf{q}_{+1}}\right]_{s_1(-);s_2(+)} &= \left[s_0\left(w_4'^{\mathrm{AB}} + iw_9'^{\mathrm{AB}}\right) + s_x\left(-w_{11}'^{\mathrm{AB}} - iw_1'^{\mathrm{AB}}\right) + s_z\left(w_5'^{\mathrm{AB}} - iw_8'^{\mathrm{AB}}\right)\right]_{s_1 s_2} \\
\left[T^{\mathrm{AB}}_{\mathbf{q}_{-1},\mathbf{b}_{M_2}+\mathbf{q}_{-1}}\right]_{s_1(-);s_2(-)} &= \left[s_0\left(iw_2'^{\mathrm{AB}} + w_6'^{\mathrm{AB}} + iw_{10}'^{\mathrm{AB}} + w_{12}'^{\mathrm{AB}}\right)\right]_{s_1 s_2} \\
\left[T^{\mathrm{AB}}_{\mathbf{q}_{+1},\mathbf{b}_{M_2}+\mathbf{q}_{+1}}\right]_{s_1(+);s_2(+)} &= \left[s_0\left(-iw_2'^{\mathrm{AB}} + w_6'^{\mathrm{AB}} + iw_{10}'^{\mathrm{AB}} - w_{12}'^{\mathrm{AB}}\right)\right]_{s_1 s_2} \\
\left[T^{\mathrm{AB}}_{\mathbf{q}_{-1},\mathbf{b}_{M_1}-2\mathbf{b}_{M_2}+\mathbf{q}_{+1}}\right]_{s_1(-);s_2(+)} &= \left[s_0\left(w_4'^{\mathrm{AB}} - iw_9'^{\mathrm{AB}}\right) + s_x\left(w_{11}'^{\mathrm{AB}} - iw_1'^{\mathrm{AB}}\right) + s_z\left(-w_5'^{\mathrm{AB}} - iw_8'^{\mathrm{AB}}\right)\right]_{s_1 s_2} \\
\left[T^{\mathrm{AB}}_{\mathbf{q}_{-1},\mathbf{b}_{M_1}-\mathbf{b}_{M_2}+\mathbf{q}_{-1}}\right]_{s_1(-);s_2(-)} &= \left[s_0\left(iw_2'^{\mathrm{AB}} + w_6'^{\mathrm{AB}} - iw_{10}'^{\mathrm{AB}} - w_{12}'^{\mathrm{AB}}\right)\right]_{s_1 s_2} \\
\left[T^{\mathrm{AB}}_{\mathbf{q}_{-1},\mathbf{b}_{M_1}-\mathbf{b}_{M_2}+\mathbf{q}_{+1}}\right]_{s_1(-);s_2(+)} &= \left[w_2^{\mathrm{AB}} s_0 - iw_1^{\mathrm{AB}} s_x - w_3^{\mathrm{AB}} s_y - iw_4^{\mathrm{AB}} s_z\right]_{s_1 s_2} \\
\left[T^{\mathrm{AB}}_{\mathbf{q}_{+1},\mathbf{b}_{M_1}-\mathbf{b}_{M_2}+\mathbf{q}_{+1}}\right]_{s_1(+);s_2(+)} &= \left[s_0\left(-iw_2'^{\mathrm{AB}} + w_6'^{\mathrm{AB}} - iw_{10}'^{\mathrm{AB}} + w_{12}'^{\mathrm{AB}}\right)\right]_{s_1 s_2} \\
\left[T^{\mathrm{AB}}_{\mathbf{q}_{-1},\mathbf{b}_{M_1}+\mathbf{q}_{-1}}\right]_{s_1(-);s_2(-)} &= \left[s_0\left(w_7'^{\mathrm{AB}} + iw_3'^{\mathrm{AB}}\right)\right]_{s_1 s_2} \\
\left[T^{\mathrm{AB}}_{\mathbf{q}_{+1},\mathbf{b}_{M_1}+\mathbf{q}_{+1}}\right]_{s_1(+);s_2(+)} &= \left[s_0\left(w_7'^{\mathrm{AB}} - iw_3'^{\mathrm{AB}}\right)\right]_{s_1 s_2}.
\end{aligned} \tag{S5.95}$$

In Eq. (S5.94) we have only listed half of the nonzero components of $T_{\mathbf{Q},\mathbf{Q}'}$ for valley $\eta = 0$. The other nonzero components in valley $\eta = 0$ can be obtained with the Hermiticity condition from Eq. (S5.89). The moiré potential in the other valleys can be obtained with the C_{3z} symmetry using Eq. (S5.91). The additional terms are within the first moiré harmonic approximation, but go beyond the two-center first monolayer harmonic approximation.

B. General form of the moiré potential restricted by the approximate $\theta = 0$ symmetries of the heterostructure

Having obtained the most general form of the moiré potential for an arbitrary non-zero angle, we now further constrain its form in the limit of vanishing twist angle $\theta \to 0$, using the exact symmetries of the untwisted configuration. In doing so, we will make the "local-stacking approximation" [2]. We will make this approximation mathematically rigorous in Section V B 2. Briefly, for small (but nonzero) twist angle θ, we can *locally* understand the heterostructure as being comprised of two monolayers which are untwisted, but are otherwise displaced by $\Delta\mathbf{R}$, where $\Delta\mathbf{R}$ is determined by the twist angle and the position within the heterostructure. We will first study the untwisted bilayer Hamiltonians $\mathcal{H}(\Delta\mathbf{R})$ for different displacements $\Delta\mathbf{R}$, and characterize their symmetries. Then, we will illustrate how the moiré continuum Hamiltonian can be obtained from $\mathcal{H}(\Delta\mathbf{R})$, which will finally allow us to further constrain the moiré potential in the limit of vanishing twist angle.

1. *Hamiltonian for the untwisted configuration*

We begin by considering a family of Hamiltonians $\mathcal{H}(\Delta\mathbf{R})$ describing an untwisted bilayer arrangement, where the top (bottom) layer is displaced by $+\frac{1}{2}\Delta\mathbf{R}$ $(-\frac{1}{2}\Delta\mathbf{R})$. In the untwisted AA-stacked configuration, the two layers are stacked directly on top of one another for $\Delta\mathbf{R} = \mathbf{0}$, while in the AB-stacked configuration, the bottom layer is rotated around the origin by 180° relative to the top layer in the $\Delta\mathbf{R} = \mathbf{0}$ case. We let $\hat{a}^\dagger_{\mathbf{R},l,s}$ denote the creation operator corresponding to an electron in layer l located at $\mathbf{r}^{\Delta\mathbf{R}}_{l,\mathbf{R}} = \mathbf{R} + \frac{l}{2}\Delta\mathbf{R}$ $(\mathbf{r}^{\Delta\mathbf{R}}_{l,\mathbf{R}} = l\mathbf{R} + \frac{l}{2}\Delta\mathbf{R})$ in the AA- (AB-)stacking untwisted configuration. The Hamiltonian $\mathcal{H}(\Delta\mathbf{R})$ can be written generically as

$$\mathcal{H}(\Delta\mathbf{R}) = \sum_l \mathcal{H}^{\mathrm{sl}}_l + \sum_{\substack{\mathbf{R}_1,l_1,s_1\\ \mathbf{R}_2,l_2,s_2}} S_{s_1 l_1; s_2 l_2}\left(\Delta\mathbf{R}, \mathbf{r}^{\Delta\mathbf{R}}_{l_2,\mathbf{R}_2} - \mathbf{r}^{\Delta\mathbf{R}}_{l_1,\mathbf{R}_1}\right) \hat{a}^\dagger_{\mathbf{R}_1,s_1,l_1} \hat{a}_{\mathbf{R}_2,s_2,l_2}, \tag{S5.96}$$

where the first term is the single-particle Hamiltonian of a single layer given by Eq. (S2.14)

$$\mathcal{H}_l^{\mathrm{sl}} = \sum_{\mathbf{k}} \left[h\left(\mathbf{k}\right)\right]_{s_1 s_2} \hat{a}^\dagger_{\mathbf{k},s_1,l} \hat{a}_{\mathbf{k},s_2,l}. \tag{S5.97}$$

The second term of Eq. (S5.96) is the additional single-particle contribution arising from stacking the two layers. It contains both interlayer terms (arising from tunneling), as well as intralayer terms, arising from *e.g.* assisted hopping through the adjacent layer. Because the Hamiltonian $\mathcal{H}\left(\Delta\mathbf{R}\right)$ retains the discrete translation symmetry of the single layer material, for a given interlayer displacement $\Delta\mathbf{R}$, the amplitude of the second term $S_{s_1 l_1; s_2 l_2}\left(\Delta\mathbf{R}, \mathbf{r}^{\Delta\mathbf{R}}_{l_2,\mathbf{R}_2} - \mathbf{r}^{\Delta\mathbf{R}}_{l_1,\mathbf{R}_1}\right)$ only depends on the distance $\mathbf{r}^{\Delta\mathbf{R}}_{l_2,\mathbf{R}_2} - \mathbf{r}^{\Delta\mathbf{R}}_{l_1,\mathbf{R}_1}$, but not on the positions $\mathbf{r}^{\Delta\mathbf{R}}_{l_2,\mathbf{R}_2}$ and $\mathbf{r}^{\Delta\mathbf{R}}_{l_1,\mathbf{R}_1}$ separately. We will also assume that the perturbation stemming from the stacking the two layers only couples fermions that are a few lattice sites apart, which means that $S_{s_1 l_1; s_2 l_2}\left(\Delta\mathbf{R}, \mathbf{r}\right) \approx 0$ for $\mathbf{r} \gg |\mathbf{a}_1|$. We will later use this condition in Section V B 2 where we derive the continuum model for the twisted moiré heterostructure.

In the untwisted bilayer arrangement, the tunneling between two fermions located at the same distance $\mathbf{r}$ is the same irrespective of whether the layers are shifted by $\Delta\mathbf{R}$ or by $\Delta\mathbf{R} + \mathbf{a}_i$ (for $1 \leq i \leq 2$), *i.e.*

$$S_{s_1 l_1; s_2 l_2}\left(\Delta\mathbf{R} + \mathbf{a}_i, \mathbf{r}\right) = S_{s_1 l_1; s_2 l_2}\left(\Delta\mathbf{R}, \mathbf{r}\right), \quad \text{for} \quad 1 \leq i \leq 2. \tag{S5.98}$$

Consider now a symmetry operation g of the zero-displacement ($\Delta\mathbf{R} = \mathbf{0}$) untwisted configuration, whose action on the lattice fermions is given by

$$g \hat{a}^\dagger_{\mathbf{R},s_1,l} g^{-1} = \sum_{s_2} \left[D^{\mathrm{sl}}(g)\right]_{s_2 s_1} \hat{a}^\dagger_{g\mathbf{R},s_2,(\epsilon_g l)}, \tag{S5.99}$$

where similarly to Eq. (S4.40), $\epsilon_g = +1$ ($\epsilon_g = -1$) if g exchanges the layers, and $D^{\mathrm{sl}}(g)$ has been introduced in Eq. (S2.5). We also comment that certain single-layer symmetry operations, such as C_{2x} or C_{2y}, also flip the layer index, which justify the presence of the ϵ_g factor. The action of the symmetry g on the untwisted, but displaced bilayer Hamiltonian is given by

$$g\mathcal{H}\left(\Delta\mathbf{R}\right) g^{-1} = \mathcal{H}\left(\epsilon_g g \Delta\mathbf{R}\right). \tag{S5.100}$$

We also provide an intuitive understanding of the above equation. By acting with a symmetry operation g, we obtain a new Hamiltonian, $g\mathcal{H}\left(\Delta\mathbf{R}\right) g^{-1}$. Considering *e.g.* the AA-stacking configuration, the symmetry operation g changes the position of a fermion operator $\hat{a}^\dagger_{\mathbf{R},s,l}$ from $\mathbf{r}^{\Delta\mathbf{R}}_{l,\mathbf{R}} = \mathbf{R} + \frac{l}{2}\Delta\mathbf{R}$ within layer l to $g\mathbf{R} + \frac{l}{2} g\Delta\mathbf{R} = \mathbf{r}^{\epsilon_g g \Delta\mathbf{R}}_{\epsilon_g l, g\mathbf{R}}$ within layer $\epsilon_g l$. Effectively, g changes the interlayer displacement from $\Delta\mathbf{R}$ to $\epsilon_g g \Delta\mathbf{R}$, thereby showing that the two Hamiltonians $g\mathcal{H}\left(\Delta\mathbf{R}\right) g^{-1}$ and $\mathcal{H}\left(\epsilon_g g\Delta\mathbf{R}\right)$ are equivalent. Since g is a symmetry of the undisplaced and untwisted configuration, we have $[g, \sum_l \mathcal{H}_l] = 0$, where $\mathcal{H}_l$ was defined in Eq. (S5.97), which means that Eqs. (S5.96) and (S5.100) simplify to

$$\begin{aligned}
&\sum_{\substack{\mathbf{R}_1, l_1, s_1, s'_1 \\ \mathbf{R}_2, l_2, s_2, s'_2}} S^{(*)}_{s_1 l_1; s_2 l_2}\left(\Delta\mathbf{R}, \mathbf{r}^{\Delta\mathbf{R}}_{l_2,\mathbf{R}_2} - \mathbf{r}^{\Delta\mathbf{R}}_{l_1,\mathbf{R}_1}\right) \left[D^{\mathrm{sl}}(g)\right]_{s'_1 s_1} \left[D^{\mathrm{sl}}(g)\right]^*_{s'_2 s_2} \hat{a}^\dagger_{g\mathbf{R}_1, s'_1, \epsilon_g l_1} \hat{a}_{g\mathbf{R}_2, s'_2, \epsilon_g l_2} \\
&= \sum_{\substack{\mathbf{R}_1, l_1, s_1 \\ \mathbf{R}_2, l_2, s_2}} S_{s_1 l_1; s_2 l_2}\left(\epsilon_g g\Delta\mathbf{R}, \mathbf{r}^{\epsilon_g g\Delta\mathbf{R}}_{l_2,\mathbf{R}_2} - \mathbf{r}^{\epsilon_g g\Delta\mathbf{R}}_{l_1,\mathbf{R}_1}\right) \hat{a}^\dagger_{\mathbf{R}_1, s_1, l_1} \hat{a}_{\mathbf{R}_2, s_2, l_2}.
\end{aligned} \tag{S5.101}$$

In Eq. (S5.101), $^{(*)}$ indicates that a complex conjugation should be performed if g is antiunitary. Equivalently, Eq. (S5.101) implies that

$$\begin{aligned}
\sum_{s_1, s_2} \left[D^{\mathrm{sl}}(g)\right]_{s'_1 s_1} S^{(*)}_{s_1 l_1; s_2 l_2}\left(\Delta\mathbf{R}, \mathbf{r}^{\Delta\mathbf{R}}_{l_2,\mathbf{R}_2} - \mathbf{r}^{\Delta\mathbf{R}}_{l_1,\mathbf{R}_1}\right) \left[D^{\mathrm{sl}}(g)\right]^*_{s'_2 s_2} &= S_{s'_1 \epsilon_g l_1; s'_2 \epsilon_g l_2}\left(\epsilon_g g\Delta\mathbf{R}, g\left(\mathbf{r}^{\Delta\mathbf{R}}_{l_2,\mathbf{R}_2} - \mathbf{r}^{\Delta\mathbf{R}}_{l_1,\mathbf{R}_1}\right)\right) \\
\sum_{s_1, s_2} \left[D^{\mathrm{sl}}(g)\right]_{s'_1 s_1} S^{(*)}_{s_1 l_1; s_2 l_2}\left(\Delta\mathbf{R}, \mathbf{r}\right) \left[D^{\mathrm{sl}}(g)\right]^*_{s'_2 s_2} &= S_{s'_1 \epsilon_g l_1; s'_2 \epsilon_g l_2}\left(\epsilon_g g\Delta\mathbf{R}, g\mathbf{r}\right).
\end{aligned} \tag{S5.102}$$

Finally, we note that as a result of Hermiticity, we must have that

$$S_{s_1 l_1; s_2 l_2}\left(\Delta\mathbf{R}, \mathbf{r}\right) = S^*_{s_2 l_2; s_1 l_1}\left(\Delta\mathbf{R}, -\mathbf{r}\right). \tag{S5.103}$$

Because $S_{s_1 l_1; s_2 l_2}\left(\Delta\mathbf{R}, \mathbf{r}\right)$ is periodic in $\Delta\mathbf{R}$, as implied by Eq. (S5.98), for a given $\mathbf{r}$, it can be expanded as a Fourier series

$$S_{s_1 l_1; s_2 l_2}\left(\Delta\mathbf{R}, \mathbf{r}\right) = \sum_{\mathbf{g}} S_{s_1 l_1; s_2 l_2}\left(\mathbf{g}, \mathbf{r}\right) e^{-i\mathbf{g}\cdot\Delta\mathbf{R}}, \tag{S5.104}$$

where $\mathbf{g}$ runs over the reciprocal lattice vectors of the single-layer material. Additionally, for a given $\Delta\mathbf{R}$ $S_{s_1l_1;s_2l_2}(\Delta\mathbf{R},\mathbf{r})$ is only defined for discrete values of $\mathbf{r} = \mathbf{r}^{\Delta\mathbf{R}}_{l_2,\mathbf{R}_2} - \mathbf{r}^{\Delta\mathbf{R}}_{l_1,\mathbf{R}_1}$. As such, $S_{s_1l_1;s_2l_2}(\Delta\mathbf{R},\mathbf{r})$ also accepts a momentum space representation in the second variable

$$S_{s_1l_1;s_2l_2}(\Delta\mathbf{R},\mathbf{r}) = \frac{1}{N}\sum_{\mathbf{k}} S_{s_1l_1;s_2l_2}(\Delta\mathbf{R},\mathbf{k})\, e^{-i\mathbf{k}\cdot\mathbf{r}}, \tag{S5.105}$$

$$S_{s_1l_1;s_2l_2}(\Delta\mathbf{R},\mathbf{k}) = \sum_{\mathbf{R}_1} S_{s_1l_1;s_2l_2}\left(\Delta\mathbf{R}, \mathbf{r}^{\Delta\mathbf{R}}_{l_2,\mathbf{R}_2} - \mathbf{r}^{\Delta\mathbf{R}}_{l_1,\mathbf{R}_1}\right) e^{i\mathbf{k}\cdot\left(\mathbf{r}^{\Delta\mathbf{R}}_{l_2,\mathbf{R}_2} - \mathbf{r}^{\Delta\mathbf{R}}_{l_1,\mathbf{R}_1}\right)}, \tag{S5.106}$$

with $\mathbf{k}$ belonging to the first BZ of the single-layer material. Eq. (S5.106) can be extended outside the first Brillouin zone by noting that

$$\begin{aligned}
S_{s_1l_1;s_2l_2}(\Delta\mathbf{R},\mathbf{k}+\mathbf{g}) &= \sum_{\mathbf{R}_1} S_{s_1l_1;s_2l_2}\left(\Delta\mathbf{R}, \mathbf{r}^{\Delta\mathbf{R}}_{l_2,\mathbf{R}_2} - \mathbf{r}^{\Delta\mathbf{R}}_{l_1,\mathbf{R}_1}\right) e^{i(\mathbf{k}+\mathbf{g})\cdot\left(\mathbf{r}^{\Delta\mathbf{R}}_{l_2,\mathbf{R}_2} - \mathbf{r}^{\Delta\mathbf{R}}_{l_1,\mathbf{R}_1}\right)} \\
&= \sum_{\mathbf{R}_1} S_{s_1l_1;s_2l_2}\left(\Delta\mathbf{R}, \mathbf{r}^{\Delta\mathbf{R}}_{l_2,\mathbf{R}_2} - \mathbf{r}^{\Delta\mathbf{R}}_{l_1,\mathbf{R}_1}\right) e^{i\mathbf{k}\cdot\left(\mathbf{r}^{\Delta\mathbf{R}}_{l_2,\mathbf{R}_2} - \mathbf{r}^{\Delta\mathbf{R}}_{l_1,\mathbf{R}_1}\right)} e^{i\mathbf{g}\cdot\Delta\mathbf{R}\frac{l_2-l_1}{2}} \\
&= S_{s_1l_1;s_2l_2}(\Delta\mathbf{R},\mathbf{k})\, e^{i\mathbf{g}\cdot\Delta\mathbf{R}\frac{l_2-l_1}{2}},
\end{aligned} \tag{S5.107}$$

where the phase is just the "embedding" factor of a typical tight-binding Hamiltonian. Finally, one can Fourier-transform over both variables and obtain

$$S_{s_1l_1;s_2l_2}(\Delta\mathbf{R},\mathbf{r}) = \frac{1}{N}\sum_{\mathbf{g},\mathbf{k}} S_{s_1l_1;s_2l_2}(\mathbf{g},\mathbf{k})\, e^{-i\mathbf{g}\cdot\Delta\mathbf{R}} e^{-i\mathbf{k}\cdot\mathbf{r}}, \tag{S5.108}$$

which, as a result of Eq. (S5.107), obeys

$$S_{s_1l_1;s_2l_2}(\mathbf{g},\mathbf{k}+\mathbf{g}') = S_{s_1l_1;s_2l_2}\left(\mathbf{g} + \frac{l_2-l_1}{2}\mathbf{g}', \mathbf{k}\right). \tag{S5.109}$$

2. Hamiltonian for the twisted configuration

We are now in a position of obtaining the Hamiltonian for the twisted bilayer arrangement. As in Section IV A, in the twisted configuration, the fermion $\hat{c}^\dagger_{\mathbf{R},s,l}$ is located at $\mathbf{r}_{l,\mathbf{R}} = R_{\theta,l}\mathbf{R}$ in the AA-stacked case or at $\mathbf{r}_{l,\mathbf{R}} = lR_{\theta,l}\mathbf{R}$ in the AB-stacked case. Consider now two fermions $\hat{c}^\dagger_{\mathbf{R}_1,s_1,l_1}$ and $\hat{c}^\dagger_{\mathbf{R}_2,s_2,l_2}$. At the single-particle level, these two fermions will only be coupled provided that $|\mathbf{r}_{l_1,\mathbf{R}_1} - \mathbf{r}_{l_2,\mathbf{R}_2}|$ is not larger than a few lattice constants of the single-layer material. As explained at the beginning of Section IV C, at a position $\mathbf{r}$, the two monolayer lattices are displaced by $\delta\mathbf{R}(\mathbf{r})$, where the effective local displacement was obtained in Eq. (S4.54). Because for any two fermions $\hat{c}^\dagger_{\mathbf{R}_1,s_1,l_1}$ and $\hat{c}^\dagger_{\mathbf{R}_2,s_2,l_2}$ coupled at the single-particle level, $|\mathbf{r}_{l_1,\mathbf{R}_1} - \mathbf{r}_{l_2,\mathbf{R}_2}| \sim |\mathbf{a}_1| \ll |\mathbf{a}_{M_1}|$, we will have $\delta\mathbf{R}(\mathbf{r}_{l_1,\mathbf{R}_1}) \approx \delta\mathbf{R}(\mathbf{r}_{l_2,\mathbf{R}_2}) \approx \delta\mathbf{R}\left(\frac{\mathbf{r}_{l_1,\mathbf{R}_1}+\mathbf{r}_{l_2,\mathbf{R}_2}}{2}\right)$. As such, we can take the single-particle term coupling these two fermions to be [2]

$$S_{s_1l_1;s_2l_2}\left(\delta\mathbf{R}\left(\frac{\mathbf{r}_{l_1,\mathbf{R}_1}+\mathbf{r}_{l_2,\mathbf{R}_2}}{2}\right), \mathbf{r}_{l_2,\mathbf{R}_2} - \mathbf{r}_{l_1,\mathbf{R}_1}\right) \hat{a}^\dagger_{\mathbf{R}_1,s_1,l_1}\hat{a}_{\mathbf{R}_2,s_2,l_2}, \tag{S5.110}$$

which allows us to write an expression for the Hamiltonian of the twisted bilayer heterostructure

$$\begin{aligned}
\mathcal{H} &= \sum_l \mathcal{H}^{\text{sl}}_l + \mathcal{H}^{\text{moiré}}, \quad \text{with} \\
\mathcal{H}^{\text{moiré}} &= \sum_{\substack{\mathbf{R}_1,l_1,s_1 \\ \mathbf{R}_2,l_2,s_2}} S_{s_1l_1;s_2l_2}\left(\delta\mathbf{R}\left(\frac{\mathbf{r}_{l_1,\mathbf{R}_1}+\mathbf{r}_{l_2,\mathbf{R}_2}}{2}\right), \mathbf{r}_{l_2,\mathbf{R}_2} - \mathbf{r}_{l_1,\mathbf{R}_1}\right) \hat{a}^\dagger_{\mathbf{R}_1,s_1,l_1}\hat{a}_{\mathbf{R}_2,s_2,l_2}.
\end{aligned} \tag{S5.111}$$

We now take a low-energy approximation for the lattice fermions, using the notation introduced in Eq. (S4.73)

$$\hat{a}^\dagger_{\mathbf{R},s,l} \approx \sqrt{\Omega_0}\sum_\eta \int \mathrm{d}^2 r\, \delta(\mathbf{r}_{l,\mathbf{R}} - \mathbf{r})\, \hat{\psi}^\dagger_{\eta,s,l}(\mathbf{r})\, e^{-iC^\eta_{3z}\mathbf{K}^l_M\cdot\mathbf{r}}, \tag{S5.112}$$

with the aid of which the moiré potential part of the Hamiltonian becomes

$$\mathcal{H}^{\text{moiré}} = \Omega_0 \sum_{\substack{\mathbf{R}_1, l_1, s_1, \eta_1 \\ \mathbf{R}_2, l_2, s_2, \eta_2}} \int \mathrm{d}^2 r_1 \, \mathrm{d}^2 r_2 \, S_{s_1 l_1; s_2 l_2} \left(\delta \mathbf{R} \left(\frac{\mathbf{r}_1 + \mathbf{r}_2}{2} \right), \mathbf{r}_2 - \mathbf{r}_1 \right) \delta \left(\mathbf{r}_{l_1, \mathbf{R}_1} - \mathbf{r}_1 \right) \delta \left(\mathbf{r}_{l_2, \mathbf{R}_2} - \mathbf{r}_2 \right)$$
$$\times \hat{\psi}^\dagger_{\eta_1, s_1, l_1} \left(\mathbf{r}_1 \right) \hat{\psi}_{\eta_2, s_2, l_2} \left(\mathbf{r}_2 \right) e^{-i C_{3z}^{\eta_1} \mathbf{K}_M^{l_1} \cdot \mathbf{r}_1} e^{i C_{3z}^{\eta_2} \mathbf{K}_M^{l_2} \cdot \mathbf{r}_2}. \tag{S5.113}$$

We then employ the Poisson summation formula

$$\sum_{\mathbf{R}} \delta \left(\mathbf{r}_{l, \mathbf{R}} - \mathbf{r} \right) = \frac{1}{\Omega_0} \sum_{\mathbf{g}} e^{i \mathcal{R}_{\theta, l} \mathbf{g} \cdot \mathbf{r}}, \tag{S5.114}$$

and simultaneously change the integration variables to the center of mass $\mathbf{x} = \frac{\mathbf{r}_1 + \mathbf{r}_2}{2}$ and displacement $\mathbf{y} = \mathbf{r}_2 - \mathbf{r}_1$ coordinates in Eq. (S5.113)

$$\mathcal{H}^{\text{moiré}} = \frac{1}{\Omega_0} \sum_{\substack{\mathbf{g}_1, l_1, s_1, \eta_1 \\ \mathbf{g}_2, l_2, s_2, \eta_2}} \int \mathrm{d}^2 x \, \mathrm{d}^2 y \, S_{s_1 l_1; s_2 l_2} \left(\delta \mathbf{R} \left(\mathbf{x} \right), \mathbf{y} \right) e^{-i \mathcal{R}_{\theta, l_1} \left(\mathbf{g}_1 + C_{3z}^{\eta_1} \mathbf{K}_M \right) \cdot \left(\mathbf{x} - \frac{\mathbf{y}}{2} \right)} e^{i \mathcal{R}_{\theta, l_2} \left(\mathbf{g}_2 + C_{3z}^{\eta_2} \mathbf{K}_M \right) \cdot \left(\mathbf{x} + \frac{\mathbf{y}}{2} \right)}$$
$$\times \hat{\psi}^\dagger_{\eta_1, s_1, l_1} \left(\mathbf{x} - \frac{\mathbf{y}}{2} \right) \hat{\psi}_{\eta_2, s_2, l_2} \left(\mathbf{x} + \frac{\mathbf{y}}{2} \right)$$
$$\approx \frac{1}{\Omega_0} \sum_{\substack{\mathbf{g}_1, l_1, s_1, \eta_1 \\ \mathbf{g}_2, l_2, s_2, \eta_2}} \int \mathrm{d}^2 x \, \mathrm{d}^2 y \, S_{s_1 l_1; s_2 l_2} \left(\delta \mathbf{R} \left(\mathbf{x} \right), \mathbf{y} \right) e^{-i \mathcal{R}_{\theta, l_1} \left(\mathbf{g}_1 + C_{3z}^{\eta_1} \mathbf{K}_M \right) \cdot \left(\mathbf{x} - \frac{\mathbf{y}}{2} \right)} e^{i \mathcal{R}_{\theta, l_2} \left(\mathbf{g}_2 + C_{3z}^{\eta_2} \mathbf{K}_M \right) \cdot \left(\mathbf{x} + \frac{\mathbf{y}}{2} \right)}$$
$$\times \hat{\psi}^\dagger_{\eta_1, s_1, l_1} \left(\mathbf{x} \right) \hat{\psi}_{\eta_2, s_2, l_2} \left(\mathbf{x} \right)$$
$$\approx \frac{1}{\Omega_0} \sum_{\substack{\mathbf{g}, \eta \\ l_1, s_1, l_2, s_2}} \int \mathrm{d}^2 x \, \mathrm{d}^2 y \, S_{s_1 l_1; s_2 l_2} \left(\delta \mathbf{R} \left(\mathbf{x} \right), \mathbf{y} \right) e^{-i \mathcal{R}_{\theta, l_1} \left(\mathbf{g} + C_{3z}^{\eta} \mathbf{K}_M \right) \cdot \left(\mathbf{x} - \frac{\mathbf{y}}{2} \right)} e^{i \mathcal{R}_{\theta, l_2} \left(\mathbf{g} + C_{3z}^{\eta} \mathbf{K}_M \right) \cdot \left(\mathbf{x} + \frac{\mathbf{y}}{2} \right)}$$
$$\times \hat{\psi}^\dagger_{\eta, s_1, l_1} \left(\mathbf{x} \right) \hat{\psi}_{\eta, s_2, l_2} \left(\mathbf{x} \right)$$
$$= \frac{1}{\Omega_0} \sum_{\substack{\mathbf{g}, \eta \\ l_1, s_1, l_2, s_2}} \int \mathrm{d}^2 x \, \mathrm{d}^2 y \, S_{s_1 l_1; s_2 l_2} \left(\delta \mathbf{R} \left(\mathbf{x} \right), \mathbf{y} \right) e^{i \left(\mathcal{R}_{\theta, l_1} + \mathcal{R}_{\theta, l_2} \right) \left(\mathbf{g} + C_{3z}^{\eta} \mathbf{K}_M \right) \cdot \frac{\mathbf{y}}{2}}$$
$$\times \hat{\psi}^\dagger_{\eta, s_1, l_1} \left(\mathbf{x} \right) \hat{\psi}_{\eta, s_2, l_2} \left(\mathbf{x} \right) e^{-i \mathcal{R}_{\theta, l_1} \left(\mathbf{g} + C_{3z}^{\eta} \mathbf{K}_M \right) \cdot \mathbf{x} + i \mathcal{R}_{\theta, l_2} \left(\mathbf{g} + C_{3z}^{\eta} \mathbf{K}_M \right) \cdot \mathbf{x}}$$
$$\approx \frac{1}{\Omega_0} \sum_{\substack{\mathbf{g}, \eta \\ l_1, s_1, l_2, s_2}} \int \mathrm{d}^2 x \, \mathrm{d}^2 y \, S_{s_1 l_1; s_2 l_2} \left(\delta \mathbf{R} \left(\mathbf{x} \right), \mathbf{y} \right) e^{i \left(\mathbf{g} + C_{3z}^{\eta} \mathbf{K}_M \right) \cdot \mathbf{y}}$$
$$\times \hat{\psi}^\dagger_{\eta, s_1, l_1} \left(\mathbf{x} \right) \hat{\psi}_{\eta, s_2, l_2} \left(\mathbf{x} \right) e^{-i \mathcal{R}_{\theta, l_1} \left(\mathbf{g} + C_{3z}^{\eta} \mathbf{K}_M \right) \cdot \mathbf{x} + i \mathcal{R}_{\theta, l_2} \left(\mathbf{g} + C_{3z}^{\eta} \mathbf{K}_M \right) \cdot \mathbf{x}} \tag{S5.115}$$

In performing the first approximation of Eq. (S5.115) (*i.e.* from the first two lines to the following two), we have used the fact that $S_{s_1 l_1; s_2 l_2} \left(\delta \mathbf{R} \left(\mathbf{x} \right), \mathbf{y} \right)$ is only non-zero for $|\mathbf{y}| \sim |\mathbf{a}_1|$, which allows us to assume $|\mathbf{y}| \lesssim |\mathbf{a}_1|$ under the integral. As we are interested in the long-wavelength description of the moiré system, we have expanded the field operators for small $|\mathbf{y}|$ and neglected all the terms containing derivatives of the fermionic field (since, by assumption, $\mathbf{y} \cdot \boldsymbol{\nabla} \hat{\psi}^\dagger_{\eta, s, l} \left(\mathbf{x} \right) \sim \frac{|\mathbf{a}_1|}{|\mathbf{a}_{M_1}|} \ll 1$), or equivalently speaking, we have approximated $\hat{\psi}^\dagger_{\eta, s, l} (\mathbf{x} \pm \mathbf{y}/2) \approx \hat{\psi}^\dagger_{\eta, s, l} (\mathbf{x})$. Additionally, from the second to the third line, we have also employed the fact the field operators are "slow" operators, and do not "vary" over the single layer lattice scale. As such, only those terms whose complex exponential factors do not significantly oscillate at the single-layer graphene scale (corresponding to $\mathbf{g}_1 = \mathbf{g}_2 = \mathbf{g}$ and $\eta_1 = \eta_2 = \eta$) will survive the integration over $\mathbf{x}$. Finally, in the last row, we have again used the fact that $S_{s_1 l_1; s_2 l_2} \left(\delta \mathbf{R} \left(\mathbf{x} \right), \mathbf{y} \right)$ is only non-zero for $|\mathbf{y}| \sim |\mathbf{a}_1| \ll |\mathbf{a}_{M_1}|$, for which we can approximate

$$e^{i \mathcal{R}_{\theta, \pm} \left(\mathbf{g} + C_{3z}^{\eta} \mathbf{K}_M \right) \cdot \frac{\mathbf{y}}{2}} \approx e^{i \left(\mathbf{g} + C_{3z}^{\eta} \mathbf{K}_M \right) \cdot \frac{\mathbf{y}}{2}}. \tag{S5.116}$$

Plugging the Fourier representation from Eq. (S5.108) into Eq. (S5.115), we obtain

$$\mathcal{H}^{\text{moiré}} = \frac{1}{N \Omega_0} \sum_{\substack{\mathbf{g}, \mathbf{g}', \mathbf{k}, \eta \\ l_1, s_1, l_2, s_2}} S_{s_1 l_1; s_2 l_2} \left(\mathbf{g}', \mathbf{k} \right) \int \mathrm{d}^2 y \, e^{-i \mathbf{k} \cdot \mathbf{y}} e^{i \left(\mathbf{g} + C_{3z}^{\eta} \mathbf{K}_M \right) \cdot \mathbf{y}}$$

$$
\begin{aligned}
&\times \int \mathrm{d}^2 x\, \hat{\psi}^\dagger_{\eta,s_1,l_1}(\mathbf{x})\, \hat{\psi}_{\eta,s_2,l_2}(\mathbf{x})\, e^{-i\mathcal{R}_{\theta,l_1}\left(\mathbf{g}+C^\eta_{3z}\mathbf{K}_M\right)\cdot\mathbf{x}} e^{i\mathcal{R}_{\theta,l_2}\left(\mathbf{g}+C^\eta_{3z}\mathbf{K}_M\right)\cdot\mathbf{x}} e^{-i\mathbf{g}'\cdot\delta\mathbf{R}(\mathbf{x})} \\
= &\sum_{\substack{\mathbf{g},\eta \\ l_1,s_1,l_2,s_2}} S_{s_1 l_1; s_2 l_2}\left(\mathbf{g}, C^\eta_{3z}\mathbf{K}_M\right) \\
&\times \int \mathrm{d}^2 x\, \hat{\psi}^\dagger_{\eta,s_1,l_1}(\mathbf{x})\, \hat{\psi}_{\eta,s_2,l_2}(\mathbf{x})\, e^{i\left(\mathcal{R}_{\theta,l_2}-\mathcal{R}_{\theta,l_1}\right)C^\eta_{3z}\mathbf{K}_M\cdot\mathbf{x}} e^{-i\mathbf{g}\cdot\delta\mathbf{R}(\mathbf{x})} \\
= &\sum_{\substack{\mathbf{g},\eta \\ l_1,s_1,l_2,s_2}} \int \mathrm{d}^2 r\, S_{s_1 l_1; s_2 l_2}\left(\mathbf{g}, C^\eta_{3z}\mathbf{K}_M\right) \hat{\psi}^\dagger_{\eta,s_1,l_1}(\mathbf{r})\, \hat{\psi}_{\eta,s_2,l_2}(\mathbf{r})\, e^{-i\frac{l_2-l_1}{2}\mathbf{q}_\eta\cdot\mathbf{r}} e^{-2i\sin\left(\frac{\theta}{2}\right)(\mathbf{g}\times\hat{\mathbf{z}})\cdot\mathbf{r}}.
\end{aligned}
\tag{S5.117}
$$

In performing the integral over $\mathbf{y}$, from the first line of Eq. (S5.117), we have used the fact that the summation over $\mathbf{k}$ only runs over a single monolayer BZ, meaning that

$$
\int \mathrm{d}^2 y\, e^{-i\mathbf{k}\cdot\mathbf{y}} e^{i\left(\mathbf{g}+C^\eta_{3z}\mathbf{K}_M\right)\cdot\mathbf{y}} = N\Omega_0 \delta_{\mathbf{g}+C^\eta_{3z}\mathbf{K}_M,\mathbf{k}} = N\Omega_0 \delta_{\mathbf{g},\mathbf{0}} \delta_{C^\eta_{3z}\mathbf{K}_M,\mathbf{k}}. \tag{S5.118}
$$

This completes the derivation of the moiré potential term. At the same time, the intralayer kinetic term can be readily expressed using the low energy operators from Eq. (S4.73) as

$$
\sum_l \mathcal{H}^{\mathrm{sl}}_l \approx -\sum_{\eta,s,l} \int \mathrm{d}^2 r\, \hat{\psi}^\dagger_{\eta,s,l}(\mathbf{r}) \left(C^\eta_{3z}\mathcal{R}^{-1}_{\theta,l}\nabla\right)^T \begin{pmatrix} \frac{1}{2m_x} & 0 \\ 0 & \frac{1}{2m_y} \end{pmatrix} \left(C^\eta_{3z}\mathcal{R}^{-1}_{\theta,l}\nabla\right) \hat{\psi}_{\eta,s,l}(\mathbf{r}). \tag{S5.119}
$$

Comparing Eqs. (S5.117) and (S5.119) with Eq. (S4.75) allows us to immediately identify the moiré potential in real space

$$
V^\eta_{s_1 l_1; s_2 l_2}(\mathbf{r}) = \sum_{\mathbf{g}} S_{s_1 l_1; s_2 l_2}\left(\mathbf{g}, C^\eta_{3z}\mathbf{K}_M\right) e^{-i\frac{l_2-l_1}{2}\mathbf{q}_\eta\cdot\mathbf{r}} e^{-2i\sin\left(\frac{\theta}{2}\right)(\mathbf{g}\times\hat{\mathbf{z}})\cdot\mathbf{r}}, \tag{S5.120}
$$

and, by inverting the Fourier transformation from Eq. (S4.76), we can obtain

$$
\begin{aligned}
\left[T_{\mathbf{q}_{\eta+l_1},\mathbf{q}_{\eta+l_2}+\mathbf{G}}\right]_{s_1 l_1; s_2 l_2} &= \frac{1}{\Omega}\int \mathrm{d}^2 r\, V^\eta_{s_1 l_1; s_2 l_2}(\mathbf{r})\, e^{-i\left(\mathbf{q}_{\eta+l_2}+\mathbf{G}-\mathbf{q}_{\eta+l_1}\right)\cdot\mathbf{r}} \\
&= \frac{1}{\Omega}\int \mathrm{d}^2 r \sum_{\mathbf{g}} S_{s_1 l_1; s_2 l_2}\left(\mathbf{g}, C^\eta_{3z}\mathbf{K}_M\right) e^{-i\frac{l_2-l_1}{2}\mathbf{q}_\eta\cdot\mathbf{r}} e^{-2i\sin\left(\frac{\theta}{2}\right)(\mathbf{g}\times\hat{\mathbf{z}})\cdot\mathbf{r}} e^{-i\left(\mathbf{q}_{\eta+l_2}+\mathbf{G}-\mathbf{q}_{\eta+l_1}\right)\cdot\mathbf{r}} \\
&= \frac{1}{\Omega}\int \mathrm{d}^2 r \sum_{\mathbf{G}'} S_{s_1 l_1; s_2 l_2}\left(\frac{\mathbf{G}'\times\hat{\mathbf{z}}}{2\sin\left(\frac{\theta}{2}\right)}, C^\eta_{3z}\mathbf{K}_M\right) e^{-i\left(\mathbf{q}_{\eta+l_2}+\mathbf{G}-\mathbf{G}'-\mathbf{q}_{\eta+l_1}+\frac{l_2-l_1}{2}\mathbf{q}_\eta\right)\cdot\mathbf{r}} \\
&= S_{s_1 l_1; s_2 l_2}\left(\frac{\left(\mathbf{q}_{\eta+l_2}+\mathbf{G}-\mathbf{q}_{\eta+l_1}+\frac{l_2-l_1}{2}\mathbf{q}_\eta\right)\times\hat{\mathbf{z}}}{2\sin\left(\frac{\theta}{2}\right)}, C^\eta_{3z}\mathbf{K}_M\right) \\
&= S_{s_1 l_1; s_2 l_2}\left(\frac{\left[\mathbf{G}+(l_1-l_2)\,\mathbf{q}_{\eta-1}\right]\times\hat{\mathbf{z}}}{2\sin\left(\frac{\theta}{2}\right)}, C^\eta_{3z}\mathbf{K}_M\right).
\end{aligned}
\tag{S5.121}
$$

We note that for

$$
\frac{\left[\mathbf{G}+(l_1-l_2)\,\mathbf{q}_{\eta-1}\right]\times\hat{\mathbf{z}}}{2\sin\left(\frac{\theta}{2}\right)} = \mathbf{g}, \tag{S5.122}
$$

if follows from our discussion surrounding Eq. (S4.54), that

$$
\mathbf{G} \in \mathbb{Z}\mathbf{b}_{M_1} + \mathbb{Z}\mathbf{b}_{M_2} \quad \text{iff} \quad \mathbf{g} \in \mathbb{Z}\mathbf{b}_1 + \mathbb{Z}\mathbf{b}_2. \tag{S5.123}
$$

As such, we can replace the summation over $\mathbf{g}$ with a summation over $\mathbf{G}$ in the third row of Eq. (S5.121).

3. Constraining the moiré potential

Due to the one-to-one equivalence from Eq. (S5.121), constraining the moiré potential $T_{\mathbf{q}_{\eta+l_1},\mathbf{q}_{\eta+l_2}+\mathbf{G}}$ in the limit of vanishing twist angle is equivalent to constraining $S_{s_1 l_1; s_2 l_2}\left(\mathbf{g}, C_{3z}^{\eta}\mathbf{K}_M\right)$. As a result of the Hermiticity property of Eq. (S5.103), the latter obeys

$$S_{s_1 l_1; s_2 l_2}\left(\mathbf{g}, C_{3z}^{\eta}\mathbf{K}_M\right) = S^*_{s_2 l_2; s_1 l_1}\left(-\mathbf{g}, C_{3z}^{\eta}\mathbf{K}_M\right). \tag{S5.124}$$

Additionally, the constraints imposed by a crystalline symmetry g in Eq. (S5.102) imply that

$$\sum_{s_1,s_2}\left[D^{\mathrm{sl}}(g)\right]_{s_1' s_1} S^{(*)}_{s_1 l_1; s_2 l_2}\left(\mathbf{g}, C_{3z}^{\eta}\mathbf{K}_M\right)\left[D^{\mathrm{sl}}(g)\right]^*_{s_2' s_2} = S_{s_1'\epsilon_g l_1; s_2'\epsilon_g l_2}\left(\epsilon_g g\mathbf{g}, gC_{3z}^{\eta}\mathbf{K}_M\right). \tag{S5.125}$$

The exact symmetries of the heterostructure in the $\theta \neq 0$ case can either be imposed through Eq. (S5.91) or through Eq. (S5.125): the two approaches are equivalent. However, the additional constraints arising from the symmetries of the zero-twist heterostructure can only be imposed through Eq. (S5.125), because they do not correspond to crystalline symmetries of the twisted heterostructure. Moreover, we note that Eq. (S5.125) *constrains* the moiré potential but does *not* impose the crystalline symmetries of the untwisted heterostructure on the full moiré Hamiltonian. For instance, in the zero-twist limit, the AA-stacked moiré Hamiltonian does not acquire inversion symmetry, unlike the untwisted AA-stacked heterostructure. Instead, as we show explicitly in Section VI A, it develops an effective mirror symmetry in the plane of the heterostructure.

To impose the zero-twist constraints, in practice, we first parameterize the moiré potential using the exact $\theta \neq 0$ symmetries, as explained in Section V A. The moiré potential can then be further simplified by imposing Eq. (S5.125) for the symmetries of the zero-twist heterostructure that are not *exact* symmetries of the $\theta \neq 0$ heterostructure. To be specific in the AA-stacking case, $S_{s_1 l_1; s_2 l_2}\left(\mathbf{g}, C_{3z}^{\eta}\mathbf{K}_M\right)$ is additionally constrained by the $\mathcal{I}$ symmetry, while in the AB-stacking case, it is additionally constrained by the M_z symmetry

$$\sum_{s_1,s_2}\left[D^{\mathrm{sl}}(\mathcal{I})\right]_{s_1' s_1} S^{\mathrm{AA}}_{s_1 l_1; s_2 l_2}\left(\mathbf{g}, C_{3z}^{\eta}\mathbf{K}_M\right)\left[D^{\mathrm{sl}}(\mathcal{I})\right]^*_{s_2' s_2} = S^{\mathrm{AA}}_{s_1'(-l_1); s_2'(-l_2)}\left(\mathbf{g}, -C_{3z}^{\eta}\mathbf{K}_M\right), \tag{S5.126}$$

$$\sum_{s_1,s_2}\left[D^{\mathrm{sl}}(M_z)\right]_{s_1' s_1} S^{\mathrm{AB}}_{s_1 l_1; s_2 l_2}\left(\mathbf{g}, C_{3z}^{\eta}\mathbf{K}_M\right)\left[D^{\mathrm{sl}}(M_z)\right]^*_{s_2' s_2} = S^{\mathrm{AB}}_{s_1'(-l_1); s_2'(-l_2)}\left(-\mathbf{g}, C_{3z}^{\eta}\mathbf{K}_M\right). \tag{S5.127}$$

Imposing these constraints on the moiré potential derived in Eqs. (S5.94) and (S5.95) (as will be done explicitly in Section VI A), we find that the latter is characterized by fewer tunneling parameters in this approximation

$$\begin{aligned} w_i^{\mathrm{AA}} &= 0 \quad \text{for} \quad 3 \leq i \leq 6, \\ w_i'^{\mathrm{AA}} &= 0 \quad \text{for} \quad 6 \leq i \leq 10, \\ w_i^{\mathrm{AB}} &= 0 \quad \text{for} \quad 3 \leq i \leq 4, \\ w_i'^{\mathrm{AB}} &= 0 \quad \text{for} \quad 8 \leq i \leq 12. \end{aligned} \tag{S5.128}$$

Specifically, with the zero-twist constraints, there are only seven (nine) real tunneling parameters in the first moiré harmonic case for AA- (AB-)stacking.

VI. ADDITIONAL SYMMETRIES OF THE FIRST MOIRÉ HARMONIC MODEL IN DIFFERENT LIMITS

In this section, we investigate the additional symmetries of the first moiré harmonic model that arise under different limits (*i.e.*, when some of its parameters vanish). For now, we will focus on identifying these limits and their corresponding symmetries, leaving the discussion of how well these limits apply to the *ab initio* Hamiltonian to Section XI. We begin by showing that in the zero-twist limit, the moiré Hamiltonian has additional symmetries in both the AA- and AB-stacked cases. Specifically, under the zero-twist constraints, the moiré Hamiltonian features an additional valley-preserving symmetry, whose action in momentum space is non-symmorphic: in the AA- (AB-)stacked case, this symmetry maps $\mathbf{k}$ to $\mathbf{k} + \mathbf{q}_\eta$ $(-\mathbf{k} + \mathbf{q}_\eta)$ in valley η. In the AA-stacked case, this symmetry resembles an effective mirror-z operation, allowing the moiré Hamiltonian to be block-diagonalized in real space. We also provide a simple lattice model with the same symmetry. In the AB-stacked case, the additional momentum-space non-symmorphic symmetry is equivalent to an effective inversion.

Next, we explore two further limits: the C_{2z} limit, where the moiré Hamiltonian is C_{2z} symmetric, making the AA- and AB-stacked parameterizations identical, and the SU (2) limit, where the model has SU (2) symmetry within each valley. We also investigate the additional effective symmetries of the two-center first monolayer harmonic model. Finally, we perform a symmetry analysis of the models whose moiré Hamiltonian is described by only three parameters and which best reproduce the *ab initio* band structure at small angles of $SnSe_2$ and ZrS_2, as shown in Section XI.

A. Additional symmetries in the zero-twist limit

This first section considers the consequences of Eqs. (S5.126) and (S5.127) on the moiré potential in more detail. Specifically, we will employ Eqs. (S5.126) and (S5.127), as well as the mapping from Eq. (S5.121) to explicitly constrain the moiré potential $T_{\mathbf{Q},\mathbf{Q}'}$ in the zero-twist limit.

1. *Momentum-space non-symmorphic symmetries in both the AA- and AB-stacking configurations*

Starting with the AA-stacking case, we find that Eq. (S5.126) becomes equivalent to

$$\begin{aligned}\sum_{s_1,s_2}\left[D^{\mathrm{sl}}\left(\mathcal{I}\right)\right]_{s_1' s_1} S^{\mathrm{AA}}_{s_1 l_1; s_2 l_2}\left(\mathbf{g}, C_{3z}^{\eta}\mathbf{K}_M\right)\left[D^{\mathrm{sl}}\left(\mathcal{I}\right)\right]^*_{s_2' s_2} &= S^{\mathrm{AA}}_{s_1'(-l_1);s_2'(-l_2)}\left(\mathbf{g}, -C_{3z}^{\eta}\mathbf{K}_M\right)\\ &= S^{\mathrm{AA}}_{s_1'(-l_1);s_2'(-l_2)}\left(\mathbf{g}, C_{3z}^{\eta}\mathbf{K}_M - 2C_{3z}^{\eta}\mathbf{K}_M\right)\\ &= S^{\mathrm{AA}}_{s_1'(-l_1);s_2'(-l_2)}\left(\mathbf{g} + \left(l_2 - l_1\right)C_{3z}^{\eta}\mathbf{K}_M, C_{3z}^{\eta}\mathbf{K}_M\right),\end{aligned} \tag{S6.129}$$

where we have used Eq. (S5.109) in the last line. Since Eq. (S6.129) holds for any $\mathbf{g} \in \mathbb{Z}\mathbf{b}_1 + \mathbb{Z}\mathbf{b}_2$, then for given $0 \leq \eta \leq 2$, $l_1, l_2 = \pm$, and $\mathbf{G} \in \mathcal{Q}$, it should also hold for

$$\mathbf{g} = \frac{\left(\mathbf{q}_{\eta+l_2} + \mathbf{G} - \mathbf{q}_{\eta+l_1} + \frac{l_2 - l_1}{2}\mathbf{q}_\eta\right)\times\hat{\mathbf{z}}}{2\sin\left(\frac{\theta}{2}\right)}. \tag{S6.130}$$

Upon substituting Eq. (S6.130) into Eq. (S6.129), the latter becomes

$$\begin{aligned}&\sum_{s_1,s_2}\left[D^{\mathrm{sl}}\left(\mathcal{I}\right)\right]_{s_1' s_1} S^{\mathrm{AA}}_{s_1 l_1; s_2 l_2}\left(\frac{\left(\mathbf{q}_{\eta+l_2} + \mathbf{G} - \mathbf{q}_{\eta+l_1} + \frac{l_2 - l_1}{2}\mathbf{q}_\eta\right)\times\hat{\mathbf{z}}}{2\sin\left(\frac{\theta}{2}\right)}, C_{3z}^{\eta}\mathbf{K}_M\right)\left[D^{\mathrm{sl}}\left(\mathcal{I}\right)\right]^*_{s_2' s_2}\\ =&S^{\mathrm{AA}}_{s_1'(-l_1);s_2'(-l_2)}\left(\frac{\left(\mathbf{q}_{\eta+l_2} + \mathbf{G} - \mathbf{q}_{\eta+l_1} + \frac{l_2 - l_1}{2}\mathbf{q}_\eta\right)\times\hat{\mathbf{z}}}{2\sin\left(\frac{\theta}{2}\right)} + \left(l_2 - l_1\right)C_{3z}^{\eta}\mathbf{K}_M, C_{3z}^{\eta}\mathbf{K}_M\right)\\ =&S^{\mathrm{AA}}_{s_1'(-l_1);s_2'(-l_2)}\left(\frac{\left[\mathbf{q}_{\eta+l_2} + \mathbf{G} - \mathbf{q}_{\eta+l_1} + \frac{l_2 - l_1}{2}\mathbf{q}_\eta - \left(l_2 - l_1\right)\mathbf{q}_\eta\right]\times\hat{\mathbf{z}}}{2\sin\left(\frac{\theta}{2}\right)}, C_{3z}^{\eta}\mathbf{K}_M\right)\\ =&S^{\mathrm{AA}}_{s_1'(-l_1);s_2'(-l_2)}\left(\frac{\left(\mathbf{q}_{\eta+l_2} + \mathbf{G} - \mathbf{q}_{\eta+l_1} - \frac{l_2 - l_1}{2}\mathbf{q}_\eta\right)\times\hat{\mathbf{z}}}{2\sin\left(\frac{\theta}{2}\right)}, C_{3z}^{\eta}\mathbf{K}_M\right)\\ =&S^{\mathrm{AA}}_{s_1'(-l_1);s_2'(-l_2)}\left(\frac{\left[\mathbf{q}_{\eta-l_2} + \left(\mathbf{G} - 2\mathbf{q}_{\eta-l_2} + 2\mathbf{q}_{\eta-l_1}\right) - \mathbf{q}_{\eta-l_1} + \frac{(-l_2)-(-l_1)}{2}\mathbf{q}_\eta\right]\times\hat{\mathbf{z}}}{2\sin\left(\frac{\theta}{2}\right)}, C_{3z}^{\eta}\mathbf{K}_M\right),\end{aligned} \tag{S6.131}$$

where, in the last equality, we have applied $\mathbf{q}_{\eta+l} + \mathbf{q}_\eta = -\mathbf{q}_{\eta-l} = \mathbf{q}_{\eta-l} - 2\mathbf{q}_{\eta-l}$. We now apply the one-to-one mapping between the $S_{s_1 l_1; s_2 l_2}\left(\mathbf{g}, C_{3z}^{\eta}\mathbf{K}_M\right)$ and $T_{\mathbf{q}_{\eta+l_1},\mathbf{q}_{\eta+l_2}+\mathbf{G}}$ matrices from Eq. (S5.121), along with the representation matrix of $\mathcal{I}$ from Eq. (S2.6). Together with Eq. (S6.131), this allows us to show that in the zero-twist limit, we have

$$\begin{aligned}\left[T^{\mathrm{AA}}_{\mathbf{q}_{\eta+l_1},\mathbf{q}_{\eta+l_2}+\mathbf{G}}\right]_{s_1 l_1; s_2 l_2} &= \left[T^{\mathrm{AA}}_{\mathbf{q}_{\eta-l_1},\mathbf{q}_{\eta-l_2}+\mathbf{G}-2\mathbf{q}_{\eta-l_2}+2\mathbf{q}_{\eta-l_1}}\right]_{s_1(-l_1);s_2(-l_2)}\\ &= \left[T^{\mathrm{AA}}_{-\mathbf{q}_{\eta-l_1},-\mathbf{q}_{\eta-l_2}+\mathbf{G}}\right]_{s_1(-l_1);s_2(-l_2)}\\ &= \left[T^{\mathrm{AA}}_{\mathbf{q}_{\eta+l_1}+\mathbf{q}_\eta,\mathbf{q}_{\eta+l_2}+\mathbf{G}+\mathbf{q}_\eta}\right]_{s_1(-l_1);s_2(-l_2)}.\end{aligned} \tag{S6.132}$$

Before investigating the consequences of Eq. (S6.132) on the moiré Hamiltonian, we also consider the AB-stacking case, where evaluating Eq. (S5.127) at the monolayer reciprocal vector from Eq. (S6.130) leads to

$$\sum_{s_1,s_2} \left[D^{\mathrm{sl}}\left(M_z\right)\right]_{s_1' s_1} S^{\mathrm{AB}}_{s_1 l_1; s_2 l_2} \left(\frac{\left(\mathbf{q}_{\eta+l_2} + \mathbf{G} - \mathbf{q}_{\eta+l_1} + \frac{l_2-l_1}{2}\mathbf{q}_\eta\right)\times \hat{\mathbf{z}}}{2\sin\left(\frac{\theta}{2}\right)}, C^{\eta}_{3z}\mathbf{K}_M \right) \left[D^{\mathrm{sl}}\left(M_z\right)\right]^*_{s_2' s_2}$$
$$= S^{\mathrm{AB}}_{s_1'(-l_1); s_2'(-l_2)} \left(\frac{\left(-\mathbf{q}_{\eta+l_2} - \mathbf{G} + \mathbf{q}_{\eta+l_1} - \frac{l_2-l_1}{2}\mathbf{q}_\eta\right)\times \hat{\mathbf{z}}}{2\sin\left(\frac{\theta}{2}\right)}, C^{\eta}_{3z}\mathbf{K}_M \right). \tag{S6.133}$$

Using again Eq. (S5.121), Eq. (S6.133) implies that in the zero-twist limit, the moiré potential matrix obeys

$$\sum_{s_1,s_2} \left[D^{\mathrm{sl}}\left(M_z\right)\right]_{s_1' s_1} \left[T^{\mathrm{AB}}_{\mathbf{q}_{\eta+l_1},\mathbf{q}_{\eta+l_2}+\mathbf{G}}\right]_{s_1 l_1; s_2 l_2} \left[D^{\mathrm{sl}}\left(M_z\right)\right]^*_{s_2' s_2} = \left[T^{\mathrm{AB}}_{-\mathbf{q}_{\eta+l_1}+\mathbf{q}_\eta, -\mathbf{q}_{\eta+l_2}-\mathbf{G}+\mathbf{q}_\eta}\right]_{s_1'(-l_1); s_2'(-l_2)}, \tag{S6.134}$$

with the reader being reminded that the representation matrix of the M_z symmetry was given in Eq. (S4.48) and reads as $D^{\mathrm{sl}}\left(M_z\right) = -i s_z$.

When the zero-twist constraints are imposed, one should also ignore the relative rotation of the monolayer kinetic terms appearing in Eq. (S4.68) and approximate the first-quantized Hamiltonian $h_{\mathbf{Q},\mathbf{Q}'}\left(\mathbf{k}\right)$ by

$$\left[h_{\mathbf{Q},\mathbf{Q}'}\left(\mathbf{k}\right)\right]_{s_1 l_1; s_2 l_2} = \delta_{\mathbf{Q},\mathbf{Q}'}\delta_{s_1 s_2}\delta_{l_1 l_2} \left(\frac{\delta k_x^2}{2m_x} + \frac{\delta k_y^2}{2m_y} \right)\Bigg|_{\delta\mathbf{k} = C_{3z}^{l_1-\zeta_{\mathbf{Q}}}(\mathbf{k}-\mathbf{Q})} + \left[T_{\mathbf{Q},\mathbf{Q}'}\right]_{s_1 l_1; s_2 l_2}, \tag{S6.135}$$

where the moiré potential $T_{\mathbf{Q},\mathbf{Q}'}$ matrix obeys the zero-twist constraints from Eqs. (S6.132) and (S6.134), in that AA- and AB-stacking case, respectively. We find that *in the zero-twist limit*, and depending on the stacking configuration, the moiré Hamiltonian obeys one of two symmetries whose action is *non-symmorphic* in momentum space [35, 36]

$$\left[h^{\mathrm{AA}}_{\mathbf{Q},\mathbf{Q}'}\left(\mathbf{k}\right)\right]_{s_1 l_1; s_2 l_2} = \left[h^{\mathrm{AA}}_{\mathbf{Q}+\mathbf{q}_\eta,\mathbf{Q}'+\mathbf{q}_\eta}\left(\mathbf{k}+\mathbf{q}_\eta\right)\right]_{s_1(-l_1); s_2(-l_2)}, \tag{S6.136}$$

$$\sum_{s_1,s_2} \left[D^{\mathrm{sl}}\left(M_z\right)\right]_{s_1' s_1} \left[h^{\mathrm{AB}}_{\mathbf{Q},\mathbf{Q}'}\left(\mathbf{k}\right)\right]_{s_1 l_1; s_2 l_2} \left[D^{\mathrm{sl}}\left(M_z\right)\right]^*_{s_2' s_2} = \left[h^{\mathrm{AB}}_{-\mathbf{Q}+\mathbf{q}_\eta,-\mathbf{Q}'+\mathbf{q}_\eta}\left(-\mathbf{k}+\mathbf{q}_\eta\right)\right]_{s_1(-l_1); s(-l_2)}, \tag{S6.137}$$

for $\mathbf{Q} \in \mathcal{Q}_{\eta+l_1}, \mathbf{Q}' \in \mathcal{Q}_{\eta+l_2}$, with $0 \leq \eta \leq 2$. Specifically, in the AA-stacking case and in the zero-twist limit, the system is symmetric under an effective mirror-z symmetry, which we denote as $\tilde{M}_z$ and whose action on the moiré fermions is given by

$$\tilde{M}_z \hat{c}^\dagger_{\mathbf{k},\mathbf{Q},s,l}\tilde{M}_z^{-1} = \hat{c}^\dagger_{\mathbf{k}+\mathbf{q}_\eta,\mathbf{Q}+\mathbf{q}_\eta,s,-l}, \quad \text{for} \quad \mathbf{Q}\in\mathcal{Q}_{\eta+l}, \quad \text{and} \quad \tilde{M}_z \hat{\psi}^\dagger_{\eta,s,l}\left(\mathbf{r}\right)\tilde{M}_z^{-1} = \hat{\psi}^\dagger_{\eta,s,-l}\left(\mathbf{r}\right). \tag{S6.138}$$

In the zero twist limit $\left[\tilde{M}_z, \mathcal{H}^{\mathrm{AA}}\right] = 0$. At the same time, in the AB-stacking case, the moiré Hamiltonian is symmetric under an effective inversion symmetry, which we denote as $\tilde{\mathcal{I}}$, and whose action on the moiré fermions reads as

$$\tilde{\mathcal{I}} \hat{c}^\dagger_{\mathbf{k},\mathbf{Q},s,l}\tilde{\mathcal{I}}^{-1} = (-1)^s \hat{c}^\dagger_{-\mathbf{k}+\mathbf{q}_\eta,-\mathbf{Q}+\mathbf{q}_\eta,s,-l}, \quad \text{for} \quad \mathbf{Q}\in\mathcal{Q}_{\eta+l}, \quad \text{and} \quad \tilde{\mathcal{I}} \hat{\psi}^\dagger_{\eta,s,l}\left(\mathbf{r}\right)\tilde{\mathcal{I}}^{-1} = (-1)^s \hat{\psi}^\dagger_{\eta,s,-l}\left(-\mathbf{r}\right), \tag{S6.139}$$

where the factor $(-1)^s$ is given by

$$(-1)^s = \begin{cases} +1 & \text{for} \quad s=\uparrow \\ -1 & \text{for} \quad s=\downarrow \end{cases}. \tag{S6.140}$$

In the zero-twist limit, $\left[\tilde{\mathcal{I}}, \mathcal{H}^{\mathrm{AB}}\right] = 0$.

We note that both $\tilde{M}_z$ and $\tilde{\mathcal{I}}$ are termed effective symmetries because, while their action on the spatial degrees of freedom is conventional, their action on the spins does not match a conventional mirror-z or inversion symmetry. Additionally, both $\tilde{M}_z$ and $\tilde{\mathcal{I}}$ preserve the valley quantum number, meaning that they enhance the single-valley symmetry group. Furthermore, when the zero-twist constraints are imposed, $\tilde{M}_z$ ensures that the spectra of the AA-stacked moiré Hamiltonian in valley η at $\mathbf{k}$ and $\mathbf{k}+\mathbf{q}_\eta$ are identical. This can be seen from Eq. (S6.136), which implies that in the zero-twist limit, the valley-η blocks of the Hamiltonian matrix at $\mathbf{k}$ and $\mathbf{k}+\mathbf{q}_\eta$ are unitarily related. Similarly, in the AB-stacked case, $\tilde{\mathcal{I}}$ ensures that the spectra of the moiré Hamiltonian in valley η at $\mathbf{k}$ and $-\mathbf{k}+\mathbf{q}_\eta$ are also identical, in the zero-twist limit.

It is important to note that Eqs. (S6.136) and (S6.137) hold if and only if the zero-twist constraints from Eqs. (S5.126) and (S5.127) are imposed, respectively. Consequently, the AA- and AB-stacked moiré Hamiltonians retain the momentum-space non-symmorphic symmetries $\tilde{M}_z$ and $\tilde{\mathcal{I}}$ even *beyond* the first moiré harmonic level, as long as the zero-twist constraints from Eqs. (S5.126) and (S5.127) are enforced.

For AA-stacking, the $\tilde{M}_z$ symmetry preserves the spatial position and therefore allows one to define an even and odd basis for the real-space low-energy fermions. We will now discuss this in more detail in the following Section VI A 2.

2. Even-odd basis of the AA-stacked moiré Hamiltonian

As shown in Eq. (S6.138) the AA-stacked configuration the effective $\tilde{M}_z$ symmetry does not change the spatial position of the real-space moiré fermion operators. Therefore, we can introduce fermion operators that diagonalize the $\tilde{M}_z$ operator at a given position

$$\hat{\varphi}^\dagger_{\eta,s,p}(\mathbf{r}) \equiv \frac{1}{\sqrt{2}}\left(\hat{\psi}^\dagger_{\eta,s,+}(\mathbf{r}) + p\hat{\psi}^\dagger_{\eta,s,-}(\mathbf{r})\right), \tag{S6.141}$$

where $p=\pm 1$ denotes the parity of the $\hat{\varphi}^\dagger_{\eta,s,p}(\mathbf{r})$ operator under the $\tilde{M}_z$ symmetry. The action of the $\tilde{M}_z$ symmetry and of the other (exact) symmetries of the AA-stacked moiré heterostructure on the $\tilde{M}_z$-symmetric states are given by

$$g\hat{\varphi}^\dagger_{\eta_1,s_1,p_1}(\mathbf{r})\,g^{-1} = \sum_{\eta_2,s_2,p_2}\left[D_\varphi(g)\right]_{\eta_2 s_2 p_2;\eta_1 s_1 p_1}\hat{\varphi}^\dagger_{\eta_2,s_2,p_2}(g\mathbf{r}), \tag{S6.142}$$

with the corresponding real space representation matrices reading as

$$\begin{aligned}
D_\varphi(\mathcal{T}) &= i\begin{pmatrix}1&0&0\\0&1&0\\0&0&1\end{pmatrix}s_y\xi_0, \qquad D_\varphi(C_{3z}) = \begin{pmatrix}0&0&1\\1&0&0\\0&1&0\end{pmatrix}e^{-\frac{\pi i}{3}s_z}\xi_0,\\
D_\varphi(C_{2x}) &= -i\begin{pmatrix}1&0&0\\0&0&1\\0&1&0\end{pmatrix}s_x\xi_z, \quad D_\varphi\left(\tilde{M}_z\right) = \begin{pmatrix}1&0&0\\0&1&0\\0&0&1\end{pmatrix}s_0\xi_z,
\end{aligned} \tag{S6.143}$$

where ξ_a (for $a=0,x,y,z$) are the identity and the three Pauli matrices acting on the $\tilde{M}_z$-symmetric fermionic operators. Note that the layer-exchanging C_{2x} symmetry also becomes diagonal in parity in the even-odd basis. Under the moiré translation operators, the $\tilde{M}_z$-symmetric operators transform as

$$\begin{aligned}
T_{\mathbf{R}_M}\hat{\varphi}^\dagger_{\eta,s,p}(\mathbf{r})\,T^{-1}_{\mathbf{R}_M} &= \hat{\psi}^\dagger_{\eta,s,+}(\mathbf{r}+\mathbf{R}_M)\,e^{-iC^\eta_{3z}\mathbf{K}^+_M\cdot\mathbf{R}_M} + p\hat{\psi}^\dagger_{\eta,s,-}(\mathbf{r}+\mathbf{R}_M)\,e^{-iC^\eta_{3z}\mathbf{K}^-_M\cdot\mathbf{R}_M}\\
&= \left[\hat{\psi}^\dagger_{\eta,s,+}(\mathbf{r}+\mathbf{R}_M) + pe^{-iC^\eta_{3z}\left(\mathbf{K}^-_M-\mathbf{K}^+_M\right)\cdot\mathbf{R}_M}\hat{\psi}^\dagger_{\eta,s,-}(\mathbf{r}+\mathbf{R}_M)\right]e^{-iC^\eta_{3z}\mathbf{K}^+_M\cdot\mathbf{R}_M}\\
&= \left(\hat{\psi}^\dagger_{\eta,s,+}(\mathbf{r}+\mathbf{R}_M) + pe^{-i\mathbf{q}_\eta\cdot\mathbf{R}_M}\hat{\psi}^\dagger_{\eta,s,-}(\mathbf{r}+\mathbf{R}_M)\right)e^{-iC^\eta_{3z}\mathbf{K}^+_M\cdot\mathbf{R}_M}\\
&= \hat{\varphi}^\dagger_{\eta,s,pe^{-i\mathbf{q}_\eta\cdot\mathbf{R}_M}}(\mathbf{r}+\mathbf{R}_M)\,e^{-iC^\eta_{3z}\mathbf{K}^+_M\cdot\mathbf{R}_M}, \quad \text{for} \quad \mathbf{R}_M\in\mathbb{Z}\mathbf{a}_{M_1}+\mathbb{Z}\mathbf{a}_{M_2},
\end{aligned} \tag{S6.144}$$

where $e^{-i\mathbf{q}_\eta\cdot\mathbf{R}_M}=\pm 1$. In particular, we find that

$$T_{C^\eta_{3z}\mathbf{a}_{M_1}}\hat{\varphi}^\dagger_{\eta,s,p}(\mathbf{r})\,T^{-1}_{C^\eta_{3z}\mathbf{a}_{M_1}} = \hat{\varphi}^\dagger_{\eta,s,-p}(\mathbf{r}+C^\eta_{3z}\mathbf{a}_{M_1})\,e^{-iC^\eta_{3z}\mathbf{K}^+_M\cdot\mathbf{a}_{M_1}}, \tag{S6.145}$$

$$T_{C^\eta_{3z}\mathbf{a}_{M_2}}\hat{\varphi}^\dagger_{\eta,s,p}(\mathbf{r})\,T^{-1}_{C^\eta_{3z}\mathbf{a}_{M_1}} = \hat{\varphi}^\dagger_{\eta,s,p}(\mathbf{r}+C^\eta_{3z}\mathbf{a}_{M_2})\,e^{-iC^\eta_{3z}\mathbf{K}^+_M\cdot\mathbf{a}_{M_2}}, \tag{S6.146}$$

which implies that $T_{C^\eta_{3z}\mathbf{a}_{M_1}}$ not only effects a translation of the $\tilde{M}_z$-symmetric fermionic operators, but also exchanges their parity (unlike a conventional mirror-z symmetry).

In the zero-twist limit, the AA-stacked moiré Hamiltonian becomes block-diagonal in the $\tilde{M}_z$-symmetric basis

$$\mathcal{H}^{\text{AA}} = -\sum_{\eta,s,p}\int \mathrm{d}^2r\,\hat{\varphi}^\dagger_{\eta,s,p}(\mathbf{r})\left(C^{-\eta}_{3z}\nabla\right)^T\begin{pmatrix}\frac{1}{2m_x}&0\\0&\frac{1}{2m_y}\end{pmatrix}\left(C^{-\eta}_{3z}\nabla\right)\hat{\varphi}_{\eta,s,p}(\mathbf{r})$$

$$+\sum_{\eta,p,s_1,s_2}\int \mathrm{d}^2r\, W^{\eta,p}_{s_1s_2}\left(\mathbf{r}\right)\hat{\varphi}^{\dagger}_{\eta,s_1,p}\left(\mathbf{r}\right)\hat{\varphi}_{\eta,s_2,p}\left(\mathbf{r}\right), \tag{S6.147}$$

with the $\tilde{M}_z$-symmetric real-space moiré potential being given by

$$W^{\eta,p}_{s_1s_2}\left(\mathbf{r}\right)=V^{\eta,\mathrm{AA}}_{s_1+;s_2+}\left(\mathbf{r}\right)+pV^{\eta,\mathrm{AA}}_{s_1+;s_2-}\left(\mathbf{r}\right). \tag{S6.148}$$

The $\tilde{M}_z$-even and $\tilde{M}_z$-odd electrons are therefore decoupled at the single-particle level. Additionally, as a result of the $\tilde{M}_z$ symmetry from Eq. (S6.132), the real-space AA-stacked moiré potential obeys

$$V^{\eta,\mathrm{AA}}_{s_1l_1;s_2l_2}\left(\mathbf{r}\right)=V^{\eta,\mathrm{AA}}_{s_1(-l_1);s_2(-l_2)}\left(\mathbf{r}\right). \tag{S6.149}$$

As a result of Eqs. (S4.78) and (S4.79), the $\tilde{M}_z$-symmetric real-space moiré potential satisfies the following periodicity condition

$$W^{\eta,p}_{s_1s_2}\left(\mathbf{r}+\mathbf{a}^{\eta}_{W_1}\right)=W^{\eta,p}_{s_1s_2}\left(\mathbf{r}\right),\quad W^{\eta,p}_{s_1s_2}\left(\mathbf{r}+\mathbf{a}^{\eta}_{W_2}\right)=W^{\eta,p}_{s_1s_2}\left(\mathbf{r}\right), \tag{S6.150}$$

where we have defined the following *rectangular* lattice vectors

$$\mathbf{a}^{\eta}_{W_1}=2C^{\eta}_{3z}\mathbf{a}_{M_1}+C^{\eta}_{3z}\mathbf{a}_{M_2}.\quad\text{and}\quad \mathbf{a}^{\eta}_{W_2}=C^{\eta}_{3z}\mathbf{a}_{M_2}. \tag{S6.151}$$

Moreover, as a result of Eqs. (S4.78) and (S4.79), we can show that

$$W^{\eta,+1}_{s_1s_2}\left(\mathbf{r}+C^{\eta}_{3z}\mathbf{a}_{M_1}\right)=W^{\eta,-1}_{s_1s_2}\left(\mathbf{r}\right), \tag{S6.152}$$

which enables us to rewrite the AA-stacked moiré Hamiltonian in the zero-twist limit as

$$\begin{aligned}\mathcal{H}^{\mathrm{AA}}=&-\sum_{\eta,s,p}\int \mathrm{d}^2r\,\hat{\varphi}^{\dagger}_{\eta,s,p}\left(\mathbf{r}\right)\left(C^{-\eta}_{3z}\nabla\right)^T\begin{pmatrix}\frac{1}{2m_x} & 0\\ 0 & \frac{1}{2m_y}\end{pmatrix}\left(C^{-\eta}_{3z}\nabla\right)\hat{\varphi}_{\eta,s,p}\left(\mathbf{r}\right)\\&+\sum_{\eta,s_1,s_2}\int \mathrm{d}^2r\, W^{\eta,+1}_{s_1s_2}\left(\mathbf{r}\right)\left(\hat{\varphi}^{\dagger}_{\eta,s_1,+1}\left(\mathbf{r}\right)\hat{\varphi}_{\eta,s_2,+1}\left(\mathbf{r}\right)+\hat{\varphi}^{\dagger}_{\eta,s_1,-1}\left(\mathbf{r}+C^{\eta}_{3z}\mathbf{a}_{M_1}\right)\hat{\varphi}_{\eta,s_2,-1}\left(\mathbf{r}+C^{\eta}_{3z}\mathbf{a}_{M_1}\right)\right).\end{aligned} \tag{S6.153}$$

Thus, within the valley η, each $\tilde{M}_z$-symmetry sector from the Hamiltonian in Eq. (S6.147) is equivalent to the problem of a free fermion $\hat{\varphi}^{\dagger}_{\eta,s,p}\left(\mathbf{r}\right)$ moving in a conventional two-dimensional periodic *rectangular* potential $W^{\eta,p}_{s_1s_2}\left(\mathbf{r}\right)$, with periodicity defined by the lattice vectors $\mathbf{a}^{\eta}_{W_i}$ (for $i=1,2$). These vectors define a unit cell that is twice the size of the original moiré unit cell. Since the translation operator $T_{C^{\eta}_{3z}\mathbf{a}_{M_1}}$ maps between the two mirror sectors, as shown in Eq. (S6.145), it is sufficient to focus on just one $\tilde{M}_z$-symmetry sector when analyzing the single-particle properties of the system.

Now, consider the scenario where the system exhibits spin SU(2) symmetry, such as the limit discussed in Section VI C. Assuming an isolated spin-degenerate band is trivial, it can support exponentially localized Wannier orbitals that respect the system's symmetries. To be specific, we focus on the $\eta=0$ valley. In this case, the Wannier orbitals of the isolated spinful band, corresponding to different $\tilde{M}_z$ sectors, are decoupled at the single-particle level and form a rectangular lattice spanned by $\mathbf{a}^{0}_{W_i}$ (for $i=1,2$). Since the translation operator $T_{\mathbf{a}_{M_1}}$ maps between the two mirror sectors in the $\eta=0$ valley, we find that the Wannier centers of the $\tilde{M}_z$-even and $\tilde{M}_z$-odd fermions are shifted by $\mathbf{a}_{M_1}$. When combining the Wannier orbitals of the even and odd electrons, they form the hexagonal moiré lattice spanned by $\mathbf{a}_{M_i}$ (for $i=1,2$). However, if we consider only the electrons in the even (or odd) basis and assume that the Wannier orbitals have approximately isotropic spread, then the hopping along the $\hat{\mathbf{y}}$-direction must be much stronger than the one along the $\hat{\mathbf{x}}$-direction. This asymmetry arises because the distance between two neighboring Wannier orbitals along the $\hat{\mathbf{x}}$-direction ($\left|\mathbf{a}^{0}_{W_1}\right|$) is larger than the distance along the $\hat{\mathbf{y}}$-direction ($\left|\mathbf{a}^{0}_{W_2}\right|=\frac{\left|\mathbf{a}^{0}_{W_1}\right|}{\sqrt{3}}$). Consequently, the system behaves effectively as one-dimensional at the single-particle level, with flat(er) dispersion along the $\hat{\mathbf{x}}$-direction in valley $\eta=0$.

3. Toy model for momentum-space non-symmorphic symmetry

To better understand the momentum-space non-symmorphic $\tilde{M}_z$ symmetry of the AA-stacked moiré Hamiltonian in the zero-twist limit, we build a simple one-dimensional tight-binding toy model that also harbors such a symmetry.

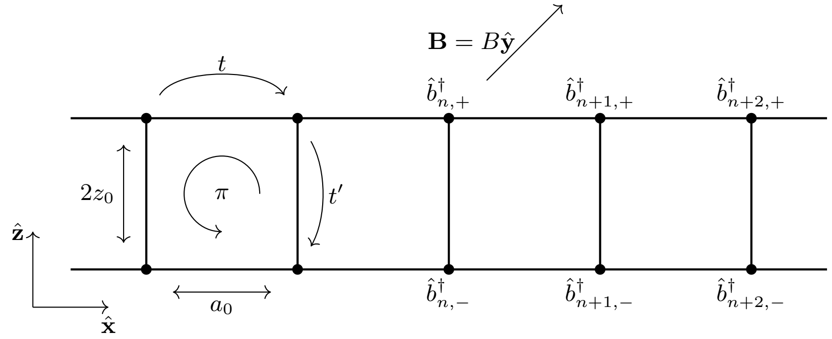

FIG. S16. Lattice model with momentum-space non-symmorphic $\tilde{M}_z$ symmetry. We consider a one-dimensional ladder of fermions. The real hopping amplitudes along the $\hat{\mathbf{x}}$ and $\hat{\mathbf{z}}$ directions are given by t and t', respectively. Additionally, each plaquette is pierced by a π magnetic flux stemming from a magnetic field applied along the $\hat{\mathbf{y}}$ direction.

The connection between this toy model and the AA-stacked moiré Hamiltonian will be explained more carefully in Section VI A 4. We consider two chains of fermions located at

$$\left\{(na_0, z_0)\,\middle|n \in \mathbb{Z}\right\} \cup \left\{(na_0, -z_0)\,\middle|n \in \mathbb{Z}\right\} \tag{S6.154}$$

and let $\hat{b}^\dagger_{n,\pm}$ create an s orbital electron at position $(na_0, \pm z_0)$. We then introduce a magnetic flux along the $\hat{\mathbf{y}}$ direction such that there is a π magnetic flux for each small plaquette with vertices at

$$(na_0, z_0)\,, \quad ((n+1)a_0, z_0)\,, \quad ((n+1)a_0, -z_0)\,, \quad (na_0, -z_0)\,. \tag{S6.155}$$

We set the nearest-neighbor zero-field *real* hopping amplitudes to be t and t' along the $\hat{\mathbf{x}}$ and $\hat{\mathbf{z}}$ directions, as depicted in Fig. S16. In the presence of a magnetic field, a phase is introduced to all hoppings such that the product of phase factors around each plaquette equals $e^{i\pi}$ (corresponding to the π flux induced by the magnetic field). We work in a gauge where the hopping of the bottom fermions acquires a negative sign, resulting in the following Hamiltonian for the system

$$\hat{H}_{1\mathrm{D}} = \sum_{n,l} t\left(l\hat{b}^\dagger_{n,l}\hat{b}_{n+1,l} + \text{h.c.}\right) + \sum_{n} t'\left(\hat{b}^\dagger_{n,+}\hat{b}_{n,-} + \text{h.c.}\right). \tag{S6.156}$$

In Eq. (S6.156), the additional phase factor $l = \pm$ stems from the nonzero magnetic flux piercing each plaquette.

We now discuss the symmetries of the model. The system has translation symmetry T_{a_0} and C_{2y} rotation symmetry. Additionally, the system also has $\tilde{M}_z$ and $\mathcal{T}$ symmetry. This is because, under either $\tilde{M}_z$ or $\mathcal{T}$, the magnetic field (and hence the magnetic flux) will flip sign. Since a π flux and a $-\pi$ flux are equivalent, $\tilde{M}_z$ and $\mathcal{T}$ are symmetries of the system. The actions of these symmetries on the fermions of the model are given by

$$\begin{aligned} T_{a_0}\hat{b}^\dagger_{n,l}T^{-1}_{a_0} &= \hat{b}^\dagger_{n+1,l}, & \mathcal{T}\hat{b}^\dagger_{n,l}\mathcal{T}^{-1} &= \hat{b}^\dagger_{n,l}, \\ \tilde{M}_z\hat{b}^\dagger_{n,l}\tilde{M}^{-1}_z &= \hat{b}^\dagger_{n,-l}(-1)^n, & C_{2y}\hat{b}^\dagger_{n,l}C^{-1}_{2y} &= il\hat{b}^\dagger_{-n,-l}. \end{aligned} \tag{S6.157}$$

By considering the successive action of the T_{a_0} and $\tilde{M}_z$ operators

$$\begin{aligned} T_{a_0}\tilde{M}_z\hat{b}^\dagger_{n,l}\tilde{M}^{-1}_z T^{-1}_{a_0} &= (-1)^n\hat{b}^\dagger_{n+1,-l}, \\ \tilde{M}_z T_{a_0}\hat{b}^\dagger_{n,l}T^{-1}_{a}\tilde{M}^{-1}_z &= (-1)^{n+1}\hat{b}^\dagger_{n+1,-l}, \\ T_{a_0}\tilde{M}_z\hat{b}^\dagger_{n,l}\tilde{M}^{-1}_z T^{-1}_{a_0} &= -\tilde{M}_z T_{a_0}\hat{b}^\dagger_{n,l}T^{-1}_{a_0}\tilde{M}^{-1}_z, \end{aligned} \tag{S6.158}$$

we can see that the real-space operators $\hat{b}^\dagger_{n,l}$ form a projective representation of the space group since [35, 36]

$$\left\{\tilde{M}_z, T_{a_0}\right\} = 0, \tag{S6.159}$$

which arises from the applied magnetic π flux.

We now discuss single-particle dispersion of the $\hat{H}_{1\mathrm{D}}$ Hamiltonian. Since $\left[\hat{H}_{1\mathrm{D}}, T_{a_0}\right]$, we can introduce the following momentum space operators

$$\hat{b}^\dagger_{k,l} = \frac{1}{\sqrt{N}} \sum_k \hat{b}^\dagger_{n,l} e^{ikn}. \tag{S6.160}$$

Next, consider a certain eigenstate of the single particle Hamiltonian $\hat{H}_{1D}$ at momentum k

$$|u_k\rangle = \sum_l u_{k,l} \hat{b}^\dagger_{k,l} |0\rangle, \tag{S6.161}$$

where $u_{k,l}$ is the wave function of the state and

$$\hat{H}_{1\mathrm{D}} |u_k\rangle = E_k |u_k\rangle. \tag{S6.162}$$

From $\tilde{M}_z$ symmetry, we find

$$\begin{aligned}\tilde{M}_z |u_k\rangle &= \tilde{M}_z \sum_l u_{k,l} \sum_n \frac{1}{\sqrt{N}} \hat{b}^\dagger_{n,l} e^{ikn} |0\rangle \\ &= \sum_l u_{k,l} \sum_n \frac{1}{\sqrt{N}} \hat{b}^\dagger_{n,-l} e^{ikn + i\pi n} |0\rangle = \sum_l u_{k,l} \hat{b}^\dagger_{k+\pi,-l} |0\rangle,\end{aligned} \tag{S6.163}$$

meaning that $\tilde{M}_z$ maps a state with momentum k to a state with momentum $k+\pi$. Since $\tilde{M}_z$ commutes with Hamiltonian, $\tilde{M}_z |u_k\rangle$ is also a eingenstate of the Hamiltonian $\hat{H}_{1\mathrm{D}}$ with same eigenvalue E_k. Therefore, we conclude the spectra of the Hamiltonian at k and $k+\pi$ are identical. To show this explicitly, we rewrite the Hamiltonian $\hat{H}_{1\mathrm{D}}$ in momentum space

$$\hat{H}_{1\mathrm{D}} = \sum_{l,k} 2lt \cos(k) \hat{b}^\dagger_{k,l} \hat{b}_{k,l} + \sum_{l,k} t' \left(\hat{b}^\dagger_{k,+} \hat{b}_{k,-} + \text{h.c.} \right). \tag{S6.164}$$

The dispersion of the Hamiltonian is given by

$$E_{k,\pm} = \pm \sqrt{\left(2t\cos(k)\right)^2 + t'^2} \tag{S6.165}$$

and is seen to obey $E_{k+\pi,\pm} = E_{k,\pm}$. We note that one can also make the action of $\tilde{M}_z$ symmorphic in momentum space by "folding" the BZ so that k and $k+\pi$ are identified. This is equivalent to doubling the real-space unit cell. However, adopting this convention obscures the fact that the system retains translation symmetry generated by T_{a_0}, rather than by T_{2a_0}.

4. Connecting the toy model and AA-stacked moiré Hamiltonian

In the simple one-dimensional model from Eq. (S6.156), the non-symmorphic action of the $\tilde{M}_z$ symmetry in momentum space (*i.e.*, its anticommutation with the T_{a_0} translation operator) is seen to arise from an externally applied magnetic field. To make the connection between $\tilde{M}_z$ and a magnetic field more apparent for the moiré AA-stacked Hamiltonian with the zero-twist constraints imposed, we can make a gauge transformation and define new operators

$$\hat{\Psi}^\dagger_{\eta,s,l}(\mathbf{r}) = \hat{\psi}^\dagger_{\eta,s,l}(\mathbf{r}) e^{-il\mathbf{q}_\eta \cdot \mathbf{r}/2}. \tag{S6.166}$$

As we show below, by expressing the AA-stacked moiré Hamiltonian with zero-twist constraints in this new basis, the presence of an effective magnetic field becomes explicit

$$\begin{aligned}\mathcal{H}^{\mathrm{AA}} =& \sum_{\eta,s,l} \int \mathrm{d}^2 r\, \hat{\Psi}^\dagger_{\eta,s,l}(\mathbf{r}) \left[C_{3z}^{-\eta} \left(-i\nabla - e\mathbf{A}^\eta_l(\mathbf{r}) \right) \right]^T \begin{pmatrix} \frac{1}{2m_x} & 0 \\ 0 & \frac{1}{2m_y} \end{pmatrix} \left[C_{3z}^{-\eta} \left(-i\nabla - e\mathbf{A}^\eta_l(\mathbf{r}) \right) \right] \hat{\Psi}_{\eta,s,l}(\mathbf{r}) \\ &+ \sum_{\eta,l,s_1,s_2} \int \mathrm{d}^2 r \left(\tilde{T}^{\eta,l}_{s_1 s_2}(\mathbf{r}) \hat{\Psi}^\dagger_{\eta,s_1,l}(\mathbf{r}) \hat{\Psi}_{\eta,s_2,(-l)}(\mathbf{r}) + \tilde{V}^\eta_{s_1 s_2}(\mathbf{r}) \hat{\Psi}^\dagger_{\eta,s_1,l}(\mathbf{r}) \hat{\Psi}_{\eta,s_2,l}(\mathbf{r}) \right).\end{aligned} \tag{S6.167}$$

In Eq. (S6.167), we have employed Eq. (S6.149) and have also defined the following intra-, inter- and vector potentials

$$\tilde{T}^{\eta,l}_{s_1 s_2}(\mathbf{r}) = V^{\eta,\mathrm{AA}}_{s_1+;s_2-}(\mathbf{r})\, e^{il\mathbf{q}_\eta\cdot\mathbf{r}}, \tag{S6.168}$$

$$\tilde{V}^{\eta}_{s_1 s_2}(\mathbf{r}) = V^{\eta,\mathrm{AA}}_{s_1+;s_2+}(\mathbf{r}), \tag{S6.169}$$

$$\mathbf{A}^{\eta}_{l}(\mathbf{r}) = -\frac{l}{2e}\mathbf{q}_\eta, \tag{S6.170}$$

with e being the electronic charge. In this new basis, both the inter- and intra-layer potentials satisfy the moiré periodicity

$$\tilde{T}^{\eta,l}_{s_1 s_2}(\mathbf{r}) = \tilde{T}^{\eta,l}_{s_1 s_2}(\mathbf{r}+\mathbf{R}_M), \tag{S6.171}$$

$$\tilde{V}^{\eta}_{s_1 s_2}(\mathbf{r}) = \tilde{V}^{\eta}_{s_1 s_2}(\mathbf{r}+\mathbf{R}_M), \tag{S6.172}$$

for $\mathbf{R}_M \in \mathbb{Z}\mathbf{b}_{M_1} + \mathbb{Z}\mathbf{b}_{M_2}$. In contrast, the moiré potential corresponding to the $\hat{\psi}^\dagger_{\eta,s,l}(\mathbf{r})$ operators introduced in Eq. (S4.76) are only periodic up to a nontrivial phase, as shown in Eq. (S4.77). In the $\hat{\Psi}^\dagger_{\eta,s,l}(\mathbf{r})$ basis, the Hamiltonian $\mathcal{H}^{\mathrm{AA}}$ also has a coupling to an effective valley-dependent magnetic field. Within valley $\eta = 0$, the magnetic field is along the $\hat{\mathbf{y}}$ direction and gives rise to a flux

$$\int_{\mathbf{r}=\mathbf{0}}^{\mathbf{r}=\mathbf{a}_{M_1}} e\mathbf{A}^0_-(\mathbf{r})\cdot d\mathbf{r} + \int_{\mathbf{r}=\mathbf{a}_{M_1}}^{\mathbf{r}=\mathbf{0}} e\mathbf{A}^0_+(\mathbf{r})\cdot d\mathbf{r} = \pi, \tag{S6.173}$$

piercing each moiré unit cell from the side.

B. The C_{2z} symmetric limit

Another limit that we consider is termed the C_{2z} limit. In this limit, the AA- and AB-stacked moiré potentials have identical parameterizations and, within valley $\eta = 0$, the Hamiltonian is symmetric under C_{2x}, C_{2y}, and C_{2z} symmetries. Specifically, the C_{2z} limit is established whenever

$$w^{\mathrm{AA}}_1 = 0,\quad w^{\mathrm{AA}}_5 = 0,\quad w^{\mathrm{AA}}_6 = 0,\quad w'^{\mathrm{AA}}_1 = 0,\quad w'^{\mathrm{AA}}_2 = 0,\quad w'^{\mathrm{AA}}_7 = 0,\quad w'^{\mathrm{AA}}_8 = w'^{\mathrm{AA}}_9, \tag{S6.174}$$

$$w^{\mathrm{AB}}_1 = 0,\quad w'^{\mathrm{AB}}_1 = 0,\quad w'^{\mathrm{AB}}_2 = 0,\quad w'^{\mathrm{AB}}_3 = 0,\quad w'^{\mathrm{AB}}_5 = 0,\quad w'^{\mathrm{AB}}_9 = 0,\quad w'^{\mathrm{AB}}_{10} = 0. \tag{S6.175}$$

The representation matrices of the C_{2x} and C_{2y} were given in Section IV C 3. The action of the C_{2z} symmetry is given by Eqs. (S4.80) and (S4.83), where the momentum- and real-space representation matrices, respectively, read as

$$D(C_{2z}) = -is_z\sigma_0 \quad \text{and} \quad D_\psi(C_{2z}) = -i\begin{pmatrix}1&0&0\\0&1&0\\0&0&1\end{pmatrix} s_z\sigma_0. \tag{S6.176}$$

In the C_{2z}-symmetric limit, the parameterizations of the AA- and AB-stacked moiré potentials are identical with the following correspondence between their parameters

$$\begin{aligned} &w^{\mathrm{AA}}_2 \leftrightarrow w^{\mathrm{AB}}_2,\quad w^{\mathrm{AA}}_3 \leftrightarrow w^{\mathrm{AB}}_4,\quad w^{\mathrm{AA}}_4 \leftrightarrow w^{\mathrm{AB}}_3,\quad w'^{\mathrm{AA}}_3 \leftrightarrow w'^{\mathrm{AB}}_4,\quad w'^{\mathrm{AA}}_4 \leftrightarrow w'^{\mathrm{AB}}_6,\\ &w'^{\mathrm{AA}}_5 \leftrightarrow w'^{\mathrm{AB}}_7,\quad w'^{\mathrm{AA}}_6 \leftrightarrow w'^{\mathrm{AB}}_8,\quad w'^{\mathrm{AA}}_8 = w'^{\mathrm{AA}}_9 \leftrightarrow w'^{\mathrm{AB}}_{11},\quad w'^{\mathrm{AA}}_{10} \leftrightarrow w'^{\mathrm{AB}}_{12}. \end{aligned} \tag{S6.177}$$

C. The SU (2) symmetric limit

In the SU (2) symmetric limit, the moiré potential is spin-diagonal. This effectively enhances the U (1) × U (1) × U (1) valley-charge symmetry to a U (2) × U (2) × U (2) spin-valley-charge one. The 3 × 4 = 12 corresponding generators are given by

$$\hat{S}^{\eta}_a = \sum_{\mathbf{k},l,s,s'} \sum_{\mathbf{Q}\in\mathcal{Q}_{\eta+l}} [s_a]_{s_1,s_2}\, \hat{c}^\dagger_{\mathbf{k},\mathbf{Q},s_1,l}\hat{c}_{\mathbf{k},\mathbf{Q},s_2,l}, \quad \text{for} \quad a = 0,x,y,z \quad \text{and} \quad \eta = 0,1,2. \tag{S6.178}$$

Within the first moiré harmonic model, this symmetry is established whenever

$$w_3^{\mathrm{AA}}=0,\quad w_4^{\mathrm{AA}}=0,\quad w_5^{\mathrm{AA}}=0,\quad w_6^{\mathrm{AA}}=0,\quad w_6'^{\mathrm{AA}}=0,\quad w_8'^{\mathrm{AA}}=0,\quad w_9'^{\mathrm{AA}}=0, \tag{S6.179}$$

$$w_1^{\mathrm{AB}}=0,\quad w_3^{\mathrm{AB}}=0,\quad w_4^{\mathrm{AB}}=0,\quad w_5'^{\mathrm{AB}}=0,\quad w_8'^{\mathrm{AB}}=0,\quad w_{11}'^{\mathrm{AB}}=0, \tag{S6.180}$$

in the AA- and AB-stacked case, respectively. It is worth noting that for the AA-stacked case, the zero-twist constraints also imply the SU (2) symmetric limit. This contrasts with the AB-stacked arrangement (or even the AA-stacked case beyond the first moiré harmonic limit), where the zero-twist constraints *do not* necessarily imply that the system has SU (2) symmetry.

D. Additional symmetries in the two-center first monolayer harmonic model

In the two-center first monolayer harmonic model, the moiré potential from Eq. (S4.76) features continuous translation symmetry along one direction. Restricting ourselves to valley $\eta=0$ without loss of generality, the real-space moiré potential contains only interlayer terms and is given by

$$V^{0,\mathrm{AA}}_{s_1(-l);s_2 l}(\mathbf{r})=\sum_{n=\pm1}\left[\left(-inlw_1^{\mathrm{AA}}+w_2^{\mathrm{AA}}\right)s_0+n\left(w_4^{\mathrm{AA}}+w_6^{\mathrm{AA}}e^{inl\frac{\pi}{6}}\right)s_y+\left(-ilw_3^{\mathrm{AA}}+nw_5^{\mathrm{AA}}\right)s_z\right]_{s_1 s_2}e^{-inl\mathbf{q}_0\cdot\mathbf{r}}, \tag{S6.181}$$

$$V^{0,\mathrm{AB}}_{s_1(-l);s_2 l}(\mathbf{r})=\sum_{n=\pm1}\left[w_2^{\mathrm{AB}}s_0-ilw_1^{\mathrm{AB}}s_x+nw_3^{\mathrm{AB}}s_y-ilw_4^{\mathrm{AB}}s_z\right]_{s_1 s_2}e^{-inl\mathbf{q}_0\cdot\mathbf{r}}, \tag{S6.182}$$

while

$$V^{\eta}_{s_1 l;s_2 l}(\mathbf{r})=0. \tag{S6.183}$$

As a result, one finds that

$$V^{0}_{s_1 l_1;s_2 l_2}(x,y)=V^{0}_{s_1 l_1;s_2 l_2}(x,y+\delta y), \tag{S6.184}$$

making the moiré Hamiltonian from Eq. (S4.75) and valley $\eta=0$ invariant with respect to translations along the $\hat{\mathbf{y}}$ direction. In the two-center approximation, the system is quasi-one-dimensional within each valley, having continuous translation symmetry along the $C_{3z}^{\eta}\hat{\mathbf{y}}$ direction in valley η.

E. Additional symmetries of the simplified three-parameters models

In Section XI, we show that the *ab initio* band structures of AA- and AB-stacked $SnSe_2$ and ZrS_2 can be described by simplified models featuring only three parameters: w_1^{AA}, w_2^{AA}, and $w_3'^{\mathrm{AA}}$ (in the AA-stacked case), or w_2^{AB}, $w_3'^{\mathrm{AB}}$, and $w_4'^{\mathrm{AB}}$ (in the AB-stacked case). In this section, we examine the symmetries of these two simplified models and briefly discuss their implications on the band structure of the corresponding moiré Hamiltonian.

1. *The three-parameter AA-stacked model*

We begin by analyzing the three-parameter moiré Hamiltonian for the AA-stacked configuration. On the **Q**-lattice depicted in Fig. S15(c) corresponding to valley $\eta=0$, w_1^{AA} and w_2^{AA} describe the real and imaginary parts of the NN "hopping" along the $\hat{\mathbf{x}}$ direction, while $w_3'^{\mathrm{AA}}$ represents the NN "hopping" along the $\hat{\mathbf{y}}$ direction. This three-parameter model involves only interlayer moiré terms. It is already in both the zero-twist limit and the SU (2) limit, and therefore inherits all symmetries previously discussed in Sections VI A and VI C.

To uncover any additional symmetries, we cast the moiré potential in real space to find

$$V^{0,\mathrm{AA}}_{s_1(-l);s_2 l}(\mathbf{r})=\left[2\sqrt{\left(w_1^{\mathrm{AA}}\right)^2+\left(w_2^{\mathrm{AA}}\right)^2}\cos\left(|\mathbf{q}_0|x+\alpha\right)+2w_3'^{\mathrm{AA}}\cos\left(\sqrt{3}|\mathbf{q}_0|y\right)\right][s_0]_{s_1 s_2},$$
$$V^{0,\mathrm{AA}}_{s_1 l;s_2 l}(\mathbf{r})=0, \tag{S6.185}$$

where we have introduced a phase factor $0 \leq \alpha < 2\pi$, such that

$$e^{i\alpha} = \frac{i w_1^{\mathrm{AA}} + w_2^{\mathrm{AA}}}{\sqrt{\left(w_1^{\mathrm{AA}}\right)^2 + \left(w_2^{\mathrm{AA}}\right)^2}}. \tag{S6.186}$$

From Eq. (S6.185) it becomes evident that the system possesses an additional two-fold rotation symmetry, which we denote by $\tilde{C}_{2z}$, and whose center of rotation does *not* coincide with the unit cell origin (located at the intersection of the C_{2x} and C_{3z} rotation axes). The action of this symmetry on the moiré fermions in real space is given by

$$\tilde{C}_{2z}\hat{\psi}^\dagger_{\eta_1,s_1,l_1}(\mathbf{r})\,\tilde{C}_{2z}^{-1} = \sum_{\eta_2,s_2,p_2}\left[D_\psi\left(\tilde{C}_{2z}\right)\right]_{\eta_2 s_2 l_2;\eta_1 s_1 l_1}\hat{\psi}^\dagger_{\eta_2,s_2,l_2}\left(-\mathbf{r} + \frac{2\alpha\mathbf{q}_\eta}{|\mathbf{q}_\eta|^2}\right), \tag{S6.187}$$

with

$$D_\psi\left(\tilde{C}_{2z}\right) = i\begin{pmatrix}1&0&0\\0&1&0\\0&0&1\end{pmatrix} s_z\xi_0. \tag{S6.188}$$

Note that, within each valley η, the centers of rotation for the $\tilde{C}_{2z}$ symmetry operation are located at the generic points $\frac{\alpha\mathbf{q}_\eta}{|\mathbf{q}_\eta|^2}$, $\frac{\alpha\mathbf{q}_\eta}{|\mathbf{q}_\eta|^2} + \frac{\mathbf{a}_{M_1}}{2}$, $\frac{\alpha\mathbf{q}_\eta}{|\mathbf{q}_\eta|^2} + \frac{\mathbf{a}_{M_2}}{2}$, and $\frac{\alpha\mathbf{q}_\eta}{|\mathbf{q}_\eta|^2} + \frac{\mathbf{a}_{M_1}+\mathbf{a}_{M_2}}{2}$, none of which correspond to the unit cell origin (or any C_{3z}-symmetric Wyckoff position). Thus, while the symmetry group within a *single* valley is enhanced to a larger crystallographic space group, the *overall* symmetry group of the Hamiltonian (which incorporates all three valleys) does not *necessarily* correspond to a crystallographic space group. This occurs because the $\tilde{C}_{2z}$ rotation centers vary between the three valleys. However, when one of the four $\tilde{C}_{2z}$ rotation centers coincides with one of the three C_{3z} symmetric Wyckoff positions of the AA-stacked moiré Hamiltonian, the symmetry group of the *entire* moiré Hamiltonian is similarly enhanced by the $\tilde{C}_{2z}$ symmetry to form a *bona fide* crystallographic space group. The enhanced symmetry group of the full moiré Hamiltonian becomes a true crystallographic group whenever

$$\frac{\alpha\mathbf{q}_0}{|\mathbf{q}_0|^2} = \frac{k}{3}\left(2\mathbf{a}_{M_1} + \mathbf{a}_{M_2}\right) + \frac{m}{2}\mathbf{a}_{M_1} + \frac{n}{2}\left(\mathbf{a}_{M_1} + \mathbf{a}_{M_2}\right), \quad \text{for} \quad k = 0,1,2 \quad \text{and} \quad m,n \in \mathbb{Z}. \tag{S6.189}$$

Eq. (S6.189) holds only when $m = n$, leading to the condition

$$\frac{\alpha\mathbf{q}_0}{|\mathbf{q}_0|^2} = \frac{2k+3m}{6}\left(2\mathbf{a}_{M_1} + \mathbf{a}_{M_2}\right), \quad \text{for} \quad k = 0,1,2 \quad \text{and} \quad m \in \mathbb{Z}. \tag{S6.190}$$

which is satisfied for $\alpha = \frac{2\pi m}{6}$, where $m = 0, 1, \ldots, 5$. Finally, we note that the $\tilde{C}_{2z}$ symmetry, when combined with $\tilde{M}_z$, generates an effective momentum-space non-symmorphic inversion symmetry, denoted as $\tilde{C}_{2z}\tilde{M}_z$. This symmetry behaves similarly to the $\tilde{\mathcal{I}}$ symmetry described in Eq. (S6.139), with the key distinction being that the inversion center of $\tilde{C}_{2z}\tilde{M}_z$ is not at the unit cell origin, but at the point $\frac{\alpha\mathbf{q}_\eta}{|\mathbf{q}_\eta|^2}$.

We now turn to the topological characteristics of the three-parameter AA-stacked Hamiltonian. Due to the SU(2) symmetry present in each valley, the system can be effectively treated as spinless. Moreover, the presence of the $\tilde{C}_{2z}\mathcal{T}$ symmetry allows us to fix the gauge such that the wave function for each isolated spin-degenerate band becomes real. This gauge choice leads to a vanishing Berry curvature for each isolated spin-degenerate band. Consequently, we conclude that every isolated band in the system is topologically trivial within the three-parameter model. Because the *ab initio* band structure and the one approximated by the three-parameter model are adiabatically connected, we can also conclude that each *approximately* degenerate set of isolated bands of the AA-stacked moiré Hamiltonian are topologically trivial. As discussed at the end of Section VI A 2, these bands can be quasi-one-dimensional as a result of the $\tilde{M}_z$ symmetry.

Finally, for the topologically trivial bands within the three-parameter model, it is instructive to discuss the positions of the corresponding Wannier orbitals. In the even-odd basis introduced in Section VI A 2, the moiré potential from Eq. (S6.185) is given by

$$W^{0,p}_{s_1 s_2}(\mathbf{r}) = p\left[2\sqrt{\left(w_1^{\mathrm{AA}}\right)^2 + \left(w_2^{\mathrm{AA}}\right)^2}\cos\left(|\mathbf{q}_0|x + \alpha\right) + 2w_3'^{\mathrm{AA}}\cos\left(\sqrt{3}|\mathbf{q}_0|y\right)\right]\left[s_0\right]_{s_1 s_2}. \tag{S6.191}$$

For the mirror-even ($p = +1$) or mirror-odd ($p = -1$) sectors, the minimum of $W^{0,p}_{s_1 s_2}(\mathbf{r})$ occurs at $|\mathbf{q}_0|x + \alpha = (1+p)\frac{\pi}{2} + 2m_1\pi$ and $\sqrt{3}|\mathbf{q}_0|y = \left(1 + p\,\mathrm{sgn}\left(w_3'^{\mathrm{AA}}\right)\right)\frac{\pi}{2} + 2m_2\pi$, for $m_1, m_2 \in \mathbb{Z}$. Since the Wannier orbitals for each

mirror sector form at the potential minima, we conclude that all Wannier orbitals (*i.e.*, from both mirror sectors) in valley $\eta = 0$ are localized at

$$(x, y) \in \left\{ \frac{\pi}{|\mathbf{q}_0|} \left(\frac{1+p}{2} + 2m_1 - \frac{\alpha}{\pi}, \frac{\frac{1+p\,\mathrm{sgn}\left(w_3'^{\mathrm{AA}}\right)}{2} + 2m_2}{\sqrt{3}} \right) \middle| m_1, m_2 \in \mathbb{Z}, p = \pm 1 \right\}, \tag{S6.192}$$

or, equivalently, at

$$\begin{aligned} (x, y) &= \left(2m_1 - \frac{\alpha}{\pi}\right) \mathbf{a}_{M_1} + \left(m_1 + m_2 - \frac{\alpha}{2\pi} + \frac{1 - \mathrm{sgn}\left(w_3'^{\mathrm{AA}}\right)}{4}\right) \mathbf{a}_{M_2} \quad \text{and} \\ (x, y) &= \left(2m_1 + 1 - \frac{\alpha}{\pi}\right) \mathbf{a}_{M_1} + \left(m_1 + m_2 - \frac{\alpha}{2\pi} + \frac{3 + \mathrm{sgn}\left(w_3'^{\mathrm{AA}}\right)}{4}\right) \mathbf{a}_{M_2}, \quad \text{for} \quad m_1, m_2 \in \mathbb{Z}. \end{aligned} \tag{S6.193}$$

This indicates that the positions of the Wannier orbitals within the unit cell in valley η are given by

$$(x, y) = C_{3z}^{\eta} \left[-\frac{\alpha}{\pi} \mathbf{a}_{M_1} + \left(\frac{1 - \mathrm{sgn}\left(w_3'^{\mathrm{AA}}\right)}{4} - \frac{\alpha}{2\pi} \right) \mathbf{a}_{M_2} \right], \quad \text{with} \quad \alpha = \arg\left(i w_1^{\mathrm{AA}} + w_2^{\mathrm{AA}}\right) \tag{S6.194}$$

and thus depend on the energetics of the system.

2. *The three-parameter AB-stacked model*

The simplified model for the AB-stacked moiré Hamiltonian is defined by three parameters: w_2^{AB}, $w_3'^{\mathrm{AB}}$, and $w_4'^{\mathrm{AB}}$, corresponding to the NN "hopping" in the $\hat{\mathbf{x}}$-direction, NN "hopping" in the $\hat{\mathbf{y}}$-direction, and next NN "hopping" in the $\hat{\mathbf{x}}$-direction, respectively, on the $\mathbf{Q}$-lattice shown in Fig. S15(c) for valley $\eta = 0$. In the AB-stacked configuration, the three-parameter model includes both interlayer and intralayer terms

$$\begin{aligned} V^{0,\mathrm{AB}}_{s_1(-l);s_2 l}(\mathbf{r}) &= \left[2w_2^{\mathrm{AB}} \cos\left(|\mathbf{q}_0| x\right) + 2w_4'^{\mathrm{AB}} \cos\left(\sqrt{3}|\mathbf{q}_0| y\right) \right] [s_0]_{s_1 s_2}, \\ V^{0,\mathrm{AB}}_{s_1 l;s_2 l}(\mathbf{r}) &= \left[2l w_3'^{\mathrm{AB}} \sin\left(2|\mathbf{q}_0| x\right) \right] [s_0]_{s_1 s_2}, \end{aligned} \tag{S6.195}$$

This model is already in both the zero-twist and SU (2) limits, thereby inheriting all the symmetries discussed earlier in Sections VI A and VI C.

To discuss the topological properties of this model, we can simplify it further by setting $w_3'^{\mathrm{AB}}$ to zero. This is justified because: (1) in the *ab initio* calculations, $w_3'^{\mathrm{AB}}$ is smaller than w_2^{AB} and $w_4'^{\mathrm{AB}}$ for both SnSe_2 and ZrS_2, and (2) reducing $w_3'^{\mathrm{AB}}$ to zero still keeps the first two sets of bands individually gapped. The resulting two-parameter AB-stacked Hamiltonian reaches the C_{2z} limit as outlined in Section VI B. Due to the SU (2) symmetry in each valley, the system can again be treated as effectively spinless. As with the three-parameter AA-stacked Hamiltonian in Section VI E 1, this two-parameter AB-stacked model also possesses $C_{2z}\mathcal{T}$ symmetry, which allows for a gauge choice where the wave functions of isolated spin-degenerate bands become real. This leads to vanishing Berry curvature for each isolated spin-degenerate band, confirming that all isolated bands in the system are topologically trivial. The two-parameter model is adiabatically connected to the *ab initio* band structure, meaning that each approximately degenerate set of isolated bands in the AB-stacked moiré Hamiltonian is topologically trivial.

Furthermore, because the simplified two-parameter AB-stacked model retains both C_{2z} symmetry (from the C_{2z} limit) and $\tilde{\mathcal{I}}$ symmetry (from the zero-twist limit), it also exhibits $\tilde{M}_z = \tilde{\mathcal{I}} C_{2z}$ symmetry. Consequently, depending on the orbital shape, each gapped spin-degenerate topologically trivial band can be quasi-one-dimensional, as discussed in Section VI A 2, similar to the AA-stacked case. Moreover, we note that the two-parameter AB-stacked model can be mapped to the three-parameter AA-stacked model from Section VI E 1 by setting $w_1^{\mathrm{AA}} \to 0$, $w_2^{\mathrm{AA}} \to w_2^{\mathrm{AB}}$, and $w_3'^{\mathrm{AA}} \to w_4'^{\mathrm{AB}}$ in the latter. As such, for the trivial bands of the AB-stacked two-parameter model, the Wannier orbitals will be located at

$$(x, y) = C_{3z}^{\eta} \left[\left(\frac{\mathrm{sgn}\left(w_2^{\mathrm{AB}}\right) - \mathrm{sgn}\left(w_4'^{\mathrm{AB}}\right)}{4} \right) \mathbf{a}_{M_2} \right], \tag{S6.196}$$

in valley η.

Model or limit	Stacking	Zero-parameters	Symmetries			Crystallographic space group		Valley $\mathrm{U}(N)$
			$\tilde{M}_z$	$\tilde{\mathcal{I}}$	$C_{2z}/\tilde{C}_{2z}$	Valley η	All valleys	
First moiré harmonic	AA	None	✗	✗	✗	$P21'$ (SSG 3.2)	$P3121'$ (SSG 149.22)	$\mathrm{U}(1)$
	AB	None	✗	✗	✗		$P3211'$ (SSG 150.26)	
Zero-twist	AA	w_i^{AA} for $3 \leq i \leq 6$, $w_i'^{\mathrm{AA}}$ for $6 \leq i \leq 10$	✓	✗	✗	$Pmm21'$ (SSG 25.58)	$P\bar{6}m21'$ (SSG 187.210)	$\mathrm{U}(2)$
	AB	w_i^{AB} for $3 \leq i \leq 4$, $w_i'^{\mathrm{AB}}$ for $8 \leq i \leq 12$	✗	✓	✗	$P2/m1'$ (SSG 10.43)	$P\bar{3}m11'$ (SSG 164.86)	$\mathrm{U}(1)$
C_{2z} limit	AA	$w_1^{\mathrm{AA}}, w_5^{\mathrm{AA}}, w_6^{\mathrm{AA}}, w_1'^{\mathrm{AA}}, w_2'^{\mathrm{AA}}, w_7'^{\mathrm{AA}}, w_8'^{\mathrm{AA}} - w_9'^{\mathrm{AA}}$	✗	✗	C_{2z}	$P2221'$ (SSG 16.2)	$P6221'$ (SSG 177.150)	$\mathrm{U}(1)$
	AB	$w_1^{\mathrm{AB}}, w_1'^{\mathrm{AB}}, w_2'^{\mathrm{AB}}, w_3'^{\mathrm{AB}}, w_5'^{\mathrm{AB}}, w_9'^{\mathrm{AB}}, w_{10}'^{\mathrm{AB}}$						
$\mathrm{SU}(2)$ limit	AA	$w_3^{\mathrm{AA}}, w_4^{\mathrm{AA}}, w_5^{\mathrm{AA}}, w_6^{\mathrm{AA}}, w_6'^{\mathrm{AA}}, w_8'^{\mathrm{AA}}, w_9'^{\mathrm{AA}}$	✗	✗	✗	$P21'$ (SSG 3.2)	$P3121'$ (SSG 149.22)	$\mathrm{U}(2)$
	AB	$w_1^{\mathrm{AB}}, w_3^{\mathrm{AB}}, w_4^{\mathrm{AB}}, w_5'^{\mathrm{AB}}, w_8'^{\mathrm{AB}}, w_{11}'^{\mathrm{AB}}$					$P3211'$ (SSG 150.26)	
Two-center first monolayer harmonic	AA	$w_i'^{\mathrm{AA}}$ for $1 \leq i \leq 10$	✗	✗	✗	$P21'$ (SSG 3.2) Continuous translation along $C_{3z}^{\eta}\hat{\mathbf{y}}$	$P3121'$ (SSG 149.22)	$\mathrm{U}(1)$
	AB	$w_i'^{\mathrm{AA}}$ for $1 \leq i \leq 12$	✗	✗	✗		$P3211'$ (SSG 150.26)	
Three-parameter	AA	All *except* w_1^{AA}, w_2^{AA}, and $w_3'^{\mathrm{AA}}$	✓	✗	$\tilde{C}_{2z}$	$Cmmm1'$ (SSG 65.482)	$P6/mmm1'$ (SSG 191.234)	$\mathrm{U}(2)$
	AB	All *except* w_2^{AB}, $w_3'^{\mathrm{AB}}$, and $w_4'^{\mathrm{AB}}$	✗	✓	✗	$P2/m1'$ (SSG 10.43)	$P\bar{3}m11'$ (SSG 164.86)	
Two-parameter	AB	All *except* w_2^{AB} and $w_4'^{\mathrm{AB}}$	✓	✓	C_{2z}	$Cmmm1'$ (SSG 65.482)	$P6/mmm1'$ (SSG 191.234)	$\mathrm{U}(2)$

TABLE S3. Summary of the additional symmetries present in the first moiré harmonic model under various limits for both AA- and AB-stacking configurations. Each limit specifies the parameters set to zero, highlights any effective symmetries with momentum-space non-symmorphic action (*i.e.*, $\tilde{M}_z$ and $\tilde{\mathcal{I}}$), and indicates the presence of two-fold rotation symmetries perpendicular to the heterostructure's plane, such as C_{2z} or the effective $\tilde{C}_{2z}$. The corresponding crystallographic space groups are listed assuming the momentum-space non-symmorphic symmetries act conventionally (*i.e.*, with symmorphic momentum-space actions). For the three-parameter AA-stacked model, the space group for the full model is provided when $\alpha = \frac{\pi m}{3}$, for $m = 0, 1, \ldots, 5$. Lastly, the continuous symmetry group in each valley is also indicated.

F. Summary of symmetries in different limits

This section summarizes the symmetries of the first moiré harmonic model across different limits for AA- and AB-stacking configurations, as outlined in Table S3. Notably, the low-energy *ab initio* spectra of both $SnSe_2$ and ZrS_2 are well-approximated by simplified three-parameter and two-parameter models for the AA- and AB-stacked cases, respectively. These models exhibit enhanced symmetries, including effective symmetries acting non-symmorphically in momentum space. The table compares the various limits, listing the parameters set to zero, the presence of effective symmetries, and the relevant crystallographic space groups. These space groups are determined by assuming that the symmetries with momentum-space non-symmorphic actions act conventionally (*i.e.*, symmorphically).

VII. DIRECT GENERAL DERIVATION OF THE MOIRÉ POTENTIAL INCLUDING GRADIENT TERMS

This section further generalizes the results of the previous Section V by considering gradient terms in the moiré potential. We begin by writing down the most general form of the moiré Hamiltonian, which includes the aforementioned gradient terms. We then show how the form of the latter can be efficiently parameterized and constrained by the exact symmetries of the $\theta \neq 0$ heterostructure, as well as by the *approximate* symmetries of the $\theta = 0$ heterostructure.

A. General form of the moiré Hamiltonian with gradient terms

Following Ref. [5], we write down the most general expression for the single-particle moiré Hamiltonian

$$\mathcal{H} = \frac{1}{2} \sum_{\eta} \sum_{n_x, n_y} \sum_{\substack{s_1, l_1 \\ s_2, l_2}} \int \mathrm{d}^2 r \, t^{\eta, n_x, n_y}_{s_1 l_1; s_2 l_2} (\mathbf{r}) \left[i^{n_x + n_y} \left(\partial_x^{n_x} \partial_y^{n_y} \hat{\psi}^{\dagger}_{\eta, s_1, l_1} (\mathbf{r}) \right) \hat{\psi}_{\eta, s_2, l_2} (\mathbf{r}) \right.$$
$$\left. + (-i)^{n_x + n_y} \hat{\psi}^{\dagger}_{\eta, s_1, l_1} (\mathbf{r}) \, \partial_x^{n_x} \partial_y^{n_y} \hat{\psi}_{\eta, s_2, l_2} (\mathbf{r}) \right], \tag{S7.197}$$

which contains terms proportional to arbitrary gradients of the low-energy field operators introduced in Eq. (S4.73). In Eq. (S7.197), $t^{\eta,n_x,n_y}_{s_1 l_1;s_2 l_2}(\mathbf{r})$ denotes the generalized moiré potential and obeys the following Hermiticity property

$$t^{\eta,n_x,n_y}_{s_1 l_1;s_2 l_2}(\mathbf{r}) = \left(t^{\eta,n_x,n_y}_{s_2 l_2;s_1 l_1}(\mathbf{r})\right)^*. \tag{S7.198}$$

As a result, the integrand of Eq. (S7.197) (and not just the Hamiltonian as a whole) is a Hermitian operator. We have also implicitly incorporated the intralayer "kinetic" part into the function $t^{\eta,n_x,n_y}_{s_1 l_1;s_2 l_2}(\mathbf{r})$, as the distinction between the conventional "kinetic" and moiré potential fades away in the general gradient expansion case. Note that in Eq. (S7.197), without loss of generality, only terms where all derivatives act on *either* the creation *or* the annihilation operators need to be considered, as we will prove below.

To see that Eq. (S7.197) indeed represents *the most general form* of the moiré Hamiltonian, we first notice that any term containing spatial derivatives acting on the fermionic fields has the form

$$\begin{aligned}
&\sum_{\eta}\sum_{\substack{s_1,l_1\\s_2,l_2}}\int \mathrm{d}^2 r\, t'^{\eta}_{s_1 l_1;s_2 l_2}(\mathbf{r})\, i^{n_x+n_y}(-i)^{n'_x+n'_y}\left(\partial_x^{n_x}\partial_y^{n_y}\hat{\psi}^\dagger_{\eta,s_1,l_1}(\mathbf{r})\right)\partial_x^{n'_x}\partial_y^{n'_y}\hat{\psi}_{\eta,s_2,l_2}(\mathbf{r})\\
=&\sum_{\eta}\sum_{\substack{s_1,l_1\\s_2,l_2}}\int \mathrm{d}^2 r\, i^{n_x+n'_x+n_y+n'_y}t'^{\eta}_{s_1 l_1;s_2 l_2}(\mathbf{r})\left(\partial_x^{n_x+n'_x}\partial_y^{n_y+n'_y}\hat{\psi}^\dagger_{\eta,s_1,l_1}(\mathbf{r})\right)\hat{\psi}_{\eta,s_2,l_2}(\mathbf{r})\\
&+\left[\text{terms proportional to gradients of } t'^{\eta}_{s_1 l_1;s_2 l_2}(\mathbf{r})\right]\dots\\
=&\frac{1}{2}\sum_{\eta}\sum_{\substack{s_1,l_1\\s_2,l_2}}\int \mathrm{d}^2 r\, t'^{\eta}_{s_1 l_1;s_2 l_2}(\mathbf{r})\left[i^{n_x+n'_x+n_y+n'_y}\left(\partial_x^{n_x+n'_x}\partial_y^{n_y+n'_y}\hat{\psi}^\dagger_{\eta,s_1,l_1}(\mathbf{r})\right)\hat{\psi}_{\eta,s_2,l_2}(\mathbf{r})\right.\\
&\left.+(-i)^{n_x+n'_x+n_y+n'_y}\hat{\psi}^\dagger_{\eta,s_1,l_1}(\mathbf{r})\,\partial_x^{n_x+n'_x}\partial_y^{n_y+n'_y}\hat{\psi}_{\eta,s_2,l_2}(\mathbf{r})\right]\\
&+\left[\text{terms proportional to gradients of } t'^{\eta}_{s_1 l_1;s_2 l_2}(\mathbf{r})\right]\dots,
\end{aligned}\tag{S7.199}$$

where $n_x, n_y, n'_x, n'_y \in \mathbb{N}$, $t'^{\eta}_{s_1 l_1;s_2 l_2}(\mathbf{r})$ is some moiré potential and the ellipses denote terms in which the field operators have less than $n_x + n'_x$ x-derivatives and less than $n_y + n'_y$ y-derivatives. We can therefore conclude that any term appearing in the Hamiltonian featuring spatial derivatives of the fermionic field can be written as the sum between a term where all derivatives act on the creation field and another term where the derivatives act on the annihilation field, in addition to other terms containing strictly fewer derivatives acting on the fermionic fields. Using this observation, we can straightforwardly prove (through induction) that any term having the form of the first line of Eq. (S7.199) can be written as a sum of pairs of the form of the last equality of Eq. (S7.199): each pair is a sum between one term in which all spatial derivatives act on the creation field and a term where all spatial derivatives act on the annihilation field; each pair contains an equal or smaller number of spatial derivatives as the original term

$$\begin{aligned}
&\sum_{\eta}\sum_{\substack{s_1,l_1\\s_2,l_2}}\int \mathrm{d}^2 r\, t'^{\eta}_{s_1 l_1;s_2 l_2}(\mathbf{r})\, i^{n_x+n_y}(-i)^{n'_x+n'_y}\left(\partial_x^{n_x}\partial_y^{n_y}\hat{\psi}^\dagger_{\eta,s_1,l_1}(\mathbf{r})\right)\partial_x^{n'_x}\partial_y^{n'_y}\hat{\psi}_{\eta,s_2,l_2}(\mathbf{r})\\
=&\frac{1}{2}\sum_{\substack{m_x\le n_x+n'_x\\m_y\le n_y+n'_y}}\sum_{\eta}\sum_{\substack{s_1,l_1\\s_2,l_2}}\int \mathrm{d}^2 r\, t^{\eta,m_x,m_y}_{s_1 l_1;s_2 l_2}(\mathbf{r})\left[i^{m_x+m_y}\left(\partial_x^{m_x}\partial_y^{m_y}\hat{\psi}^\dagger_{\eta,s_1,l_1}(\mathbf{r})\right)\hat{\psi}_{\eta,s_2,l_2}(\mathbf{r})\right.\\
&\left.+(-i)^{m_x+m_y}\hat{\psi}^\dagger_{\eta,s_1,l_1}(\mathbf{r})\,\partial_x^{m_x}\partial_y^{m_y}\hat{\psi}_{\eta,s_2,l_2}(\mathbf{r})\right].
\end{aligned}\tag{S7.200}$$

To prove Eq. (S7.200), we note that Eq. (S7.200) is trivially valid for $n_x+n'_x = n_y+n'_y = 0$. One then assumes it holds for some $n_x + n'_x = M_x$ and $n_y + n'_y = M_y$ (where $M_x, M_y \in \mathbb{N}$). Because the ellipses in Eq. (S7.199) denote terms with fewer spatial derivatives acting on the fermionic fields, it follows that if Eq. (S7.200) holds for $n_x + n'_x = M_x$ and $n_y + n'_y = M_y$, it should also hold for $n_x + n'_x = M_x + 1$ and $n_y + n'_y = M_y$ or $n_x + n'_x = M_x$ and $n_y + n'_y = M_y + 1$, thus completing the inductive proof. In what follows, we will employ Eq. (S7.197) as the most general form of the moiré potential.

As in the case where the moiré potential contains no gradient terms, the Hamiltonian from Eq. (S7.197) is diagonal in the valley subspace and translation-invariant at the moiré lattice scale. As a result of the latter property, the generalized moiré potential obeys

$$t^{\eta,n_x,n_y}_{s_1 l_1;s_2 l_2}(\mathbf{r}+\mathbf{R}_M) = t^{\eta,n_x,n_y}_{s_1 l_1;s_2 l_2}(\mathbf{r})\, e^{i\left(\mathbf{q}_{\eta+l_2}-\mathbf{q}_{\eta+l_1}\right)\cdot\mathbf{R}_M}, \quad \text{for any} \quad \mathbf{R}_M \in \mathbb{Z}\mathbf{a}_{M_1}+\mathbb{Z}\mathbf{a}_{M_2}, \tag{S7.201}$$

which is identical in form to Eq. (S4.77). This follows from Eq. (S4.73) which requires that

$$\left(\partial_x^{n_x}\partial_y^{n_y}\hat{\psi}^\dagger_{\eta,s_1,l_1}\left(\mathbf{r}+\mathbf{R}_M\right)\right)\left(\partial_x^{m_x}\partial_y^{m_y}\hat{\psi}_{\eta,s_2,l_2}\left(\mathbf{r}+\mathbf{R}_M\right)\right) \\ =\left(\partial_x^{n_x}\partial_y^{n_y}\hat{\psi}^\dagger_{\eta,s_1,l_1}\left(\mathbf{r}\right)\right)\left(\partial_x^{m_x}\partial_y^{m_y}\hat{\psi}_{\eta,s_2,l_2}\left(\mathbf{r}\right)\right)e^{-i\left(\mathbf{q}_{\eta+l_2}-\mathbf{q}_{\eta+l_1}\right)\cdot\mathbf{R}_M}, \tag{S7.202}$$

for any $n_x, n_y, m_x, m_y \in \mathbb{N}$ and $\mathbf{R}_M \in \mathbb{Z}\mathbf{a}_{M_1}+\mathbb{Z}\mathbf{a}_{M_2}$. In turn, Eq. (S7.201) enables us to express the generalized moiré potential as the following Fourier series

$$t^{\eta,n_x,n_y}_{s_1l_1;s_2l_2}\left(\mathbf{r}\right)=\sum_{\mathbf{G}\in\mathcal{Q}}\left[T^{n_x,n_y}_{\mathbf{q}_{\eta+l_1},\mathbf{q}_{\eta+l_2}+\mathbf{G}}\right]_{s_1l_1;s_2l_2}e^{i\left(\mathbf{q}_{\eta+l_2}+\mathbf{G}-\mathbf{q}_{\eta+l_1}\right)\cdot\mathbf{r}}, \tag{S7.203}$$

$$\left[T^{n_x,n_y}_{\mathbf{q}_{\eta+l_1},\mathbf{q}_{\eta+l_2}+\mathbf{G}}\right]_{s_1l_1;s_2l_2}=\frac{1}{\Omega}\int\mathrm{d}^2r\, t^{\eta,n_x,n_y}_{s_1l_1;s_2l_2}\left(\mathbf{r}\right)e^{-i\left(\mathbf{q}_{\eta+l_2}+\mathbf{G}-\mathbf{q}_{\eta+l_1}\right)\cdot\mathbf{r}}, \tag{S7.204}$$

where we have used a notation analogous to Eq. (S4.76), and cast the Hamiltonian from Eq. (S7.197) in momentum space

$$\mathcal{H}=\sum_{\substack{\mathbf{k},n_x,n_y\\ \mathbf{Q},\mathbf{Q}'\in\mathcal{Q}_{\mathrm{tot}}}}\sum_{\substack{s_1,l_1\\ s_2,l_2}}\frac{\left(k_x-Q_x\right)^{n_x}\left(k_y-Q_y\right)^{n_y}+\left(k_x-Q'_x\right)^{n_x}\left(k_y-Q'_y\right)^{n_y}}{2}\left[T^{n_x,n_y}_{\mathbf{Q},\mathbf{Q}'}\right]_{s_1l_1;s_2l_2}\hat{c}^\dagger_{\mathbf{k},\mathbf{Q},s_1,l_1}\hat{c}_{\mathbf{k},\mathbf{Q}',s_2,l_2}. \tag{S7.205}$$

In Eq. (S7.205) the momentum-space moiré potential matrix coupling *any* plane wave-vectors $\mathbf{Q},\mathbf{Q}'\in\mathcal{Q}_{\mathrm{tot}}$ is obtained from Eq. (S7.204) through

$$\left[T^{n_x,n_y}_{\mathbf{q}_{\eta+l_1}+\mathbf{G}_1,\mathbf{q}_{\eta+l_2}+\mathbf{G}_2}\right]_{s_1l_1;s_2l_2}=\left[T^{n_x,n_y}_{\mathbf{q}_{\eta+l_1},\mathbf{q}_{\eta+l_2}+\mathbf{G}_2-\mathbf{G}_1}\right]_{s_1l_1;s_2l_2},\quad \text{for any}\quad \mathbf{G}_1,\mathbf{G}_2\in\mathcal{Q}_0. \tag{S7.206}$$

B. Restricting the generalized moiré potential using the exact $\theta \neq 0$ symmetries of the heterostructure

The form of the generalized moiré potential matrix $T^{n_x,n_y}_{\mathbf{Q},\mathbf{Q}'}$ can be constrained using the exact symmetries of the twisted heterostructure in the $\theta \neq 0$ case. To start with, the moiré Hamiltonian from Eq. (S7.205) is Hermitian, which immediately implies that

$$\left[T^{n_x,n_y}_{\mathbf{Q},\mathbf{Q}'}\right]_{s_1l_1;s_2l_2}=\left[T^{n_x,n_y}_{\mathbf{Q}',\mathbf{Q}}\right]^*_{s_2l_2;s_1l_1}. \tag{S7.207}$$

At the same time, moiré periodicity requires that

$$T^{n_x,n_y}_{\mathbf{Q},\mathbf{Q}'}=T^{n_x,n_y}_{\mathbf{Q}+\mathbf{G},\mathbf{Q}'+\mathbf{G}},\quad \text{for}\quad \mathbf{G}\in\mathcal{Q}, \tag{S7.208}$$

which is equivalent to Eq. (S7.206). Additionally, the $\mathrm{U}\left(1\right)\times\mathrm{U}\left(1\right)\times\mathrm{U}\left(1\right)$ valley-charge symmetry will require that the moiré potential always satisfies the analogue of Eq. (S4.72)

$$\left[T^{n_x,n_y}_{\mathbf{Q}',\mathbf{Q}}\right]_{s_1l_1;s_2l_2}=0,\quad \text{for}\quad \mathbf{Q}\in\mathcal{Q}_{\eta_1+l_1}\quad \text{and}\quad \mathbf{Q}'\in\mathcal{Q}_{\eta_2+l_2},\quad \text{with}\quad \eta_1\neq\eta_2. \tag{S7.209}$$

To obtain the constraints arising from crystalline symmetries, we note that for any symmetry g and momentum $\mathbf{k}$

$$\left(\left[g\mathbf{k}\right]_x\right)^{n_x}\left(\left[g\mathbf{k}\right]_y\right)^{n_y}=\sum_{\substack{n'_x,n'_y\\ n'_x+n'_y=n_x+n_y}}U_{n'_x,n'_y;n_x,n_y}(g)k_x^{n'_x}k_y^{n'_y}, \tag{S7.210}$$

with $\left[g\mathbf{k}\right]_x$ denoting the x component of $g\mathbf{k}$. In other words, under a symmetry g a monomial in momentum components transforms into a polynomial function of momentum components, in which each term has the same total power in momentum as the original monomial. The corresponding matrix $U_{n_x,n_y;n'_x,n'_y}(g)$ is related to the totally symmetric representation of g and can be obtained by direct construction from the cartesian representation of g. As such, for every crystalline symmetry g, the generalized moiré potential will obey

$$\sum_{\substack{n_x,n_y\\ n_x+n_y=n'_x+n'_y}}U_{n'_x,n'_y;n_x,n_y}(g)T^{n_x,n_y}_{g\mathbf{Q},g\mathbf{Q}'}=D(g)\left(T^{n'_x,n'_y}_{\mathbf{Q},\mathbf{Q}'}\right)^{(*)}D^\dagger(g), \tag{S7.211}$$

where $^{(*)}$ indicates that a complex conjugation should be taken in the cases when g is antiunitary. Taken together, Eqs. (S7.207) and (S7.211) constitute a series of homogeneous linear equations for the components of $T^{n_x,n_y}_{\mathbf{Q},\mathbf{Q}'}$. By solving the resulting system of equations, one can obtain the independent components of generalized moiré potential, as well as its parameterization.

C. Restricting the generalized moiré potential using the approximate $\theta = 0$ symmetries of the heterostructure

In the limit of vanishing twist angle $\theta \to 0$, the generalized moiré potential can be additionally constrained by the symmetries of the untwisted heterostructure. We follow the same strategy that we used to constrain the moiré potential without gradient terms in Section V B. We start from the family of Hamiltonian $\mathcal{H}(\Delta\mathbf{R})$ introduced in Eq. (S5.96), but absorb the single-particle Hamiltonian of the single-layer material into the second term by a redefinition of the $S_{s_1 l_1; s_2 l_2}\left(\Delta\mathbf{R}, \mathbf{r}^{\Delta\mathbf{R}}_{l_2,\mathbf{R}_2} - \mathbf{r}^{\Delta\mathbf{R}}_{l_1,\mathbf{R}_1}\right)$ matrix, such that the Hamiltonian $\mathcal{H}(\Delta\mathbf{R})$ reads as

$$\mathcal{H}(\Delta\mathbf{R}) = \sum_{\substack{\mathbf{R}_1, l_1, s_1 \\ \mathbf{R}_2, l_2, s_2}} S_{s_1 l_1; s_2 l_2}\left(\Delta\mathbf{R}, \mathbf{r}^{\Delta\mathbf{R}}_{l_2,\mathbf{R}_2} - \mathbf{r}^{\Delta\mathbf{R}}_{l_1,\mathbf{R}_1}\right) \hat{a}^\dagger_{\mathbf{R}_1, s_1, l_1} \hat{a}_{\mathbf{R}_2, s_2, l_2}. \tag{S7.212}$$

Such a notation treats the single-particle Hamiltonian of the monolayer material, as well as the interlayer contribution on equal footing.

1. *Moiré Hamiltonian in the local-stacking approximation with gradient terms*

Making the same local-stacking approximation as in Section V B 2, we find that the moiré Hamiltonian of the twisted configuration is given by

$$\begin{aligned}
\mathcal{H} =& \frac{1}{\Omega_0} \sum_{\substack{\mathbf{g}_1, l_1, s_1, \eta_1 \\ \mathbf{g}_2, l_2, s_2, \eta_2}} \int \mathrm{d}^2x\, \mathrm{d}^2y\, S_{s_1 l_1; s_2 l_2}\left(\delta\mathbf{R}(\mathbf{x}), \mathbf{y}\right) e^{-i\mathcal{R}_{\theta,l_1}\left(\mathbf{g}_1 + C^{\eta_1}_{3z}\mathbf{K}_M\right)\cdot\left(\mathbf{x} - \frac{\mathbf{y}}{2}\right)} e^{i\mathcal{R}_{\theta,l_2}\left(\mathbf{g}_2 + C^{\eta_2}_{3z}\mathbf{K}_M\right)\cdot\left(\mathbf{x} + \frac{\mathbf{y}}{2}\right)} \\
& \times \hat{\psi}^\dagger_{\eta_1, s_1, l_1}\left(\mathbf{x} - \frac{\mathbf{y}}{2}\right) \hat{\psi}_{\eta_2, s_2, l_2}\left(\mathbf{x} + \frac{\mathbf{y}}{2}\right) \\
\approx& \frac{1}{\Omega_0} \sum_{\substack{\mathbf{g}, \eta \\ l_1, s_1, l_2, s_2}} \int \mathrm{d}^2x\, \mathrm{d}^2y\, S_{s_1 l_1; s_2 l_2}\left(\delta\mathbf{R}(\mathbf{x}), \mathbf{y}\right) e^{-i\mathcal{R}_{\theta,l_1}\left(\mathbf{g} + C^{\eta}_{3z}\mathbf{K}_M\right)\cdot\left(\mathbf{x} - \frac{\mathbf{y}}{2}\right)} e^{i\mathcal{R}_{\theta,l_2}\left(\mathbf{g} + C^{\eta}_{3z}\mathbf{K}_M\right)\cdot\left(\mathbf{x} + \frac{\mathbf{y}}{2}\right)} \\
& \times \hat{\psi}^\dagger_{\eta, s_1, l_1}\left(\mathbf{x} - \frac{\mathbf{y}}{2}\right) \hat{\psi}_{\eta, s_2, l_2}\left(\mathbf{x} + \frac{\mathbf{y}}{2}\right) \\
=& \frac{1}{2\Omega_0} \sum_{\substack{\mathbf{g}, \eta \\ l_1, s_1, l_2, s_2}} \int \mathrm{d}^2x\, \mathrm{d}^2y\, e^{i\left(\mathcal{R}_{\theta,l_2} - \mathcal{R}_{\theta,l_1}\right)\left(\mathbf{g} + C^{\eta}_{3z}\mathbf{K}_M\right)\cdot\mathbf{x}} \\
& \times \left(S_{s_1 l_1; s_2 l_2}\left(\delta\mathbf{R}\left(\mathbf{x} - \frac{\mathbf{y}}{2}\right), \mathbf{y}\right) \hat{\psi}^\dagger_{\eta, s_1, l_1}\left(\mathbf{x} - \mathbf{y}\right) \hat{\psi}_{\eta, s_2, l_2}\left(\mathbf{x}\right) e^{i\mathcal{R}_{\theta,l_1}\left(\mathbf{g} + C^{\eta}_{3z}\mathbf{K}_M\right)\cdot\mathbf{y}}\right. \\
& \left. + S_{s_1 l_1; s_2 l_2}\left(\delta\mathbf{R}\left(\mathbf{x} + \frac{\mathbf{y}}{2}\right), \mathbf{y}\right) \hat{\psi}^\dagger_{\eta, s_1, l_1}\left(\mathbf{x}\right) \hat{\psi}_{\eta, s_2, l_2}\left(\mathbf{x} + \mathbf{y}\right) e^{i\mathcal{R}_{\theta,l_2}\left(\mathbf{g} + C^{\eta}_{3z}\mathbf{K}_M\right)\cdot\mathbf{y}}\right).
\end{aligned} \tag{S7.213}$$

The first two lines of Eq. (S7.213) are identical to the first two lines of Eq. (S5.115), with the exception that the single-layer contribution has been incorporated into the $S_{s_1 l_1; s_2 l_2}\left(\delta\mathbf{R}(\mathbf{x}), \mathbf{y}\right)$ matrix. From the first two lines to the following two, we used the fact that $S_{s_1 l_1; s_2 l_2}\left(\delta\mathbf{R}(\mathbf{x}), \mathbf{y}\right)$ is only nonvanishing for $|\mathbf{y}| \lesssim |\mathbf{a}_1|$, and varies slowly in $\mathbf{x}$ on the monolayer lattice scale, just as the low-energy fermionic fields. As a result, only the term for which the complex exponential factor does not oscillate strongly on the single-layer lattice scale will survive. Compared to Section V B 2, we will not make any further assumption about the length-scale over which the low-energy fermion operators vary.

We will now focus on simplifying each term of Eq. (S7.213). Because both terms are similar in form, we can simplify only the second one, with the first one following in the same way. To do so, we begin by noting that the fermion field operators can be expanded as

$$\hat{\psi}_{\eta,s,l}\left(\mathbf{x} + \mathbf{y}\right) = \sum_{n=0}^{\infty} \frac{1}{n!} \left(\mathbf{y} \cdot \boldsymbol{\nabla}\right)^n \hat{\psi}_{\eta,s,l}\left(\mathbf{x}\right)$$

$$= \sum_{n_x,n_y=0}^{\infty} \frac{\binom{n_x+n_y}{n_x} y_x^{n_x} y_y^{n_y}}{(n_x+n_y)!} \partial_x^{n_x} \partial_y^{n_y} \hat{\psi}_{\eta,s,l}(\mathbf{x})$$

$$= \sum_{n_x,n_y=0}^{\infty} \frac{y_x^{n_x} y_y^{n_y}}{n_x! n_y!} \partial_x^{n_x} \partial_y^{n_y} \hat{\psi}_{\eta,s,l}(\mathbf{x}) . \tag{S7.214}$$

In turn, this allows us to rewrite the second term of Eq. (S7.213) as

$$\frac{1}{2\Omega_0} \sum_{\substack{\mathbf{g},\eta \\ l_1,s_1,l_2,s_2}} \int \mathrm{d}^2x \, \mathrm{d}^2y \, e^{i\left(\mathcal{R}_{\theta,l_2}-\mathcal{R}_{\theta,l_1}\right)\left(\mathbf{g}+C_{3z}^{\eta}\mathbf{K}_M\right)\cdot\mathbf{x}} e^{i\mathcal{R}_{\theta,l_2}\left(\mathbf{g}+C_{3z}^{\eta}\mathbf{K}_M\right)\cdot\mathbf{y}}$$
$$\times S_{s_1l_1;s_2l_2}\left(\delta\mathbf{R}\left(\mathbf{x}+\frac{\mathbf{y}}{2}\right),\mathbf{y}\right) \hat{\psi}^\dagger_{\eta,s_1,l_1}(\mathbf{x}) \hat{\psi}_{\eta,s_2,l_2}(\mathbf{x}+\mathbf{y}) =$$
$$=\frac{1}{2\Omega_0} \sum_{\substack{\mathbf{g},\eta \\ l_1,s_1,l_2,s_2}} \int \mathrm{d}^2x \, \mathrm{d}^2y \, e^{i\left(\mathcal{R}_{\theta,l_2}-\mathcal{R}_{\theta,l_1}\right)\left(\mathbf{g}+C_{3z}^{\eta}\mathbf{K}_M\right)\cdot\mathbf{x}} e^{i\mathcal{R}_{\theta,l_2}\left(\mathbf{g}+C_{3z}^{\eta}\mathbf{K}_M\right)\cdot\mathbf{y}}$$
$$\times \frac{y_x^{n_x} y_y^{n_y}}{n_x! n_y!} S_{s_1l_1;s_2l_2}\left(\delta\mathbf{R}\left(\mathbf{x}+\frac{\mathbf{y}}{2}\right),\mathbf{y}\right) \hat{\psi}^\dagger_{\eta,s_1,l_1}(\mathbf{x}) \partial_x^{n_x} \partial_y^{n_y} \hat{\psi}_{\eta,s_2,l_2}(\mathbf{x})$$
$$=\frac{1}{2N\Omega_0} \sum_{\substack{\mathbf{g},\mathbf{g}',\mathbf{k},\eta \\ l_1,s_1,l_2,s_2}} \int \mathrm{d}^2x \, \mathrm{d}^2y \, e^{i\left(\mathcal{R}_{\theta,l_2}-\mathcal{R}_{\theta,l_1}\right)\left(\mathbf{g}+C_{3z}^{\eta}\mathbf{K}_M\right)\cdot\mathbf{x}} e^{i\mathcal{R}_{\theta,l_2}\left(\mathbf{g}+C_{3z}^{\eta}\mathbf{K}_M\right)\cdot\mathbf{y}} e^{-i\mathbf{g}'\cdot\delta\mathbf{R}\left(\mathbf{x}+\frac{\mathbf{y}}{2}\right)} e^{-i\mathbf{k}\cdot\mathbf{y}}$$
$$\times \frac{y_x^{n_x} y_y^{n_y}}{n_x! n_y!} S_{s_1l_1;s_2l_2}\left(\mathbf{g}',\mathbf{k}\right) \hat{\psi}^\dagger_{\eta,s_1,l_1}(\mathbf{x}) \partial_x^{n_x} \partial_y^{n_y} \hat{\psi}_{\eta,s_2,l_2}(\mathbf{x})$$
$$=\frac{1}{2N\Omega_0} \sum_{\substack{\mathbf{g},\mathbf{g}',\mathbf{k},\eta \\ l_1,s_1,l_2,s_2}} \int \mathrm{d}^2x \, \mathrm{d}^2y \, e^{i\left(\mathcal{R}_{\theta,l_2}-\mathcal{R}_{\theta,l_1}\right)\left(\mathbf{g}+C_{3z}^{\eta}\mathbf{K}_M\right)\cdot\mathbf{x}} e^{i\mathcal{R}_{\theta,l_2}\left(\mathbf{g}+C_{3z}^{\eta}\mathbf{K}_M\right)\cdot\mathbf{y}} e^{-i\mathbf{g}'\cdot\delta\mathbf{R}\left(\mathbf{x}+\frac{\mathbf{y}}{2}\right)} e^{-i\mathbf{k}\cdot\mathbf{y}}$$
$$\times \frac{(-i)^{n_x+n_y} \partial_{k_x}^{n_x} \partial_{k_y}^{n_y}}{n_x! n_y!} S_{s_1l_1;s_2l_2}\left(\mathbf{g}',\mathbf{k}\right) \hat{\psi}^\dagger_{\eta,s_1,l_1}(\mathbf{x}) \partial_x^{n_x} \partial_y^{n_y} \hat{\psi}_{\eta,s_2,l_2}(\mathbf{x})$$
$$=\frac{1}{2N\Omega_0} \sum_{\substack{\mathbf{g},\mathbf{k},\eta \\ l_1,s_1,l_2,s_2}} \int \mathrm{d}^2x \, \mathrm{d}^2y \, e^{-i\frac{l_2-l_1}{2}\mathbf{q}_\eta\cdot\mathbf{x}} e^{i\mathcal{R}_{\theta,l_2}C_{3z}^{\eta}\mathbf{K}_M\cdot\mathbf{y}} e^{-i\mathbf{g}\cdot\delta\mathbf{R}(\mathbf{x})} e^{-2i\sin\left(\frac{\theta}{2}\right)(\mathbf{g}\times\hat{\mathbf{z}})\cdot\frac{\mathbf{y}}{2}} e^{-i\mathbf{k}\cdot\mathbf{y}}$$
$$\times \frac{(-i)^{n_x+n_y} \partial_{k_x}^{n_x} \partial_{k_y}^{n_y}}{n_x! n_y!} S_{s_1l_1;s_2l_2}\left(\mathbf{g},\mathbf{k}\right) \hat{\psi}^\dagger_{\eta,s_1,l_1}(\mathbf{x}) \partial_x^{n_x} \partial_y^{n_y} \hat{\psi}_{\eta,s_2,l_2}(\mathbf{x})$$
$$=\frac{1}{2} \sum_{\substack{\mathbf{g},\eta \\ l_1,s_1,l_2,s_2}} \int \mathrm{d}^2x \, e^{-i\frac{l_2-l_1}{2}\mathbf{q}_\eta\cdot\mathbf{x}} e^{-i\mathbf{g}\cdot\delta\mathbf{R}(\mathbf{x})} \hat{\psi}^\dagger_{\eta,s_1,l_1}(\mathbf{x}) (-i)^{n_x+n_y} \partial_x^{n_x} \partial_y^{n_y} \hat{\psi}_{\eta,s_2,l_2}(\mathbf{x})$$
$$\times \left. \frac{\partial_{k_x}^{n_x} \partial_{k_y}^{n_y}}{n_x! n_y!} S_{s_1l_1;s_2l_2}\left(\mathbf{g},\mathbf{k}\right) \right|_{\mathbf{k}=\mathcal{R}_{\theta,l_2}C_{3z}^{\eta}\mathbf{K}_M-\sin\left(\frac{\theta}{2}\right)(\mathbf{g}\times\hat{\mathbf{z}})} , \tag{S7.215}$$

where we have also used the Fourier representation from Eq. (S5.108). Similarly, for the first term of Eq. (S7.213), we find that

$$\frac{1}{2\Omega_0} \sum_{\substack{\mathbf{g},\eta \\ l_1,s_1,l_2,s_2}} \int \mathrm{d}^2x \, \mathrm{d}^2y \, e^{i\left(\mathcal{R}_{\theta,l_2}-\mathcal{R}_{\theta,l_1}\right)\left(\mathbf{g}+C_{3z}^{\eta}\mathbf{K}_M\right)\cdot\mathbf{x}} e^{i\mathcal{R}_{\theta,l_1}\left(\mathbf{g}+C_{3z}^{\eta}\mathbf{K}_M\right)\cdot\mathbf{y}}$$
$$\times S_{s_1l_1;s_2l_2}\left(\delta\mathbf{R}\left(\mathbf{x}-\frac{\mathbf{y}}{2}\right),\mathbf{y}\right) \hat{\psi}^\dagger_{\eta,s_1,l_1}(\mathbf{x}-\mathbf{y}) \hat{\psi}_{\eta,s_2,l_2}(\mathbf{x}) =$$
$$=\frac{1}{2} \sum_{\substack{\mathbf{g},\eta \\ l_1,s_1,l_2,s_2}} \int \mathrm{d}^2x \, e^{-i\frac{l_2-l_1}{2}\mathbf{q}_\eta\cdot\mathbf{x}} e^{-i\mathbf{g}\cdot\delta\mathbf{R}(\mathbf{x})} \left(i^{n_x+n_y} \partial_x^{n_x} \partial_y^{n_y} \hat{\psi}^\dagger_{\eta,s_1,l_1}(\mathbf{x})\right) \hat{\psi}_{\eta,s_2,l_2}(\mathbf{x})$$
$$\times \left. \frac{\partial_{k_x}^{n_x} \partial_{k_y}^{n_y}}{n_x! n_y!} S_{s_1l_1;s_2l_2}\left(\mathbf{g},\mathbf{k}\right) \right|_{\mathbf{k}=\mathcal{R}_{\theta,l_1}C_{3z}^{\eta}\mathbf{K}_M+\sin\left(\frac{\theta}{2}\right)(\mathbf{g}\times\hat{\mathbf{z}})} . \tag{S7.216}$$

Putting together Eqs. (S7.215) and (S7.216), we find that in the local-stacking approximation, the moiré Hamiltonian with gradient terms can be written as

$$\begin{aligned}\mathcal{H} =& \frac{1}{2} \sum_{\substack{\mathbf{g},\eta \\ l_1,s_1,l_2,s_2}} \int \mathrm{d}^2 x \, e^{-i \frac{l_2 - l_1}{2} \mathbf{q}_\eta \cdot \mathbf{x}} e^{-i\mathbf{g}\cdot \delta \mathbf{R}(\mathbf{x})} \\ &\times \left[\frac{\partial_{k_x}^{n_x} \partial_{k_y}^{n_y}}{n_x! n_y!} S_{s_1 l_1; s_2 l_2} \left(\mathbf{g}, \mathbf{k}\right) \Big|_{\mathbf{k} = \mathcal{R}_{\theta, l_1} C_{3z}^{\eta} \mathbf{K}_M + \sin\left(\frac{\theta}{2}\right) (\mathbf{g}\times \hat{\mathbf{z}})} \left(i^{n_x + n_y} \partial_x^{n_x} \partial_y^{n_y} \hat{\psi}^\dagger_{\eta, s_1, l_1} \left(\mathbf{x}\right)\right) \hat{\psi}_{\eta, s_2, l_2} \left(\mathbf{x}\right) \right. \\ &\left. + \frac{\partial_{k_x}^{n_x} \partial_{k_y}^{n_y}}{n_x! n_y!} S_{s_1 l_1; s_2 l_2} \left(\mathbf{g}, \mathbf{k}\right) \Big|_{\mathbf{k} = \mathcal{R}_{\theta, l_2} C_{3z}^{\eta} \mathbf{K}_M - \sin\left(\frac{\theta}{2}\right) (\mathbf{g}\times \hat{\mathbf{z}})} \hat{\psi}^\dagger_{\eta, s_1, l_1} \left(\mathbf{x}\right) (-i)^{n_x + n_y} \partial_x^{n_x} \partial_y^{n_y} \hat{\psi}_{\eta, s_2, l_2} \left(\mathbf{x}\right) \right]. \end{aligned} \tag{S7.217}$$

A few remarks are in order regarding Eq. (S7.217):

1. In principle, one could obtain the moiré Hamiltonian, by performing multiple *ab initio* simulations for *untwisted* bilayer systems displaced by different positions. Effectively, this amounts to computing $S_{s_1 l_1; s_2 l_2} \left(\Delta \mathbf{R}, \mathbf{r}\right)$ for multiple values of $\Delta \mathbf{R}$. In turn, this allows one to obtain the generalized moiré continuum Hamiltonian through Eq. (S7.217).

2. It is worth noting that Eq. (S7.217) does not exactly match the form of Eq. (S7.197). Specifically, in Eq. (S7.197), the matrix elements multiplying the term with all the gradients acting on the creation operator and ones multiplying the term with all the gradients on the annihilation operator are the same, unlike Eq. (S7.217), where they are merely hermitian conjugate of one another. This difference is just a matter of notation. Eq. (S7.197) represents one possible, but not the only, fully general parameterization of the generalized moiré Hamiltonian. One could recast Eq. (S7.217) into the form of Eq. (S7.197) by performing a series of integrations by parts, though this falls outside the scope of our current discussion.

3. In the following Section VII C 2, we will show that Eq. (S7.217) can be further simplified by taking the limit $\theta \to 0$. By doing so, we will show that Eq. (S7.217) becomes manifestly of the form of Eq. (S7.197).

2. *Further simplification in the zero-twist limit*

In order to simplify Eq. (S7.217) in the limit of vanishing twist angle, we now take the limit $\theta \to 0$. We cannot ignore the finite twist angle in the exponential prefactors of Eq. (S7.217), since $\mathbf{x}$ assumes values over the entire space. However, we *can* impose the limit $\theta \to 0$ in the momenta at which the $S_{s_1 l_1; s_2 l_2} \left(\mathbf{g}, \mathbf{k}\right)$ is being evaluated. By doing so, we directly obtain

$$\begin{aligned}\mathcal{H} \approx& \frac{1}{2} \sum_{\substack{\mathbf{g}',\eta \\ l_1,s_1,l_2,s_2}} \sum_{n_x, n_y = 0}^{\infty} \frac{\partial_{k_x}^{n_x} \partial_{k_y}^{n_y}}{n_x! n_y!} S_{s_1 l_1; s_2 l_2} \left(\mathbf{g}, \mathbf{k}\right) \Big|_{\mathbf{k} = C_{3z}^{\eta} \mathbf{K}_M} \\ &\times \int \mathrm{d}^2 r \, e^{-i \frac{l_2 - l_1}{2} \mathbf{q}_\eta \cdot \mathbf{r}} e^{-2i \sin\left(\frac{\theta}{2}\right) (\mathbf{g}\times \hat{\mathbf{z}})\cdot \mathbf{r}} \\ &\times \left[\left(i^{n_x + n_y} \partial_x^{n_x} \partial_y^{n_y} \hat{\psi}^\dagger_{\eta, s_1, l_1} \left(\mathbf{r}\right)\right) \hat{\psi}_{\eta, s_2, l_2} \left(\mathbf{r}\right) + (-i)^{n_x + n_y} \hat{\psi}^\dagger_{\eta, s_1, l_1} \left(\mathbf{r}\right) \partial_x^{n_x} \partial_y^{n_y} \hat{\psi}_{\eta, s_2, l_2} \left(\mathbf{r}\right) \right]. \end{aligned} \tag{S7.218}$$

Comparing Eqs. (S7.197) and (S7.218), we find that in the local-stacking approximation and with the zero-twist limit imposed, the real-space generalized moiré potential is given by

$$t^{\eta, n_x, n_y}_{s_1 l_1; s_2 l_2} \left(\mathbf{r}\right) = \sum_{\mathbf{g}} \frac{\partial_{k_x}^{n_x} \partial_{k_y}^{n_y}}{n_x! n_y!} S_{s_1 l_1; s_2 l_2} \left(\mathbf{g}, \mathbf{k}\right) \Big|_{\mathbf{k} = C_{3z}^{\eta} \mathbf{K}_M} e^{-i \frac{l_2 - l_1}{2} \mathbf{q}_\eta \cdot \mathbf{r}} e^{-2i \sin\left(\frac{\theta}{2}\right) (\mathbf{g}\times \hat{\mathbf{z}})\cdot \mathbf{r}}. \tag{S7.219}$$

To obtain the momentum-space generalized moiré potential, we employ Eq. (S7.204) and perform the integral in the same way as it was done in Eq. (S5.121)

$$\left[T^{n_x, n_y}_{\mathbf{q}_{\eta + l_1}, \mathbf{q}_{\eta + l_2} + \mathbf{G}} \right]_{s_1 l_1; s_2 l_2} = \frac{\partial_{k_x}^{n_x} \partial_{k_y}^{n_y}}{n_x! n_y!} S_{s_1 l_1; s_2 l_2} \left(\frac{\left[\mathbf{G} + (l_1 - l_2) \mathbf{q}_{\eta - 1}\right] \times \hat{\mathbf{z}}}{2 \sin\left(\frac{\theta}{2}\right)}, \mathbf{k} \right) \Bigg|_{\mathbf{k} = C_{3z}^{\eta} \mathbf{K}_M}. \tag{S7.220}$$

Eq. (S7.220) is a one-to-one correspondence between the moiré potential and momentum derivatives of the $S_{s_1 l_1; s_2 l_2} \left(\mathbf{g}, \mathbf{k}\right)$ matrix evaluated at the $C_{3z}^{\eta} \mathbf{K}_M$ point, akin to the one of implied by Eq. (S5.121) for the case without gradient terms.

3. Constraining the generalized moiré potential in the local-stacking approximation

As in Section V B 3, we can additionally constrain the generalized moiré potential $T^{n_x,n_y}_{\mathbf{Q},\mathbf{Q}'}$ in the limit of vanishing twist angle by constraining the $S_{s_1l_1;s_2l_2}(\mathbf{g},\mathbf{k})$ matrix. Specifically, as a result of Eq. (S5.102), under a crystalline symmetry g, the latter obeys

$$\sum_{s_1,s_2}\left[D^{\mathrm{sl}}(g)\right]_{s_1's_1} S^{(*)}_{s_1l_1;s_2l_2}(\mathbf{g},\mathbf{k})\left[D^{\mathrm{sl}}(g)\right]^*_{s_2's_2} = S_{s_1'\epsilon_g l_1;s_2'\epsilon_g l_2}(\epsilon_g g\mathbf{g}, g\mathbf{k}). \tag{S7.221}$$

Because the momentum derivatives of $S_{s_1l_1;s_2l_2}(\mathbf{g},\mathbf{k})$ appear in Eq. (S7.220) it is useful to derive the symmetry constrains imposed by g on the partial derivatives of $S_{s_1l_1;s_2l_2}(\mathbf{g},\mathbf{k})$ with respect to $\mathbf{k}$. To do so, we begin by noting that

$$\frac{\partial^{n_x}_{k_x}\partial^{n_y}_{k_y}}{n_x!n_y!}k_x^{m_x}k_y^{m_y} = \frac{\partial^{n_x}_{[g\mathbf{k}]_x}\partial^{n_y}_{[g\mathbf{k}]_y}}{n_x!n_y!}\left([g\mathbf{k}]_x\right)^{m_x}\left([g\mathbf{k}]_y\right)^{m_y} = \delta_{n_x,m_x}\delta_{n_y,m_y}. \tag{S7.222}$$

Because the components of $g\mathbf{k}$ are linear combinations of the components of $\mathbf{k}$, we must have that

$$\frac{\partial^{n_x}_{[g\mathbf{k}]_x}\partial^{n_y}_{[g\mathbf{k}]_y}}{n_x!n_y!} = \sum_{\substack{n_x',n_y'\\ n_x'+n_y'=n_x+n_y}} W_{n_x,n_y;n_x',n_y'}(g)\frac{\partial^{n_x'}_{k_x}\partial^{n_y'}_{k_y}}{n_x'!n_y'!}, \tag{S7.223}$$

where $W_{n_x,n_y;n_x',n_y'}(g)$ is a matrix which will be obtained below. Using the second equality in Eq. (S7.222), as well as Eq. (S7.210), we find that

$$\begin{aligned}
\delta_{n_x,m_x}\delta_{n_y,m_y} &= \sum_{\substack{n_x',n_y'\\ n_x'+n_y'=n_x+n_y}} W_{n_x,n_y;n_x',n_y'}(g)\frac{\partial^{n_x'}_{k_x}\partial^{n_y'}_{k_y}}{n_x'!n_y'!}\left([g\mathbf{k}]_x\right)^{m_x}\left([g\mathbf{k}]_y\right)^{m_y}\\
&= \sum_{\substack{n_x',n_y'\\ n_x'+n_y'=n_x+n_y}}\ \sum_{\substack{m_x',m_y'\\ m_x'+m_y'=m_x+m_y}} W_{n_x,n_y;n_x',n_y'}(g)\frac{\partial^{n_x'}_{k_x}\partial^{n_y'}_{k_y}}{n_x'!n_y'!}U_{m_x',m_y';m_x,m_y}(g)k_x^{m_x'}k_y^{m_y'}\\
&= \sum_{\substack{n_x',n_y'\\ n_x'+n_y'=n_x+n_y\\ m_x+m_y=n_x+n_y}} W_{n_x,n_y;n_x',n_y'}(g)U_{n_x',n_y';m_x,m_y}(g),
\end{aligned} \tag{S7.224}$$

from which we can immediately conclude that

$$W(g) = U^{-1}(g), \tag{S7.225}$$

where the matrix $U(g)$ was defined in Eq. (S7.210). As a result of Eqs. (S7.223) and (S7.225), we can conclude that

$$\frac{\partial^{n_x}_{k_x}\partial^{n_y}_{k_y}}{n_x!n_y!} = \sum_{\substack{n_x',n_y'\\ n_x'+n_y'=n_x+n_y}} U_{n_x,n_y;n_x',n_y'}(g)\frac{\partial^{n_x'}_{[g\mathbf{k}]_x}\partial^{n_y'}_{[g\mathbf{k}]_y}}{n_x'!n_y'!}, \tag{S7.226}$$

By differentiating Eq. (S7.221) with respect to $\mathbf{k}$ and employing Eq. (S7.226), we obtain

$$\begin{aligned}
&\sum_{s_1,s_2}\left[D^{\mathrm{sl}}(g)\right]_{s_1's_1}\frac{\partial^{n_x}_{k_x}\partial^{n_y}_{k_y}}{n_x!n_y!}S^{(*)}_{s_1l_1;s_2l_2}(\mathbf{g},\mathbf{k})\left[D^{\mathrm{sl}}(g)\right]^*_{s_2's_2}\\
=&\frac{\partial^{n_x}_{k_x}\partial^{n_y}_{k_y}}{n_x!n_y!}S_{s_1'\epsilon_g l_1;s_2'\epsilon_g l_2}(\epsilon_g g\mathbf{g}, g\mathbf{k})
\end{aligned}$$

$$\sum_{s_1,s_2}\left[D^{\mathrm{sl}}(g)\right]_{s_1's_1}\frac{\partial_{k_x}^{n_x}\partial_{k_y}^{n_y}}{n_x!n_y!}S^{(*)}_{s_1l_1;s_2l_2}(\mathbf{g},\mathbf{k})\left[D^{\mathrm{sl}}(g)\right]^*_{s_2's_2}$$

$$=\sum_{\substack{n_x',n_y'\\ n_x'+n_y'=n_x+n_y}}U_{n_x,n_y;n_x',n_y'}(g)\frac{\partial_{[g\mathbf{k}]_x}^{n_x'}\partial_{[g\mathbf{k}]_y}^{n_y'}}{n_x'!n_y'!}S_{s_1'\epsilon_g l_1;s_2'\epsilon_g l_2}(\epsilon_g g\mathbf{g},g\mathbf{k})$$

$$\sum_{s_1,s_2}\left[D^{\mathrm{sl}}(g)\right]_{s_1's_1}\frac{\partial_{k_x}^{n_x}\partial_{k_y}^{n_y}}{n_x!n_y!}S^{(*)}_{s_1l_1;s_2l_2}(\mathbf{g},\mathbf{k})\left[D^{\mathrm{sl}}(g)\right]^*_{s_2's_2}$$

$$=\sum_{\substack{n_x',n_y'\\ n_x'+n_y'=n_x+n_y}}U_{n_x,n_y;n_x',n_y'}(g)\left.\left(\frac{\partial_{k_x'}^{n_x'}\partial_{k_y'}^{n_y'}}{n_x'!n_y'!}S_{s_1'\epsilon_g l_1;s_2'\epsilon_g l_2}(\epsilon_g g\mathbf{g},\mathbf{k}')\right)\right|_{\mathbf{k}'=g\mathbf{k}}, \tag{S7.227}$$

which, upon substituting $\mathbf{k}=C_{3z}^{\eta}\mathbf{K}_M$, becomes

$$\sum_{s_1,s_2}\left[D^{\mathrm{sl}}(g)\right]_{s_1's_1}\left.\frac{\partial_{k_x}^{n_x}\partial_{k_y}^{n_y}}{n_x!n_y!}S^{(*)}_{s_1l_1;s_2l_2}(\mathbf{g},\mathbf{k})\right|_{\mathbf{k}=C_{3z}^{\eta}\mathbf{K}_M}\left[D^{\mathrm{sl}}(g)\right]^*_{s_2's_2}$$

$$=\sum_{\substack{n_x',n_y'\\ n_x'+n_y'=n_x+n_y}}U_{n_x,n_y;n_x',n_y'}(g)\left.\left(\frac{\partial_{k_x}^{n_x'}\partial_{k_y}^{n_y'}}{n_x'!n_y'!}S_{s_1'\epsilon_g l_1;s_2'\epsilon_g l_2}(\epsilon_g g\mathbf{g},\mathbf{k})\right)\right|_{\mathbf{k}=gC_{3z}^{\eta}\mathbf{K}_M}. \tag{S7.228}$$

As in Section V B 3, the exact symmetries of the heterostructure in the $\theta\neq 0$ case can either be imposed through Eq. (S7.211) or through Eq. (S7.228), while the zero-twist symmetries can only be imposed indirectly through Eq. (S7.228). In practice, we first impose the exact $\theta\neq 0$ symmetries on the generalized moiré potential through Eq. (S7.211). One can then further simplify the generalized moiré potential by using Eqs. (S7.220) and (S7.228). In the AA-stacking case, additional constraints arise from the $\mathcal{I}$ symmetry, while in the AB-stacking case, the generalized moiré potential can be additionally constrained by the M_z symmetries.

VIII. FIRST-PRINCIPLES RESULTS FOR TWISTED BILAYER MATERIALS WITH VALLEY PROJECTION

This section is dedicated to the valley-projected Hamiltonians of twisted $SnSe_2$ and ZrS_2, as obtained from *ab initio* methods. We begin by outlining how the valley-projected Hamiltonians are constructed from the corresponding OpenMX ones [37–40]. A key method employed to this end is the Löwdin orthogonalization [41–44], which will also be briefly reviewed. The resulting valley-projected Hamiltonians will be employed in Section XI to directly obtain the parameterized continuum moiré Hamiltonians. Lastly, we present some preliminary *ab initio* results on the valley-projected spectrum of twisted $SnSe_2$ and ZrS_2, including the energy dispersion across a range of commensurate angles and the charge density distribution (CDD) at the smallest twist angle considered, $\theta=3.89°$. More comprehensive results at all angles, various continuum models, and further discussion are provided in Section XI.

A. Löwdin orthogonalization

We begin by reviewing Löwdin's orthogonalization method [41–44]. Since this approach can be applied more broadly (*i.e.*, not just in the context of moiré materials) and to avoid keeping track of many indices, we will employ a streamlined notation in this section, which is also summarized in Table S4. Consider a Hamiltonian operator $\hat{H}$, whose energies and corresponding eigenstates are given by ϵ_i and $\left|\psi_i\right\rangle$, respectively, such that

$$\hat{H}\left|\psi_i\right\rangle=\epsilon_i\left|\psi_i\right\rangle. \tag{S8.229}$$

In Eq. (S8.229), i indexes each eigenstate and

$$\left\langle\psi_i|\psi_j\right\rangle=\delta_{ij}. \tag{S8.230}$$

States	Defined in	Meaning
$\lvert\psi_i\rangle$	Eq. (S8.229)	Eigenstates of $\hat{H}$
$\lvert\phi_i\rangle$	Section VIII A	Trial states
$\lvert\bar{\phi}_i\rangle$	Eq. (S8.232)	Projected trial states
$\lvert\tilde{\phi}_i\rangle$	Eq. (S8.234)	Refined trial states
$\lvert\varphi^{\beta}\rangle$	Eq. (S8.239)	Non-orthonormal basis states

TABLE S4. Summary of notation used in Section VIII. Each state is listed along with the place where it is defined and a brief description of its physical meaning.

We now take a set of N linearly independent trial states $|\phi_j\rangle$, indexed by $1 \leq j \leq N$. Our goal is to project the Hamiltonian $\hat{H}$ onto these trial states. We first assume that the Hilbert space spanned by $|\phi_j\rangle$ is *exactly* identical to an N-dimensional eigensubspace of $\hat{H}$ and the trial states $|\phi_j\rangle$ are orthonormal. The spectrum of the projected Hamiltonian, whose matrix elements are given by $\langle\phi_j|\hat{H}|\phi_k\rangle$ (for $1 \leq j,k \leq N$) *exactly* matches the N-dimensional eigensubspace of $\hat{H}$.

More often than not, the trial states do *not* exactly span an N-dimensional eigensubspace of $\hat{H}$. For example, the s orbitals of Sn in monolayer $SnSe_2$ have a high overlap with the lowest gapped conduction band, as shown in Fig. S1, but the Wannier orbitals of the band are described by *effective* molecular s-like orbitals that also have significant weight on the Se orbitals, and not just on the s orbitals of Sn. In such cases, any eigensubspace of $\hat{H}$ will not be *exactly* preserved under the naive projection $\langle\phi_j|\hat{H}|\phi_k\rangle$ (for $1 \leq j,k \leq N$).

To address this, we aim to obtain a refined set of orthonormalized states $\left|\tilde{\phi}_j\right\rangle$ that are *adiabatically* connected to the original trial states $|\phi_j\rangle$, but *exactly* span an N-dimensional eigensubspace of $\hat{H}$. This ensures that the projected spectrum of $\hat{H}$ is *identical* to a particular N-dimensional eigensubspace of $\hat{H}$. In our $SnSe_2$ analogy, $|\phi_j\rangle$ represent the linearly independent (but in general non-orthonormal, due to the nonzero overlaps of different trial states) s orbitals of Sn, while $\left|\tilde{\phi}_j\right\rangle$ correspond to the orthonormal Wannier orbitals of the bottom conduction band, which also behave as effective s orbitals located at the $1a$ Wyckoff position from a symmetry standpoint. Löwdin orthogonalization is a method used to adiabatically refine a set of trial states $|\phi_j\rangle$ (with $1 \leq j \leq N$), such that a relevant N-dimensional portion of the energy spectrum of $\hat{H}$ is *exactly* preserved under the projection onto the resulting *refined* trial states $\left|\tilde{\phi}_j\right\rangle$.

1. *Obtaining the refined trial states*

To illustrate Löwdin orthogonalization, we consider a gapped N-dimensional eigensubspace of $\hat{H}$ spanned by the states $|\psi_j\rangle$ defined in Eq. (S8.229), for $j \in \mathcal{A}$. Given the N trial states $|\phi_i\rangle$, we want to obtain N *refined* trial states $\left|\tilde{\phi}_i\right\rangle$ (adiabatically connected to the $|\phi_i\rangle$ states), such that the eigensubspace $\mathcal{A}$ is exactly preserved under the projection of $\hat{H}$ onto the states $\left|\tilde{\phi}_i\right\rangle$. To this end, we define the projector in the eigensubspace $\mathcal{A}$ to be

$$\hat{P}_{\mathcal{A}} = \sum_{j\in\mathcal{A}} |\psi_j\rangle\langle\psi_j| . \tag{S8.231}$$

The projected trial states

$$\left|\bar{\phi}_j\right\rangle = \hat{P}_{\mathcal{A}}|\phi_j\rangle , \quad \text{for} \quad 1 \leq j \leq N \tag{S8.232}$$

will exactly span the eigensubspace $\mathcal{A}$ (provided that $\left|\bar{\phi}_j\right\rangle$ are linearly independent, which we assume to be the case[7]). They are, however, not orthonormal, with their (Hermitian) overlap matrix being given by

$$\mathcal{S}_{ij} \equiv \left\langle\bar{\phi}_i\middle|\bar{\phi}_j\right\rangle = \langle\phi_i|\hat{P}_{\mathcal{A}}|\phi_j\rangle , \quad \text{for} \quad 1 \leq i,j \leq N. \tag{S8.233}$$

[7] We also assume that the trial states $|\phi_j\rangle$ have finite overlap with the eigensubspace $\mathcal{A}$. In other words, $\hat{P}_{\mathcal{A}}|\phi_j\rangle \neq 0$.

The matrix $\mathcal{S}$ is positive semi-definite, since it is the restriction of the positive semi-definite operator $\hat{P}_{\mathcal{A}}$ on the space spanned by $|\phi_j\rangle$. As we *assume* $|\bar{\phi}_j\rangle$ are linearly independent and nonzero, the matrix $\mathcal{S}$ has no zero eigenvalues, meaning that it is positive *definite*. This allows us to orthonormalize the projected trial state $|\bar{\phi}_j\rangle$ and obtain the *refined* trial state [41–44]

$$\left|\tilde{\phi}_j\right\rangle = \sum_{i=1}^{N} |\bar{\phi}_i\rangle \left(\mathcal{S}^{-1/2}\right)_{ij}, \tag{S8.234}$$

where $\mathcal{S}^{-1/2}$ is the *unique* positive *definite* matrix[8], such that $\left(\mathcal{S}^{-1/2}\right)^2 = \mathcal{S}^{-1}$. The trial states are orthonormal

$$\left\langle\tilde{\phi}_i\middle|\tilde{\phi}_j\right\rangle = \sum_{i',j'=1}^{N} \left(\mathcal{S}^{-1/2}\right)_{ii'} \langle\bar{\phi}_{i'}|\bar{\phi}_{j'}\rangle \left(\mathcal{S}^{-1/2}\right)_{j'j} = \sum_{i',j'=1}^{N} \left(\mathcal{S}^{-1/2}\right)_{ii'} \mathcal{S}_{i'j'} \left(\mathcal{S}^{-1/2}\right)_{j'j} = \delta_{ij} \tag{S8.235}$$

and exactly span the eigensubspace $\mathcal{A}$, by virtue of being linear combinations of the $|\bar{\phi}_j\rangle$ states. The projected Hamiltonian matrix into the $\left|\tilde{\phi}_j\right\rangle$ basis is given by $\tilde{H}_{ij} \equiv \left\langle\tilde{\phi}_i\right|\hat{H}\left|\tilde{\phi}_j\right\rangle$.

All that is left to prove is that the refined states $\left|\tilde{\phi}_j\right\rangle$ are adiabatically connected to the trial states $|\phi_j\rangle$ (meaning that $|\phi_j\rangle$ transform in the same way as $\left|\tilde{\phi}_j\right\rangle$ under the symmetries of the problem). To this end, assume that under a symmetry g of $\hat{H}$ ($\left[\hat{H}, g\right] = 0$), the trial states transform as

$$g|\phi_j\rangle = \sum_{i=1}^{N} U_{ij}|\phi_i\rangle, \tag{S8.236}$$

where U_{ij} is some N-dimensional unitary matrix. Since $\left[\hat{P}_{\mathcal{A}}, g\right] = 0$, it follows that the projected trial states introduced in Eq. (S8.232) transform in the same way as Eq. (S8.236) under the symmetry g, which implies that

$$\mathcal{S}_{ij} = \sum_{i',j'=1}^{N} U^*_{i'i}\mathcal{S}^{(*)}_{i'j'}U_{j'j}, \tag{S8.237}$$

with $^{(*)}$ denoting a complex conjugation in the cases in which g is antiunitary. As a result, we find that

$$\begin{aligned} g\left|\tilde{\phi}_j\right\rangle &= \sum_{i,i'=1}^{N} |\bar{\phi}_{i'}\rangle U_{i'i} \left(\mathcal{S}^{-1/2}\right)^{(*)}_{ij} \\ &= \sum_{i',j'=1}^{N} |\bar{\phi}_{i'}\rangle \left(\mathcal{S}^{-1/2}\right)^{(*)}_{i'j'} U_{j'j} \\ &= \sum_{j'=1}^{N} \left|\tilde{\phi}'_j\right\rangle U_{j'j}, \end{aligned} \tag{S8.238}$$

which means that the refined states transform in the same way as the original trial states, while spanning the N-dimensional eigensubspace $\mathcal{A}$ of $\hat{H}$.

Finally, it is important to reiterate the significance of Löwdin orthogonalization: it refines a set of trial states such that a chosen eigensubspace $\mathcal{A}$ is *exactly* preserved under the projection onto the refined basis. One might wonder, since Löwdin orthogonalization employs the projector $\hat{P}_{\mathcal{A}}$ (which, as shown in Eq. (S8.231), requires explicitly obtaining the eigenstates from $\mathcal{A}$), why not simply use the states $|\psi\rangle_j$ (for $j \in \mathcal{A}$) to form the projected Hamiltonian directly? There are two key reasons:

---

[8] In practice, $\mathcal{S}^{-1/2}$ is obtained by diagonalizing the $\mathcal{S}$ matrix as $\mathcal{S} = VDV^\dagger$, where V is unitary and D is diagonal with strictly positive entries. Defining $D^{-1/2}$ as the diagonal matrix with elements $\left(D^{-1/2}\right)_{ii} = \frac{1}{\sqrt{D_{ii}}}$, we compute $\mathcal{S}^{-1/2}$ as $\mathcal{S}^{-1/2} = VD^{-1/2}V^\dagger$.

- Projecting $\hat{H}$ onto $|\psi_j\rangle$ affords the projected Hamiltonian in the *band* basis, but not in a basis adiabatically connected to the trial states. For example, in the case of the moiré Hamiltonians, we need the projected moiré Hamiltonian in the plane-wave basis, not the band basis.
- While constructing the entire projector $\hat{P}_{\mathcal{A}}$ requires finding the states $|\psi\rangle_j$ (for $j \in \mathcal{A}$) via full diagonalization or shift-and-invert methods, its action on the trial states (*i.e.*, $\hat{P}_{\mathcal{A}} |\phi_j\rangle$) can be computed more efficiently using contour integration techniques [45].

2. *The Löwdin orthogonalization in a non-orthonormal basis*

The Kohn-Sham Hamiltonians used in this work, obtained through *ab initio* methods, are generated with OpenMX [37–40], which employs a pseudo-atomic localized basis[9]. This basis is linearly independent but is *not* orthonormal. For completeness, we now show how the Löwdin orthogonalization, reviewed generally in Section VIII A 1, can be applied for such a non-orthonormal basis.

Let $|\alpha\rangle$ denote a complete non-orthonormal basis (for a lattice Hamiltonian, this would be the atomic orbital basis). The matrix elements of the Hamiltonian $\hat{H}$ in this basis, and the overlap matrix of the $|\varphi^\alpha\rangle$ states, are given by

$$H^{\alpha\beta} \equiv \langle\varphi^\alpha| \hat{H} |\varphi^\beta\rangle , \tag{S8.239}$$

$$S^{\alpha\beta} \equiv \langle\varphi^\alpha|\varphi^\beta\rangle , \tag{S8.240}$$

where $S_{\alpha\beta}$ is a positive definite matrix. The components of the eigenstates of $\hat{H}$ and those of the trial states $|\phi_i\rangle$ in the basis $|\varphi^\alpha\rangle$ are

$$|\psi_i\rangle = \sum_\alpha \psi_i^\alpha |\varphi^\alpha\rangle , \tag{S8.241}$$

$$|\phi_i\rangle = \sum_\alpha \phi_i^\alpha |\varphi^\alpha\rangle , \tag{S8.242}$$

with the vectors ψ_i^α satisfying the generalized eigenvalue equation

$$\sum_\beta H^{\alpha\beta}\psi_i^\beta = \epsilon_i \sum_\beta S^{\alpha\beta}\psi_i^\beta, \tag{S8.243}$$

and being orthonormal with respect to the overlap matrix $S^{\alpha\beta}$

$$\sum_{\alpha,\beta} (\psi_i^\alpha)^* S^{\alpha\beta}\psi_j^\beta = \delta_{ij}. \tag{S8.244}$$

To perform the Löwdin orthonormalization of the $|\bar{\phi}_i\rangle$ projected trial states, we follow the same procedure as described in Section VIII A 1. The Löwdin overlap matrix, as given in Eq. (S8.233), is expressed as

$$\mathcal{S}_{ij} = \langle\bar{\phi}_i|\bar{\phi}_j\rangle = \langle\phi_i| \hat{P}_{\mathcal{A}} |\phi_j\rangle = \sum_{k\in\mathcal{A}} \langle\phi_i|\psi_k\rangle \langle\psi_k|\phi_j\rangle = \sum_{\alpha,\beta,\gamma,\delta} \sum_{k\in\mathcal{A}} (\phi_i^\alpha)^* S^{\alpha\beta}\psi_k^\beta (\psi_k^\gamma)^* S^{\gamma\delta}\phi_j^\delta. \tag{S8.245}$$

Using the Löwdin overlap matrix $\mathcal{S}$, the refined trial states can then be directly obtained from Eq. (S8.234). In the non-orthonormal basis $|\alpha\rangle$, the refined trial states are expressed as

$$\left|\tilde{\phi}_i\right\rangle = \sum_\alpha \tilde{\phi}_i^\alpha |\varphi^\alpha\rangle , \tag{S8.246}$$

with

$$\tilde{\phi}_j^\alpha = \sum_{i=1}^N \sum_{k\in\mathcal{A}} \sum_{\beta,\gamma} \psi_k^\alpha \left(\psi_k^\beta\right)^* S^{\beta\gamma}\phi_i^\gamma \left(\mathcal{S}^{-1/2}\right)_{ij} . \tag{S8.247}$$

Finally, the projected Hamiltonian matrix reads as

$$\tilde{H}_{ij} \equiv \left\langle\tilde{\phi}_i\right| \hat{H} \left|\tilde{\phi}_j\right\rangle = \sum_{\alpha,\beta} \left(\tilde{\phi}_i^\alpha\right)^* H^{\alpha\beta}\tilde{\phi}_j^\beta. \tag{S8.248}$$

---

[9] The pseudo-atomic basis has the same symmetry properties as atomic orbitals but is variationally optimized to reduce the computational cost for large-scale *ab initio* calculations [37–40]

B. Obtaining the plane-wave moiré Hamiltonians from *ab initio* simulations

We employ the Löwdin orthogonalization method followed by projection into the refined basis, in order to construct the valley-projected Hamiltonians for the twisted bilayer systems. This procedure is carried out in two steps:

1. We begin by projecting the *ab initio* Kohn-Sham Hamiltonian onto a set of Wannier basis functions, which are constructed via Löwdin orthogonalization of trial atomic orbitals. For twisted $SnSe_2$, these trial states are the s orbitals of Sn, while for twisted ZrS_2, they are the d_{z^2}, $d_{x^2-y^2}$, and d_{xy} orbitals of Zr.
2. The resulting Wannier-projected Hamiltonian is further projected into each of the three M valleys, by employing a truncated atomic plane wave (TAPW) basis around each valley [46].

Although these two steps can theoretically be combined into a single step that directly projects into each valley, we find that, in practice, the second step requires a careful selection of the projection eigensubspace $\mathcal{A}$ and trial wave functions (*i.e.* number of plane wave states). Therefore, we maintain them as two distinct steps to allow for more precise tuning of the valley projection steps. In the following, we provide a more detailed explanation of each of these two steps.

Our *ab initio* simulations are performed using the OpenMX [37–40] package, which outputs the Kohn-Sham Hamiltonian in a non-orthonormal pseudo-atomic basis. We let $\left|\varphi_{i,l,\alpha,s}\left(\mathbf{R}+\boldsymbol{\tau}_{i,l,\alpha}\right)\right\rangle$ denote the basis state corresponding to orbital α and spin s, located in the moiré unit cell $\mathbf{R}$. Because we work at commensurate twist angles (such that the moiré unit cell contains an integer number of monolayer unit cells), each basis state is additionally labeled by the integer i, which indexes the monolayer unit cell from layer l within the moiré unit cell $\mathbf{R}$. The orbital is also displaced by $\boldsymbol{\tau}_{i,l,\alpha}$ from the moiré unit cell origin. In this section, we adopt the notation outlined in Section VIII A, which is further summarized in Table S4 for trial, projected trial, non-orthonormal basis states, *etc.*. Additional indices such as layer, spin, and unit cell are appended as needed.

The Kohn-Sham spectrum can be determined from the OpenMX Hamiltonian and overlap matrices, which are defined by

$$H_{i,l,\alpha,s;j,l',\beta,s'}\left(\mathbf{k}\right)=\sum_{\mathbf{R}}e^{i\mathbf{k}\cdot\left(-\boldsymbol{\tau}_{i,l,\alpha}+\mathbf{R}+\boldsymbol{\tau}_{j,l',\beta}\right)}\left\langle\varphi_{i,l,\alpha,s}\left(\boldsymbol{\tau}_{i,l,\alpha}\right)\right|\hat{H}_{\mathrm{KS}}\left|\varphi_{i,l',\beta,s'}\left(\mathbf{R}+\boldsymbol{\tau}_{j,l',\beta}\right)\right\rangle, \tag{S8.249}$$

$$S_{i,l,\alpha,s;j,l',\beta,s'}\left(\mathbf{k}\right)=\sum_{\mathbf{R}}e^{i\mathbf{k}\cdot\left(-\boldsymbol{\tau}_{i,l,\alpha}+\mathbf{R}+\boldsymbol{\tau}_{j,l',\beta}\right)}\left\langle\varphi_{i,l,\alpha,s}\left(\boldsymbol{\tau}_{i,l,\alpha}\right)\middle|\varphi_{i,l',\beta,s'}\left(\mathbf{R}+\boldsymbol{\tau}_{j,l',\beta}\right)\right\rangle, \tag{S8.250}$$

where $\mathbf{k}$ denotes the moiré crystalline momentum and $\hat{H}_{\mathrm{KS}}$ is the Kohn-Sham Hamiltonian. The *ab initio* spectrum of $\hat{H}_{\mathrm{KS}}$ can be found from $H_{i,l,\alpha,s;j,l',\beta,s'}\left(\mathbf{k}\right)$ and $S_{i,l,\alpha,s;j,l',\beta,s'}\left(\mathbf{k}\right)$ by solving a generalized eigenvalue problem.

1. *The Wannier projection step*

In the first step, which we denote as *the Wannier projection step*, an appropriate low-energy eigensubspace and a set of trial Wannier orbital states must be selected. For the case of twisted bilayer $SnSe_2$, the spinful s orbitals of Sn provide a suitable set of trial states. Note that while both Sn s and Se p orbitals contribute to the M valley, using only the Sn s orbital is sufficient to reproduce the bands when applying the Löwdin orthogonalization method to the corresponding trial states. The low-energy projection eigensubspace is defined by the lowest set of gapped moiré conduction bands originating from the bottom gapped (spinful) conduction band of the monolayer. This results in a total of $N_{\mathrm{layer}}\times N_a\times N_{\mathrm{spin}}\times N_{\mathrm{trial}}=4N_a$ trial states, where N_a is the number of monolayer unit cells within the moiré unit cell, $N_{\mathrm{spin}}=2$ is the number of spin flavors and $N_{\mathrm{trial}}=1$ is the number of trial orbitals within each monolayer unit cell. For ZrS_2, we choose the d_{z^2}, $d_{x^2-y^2}$, and d_{xy} orbitals of Zr as the trial Wannier orbitals, resulting in $N_{\mathrm{layer}}\times N_a\times N_{\mathrm{spin}}\times N_{\mathrm{trial}}=12N_a$ trial states (where $N_{\mathrm{trial}}=3$). The low-energy projection eigensubspace in this case consists of the $12N_a$ gapped moiré bands, originating from the three lowest gapped (spinful) conduction bands of the monolayer.

In the Löwdin orthogonalization method, the number of states in the eigensubspace $\mathcal{A}$ must match the number of trial states. For $SnSe_2$, the first gapped moiré conduction bands arise from the monolayer's lowest-energy spinful gapped band (see Fig. S1), so we include one atomic orbital trial state per monolayer unit cell. In contrast, for ZrS_2, these bands come from a set of three spinful gapped bands with significant overlap with the d_{z^2}, $d_{x^2-y^2}$, and d_{xy} orbitals of Zr (see Fig. S2). As such, we include three atomic orbital trial states per monolayer unit cell in ZrS_2, corresponding to the d_{z^2}, $d_{x^2-y^2}$, and d_{xy} orbitals of Zr.

Denoting the trial states by $\left|\phi_{i,l,\alpha,s}\left(\mathbf{R}+\boldsymbol{\tau}_{i,l,\alpha}\right)\right\rangle$, where $1\leq\alpha\leq N_{\mathrm{trial}}$, we can find the refined trial states $\left|\tilde{\phi}_{i,l,\alpha,s}\left(\mathbf{R}+\boldsymbol{\tau}_{i,l,\alpha}\right)\right\rangle$ through the Löwdin orthogonalization procedure reviewed in Section VIII A. The refined trial

states are adiabatically connected to the original trial atomic orbitals. As such, they can be indexed with the same quantum numbers, with $\alpha = 1$ in the case of $SnSe_2$ (since we include only one spinful s orbital per monolayer unit cell in the trial state basis) and $\alpha = 1, 2, 3$ for ZrS_2, which, respectively, correspond to the d_{z^2}, $d_{x^2-y^2}$, and d_{xy} orbitals of Zr.

The Wannier projected Kohn-Sham Hamiltonian is given by

$$\tilde{H}_{i,l,\alpha,s;j,l',\beta,s'}(\mathbf{k}) = \left\langle \tilde{\phi}_{i,l,\alpha,s}(\mathbf{k}) \right| \hat{H}_{\text{KS}} \left| \tilde{\phi}_{j,l',\beta,s'}(\mathbf{k}) \right\rangle, \tag{S8.251}$$

where the Fourier-transformed refined trial states are given by

$$\left| \tilde{\phi}_{i,l,\alpha,s}(\mathbf{k}) \right\rangle = \frac{1}{\sqrt{N}} \sum_{\mathbf{R}} e^{i\mathbf{k}\cdot(\mathbf{R}+\boldsymbol{\tau}_{i,l,\alpha})} \left| \tilde{\phi}_{i,l,\alpha,s}(\mathbf{R}+\boldsymbol{\tau}_{i,l,\alpha}) \right\rangle, \tag{S8.252}$$

with N denoting the number of moiré unit cells. Because the number of trial states is significantly smaller than the number of OpenMX basis states, the Wannier projected Hamiltonian matrix $\tilde{H}_{i,l,\alpha,s;j,l',\beta,s'}(\mathbf{k})$ is much easier to manipulate than the original OpenMX matrices from Eqs. (S8.249) and (S8.250). Additionally, the refined trial states are orthonormal, meaning that the spectrum of $\tilde{H}_{i,l,\alpha,s;j,l',\beta,s'}(\mathbf{k})$ can be found by solving a conventional eigenvalue problem.

2. *The valley projection step*

After constructing the low-energy Wannier-projected Hamiltonian, we proceed with the valley projection. This step, while similar to the TAPW method [46], incorporates an additional Löwdin orthogonalization on the trial states, ensuring that the resulting projected Hamiltonian *exactly* matches the low-energy *ab initio* spectrum. The trial states used in the valley projection are written as atomic plane-wave combinations of the refined trial states from the Wannier projection step outlined in Section VIII B 1.

$$\begin{aligned} \left|\phi_{l,s}(\mathbf{k},\mathbf{G})\right\rangle &= \frac{1}{\sqrt{N N_a}} \sum_{\mathbf{R}} \sum_{i} e^{i(\mathbf{k}+\mathbf{G})\cdot(\mathbf{R}+\boldsymbol{\tau}_{i,l,\alpha,s})} \left| \tilde{\phi}_{i,l,\alpha,s}(\mathbf{R}+\boldsymbol{\tau}_{i,l,\alpha}) \right\rangle \\ &= \frac{1}{\sqrt{N_a}} \sum_{i} e^{i\mathbf{G}\cdot\boldsymbol{\tau}_{i,l,\alpha,s}} \left| \tilde{\phi}_{i,l,\alpha,s}(\mathbf{k}) \right\rangle, \quad \text{with} \quad \alpha = 1. \end{aligned} \tag{S8.253}$$

In Eq. (S8.253), N_a is the number of monolayer unit cells in one moiré unit cell, $\mathbf{k}$ denotes the moiré Bloch momentum assuming values in the first moiré BZ, and $\mathbf{G}$ are the reciprocal moiré lattice vectors $\mathbf{G} \in \mathcal{Q}$. The moiré reciprocal vectors $\mathbf{G}$ characterizing the trial states are selected such that $\mathbf{k}+\mathbf{G}$ is close to one of the three M valleys. This gives a total of $N_{\text{layer}} \times N_{\text{spin}} \times N_a \times N_{\mathbf{G}}$ trial states used in the valley projection step, where $N_{\mathbf{G}}$ is the number of plane-wave states considered per valley. For each $\mathbf{G}$, layer l, and spin s, we select a single orbital for both $SnSe_2$ and ZrS_2, corresponding to the effective s or d_{z^2} Wannier orbitals, respectively. For ZrS_2, despite the first Wannier projection step considering three d orbitals of Zr, only the d_{z^2} orbital (having maximal overlap with the lowest-energy monolayer states near the M point) is considered during the valley projection. After Löwdin orthogonalization, the resulting refined trial states are linear combinations of all Zr d orbitals.

Armed with the trial states from Eq. (S8.253), we obtain the corresponding refined trial states $\left|\tilde{\phi}_{l,s}(\mathbf{k},\mathbf{G})\right\rangle$ through the Löwdin orthogonalization method reviewed in Section VIII A. The low-energy projection eigensubspace used for the Löwdin orthogonalization consists of the lowest-energy states of the Wannier-projected Kohn-Sham Hamiltonian defined in Eq. (S8.251). The number of states in this subspace matches the number of trial states used in the valley projection step. The moiré Hamiltonian expressed in the plane-wave basis from Eq. (S4.68) is then obtained by projecting the Kohn-Sham Hamiltonian into the refined trial states $\left|\tilde{\phi}_{l,s}(\mathbf{k},\mathbf{G})\right\rangle$

$$\left[h^{\text{DFT}}_{\mathbf{Q},\mathbf{Q}'}(\mathbf{k})\right]_{s_1 l_1; s_2 l_2} = \left\langle \phi_{l_1,s_1}(\bar{\mathbf{k}},\mathbf{G}) \right| \hat{H}_{\text{KS}} \left| \phi_{l_2,s_2}(\bar{\mathbf{k}},\mathbf{G}') \right\rangle, \tag{S8.254}$$

where

$$C_{3z}^{\eta}\mathbf{K}_M^{l_1} + \mathbf{k} - \mathbf{Q} = \bar{\mathbf{k}} + \mathbf{G}, \quad \text{with} \quad \mathbf{Q} \in \mathcal{Q}_{\eta+l_1} \quad \text{and} \quad \mathbf{G} \in \mathcal{Q}, \tag{S8.255}$$

$$C_{3z}^{\eta}\mathbf{K}_M^{l_2} + \mathbf{k} - \mathbf{Q}' = \bar{\mathbf{k}} + \mathbf{G}', \quad \text{with} \quad \mathbf{Q}' \in \mathcal{Q}_{\eta+l_2} \quad \text{and} \quad \mathbf{G}' \in \mathcal{Q}, \tag{S8.256}$$

for $0 \leq \eta \leq 2$.

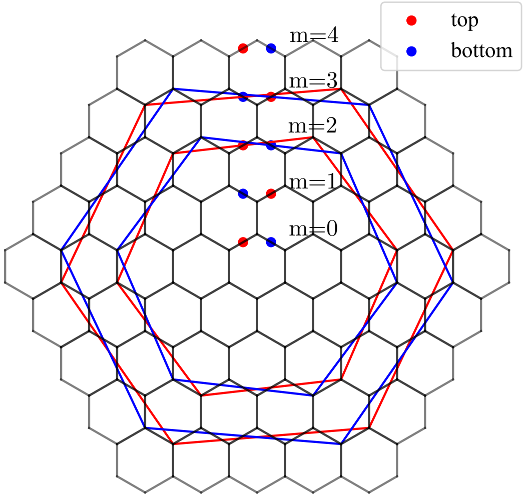

FIG. S17. Mapping between the M valleys of the monolayer and moiré BZs. The red (blue) hexagons represent the top (bottom) monolayer BZs for twist indices $m = 2$ (larger hexagons, with $\theta \approx 13.17°$) and $m = 3$ (smaller hexagons, with $\theta \approx 9.43°$). The twist index m is defined in Eq. (S8.257). Black hexagons denote the moiré BZs. The red (blue) dots correspond to the momentum sublattices $\mathcal{Q}_{+1}$ ($\mathcal{Q}_{-1}$), as defined in the continuum model and shown in Fig. S15. For twist angles with even indices m, the M point of layer l belongs to the momentum sublattice $\mathcal{Q}_l$, while for odd twist angles, it is mapped to $\mathcal{Q}_{-l}$. This behavior contrasts with that of the continuum model, where the M point of layer l is consistently mapped to sublattice $\mathcal{Q}_l$. Note that for better visualization in the plot, we maintain a fixed size for the moiré BZs while adjusting the size of the monolayer BZs. This approach is contrary to the actual scenario, where the monolayer BZs remain constant and the moiré BZs vary with different twist angles.

3. *Alternation in the mapping of M points between the monolayer and moiré BZs*

According to Eqs. (S8.251) and (S8.254), the low-energy spectrum of $\tilde{H}_{i,l,\alpha,s;j,l',\beta,s'}\left(\bar{\mathbf{k}}\right)$ should match the spectrum of $\left[h^{\text{DFT}}_{\mathbf{Q},\mathbf{Q}'}\left(\mathbf{k}\right)\right]_{s_1 l_1; s_2 l_2}$, provided that $\mathbf{k}$ and $\bar{\mathbf{k}}$ are related by Eq. (S8.255). We now examine this correspondence in detail for the commensurate twist angles considered in this study.

Fig. S17 illustrates the relationship between the moiré and twisted monolayer BZs. In our *ab initio* simulations, we employ the following commensurate twist angles, as described in [47]:

$$\theta_m = \arccos\left(\frac{3m^2+3m+1/2}{3m^2+3m+1}\right), \quad m \in \mathbb{N}, \tag{S8.257}$$

which are indexed by the *twist index* m. Considering Eqs. (S8.255) and (S8.256) at $\theta = \theta_m$, we have

$$\begin{aligned} \begin{pmatrix} -\frac{l}{2} \\ \frac{\sqrt{3}}{2}(2m+1) \end{pmatrix} |\mathbf{q}_0| + C_{3z}^{-\eta}\mathbf{k} - \mathbf{q}_l &= C_{3z}^{-\eta}\bar{\mathbf{k}} + \mathbf{G}, \quad \text{for} \quad \mathbf{G} \in \mathcal{Q}, \\ -\frac{m}{2}\mathbf{b}_{M_1} + \left(m + \frac{1-l}{2}\right)\mathbf{b}_{M_2} &= C_{3z}^{-\eta}\left(\bar{\mathbf{k}} - \mathbf{k}\right) + \mathbf{G}, \quad \text{for} \quad \mathbf{G} \in \mathcal{Q}. \end{aligned} \tag{S8.258}$$

This demonstrates that

$$\mathbf{k} = \begin{cases} \bar{\mathbf{k}}, & \text{for even} \quad m \\ \bar{\mathbf{k}} + \mathbf{q}_\eta, & \text{for odd} \quad m \end{cases}. \tag{S8.259}$$

In other words, for even commensurate twist angles, as defined by Eq. (S8.257), the low-energy spectra of the Wannier-projected Hamiltonian from Eq. (S8.251) and the moiré Hamiltonian $h^{\text{DFT}}_{\mathbf{Q},\mathbf{Q}'}\left(\mathbf{k}\right)$ are identical at the same momentum $\mathbf{k}$. For odd commensurate angles, however, the spectrum of the continuum Hamiltonian $h^{\text{DFT}}_{\mathbf{Q},\mathbf{Q}'}\left(\mathbf{k}\right)$ in valley η is shifted by $\mathbf{q}_\eta$ relative to the low-energy spectrum of the Wannier-projected Hamiltonian from Eq. (S8.251).

This relative shift can be understood visually by considering Figs. S15 and S17. In the continuum model illustrated in Fig. S15, the M point of layer l, located at $\mathbf{K}^l_M$, is consistently mapped to the moiré Bloch wave vector $\mathbf{k} = l\mathbf{q}_l$.

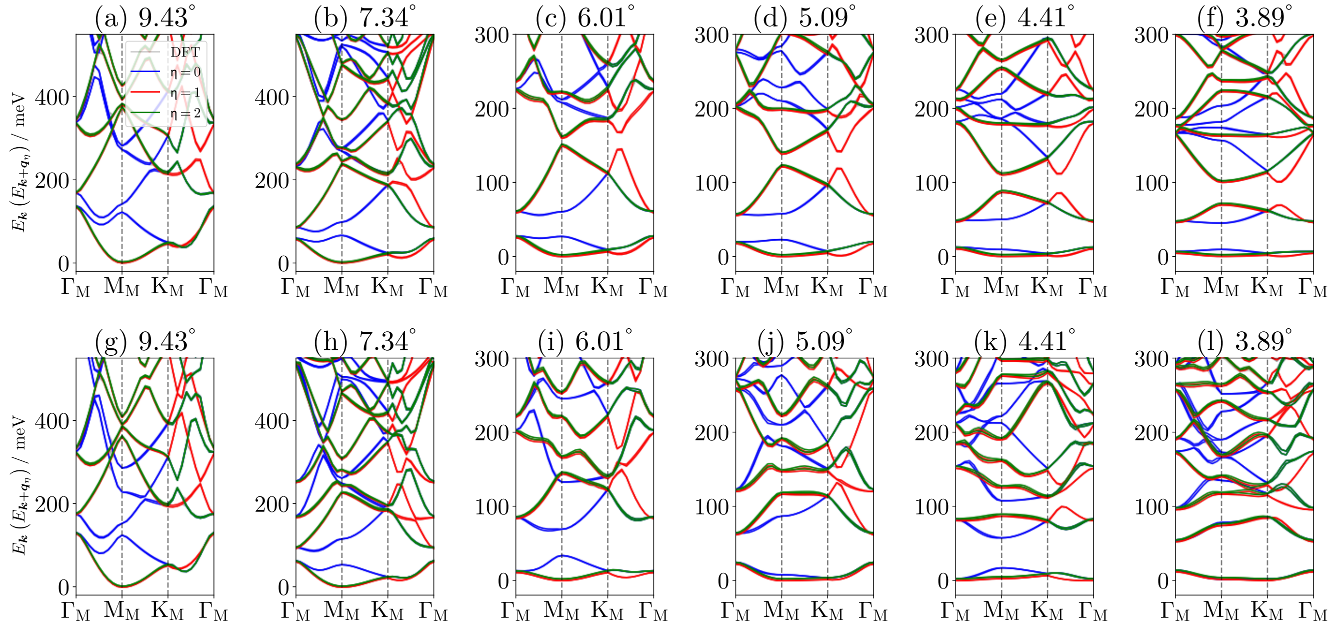

FIG. S18. Valley-projected band structures of twisted bilayer $SnSe_2$ for twist angles ranging from 9.43° to 3.89°. Panels (a)-(f) represent the AA-stacking configuration, while panels (g)-(l) correspond to the AB-stacking configuration, with the twist angle specified above each panel. The plots display only the bottom conduction bands, with contributions from the three M valleys distinguished by different colors. The high-symmetry points used in the plots are defined in Fig. S15(c).

However, for the commensurate angles defined by Eq. (S8.257), this mapping to $\mathbf{k} = l\mathbf{q}_l$ holds only for even m. For odd m, the M point of layer l is instead mapped to $\mathbf{k} = l\mathbf{q}_{-l}$.

When plotting the *ab initio* spectra, we always align $\bar{\mathbf{k}}$ along the same high-symmetry line. The spectra are then compared with those of $h_{\mathbf{Q},\mathbf{Q}'}\left(\bar{\mathbf{k}}\right)$ for even commensurate angles, and with the spectra of $h_{\mathbf{Q},\mathbf{Q}'}\left(\bar{\mathbf{k}}+\mathbf{q}_\eta\right)$ (where $\mathbf{Q}, \mathbf{Q}' \notin \mathcal{Q}_\eta$) for odd commensurate angles.

C. Valley-projected band structure and charge density of $SnSe_2$

In Fig. S18, we present the M-valley-projected band structures for $SnSe_2$ in the AA- and AB-stacking configurations. As the twist angle decreases from 9.43° to 3.89°, two sets of conduction bands near the band minimum become gapped from the higher conduction bands. Each set consists of six bands originating from the three M valleys, with each M valley contributing two nearly degenerate bands due to the approximate SU(2) symmetry of the monolayer, as discussed in Section II A 1.

Within each gapped set, the six bands further split into two subsets of two and four bands, a feature that can be understood through symmetry considerations. At small twist angles, the M valleys are decoupled, and the symmetries of the moiré heterostructure relate the valleys along the high-symmetry paths. We analyze the AA-stacking case as follows:

- Along the $\Gamma_M - \mathrm{M}_M$ line, the four nearly degenerate bands originate from valleys $\eta = 1, 2$. This line possesses C_{2x} symmetry, which exchanges the valleys $\eta = 1$ and $\eta = 2$ while preserving the valley $\eta = 0$. This symmetry leads to the observed four-fold degeneracy among bands from valleys $\eta = 1, 2$.

- Along the $\mathrm{M}_M - \mathrm{K}_M$ path, the four nearly degenerate bands also stem from valleys $\eta = 1, 2$. Here, the $C_{2x}\mathcal{T}$ symmetry of the high-symmetry line exchanges the $\eta = 1$ and $\eta = 2$ valleys while preserving the valley $\eta = 0$.

- Along the $\mathrm{K}_M - \Gamma_M$ line, the bands from valleys $\eta = 0$ and $\eta = 2$ are nearly degenerate. The $\mathrm{K}_M - \Gamma_M$ line is characterized by $C_{2,1\bar{1}0}\mathcal{T}$ symmetry (*i.e.*, a twofold rotation along the $\mathbf{a}_{M_1} - \mathbf{a}_{M_2}$ direction followed by time-reversal symmetry), which exchanges valleys $\eta = 0$ and $\eta = 2$, while leaving the valley $\eta = 1$ invariant.

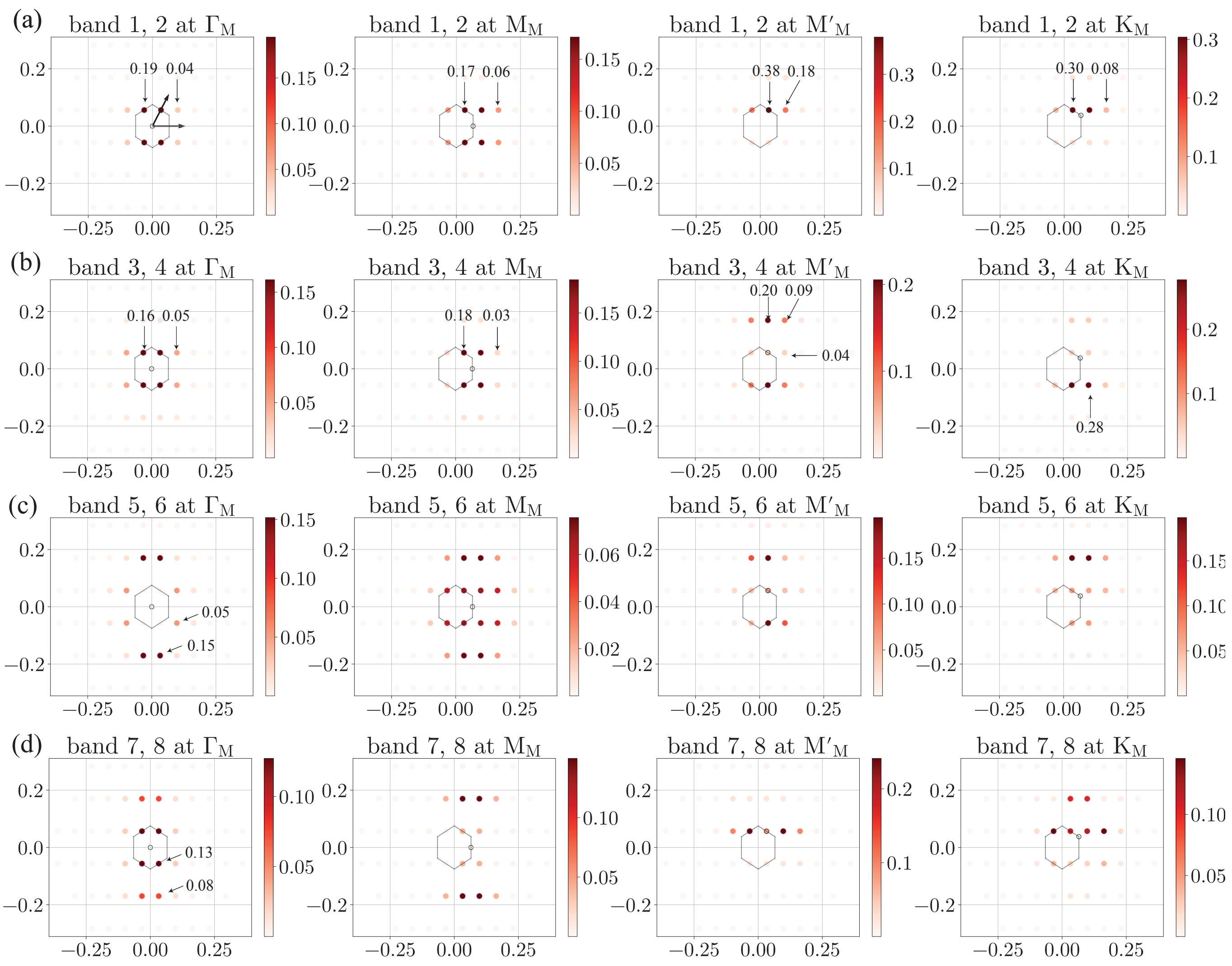

FIG. S19. The *ab initio* spectral weight on the $\mathbf{Q}$-lattice for twisted AA-stacked $SnSe_2$ at $\theta = 3.89°$. Each row, labeled (a)-(d), focuses on one of the lowest four sets of conduction bands in valley $\eta = 0$ at Γ_M, M_M, M'_M, and K_M. The high-symmetry points are marked by black circles in each plot. Each set comprises two bands originating from the two approximately SU (2)-degenerate bands. The total spectral weight is normalized to 1 in each plot. The charge at each $\mathbf{Q}$ point corresponds to the square of the respective wave function component. Notably, the charge distribution is localized at the $\mathbf{Q}$ sites close to the wave function's momentum for the lowest four bands, as expected.

In the AB-stacking case, the degeneracy between bands originating from different valleys remains the same but is protected by different symmetries. The $\Gamma_M - \mathrm{M}_M$, $\mathrm{M}_M - \mathrm{K}_M$, and $\mathrm{K}_M - \Gamma_M$ lines are invariant under the $C_{2y}\mathcal{T}$, C_{2y}, and $C_{2,110}$ symmetries, respectively. These symmetries relate the three M valleys in a manner analogous to the C_{2x}, $C_{2x}\mathcal{T}$, and $C_{2,1\bar{1}0}\mathcal{T}$ symmetries in the AA-stacking case, resulting in the same band degeneracy.

Next, we analyze the distribution of the spectral weight on the $\mathbf{Q}$-lattice at various high-symmetry points of the moiré BZ. Specifically, let the wave function of the n-th band in valley η at $\mathbf{k}$ be $u_{\mathbf{Q},s,l;\eta,n}(\mathbf{k})$. We plot $\sum_s |u_{\mathbf{Q},s,l;\eta,n}(\mathbf{k})|^2$ on the $\mathbf{Q}$-lattice. In Fig. S19, we show the spectral weight distribution on the $\mathbf{Q}$-lattice for the lowest four sets of conduction bands in valley $\eta = 0$ for AA-stacked $SnSe_2$ at $\theta = 3.89°$.

Additionally, Figs. S20(a) and S20(b) show the real-space CDD of twisted AA-stacked $SnSe_2$ at $\theta = 3.89°$. For the lowest set of conduction bands, the charge density is localized near one of the two honeycomb sites, $\frac{2}{3}\mathbf{a}_{M_1} + \frac{1}{3}\mathbf{a}_{M_2}$, forming a triangular lattice. The second-lowest set of bands displays three C_{3z}-symmetric peaks surrounding the honeycomb site, resembling a distorted kagome lattice. However, these peaks do not precisely align with the kagome sites, as the system lacks sufficient symmetry to pin the Wannier center at the kagome positions. In Figs. S20(c) and S20(d), we show the valley-projected CDD for valley $\eta = 0$. For the first set of bands, the charge density is

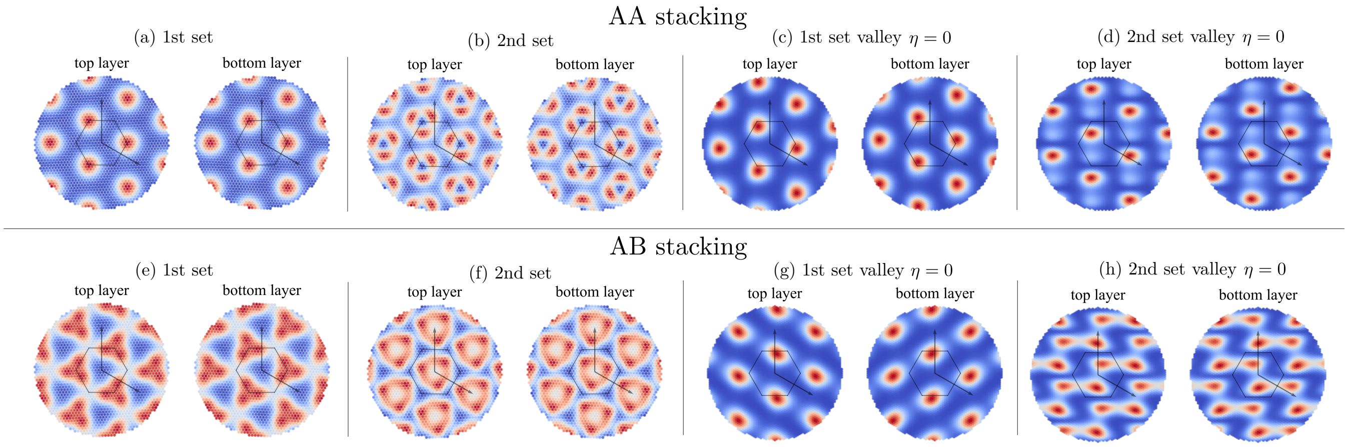

FIG. S20. The *ab initio* charge density distribution (CDD) of twisted AA- and AB-stacked bilayer $SnSe_2$ at $\theta = 3.89°$. (a) and (b) depict the total charge density from the three M valleys for the two lowest sets of conduction bands in the AA-stacking configuration (with the first set being the lowest), while (c) and (d) show the valley-projected CDD for valley $\eta = 0$. (e)-(h) are the same but for the AB-stacking configuration. In each plot, red regions indicate high CDD, blue regions indicate low CDD, the two black arrows represent the moiré unit cell basis vectors, and the hexagon marks the Wigner-Seitz cell.

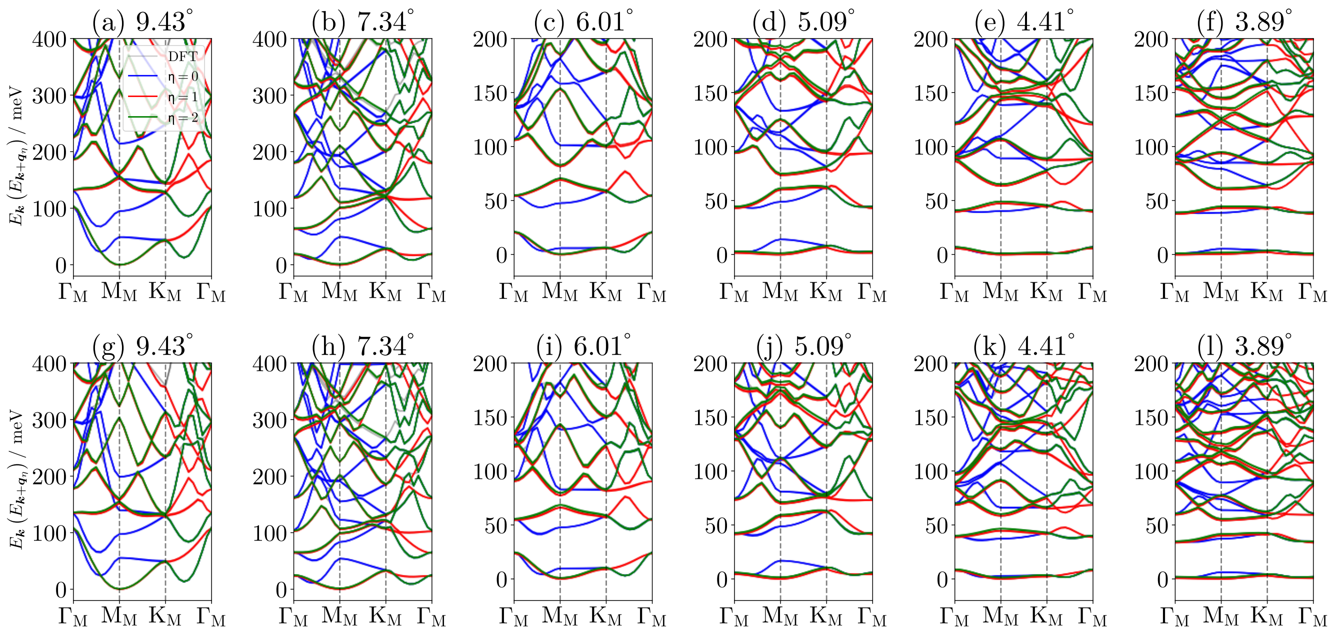

FIG. S21. Valley-projected band structures of twisted bilayer ZrS_2 for twist angles ranging from 9.43° to 3.89°. The layout of the figure is identical to Fig. S18 but applied to ZrS_2.

localized near one honeycomb site. For the second set, the charge is concentrated around $\frac{1}{2}\mathbf{a}_{M_1}$ (*i.e.*, near one of the three kagome sublattices).

In Figs. S20(e) to S20(h), we present the CDD for AB-stacked $SnSe_2$ at a twist angle of $\theta = 3.89°$. As shown in Figs. S20(e) and S20(g), the charge distribution of the first band peaks near the kagome sites, with the Wannier orbitals from each valley approximately localized at one of the three kagome sublattices.

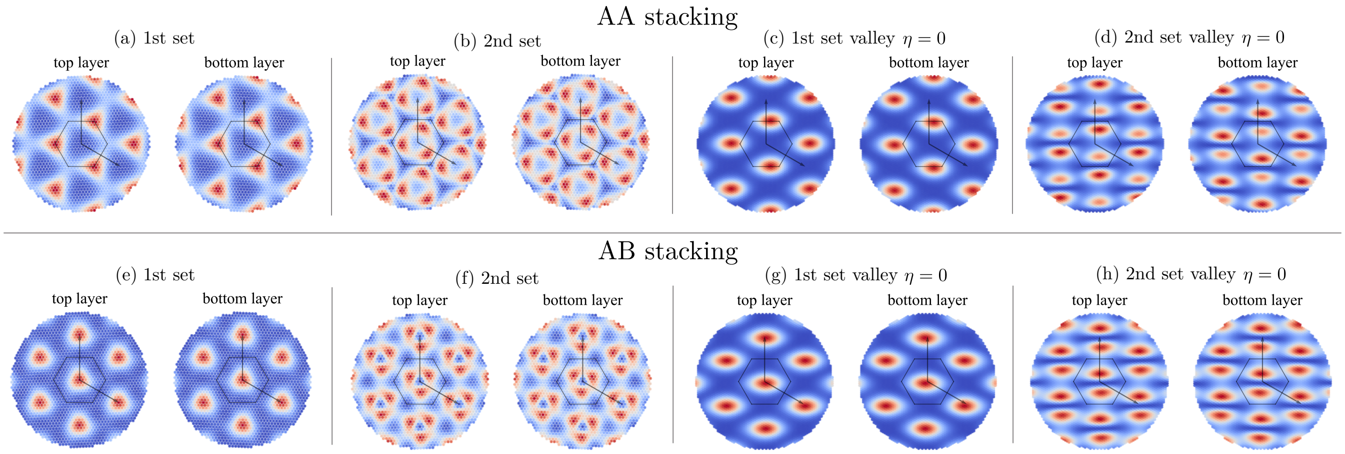

FIG. S22. The *ab initio* CDD of twisted AA- and AB-stacked bilayer ZrS_2 at $\theta = 3.89°$.The layout of the figure is identical to Fig. S20 but applied to ZrS_2.

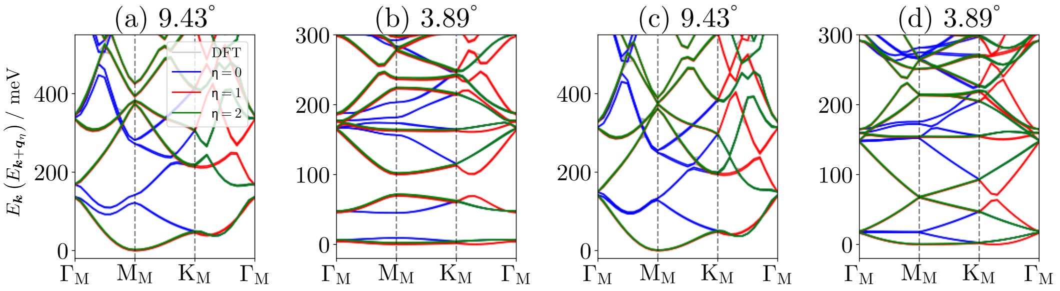

FIG. S23. Effects of lattice relaxation on the band structure of twisted AA-stacked $SnSe_2$. The panels show the valley-projected *ab initio* band structure of twisted AA-stacked $SnSe_2$ at both large and small twist angles, as indicated above each plot. (a) and (b) correspond to fully relaxed crystal structures, while (c) and (d) represent unrelaxed structures where the monolayers are rigidly stacked without considering lattice relaxation, either in-plane or out-of-plane.

D. Valley-projected band structure and charge density of ZrS_2

In Fig. S21, we show the M-valley-projected bands of twisted ZrS_2 for both the AA- and AB-stacking configurations, with twist angles ranging from 9.43° to 3.89°. Similar to $SnSe_2$, two sets of isolated bottom conduction bands emerge as the twist angle decreases to 3.89°.

The corresponding CDD of twisted ZrS_2 is shown in Fig. S22. For the AA-stacked case, the lowest set of conduction bands has its charge density localized at a single honeycomb site, $\frac{1}{3}\mathbf{a}_{M_1} + \frac{2}{3}\mathbf{a}_{M_2}$. The corresponding Wannier orbitals form three nearly overlapping triangular lattices, one for each valley. The CDD of the second-lowest set of bands exhibits three C_{3z}-symmetric peaks surrounding the same honeycomb site. Analyzing the valley-projected charge density reveals that, for the second set, the charge splits into two peaks near the honeycomb site. In the valley $\eta = 0$-projected CDD, the charge distributions between the two layers are related by C_{2x} symmetry.

For the AB-stacked configuration, shown in Figs. S22(e) to S22(h), the charge distribution resembles that of the AA-stacking case but is instead centered at the triangular site $1a$. Additionally, in the AB-stacked case, the charge densities of the two layers are related by C_{2y} symmetry.

E. Effects of lattice relaxation

Lattice relaxation has a significant impact on the electronic spectra of moiré heterostructures. A key feature of our *ab initio* calculations is the use of a comprehensive relaxation procedure that accounts for both in-plane and

out-of-plane atomic displacements. In Fig. S23, we provide a detailed comparison of the effects of lattice relaxation on the band structure of AA-stacked $SnSe_2$. Specifically, we contrast the fully relaxed energy spectra at large and small twist angles with those obtained using unrelaxed crystal structures.

For the unrelaxed structures, the monolayers are rigidly stacked without any in-plane atomic displacements (aside from those resulting from the relative twist angle), and the interlayer distance is fixed to a constant value corresponding to the *average* interlayer separation in the fully relaxed heterostructure.

Inspecting Fig. S23, we find that lattice relaxation induces both qualitative and quantitative changes in the moiré band structure. Notably, the energy gaps shrink significantly in the unrelaxed band structures. These findings highlight the crucial role of lattice relaxation in shaping the electronic spectra of M-point moiré materials, consistent with observations in other moiré heterostructures.

IX. OBTAINING CONTINUUM MODEL FROM *AB INITIO* SIMULATIONS

The *ab initio* moiré Hamiltonian from Eq. (S8.254) is computed only at a finite set of $\mathbf{k}$ points throughout the moiré BZ. However, it is often desirable to have an explicit functional form for $h_{\mathbf{Q},\mathbf{Q}'}(\mathbf{k})$ that can be evaluated, either analytically or numerically, over arbitrary momentum-space $\mathbf{k}$-meshes. To achieve this, we can parameterize $h_{\mathbf{Q},\mathbf{Q}'}(\mathbf{k})$ by enumerating all the symmetry-allowed terms (potentially including gradient terms) and then restricting them according to the *exact* or *approximate* symmetries of the model, as outlined in Sections V and VII. In this section, we describe the method used to extract or *fit* the numerical values for these symmetry-allowed parameters from the *ab initio* moiré Hamiltonian $h^{\text{DFT}}_{\mathbf{Q},\mathbf{Q}'}(\mathbf{k})$. The result is an explicit functional form of $h_{\mathbf{Q},\mathbf{Q}'}(\mathbf{k})$ that can be evaluated at any arbitrary momentum in the moiré BZ.

Because the techniques described in this section can be applied to *any* Hamiltonian matrix, and to avoid tracking many indices, we begin by introducing a simplified generic notation. We then outline how the expansion coefficients of the analytical Hamiltonian matrix, expressed in a symmetry-allowed matrix basis, can be extracted from the *ab initio* Hamiltonian. We present two methods: a linear least-squares method, similar to the one used in Refs. [3, 4] for conventional crystalline systems and in Ref. [5] for moiré models, and a nonlinear fitting method. The linear method, *which does not require parameter fitting*, is effective for obtaining numerically accurate models that quantitatively reproduce most of the *ab initio* spectrum, but potentially with a large number of parameters. In contrast, the nonlinear method is suited for deriving explicit Hamiltonians that quantitatively capture only the low-energy bands (with the higher energy bands reproduced qualitatively), using significantly fewer parameters.

A. Notation

In what follows, we denote by $h^{\text{DFT}}_{ij}(\mathbf{k})$ the *ab-initio* Hamiltonian, which is evaluated at a subset of momenta in the BZ (denoted by $\mathcal{M}$). The indices $1 \leq i, j \leq \mathcal{N}$, where $\mathcal{N}$ is the dimension of $h^{\text{DFT}}_{ij}(\mathbf{k})$, correspond to combined indices such as orbital, spin, plane wave, or other quantum numbers. Our goal is to find the *analytical* explicit matrix function $h_{ij}(\mathbf{k})$ that best matches $h^{\text{DFT}}_{ij}(\mathbf{k})$ at $\mathbf{k} \in \mathcal{M}$ according to a cost function, which will be defined later. The matrix function $h_{ij}(\mathbf{k})$ can be expanded in a predefined, symmetry-constrained basis

$$h_{ij}(\mathbf{k}) = \sum_{\alpha=1}^{d} \lambda_\alpha h_{\alpha;ij}(\mathbf{k}), \tag{S9.260}$$

where d is the dimension of the basis and $h_{\alpha;ij}(\mathbf{k})$ represent d Hermitian matrix functions that obey the symmetry of the problem. The coefficients λ_α are the parameters of the Hamiltonian function $h_{ij}(\mathbf{k})$ (which implicitly depends on them). For example, in the zero-twist limit, the AA-stacked moiré single-particle Hamiltonian can be expanded in the corresponding symmetrized matrix basis as

$$\begin{aligned}\left[h_{\mathbf{Q},\mathbf{Q}'}(\mathbf{k})\right]_{l_1 s_1; l_2 s_2} =& w_2^{\text{AA}} \sum_{n=0}^{2} \left(\delta_{\mathbf{Q},\mathbf{Q}'+\mathbf{q}_n}\delta_{l_1(-l_2)}\delta_{s_1 s_2} + \delta_{\mathbf{Q},\mathbf{Q}'-\mathbf{q}_n}\delta_{l_1(-l_2)}\delta_{s_1 s_2}\right) \\ &+ \frac{1}{2m_x}\delta_{l_1 l_2}\delta_{\mathbf{Q},\mathbf{Q}'}\delta_{s_1 s_2}\left\{(k_x - Q_x)^2\delta_{\zeta_{\mathbf{Q}}-l_1,0} + \left[\frac{(k_x-Q_x)\sqrt{3}+(k_y-Q_y)}{2}\right]^2\delta_{\zeta_{\mathbf{Q}}-l_1,2}\right. \\ &\left. + \left[\frac{(k_x-Q_x)\sqrt{3}-(k_y-Q_y)}{2}\right]^2\delta_{\zeta_{\mathbf{Q}}-l_1,1}\right\} + \dots,\end{aligned} \tag{S9.261}$$

with the ellipses denoting other terms. In Eq. (S9.261), each term comprises a parameter (*e.g.*, w_2^{AA} and $\frac{1}{2m_x}$) multiplying a Hermitian matrix.

The goal of this section is to fix the parameters of the expansion λ_α such that $h_{ij}(\mathbf{k})$ best reproduces (according to a cost function defined below) the numerically obtained $h_{ij}^{\mathrm{DFT}}(\mathbf{k})$ for $\mathbf{k} \in \mathcal{M}$. For this purpose, it is also useful to define the eigenvalues $\epsilon_n(\mathbf{k})$ and eigenstates $\phi_{n;j}(\mathbf{k})$ of the $h_{ij}^{\mathrm{DFT}}(\mathbf{k})$ and $h_{ij}(\mathbf{k})$ matrices according to

$$\sum_j h_{ij}^{(\mathrm{DFT})}(\mathbf{k})\,\phi_{n;j}^{(\mathrm{DFT})}(\mathbf{k}) = \epsilon_n^{(\mathrm{DFT})}(\mathbf{k})\,\phi_{n;i}^{(\mathrm{DFT})}(\mathbf{k}), \tag{S9.262}$$

where n indexes the eigenpairs in the order of increasing eigenvalue. We will now define two types of cost functions and briefly describe the numerical methods used for their optimization.

B. Linear Extraction

Perhaps the simplest cost function is the Frobenius norm of the matrix difference between the Hamiltonian matrix function and the *ab initio* one

$$\mathcal{C}_0(\{\lambda_\alpha\}) = \sum_{\mathbf{k}\in\mathcal{M}} \mathrm{Tr}\left[\left(h(\mathbf{k}) - h^{\mathrm{DFT}}(\mathbf{k})\right)^2\right]. \tag{S9.263}$$

In terms of the expansion coefficients from Eq. (S9.260), Eq. (S9.263) takes a quadratic form

$$\mathcal{C}_0(\{\lambda_\alpha\}) = \sum_{\alpha,\beta=1}^{d} A_{\alpha\beta}^{(0)}\lambda_\alpha\lambda_\beta - 2\sum_{\alpha=1}^{d} B_\alpha^{(0)}\lambda_\alpha + C^{(0)}, \tag{S9.264}$$

where the real coefficients are given by

$$A_{\alpha\beta}^{(0)} = \mathrm{Re}\left[\sum_{\mathbf{k}\in\mathcal{M}} \mathrm{Tr}\left(h_\alpha(\mathbf{k})\,h_\beta(\mathbf{k})\right)\right], \tag{S9.265}$$

$$B_\alpha^{(0)} = \sum_{\mathbf{k}\in\mathcal{M}} \mathrm{Tr}\left(h_\alpha(\mathbf{k})\,h^{\mathrm{DFT}}(\mathbf{k})\right), \tag{S9.266}$$

$$C^{(0)} = \sum_{\mathbf{k}\in\mathcal{M}} \mathrm{Tr}\left(h^{\mathrm{DFT}}(\mathbf{k})\,h^{\mathrm{DFT}}(\mathbf{k})\right). \tag{S9.267}$$

It is easy to check that the real symmetric matrix $A_{\alpha\beta}^{(0)}$ is also positive definite. Therefore, the cost function $\mathcal{C}_0$ admits a single minimum for

$$\lambda_\alpha = \left[\left(A^{(0)}\right)^{-1} B^{(0)}\right]_\alpha. \tag{S9.268}$$

We refer to this method as "linear extraction", analogous to the linear least-squares method. In the form given in Eq. (S9.263), this method was employed by Ref. [5] to obtain moiré Hamiltonians from *ab initio* simulations.

1. *Low-energy restriction*

The cost function from Eq. (S9.263) has one shortcoming: it attempts to fit the *entire* energy spectrum of $h^{\mathrm{DFT}}(\mathbf{k})$. This becomes clearer if we express Eq. (S9.263) in the eigenbasis of $h^{\mathrm{DFT}}(\mathbf{k})$

$$\mathcal{C}_0(\{\lambda_\alpha\}) = \sum_{\mathbf{k}\in\mathcal{M}}\sum_n \phi_{n;i}^{*\mathrm{DFT}}(\mathbf{k})\left(h_{ij}(\mathbf{k}) - h_{ij}^{\mathrm{DFT}}(\mathbf{k})\right)\left(h_{jk}(\mathbf{k}) - h_{jk}^{\mathrm{DFT}}(\mathbf{k})\right)\phi_{n;k}^{\mathrm{DFT}}(\mathbf{k}). \tag{S9.269}$$

In practice, however, we are primarily interested in the low-energy spectrum of $h^{\mathrm{DFT}}(\mathbf{k})$. To reduce the number of parameters in $h(\mathbf{k})$ and focus on improving the match with the low-energy spectrum, we can introduce an energy weighting function into the cost function from Eq. (S9.269), defining a new cost function

$$\mathcal{C}_1(\{\lambda_\alpha\}) = \sum_{\mathbf{k}\in\mathcal{M}}\sum_n w\left(\epsilon_n^{\mathrm{DFT}}(\mathbf{k})\right)\phi_{n;i}^{*\mathrm{DFT}}(\mathbf{k})\left(h_{ij}(\mathbf{k}) - h_{ij}^{\mathrm{DFT}}(\mathbf{k})\right)\left(h_{jk}(\mathbf{k}) - h_{jk}^{\mathrm{DFT}}(\mathbf{k})\right)\phi_{n;k}^{\mathrm{DFT}}(\mathbf{k}), \tag{S9.270}$$

where $w(\epsilon)$ is the energy weighting function. In this work, we use a smooth step function, parameterized by an energy cutoff ϵ_c and a spread σ

$$w(\epsilon) = \frac{1}{2}\left(1 - \tanh\left(\frac{\epsilon - \epsilon_c}{\sigma}\right)\right), \tag{S9.271}$$

with the cutoff and spread chosen based on the band structure. The cost function from Eq. (S9.270) also takes a quadratic form in terms of the expansion coefficients from Eq. (S9.260)

$$\mathcal{C}_1(\{\lambda_\alpha\}) = \sum_{\alpha,\beta=1}^{d} A^{(1)}_{\alpha\beta}\lambda_\alpha\lambda_\beta - 2\sum_{\alpha=1}^{d} B^{(1)}_\alpha\lambda_\alpha + C^{(1)}, \tag{S9.272}$$

where the coefficients are given by

$$A^{(1)}_{\alpha\beta} = \mathrm{Re}\left[\sum_{\mathbf{k}\in\mathcal{M}}\sum_n w\left(\epsilon_n^{\mathrm{DFT}}(\mathbf{k})\right)\phi^{*\mathrm{DFT}}_{n;i}(\mathbf{k})\, h_{\alpha;ij}(\mathbf{k})\, h_{\beta;jk}(\mathbf{k})\, \phi^{\mathrm{DFT}}_{n;k}(\mathbf{k})\right], \tag{S9.273}$$

$$B^{(1)}_{\alpha} = \mathrm{Re}\left[\sum_{\mathbf{k}\in\mathcal{M}}\sum_n w\left(\epsilon_n^{\mathrm{DFT}}(\mathbf{k})\right)\phi^{*\mathrm{DFT}}_{n;i}(\mathbf{k})\, h_{\alpha;ij}(\mathbf{k})\, h^{\mathrm{DFT}}_{jk}(\mathbf{k})\, \phi^{\mathrm{DFT}}_{n;k}(\mathbf{k})\right], \tag{S9.274}$$

$$C^{(1)} = \sum_{\mathbf{k}\in\mathcal{M}}\sum_n w\left(\epsilon_n^{\mathrm{DFT}}(\mathbf{k})\right)\phi^{*\mathrm{DFT}}_{n;i}(\mathbf{k})\, h^{\mathrm{DFT}}_{ij}(\mathbf{k})\, h^{\mathrm{DFT}}_{jk}(\mathbf{k})\, \phi^{\mathrm{DFT}}_{n;k}(\mathbf{k}). \tag{S9.275}$$

Since $A^{(1)}_{\alpha\beta}$ is also positive definite, the global minimum of Eq. (S9.270) can again be found analytically

$$\lambda_\alpha = \left[\left(A^{(1)}\right)^{-1} B^{(1)}\right]_\alpha. \tag{S9.276}$$

This method, though with a different cost function, was used by Refs. [3, 4] to fit *ab initio* phonon and electronic spectra.

2. *Partial low-energy restriction*

The $\mathbf{Q}$-diagonal terms of the moiré Hamiltonian characterize the single-layer dispersion around the M point. When including $\mathbf{Q}$-diagonal terms of the form in Eq. (S7.205), with a large polynomial degree corresponding to $n_x + n_y > 4$, a larger energy cutoff is required to reproduce them. Empirically, we find that a larger energy cutoff requires more moiré harmonics and higher-order gradients to obtain a good match with the low-energy bands. To address this, and to include $\mathbf{Q}$-diagonal terms with a high polynomial degree in $\mathbf{k}$ while keeping the number of moiré harmonics and their orders small, we separate the *ab initio* Hamiltonian into $\mathbf{Q}$-diagonal and $\mathbf{Q}$-off-diagonal terms

$$h^{\mathrm{DFT}}(\mathbf{k}) = h^{\mathrm{DFT,dg}}(\mathbf{k}) + h^{\mathrm{DFT,o\text{-}dg}}(\mathbf{k}). \tag{S9.277}$$

Similarly, the matrix basis from Eq. (S9.260) can be split into $\mathbf{Q}$-diagonal and $\mathbf{Q}$-off-diagonal terms. Without loss of generality, we arrange the matrix basis so that $h_\alpha(\mathbf{k})$ for $1 \leq \alpha \leq d'$ ($d' < \alpha \leq d$) are all diagonal (off-diagonal) in $\mathbf{Q}$, where $1 < d' < d$ is the number of $\mathbf{Q}$-diagonal terms considered in the matrix expansion of $h(\mathbf{k})$. The model Hamiltonian $h(\mathbf{k})$ also splits into $\mathbf{Q}$-diagonal and $\mathbf{Q}$-off-diagonal terms

$$h(\mathbf{k}) = h^{\mathrm{dg}}(\mathbf{k}) + h^{\mathrm{o\text{-}dg}}(\mathbf{k}). \tag{S9.278}$$

with

$$h^{\mathrm{dg}}(\mathbf{k}) = \sum_{\alpha=1}^{d'} h_\alpha(\mathbf{k}), \tag{S9.279}$$

$$h^{\mathrm{o\text{-}dg}}(\mathbf{k}) = \sum_{\alpha=d'+1}^{d} h_\alpha(\mathbf{k}). \tag{S9.280}$$

The cost function Eq. (S9.263) can then be separated into **Q**-diagonal and **Q**-off-diagonal parts

$$\mathcal{C}_0\left(\{\lambda_\alpha\}\right)=\sum_{\mathbf{k}\in\mathcal{M}}\operatorname{Tr}\left[\left(h^{\mathrm{dg}}\left(\mathbf{k}\right)-h^{\mathrm{DFT,dg}}\left(\mathbf{k}\right)\right)^2\right]+\sum_{\mathbf{k}\in\mathcal{M}}\operatorname{Tr}\left[\left(h^{\mathrm{o\text{-}dg}}\left(\mathbf{k}\right)-h^{\mathrm{DFT,o\text{-}dg}}\left(\mathbf{k}\right)\right)^2\right]. \tag{S9.281}$$

We can apply a similar strategy to that introduced in Section IX B 1 and introduce an energy weighting function in the second term. This allows the moiré potential terms (which are **Q**-off-diagonal) to be determined using only the low-energy sector, while the **Q**-diagonal terms, characterizing the single-layer dispersion part of the moiré Hamiltonian, are determined using the entire *ab initio* Hamiltonian. The corresponding cost function again takes a quadratic form in the fitting parameters

$$\mathcal{C}_2\left(\{\lambda_\alpha\}\right)=\sum_{\alpha,\beta=1}^{d}A^{(2)}_{\alpha\beta}\lambda_\alpha\lambda_\beta-2\sum_{\alpha=1}^{d}B^{(2)}_{\alpha}\lambda_\alpha+C^{(2)}, \tag{S9.282}$$

where the coefficients are given by

$$A^{(2)}_{\alpha\beta}=\begin{cases}\operatorname{Re}\left[\sum_{\mathbf{k}\in\mathcal{M}}\operatorname{Tr}\left(h_\alpha\left(\mathbf{k}\right)h_\beta\left(\mathbf{k}\right)\right)\right] & 1\le\alpha,\beta\le d'\\ \operatorname{Re}\left[\sum_{\mathbf{k}\in\mathcal{M}}\sum_n w\left(\epsilon^{\mathrm{DFT}}_n\left(\mathbf{k}\right)\right)\phi^{*\mathrm{DFT}}_{n;i}\left(\mathbf{k}\right)h_{\alpha;ij}\left(\mathbf{k}\right)h_{\beta;jk}\left(\mathbf{k}\right)\phi^{\mathrm{DFT}}_{n;k}\left(\mathbf{k}\right)\right] & d'+1\le\alpha,\beta\le d\\ 0 & \text{otherwise}\end{cases}, \tag{S9.283}$$

$$B^{(2)}_{\alpha}=\begin{cases}\sum_{\mathbf{k}\in\mathcal{M}}\operatorname{Tr}\left(h_\alpha\left(\mathbf{k}\right)h^{\mathrm{DFT,dg}}\left(\mathbf{k}\right)\right) & 1\le\alpha\le d'\\ \operatorname{Re}\left[\sum_{\mathbf{k}\in\mathcal{M}}\sum_n w\left(\epsilon^{\mathrm{DFT}}_n\left(\mathbf{k}\right)\right)\phi^{*\mathrm{DFT}}_{n;i}\left(\mathbf{k}\right)h_{\alpha;ij}\left(\mathbf{k}\right)h^{\mathrm{DFT}}_{jk}\left(\mathbf{k}\right)\phi^{\mathrm{DFT,o\text{-}dg}}_{n;k}\left(\mathbf{k}\right)\right] & d'+1\le\alpha\le d\end{cases}, \tag{S9.284}$$

$$\begin{aligned}C^{(2)}=&\sum_{\mathbf{k}\in\mathcal{M}}\operatorname{Tr}\left(h^{\mathrm{DFT,dg}}\left(\mathbf{k}\right)h^{\mathrm{DFT,dg}}\left(\mathbf{k}\right)\right)\\&+\sum_{\mathbf{k}\in\mathcal{M}}\sum_n w\left(\epsilon^{\mathrm{DFT}}_n\left(\mathbf{k}\right)\right)\phi^{*\mathrm{DFT}}_{n;i}\left(\mathbf{k}\right)h^{\mathrm{DFT,o\text{-}dg}}_{ij}\left(\mathbf{k}\right)h^{\mathrm{DFT,o\text{-}dg}}_{jk}\left(\mathbf{k}\right)\phi^{\mathrm{DFT}}_{n;k}\left(\mathbf{k}\right).\end{aligned} \tag{S9.285}$$

Because the matrix $A^{(2)}$ is positive definite, the global minimum can be found analytically using

$$\lambda_\alpha=\left[\left(A^{(2)}\right)^{-1}B^{(2)}\right]_\alpha. \tag{S9.286}$$

C. Nonlinear fitting

In the previous section, we outlined how the Hamiltonian matrix can be determined using a cost function based on the Frobenius norm of the difference between the model and *ab initio* Hamiltonians. The linear extraction method in the previous section does not require any parameter fitting because the global minimum can be found analytically. Empirically, when the number of parameters is very small (as in the case of the first moiré harmonic model from Section V A or its enhanced-symmetry limits discussed in Section VI), we find that linear extraction over a large energy window yields unsatisfactory results for the low-energy bands. When the energy cutoff ϵ_c is reduced, the model Hamiltonian can exhibit "ghost states" – additional bands appearing in the model Hamiltonian that are not present in the *ab initio* one. This occurs because the cost function in Eq. (S9.270) ensures that $h\left(\mathbf{k}\right)$ reproduces the eigenstates of $h^{\mathrm{DFT}}\left(\mathbf{k}\right)$ within the energy window, but does *not* prevent the emergence of extra states.

To address this issue, we can design a new cost function that ensures the *first* $\mathcal{N}_{\mathrm{Band}}$ bands of $h\left(\mathbf{k}\right)$ match the *first* $\mathcal{N}_{\mathrm{Band}}$ bands of $h^{\mathrm{DFT}}\left(\mathbf{k}\right)$, thereby preventing the appearance of ghost states within these bands. To achieve this, we introduce projector operators onto the first $\mathcal{N}_{\mathrm{Band}}$ bands of the fitted and *ab initio* Hamiltonians

$$P^{(\mathrm{DFT})}_{ij}\left(\mathbf{k}\right)=\sum_{m=1}^{\mathcal{N}_{\mathrm{Band}}}n_m\phi^{(\mathrm{DFT})}_{m;i}\left(\mathbf{k}\right)\phi^{*(\mathrm{DFT})}_{m;j}\left(\mathbf{k}\right)=\left[\frac{1}{2\pi i}\oint\mathrm{d}z\left(z\mathbb{1}-h^{(\mathrm{DFT})}\left(\mathbf{k}\right)\right)^{-1}\right]_{ij}, \tag{S9.287}$$

where

$$n_m=\begin{cases}1 & 1\le m\le\mathcal{N}_{\mathrm{Band}}\\ 0 & \mathcal{N}_{\mathrm{Band}}<m\le\mathcal{N}\end{cases}. \tag{S9.288}$$

The complex contour in Eq. (S9.287) is taken to enclose the first $\mathcal{N}_{\text{Band}}$ bands of the corresponding (*i.e.*, fitted or *ab initio*) Hamiltonian. It is also important to note that the projector $P(\mathbf{k})$ for the fitted Hamiltonian explicitly depends on the fitting parameters λ_α. A suitable cost function for fitting the first $\mathcal{N}_{\text{Band}}$ bands is

$$\mathcal{C}'(\{\lambda_\alpha\}) = \sum_{\mathbf{k}\in\mathcal{M}} \operatorname{Tr}\left[\left(P(\mathbf{k})\,h(\mathbf{k}) - P^{\text{DFT}}(\mathbf{k})\,h^{\text{DFT}}(\mathbf{k})\right)^2\right]. \tag{S9.289}$$

Due to the nontrivial dependence of the cost function $\mathcal{C}'(\{\lambda_\alpha\})$ on the fitting parameters, it can no longer be minimized analytically, as in the linear extraction method outlined in Section IX C. Therefore, the nonlinear fitting method requires parameter fitting. As a result, iterative numerical minimization techniques must be employed, and this approach is referred to as *nonlinear fitting*. In this work, we use a Quasi-Newton method (as implemented in Mathematica) to find the local minimum of $\mathcal{C}'(\{\lambda_\alpha\})$, starting from the initial conditions that globally minimize the $\mathcal{C}(\{\lambda_\alpha\})$ cost function from Eq. (S9.263).

1. *Analytical expression for the gradient of the cost function*

A key intermediate quantity in the Quasi-Newton method for minimization is the gradient of the cost function with respect to the fitting parameters. In principle, this gradient can be computed numerically using finite differences. However, for the cost function from Eq. (S9.289), the gradient has an analytical expression that is numerically much easier to compute. In what follows, we derive an analytical formula for the gradient of the cost function. Introducing the shorthand notation

$$\partial_\alpha \equiv \frac{\partial}{\partial\lambda_\alpha}, \tag{S9.290}$$

we find that

$$\partial_\alpha\mathcal{C}'(\{\lambda_\alpha\}) = 2\sum_{\mathbf{k}\in\mathcal{M}} \operatorname{Tr}\left[\left(P(\mathbf{k})\,h(\mathbf{k}) - P^{\text{DFT}}(\mathbf{k})\,h^{\text{DFT}}(\mathbf{k})\right)\partial_\alpha\left(P(\mathbf{k})\,h(\mathbf{k})\right)\right]. \tag{S9.291}$$

Since

$$\partial_\alpha\left(P(\mathbf{k})\,h(\mathbf{k})\right) = \left(\partial_\alpha P(\mathbf{k})\right)h(\mathbf{k}) + P(\mathbf{k})\,h_\alpha(\mathbf{k}), \tag{S9.292}$$

we need to compute the partial derivative of the projector $\partial_\alpha P(\mathbf{k})$. This is most easily expressed using the integral expression from Eq. (S9.287)

$$\partial_\alpha P(\mathbf{k}) = \frac{1}{2\pi i}\oint \mathrm{d}z\,\left(z\mathbb{1} - h(\mathbf{k})\right)^{-1} h_\alpha(\mathbf{k})\left(z\mathbb{1} - h(\mathbf{k})\right)^{-1}, \tag{S9.293}$$

which can be cast in the band basis

$$\begin{aligned}\sum_{i,j}\phi^{*s}_{m;i}(\mathbf{k})\left(\partial_\alpha P_{ij}(\mathbf{k})\right)\phi_{l;j}(\mathbf{k}) &= \frac{1}{2\pi i}\sum_{i,j}\oint\mathrm{d}z\,\frac{\phi^{*s}_{m;i}(\mathbf{k})\,h_{\alpha;ij}(\mathbf{k})\,\phi_{l;j}(\mathbf{k})}{\left(z-\epsilon_m(\mathbf{k})\right)\left(z-\epsilon_l(\mathbf{k})\right)}\\ &= \frac{n_m - n_l}{\epsilon_m(\mathbf{k}) - \epsilon_l(\mathbf{k})}\sum_{i,j}\phi^{*s}_{m;i}(\mathbf{k})\,h_{\alpha;ij}(\mathbf{k})\,\phi_{l;j}(\mathbf{k})\end{aligned} \tag{S9.294}$$

By plugging Eq. (S9.294) into Eq. (S9.292), we find

$$\begin{aligned}\sum_{i,j}\phi^{*s}_{m;i}(\mathbf{k})\left[\partial_\alpha\left(P(\mathbf{k})\,h(\mathbf{k})\right)\right]_{ij}\phi_{l;j}(\mathbf{k}) &= \sum_{i,j}\left(\epsilon_l(\mathbf{k})\,\phi^{*s}_{m;i}(\mathbf{k})\,\partial_\alpha P_{ij}(\mathbf{k})\,\phi_{l;j}(\mathbf{k}) + n_m\phi^{*s}_{m;i}(\mathbf{k})\,h_{\alpha;ij}(\mathbf{k})\,\phi_{l;j}(\mathbf{k})\right)\\ &= \frac{n_m\epsilon_m(\mathbf{k}) - n_l\epsilon_l(\mathbf{k})}{\epsilon_m(\mathbf{k}) - \epsilon_l(\mathbf{k})}\sum_{i,j}\phi^{*s}_{m;i}(\mathbf{k})\,h_{\alpha;ij}(\mathbf{k})\,\phi_{l;j}(\mathbf{k}).\end{aligned} \tag{S9.295}$$

Eq. (S9.295) gives the matrix elements of $\partial_\alpha\left(P(\mathbf{k})\,h(\mathbf{k})\right)$ in the band basis of the fitted Hamiltonian. Plugging this into Eq. (S9.291) provides an analytical expression for the gradient of the cost function, significantly improving the efficiency of the nonlinear minimization.

D. Reducing the number of parameter

In both the linear and nonlinear fitting methods, it is desirable to minimize the number of fitting parameters. To achieve this, we employ the concept of *step-wise* regression. In step-wise regression, a model with $\mathcal{N}-1$ parameters is derived from one with $\mathcal{N}$ parameters by removing the "least important" terms. To identify these terms, we start with the optimized solution containing $\mathcal{N}$ parameters, denoted by $\{\lambda'_\alpha\}$. We then compute the cost function (without further optimization) by successively setting each of the $\mathcal{N}$ parameters to zero

$$\mathcal{C}'_\alpha = \mathcal{C}\left(\{\lambda_\alpha\}\right) \quad \text{with} \quad \lambda_\beta = \lambda'_\beta \quad \text{for} \quad \beta \neq \alpha \quad \text{and} \quad \lambda_\alpha = 0. \tag{S9.296}$$

The term α' corresponding to the largest value $\mathcal{C}'_{\alpha'}$ is then eliminated. The model with $\mathcal{N}-1$ parameters is optimized, resulting in an optimal solution with one fewer parameter. This process is repeated until only one parameter remains. The result is $\mathcal{N}$ models, ranging from one parameter to $\mathcal{N}$ parameters. The model with the fewest parameters and an error below a certain threshold is then selected.

Finally, we also define the relative fitting error for both the linear and nonlinear fitting methods. First, we add a chemical potential term to the *ab initio* Hamiltonian

$$h^{\text{DFT}}\left(\mathbf{k}\right) \to h^{\text{DFT}}\left(\mathbf{k}\right) + \mu \mathbb{1}, \tag{S9.297}$$

with $\mu \in \mathbb{R}$, such that

$$\sum_{\mathbf{k}\in\mathcal{M}} \operatorname{Tr}\left(h^{\text{DFT}}\left(\mathbf{k}\right)\right) = 0, \tag{S9.298}$$

for the linear extraction method, or

$$\sum_{\mathbf{k}\in\mathcal{M}} \operatorname{Tr}\left(P^{\text{DFT}}\left(\mathbf{k}\right) h^{\text{DFT}}\left(\mathbf{k}\right)\right) = 0, \tag{S9.299}$$

in the nonlinear fitting method. The relative error (for a given optimized solution $\{\lambda'_\alpha\}$) is then defined as

$$\varepsilon = \frac{\mathcal{C}\left(\{\lambda_\alpha\}\right)\big|_{\lambda_\alpha=\lambda'_\alpha}}{\mathcal{C}\left(\{\lambda_\alpha\}\right)\big|_{\lambda_\alpha=0}}. \tag{S9.300}$$

X. ANALYTICAL RESULTS FOR THE MOIRÉ CONTINUUM HAMILTONIAN

In this section, we derive a series of analytical approximations for the spectrum of the moiré Hamiltonian. To achieve this, we start with the three-parameter model of twisted AA-stacked $SnSe_2$ and ZrS_2 introduced in Section VI E 1. By projecting onto a small number of plane waves, we analytically determine the spectrum of the corresponding Hamiltonian at the high-symmetry momenta of the moiré BZ. These results can be readily generalized to the two-parameter model derived for the AB-stacked case in Section VI E 2. Finally, we briefly outline how these parameters can be *estimated* from the *ab initio* spectrum of AA-stacked $SnSe_2$.

A. Analytical results at high-symmetry momentum points

The AA-stacked single-particle moiré Hamiltonian is valley-diagonal, given by

$$\mathcal{H}^{\text{AA}} = \sum_{\eta=0}^{2} \mathcal{H}^{\text{AA}}_{\eta}, \tag{S10.301}$$

where, for the three-parameter AA-stacked model, the valley $\eta = 0$ Hamiltonian is expressed as

$$\begin{aligned}\mathcal{H}^{\text{AA}}_0 = &\sum_{l,s} \sum_{\mathbf{Q}\in\mathcal{Q}_l} \hat{c}^\dagger_{\mathbf{k},\mathbf{Q},s,l} \hat{c}_{\mathbf{k},\mathbf{Q},s,l} \left[\frac{\left(k_x - Q_x\right)^2}{2m_x} + \frac{\left(k_y - Q_y\right)^2}{2m_y}\right] \\ &+ \sum_{l,s} \sum_{\substack{\mathbf{Q}\in\mathcal{Q}_l \\ \mathbf{Q}'\in\mathcal{Q}_{-l}}} \left[\left(iw^{\text{AA}}_1 + w^{\text{AA}}_2\right)\delta_{\mathbf{Q}+\mathbf{q}_0,\mathbf{Q}'} + \left(-iw^{\text{AA}}_1 + w^{\text{AA}}_2\right)\delta_{\mathbf{Q}-\mathbf{q}_0,\mathbf{Q}'}\right.\end{aligned}$$

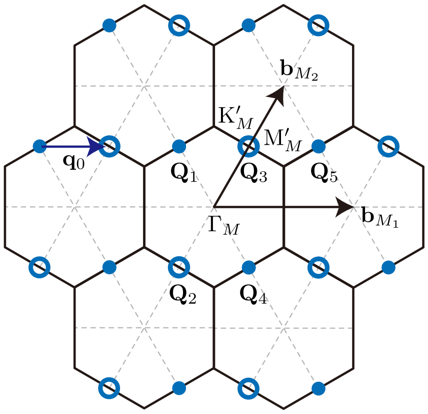

FIG. S24. Plane-wave states and high-symmetry points considered in the analytical models. The high-symmetry points of the moiré BZ defined in Eq. (S10.304) and analyzed in this section are shown, along with the $\mathbf{Q}$-points specified in Eq. (S10.305).

$$+w_3'^{\mathrm{AA}}\left(\delta_{\mathbf{Q}+\mathbf{q}_2-\mathbf{q}_1,\mathbf{Q}'}+\delta_{\mathbf{Q}-\mathbf{q}_2+\mathbf{q}_1,\mathbf{Q}'}\right)\Big]\hat{c}^\dagger_{\mathbf{k},\mathbf{Q},s,l}\hat{c}_{\mathbf{k},\mathbf{Q}',s,-l} \tag{S10.302}$$

and

$$\mathcal{H}^{\mathrm{AA}}_{\eta}=C_{3z}^{\eta}\mathcal{H}^{\mathrm{AA}}_0C_{3z}^{-\eta}. \tag{S10.303}$$

In the analysis that follows, we focus exclusively on the Hamiltonian in the $\eta=0$ valley, as the spectra for the other valleys can be obtained using C_{3z} symmetry.

We now consider the following high-symmetry momenta of the moiré BZ

$$\mathbf{k}_{\Gamma_M}=(0,0)\,,\quad \mathbf{k}_{\mathrm{K}'_M}=\left(0,\frac{2}{\sqrt{3}}\right)|\mathbf{q}_0|,\quad \mathbf{k}_{\mathrm{M}'_M}=\left(\frac{1}{2},\frac{\sqrt{3}}{2}\right)|\mathbf{q}_0|. \tag{S10.304}$$

These momenta are labeled in Fig. S24. Given the symmetries of the three-parameter AA-stacked model, we will now show that these are the only three high-symmetry points, as all others are related to these by the symmetries of the system. There are two inequivalent K_M points, which are related to one another by time-reversal symmetry $\mathcal{T}$. Consequently, we can restrict our focus to only one of them, which, without loss of generality, we choose to be $\mathbf{k}_{\mathrm{K}'_M}$. Additionally, there are three inequivalent M_M points located at $\mathbf{q}_0$, $-\mathbf{q}_1$, and $-\mathbf{q}_2$. One of these points, $\mathbf{q}_0$, labeled as M_M in Fig. S15(c), is related to Γ_M via the momentum-space non-symmorphic symmetry $\tilde{M}_z$ in valley $\eta=0$ and thus does not need to be considered. The remaining two points are related by C_{2x} symmetry, so we choose to focus on $\mathbf{k}_{\mathrm{M}'_M}=-\mathbf{q}_2$ without loss of generality.

To obtain the spectrum around the high-symmetry points defined in Eq. (S10.304), we employ a strategy similar to the tripod model [1] and its generalizations [6] for TBG. For each high-symmetry point, we include only those plane wave states corresponding to the $\mathbf{Q}$-points nearest to the respective high-symmetry points. To this end, we define the following $\mathbf{Q}$-points

$$\begin{gathered}\mathbf{Q}_1=\left(-\frac{1}{2},\frac{\sqrt{3}}{2}\right)|\mathbf{q}_0|,\quad \mathbf{Q}_2=\left(-\frac{1}{2},-\frac{\sqrt{3}}{2}\right)|\mathbf{q}_0|,\quad \mathbf{Q}_3=\left(\frac{1}{2},\frac{\sqrt{3}}{2}\right)|\mathbf{q}_0|,\\ \mathbf{Q}_4=\left(\frac{1}{2},-\frac{\sqrt{3}}{2}\right)|\mathbf{q}_0|,\quad \mathbf{Q}_5=\left(\frac{3}{2},\frac{\sqrt{3}}{2}\right)|\mathbf{q}_0|,\end{gathered} \tag{S10.305}$$

which are also shown in Fig. S24.

1. Γ_M point

To analytically obtain the spectrum of the Hamiltonian at the Γ_M point, we project the three-parameter model Hamiltonian onto the plane wave states corresponding to the four closest $\mathbf{Q}$-points. Defining the single-particle states,

$$\left|\phi^{\Gamma_M}_{1,s}(\mathbf{p})\right\rangle=\hat{c}^\dagger_{\mathbf{p},\mathbf{Q}_1,s,+}\left|0\right\rangle,\quad \left|\phi^{\Gamma_M}_{2,s}(\mathbf{p})\right\rangle=\hat{c}^\dagger_{\mathbf{p},\mathbf{Q}_2,s,-}\left|0\right\rangle,$$

$$\left|\phi_{3,s}^{\Gamma_M}(\mathbf{p})\right\rangle = \hat{c}^\dagger_{\mathbf{p},\mathbf{Q}_3,s,-}\left|0\right\rangle, \quad \left|\phi_{4,s}^{\Gamma_M}(\mathbf{p})\right\rangle = \hat{c}^\dagger_{\mathbf{p},\mathbf{Q}_4,s,+}\left|0\right\rangle, \tag{S10.306}$$

the corresponding "quadropod" Hamiltonian matrix is given by

$$H^{\Gamma_M}_{is,js'}(\mathbf{p}) = \left\langle \phi_{i,s}^{\Gamma_M}(\mathbf{p})\middle|\mathcal{H}_0^{\mathrm{AA}}\middle|\phi_{j,s'}^{\Gamma_M}(\mathbf{p})\right\rangle, \tag{S10.307}$$

where

$$H^{\Gamma_M}(\mathbf{p}) = \begin{pmatrix} \epsilon^0_{\mathbf{p}-\mathbf{Q}_1} & w_3'^{\mathrm{AA}} & iw_1^{\mathrm{AA}} + w_2^{\mathrm{AA}} & 0 \\ w_3'^{\mathrm{AA}} & \epsilon^0_{\mathbf{p}-\mathbf{Q}_2} & 0 & iw_1^{\mathrm{AA}} + w_2^{\mathrm{AA}} \\ -iw_1^{\mathrm{AA}} + w_2^{\mathrm{AA}} & 0 & \epsilon^0_{\mathbf{p}-\mathbf{Q}_3} & w_3'^{\mathrm{AA}} \\ 0 & -iw_1^{\mathrm{AA}} + w_2^{\mathrm{AA}} & w_3'^{\mathrm{AA}} & \epsilon^0_{\mathbf{p}-\mathbf{Q}_4} \end{pmatrix} \otimes s_0. \tag{S10.308}$$

In Eq. (S10.308), we have introduced

$$\epsilon^0_{\mathbf{k}} = \frac{k_x^2}{2m_x} + \frac{k_y^2}{2m_y}. \tag{S10.309}$$

At the Γ_M point ($\mathbf{p} = \mathbf{0}$), the eigenvalues of $H^{\Gamma_M}(\mathbf{p})$, each two-fold degenerate due to spin, are

$$\begin{aligned} E^1_{\Gamma_M} &= \epsilon^0_{-\mathbf{Q}_1} - \left|w_3'^{\mathrm{AA}}\right| - \sqrt{\left(w_1^{\mathrm{AA}}\right)^2 + \left(w_2^{\mathrm{AA}}\right)^2}, \\ E^2_{\Gamma_M} &= \epsilon^0_{-\mathbf{Q}_1} + \left|w_3'^{\mathrm{AA}}\right| - \sqrt{\left(w_1^{\mathrm{AA}}\right)^2 + \left(w_2^{\mathrm{AA}}\right)^2}, \\ E^3_{\Gamma_M} &= \epsilon^0_{-\mathbf{Q}_1} - \left|w_3'^{\mathrm{AA}}\right| + \sqrt{\left(w_1^{\mathrm{AA}}\right)^2 + \left(w_2^{\mathrm{AA}}\right)^2}, \\ E^4_{\Gamma_M} &= \epsilon^0_{-\mathbf{Q}_1} + \left|w_3'^{\mathrm{AA}}\right| + \sqrt{\left(w_1^{\mathrm{AA}}\right)^2 + \left(w_2^{\mathrm{AA}}\right)^2}, \end{aligned} \tag{S10.310}$$

with

$$\epsilon^0_{-\mathbf{Q}_1} = \left(\frac{1}{8m_x} + \frac{3}{8m_y}\right)\left|\mathbf{q}_0\right|^2. \tag{S10.311}$$

The *ab initio* values of w_1^{AA} and w_2^{AA}, which correspond to the "hopping" between the nearest-neighbor **Q**-points, represent the largest parameters. These dominant w_1^{AA} and w_2^{AA} terms split the four bands at Γ_M into two sets: $E^{1,2}_{\Gamma_M}$ and $E^{3,4}_{\Gamma_M}$, with the gap between the two sets of bands approximately $2\sqrt{\left(w_1^{\mathrm{AA}}\right)^2 + \left(w_2^{\mathrm{AA}}\right)^2}$. The $w_3'^{\mathrm{AA}}$ term further splits the spectrum into four spin-degenerate bands. The energy difference between $E^1_{\Gamma_M}$ ($E^3_{\Gamma_M}$) and $E^2_{\Gamma_M}$ ($E^4_{\Gamma_M}$) is $2\left|w_3'^{\mathrm{AA}}\right|$.

2. K'_M *point*

We now discuss the Hamiltonian at the K'_M point. We consider only the two **Q**-points closest to the K'_M point, corresponding to the following states

$$\left|\phi_{1,s}^{\mathrm{K}'_M}(\mathbf{p})\right\rangle = \hat{c}^\dagger_{\mathbf{p}+\mathbf{k}_{\mathrm{K}'_M},\mathbf{Q}_1,s,+}\left|0\right\rangle, \quad \left|\phi_{2,s}^{\mathrm{K}'_M}(\mathbf{p})\right\rangle = \hat{c}^\dagger_{\mathbf{p}+\mathbf{k}_{\mathrm{K}'_M},\mathbf{Q}_3,s,-}\left|0\right\rangle, \tag{S10.312}$$

where $\mathbf{Q}_1$ and $\mathbf{Q}_3$ are defined in Eq. (S10.304). The corresponding single-particle Hamiltonian is written as

$$H^{\mathrm{K}'_M}_{is,js'}(\mathbf{p}) = \left\langle \phi_{i,s}^{\mathrm{K}'_M}(\mathbf{p})\middle|\mathcal{H}_0^{\mathrm{AA}}\middle|\phi_{j,s'}^{\mathrm{K}'_M}(\mathbf{p})\right\rangle, \tag{S10.313}$$

where

$$H^{\mathrm{K}'_M}(\mathbf{p}) = \begin{pmatrix} \epsilon^0_{\mathbf{k}_{\mathrm{K}'_M}+\mathbf{p}-\mathbf{Q}_1} & iw_1^{\mathrm{AA}} + w_2^{\mathrm{AA}} \\ -iw_1^{\mathrm{AA}} + w_2^{\mathrm{AA}} & \epsilon^0_{\mathbf{k}_{\mathrm{K}'_M}+\mathbf{p}-\mathbf{Q}_2} \end{pmatrix} \otimes s_0. \tag{S10.314}$$

The Hamiltonian can be directly diagonalized, yielding the eigenvalues

$$E^1_{\mathrm{K}'_M}(\mathbf{p}) = \frac{m_x|\mathbf{q}_0|^2 + 3m_y\left(|\mathbf{q}_0|^2 + 4p_x^2\right) + 4m_x p_y\left(\sqrt{3}|\mathbf{q}_0| + 3p_y\right)}{24m_x m_y} - \frac{1}{2}\sqrt{\frac{p_x^2|\mathbf{q}_0|^2}{m_x^2} + 4\left(w_1^{\mathrm{AA}}\right)^2 + 4\left(w_2^{\mathrm{AA}}\right)^2}$$

$$E^1_{\mathrm{K}'_M}(\mathbf{0}) + \frac{p_y|\mathbf{q}_0|}{4\sqrt{3}m_x} + \frac{p_y^2}{4m_x} + \frac{p_x^2}{8m_x}\left[4 - \frac{|\mathbf{q}_0|^2}{m_x\sqrt{\left(w_1^{\mathrm{AA}}\right)^2 + \left(w_2^{\mathrm{AA}}\right)^2}}\right], \tag{S10.315}$$

$$E^2_{\mathrm{K}'_M}(\mathbf{p}) = \frac{m_x|\mathbf{q}_0|^2 + 4m_x p_y\left(\sqrt{3}|\mathbf{q}_0| + 3p_y\right) + 3m_y|\mathbf{q}_0|^2 + 12m_y p_x^2}{24m_x m_y} + \frac{1}{2}\sqrt{\frac{p_x^2|\mathbf{q}_0|^2}{m_x^2} + 4\left(w_1^{\mathrm{AA}}\right)^2 + 4\left(w_2^{\mathrm{AA}}\right)^2}$$

$$\approx E^1_{\mathrm{K}'_M}(\mathbf{0}) + \frac{p_y|\mathbf{q}_0|}{4\sqrt{3}m_x} + \frac{p_y^2}{4m_x} + \frac{p_x^2}{8m_x}\left[4 + \frac{|\mathbf{q}_0|^2}{m_x\sqrt{\left(w_1^{\mathrm{AA}}\right)^2 + \left(w_2^{\mathrm{AA}}\right)^2}}\right], \tag{S10.316}$$

with

$$E^1_{\mathrm{K}'_M}(\mathbf{0}) = \frac{m_x|\mathbf{q}_0|^2 + 3m_y|\mathbf{q}_0|^2}{24m_x m_y} - \sqrt{\left(w_1^{\mathrm{AA}}\right)^2 + \left(w_2^{\mathrm{AA}}\right)^2}, \tag{S10.317}$$

$$E^1_{\mathrm{K}'_M}(\mathbf{0}) = \frac{m_x|\mathbf{q}_0|^2 + 3m_y|\mathbf{q}_0|^2}{24m_x m_y} + \sqrt{\left(w_1^{\mathrm{AA}}\right)^2 + \left(w_2^{\mathrm{AA}}\right)^2}, \tag{S10.318}$$

exactly at the K'_M point ($\mathbf{p} = \mathbf{0}$). The energy difference between the two lowest bands is proportional to the nearest-neighbor hopping amplitude on the $\mathbf{Q}$-lattice, given by $2\sqrt{\left(w_1^{\mathrm{AA}}\right)^2 + \left(w_2^{\mathrm{AA}}\right)^2}$. The eigenvectors at $\mathbf{p} = \mathbf{0}$ are

$$v^1_{\mathrm{K}'_M}(\mathbf{0}) \propto \begin{pmatrix} \frac{-iw_1^{\mathrm{AA}} - w_2^{\mathrm{AA}}}{\sqrt{\left(w_1^{\mathrm{AA}}\right)^2 + \left(w_2^{\mathrm{AA}}\right)^2}} & 1 \end{pmatrix}, \quad v^2_{\mathrm{K}'_M}(\mathbf{0}) \propto \begin{pmatrix} \frac{iw_1^{\mathrm{AA}} + w_2^{\mathrm{AA}}}{\sqrt{\left(w_1^{\mathrm{AA}}\right)^2 + \left(w_2^{\mathrm{AA}}\right)^2}} & 1 \end{pmatrix}. \tag{S10.319}$$

As discussed in the main text and further in Section XI, the lowest set of bands exhibits flat dispersion along the $\hat{\mathbf{x}}$ direction in valley $\eta = 0$ for $SnSe_2$, due to the $\tilde{M}_z$ symmetry and orbital shape. The dispersion along the $\hat{\mathbf{y}}$ direction can be estimated by comparing the energy difference of the lowest band between the Γ_M and K'_M points

$$E^1_{\Gamma_M}(\mathbf{0}) - E^1_{\mathrm{K}'_M}(\mathbf{0}) = \frac{|\mathbf{q}_0|^2}{8m_x} + \frac{3|\mathbf{q}_0|^2}{8m_y} - \left|w_3'^{\mathrm{AA}}\right| - \sqrt{\left(w_1^{\mathrm{AA}}\right)^2 + \left(w_2^{\mathrm{AA}}\right)^2} - \left[\frac{|\mathbf{q}_0|^2}{24m_y} - \frac{|\mathbf{q}_0|^2}{8m_x} - \sqrt{\left(w_1^{\mathrm{AA}}\right)^2 + \left(w_2^{\mathrm{AA}}\right)^2}\right]$$

$$= \frac{|\mathbf{q}_0|^2}{3m_y} - \left|w_3'^{\mathrm{AA}}\right|. \tag{S10.320}$$

3. M'_M *point*

Finally, we discuss the Hamiltonian at the M'_M point. Since the M'_M point is located exactly at the $\mathbf{Q}_3$ point, considering only the nearest $\mathbf{Q}$-point would result in the energy of the lowest band being independent of any parameters of the moiré potential and equal to zero. To capture its leading nontrivial dependence on the moiré potential parameters, we include the next-nearest $\mathbf{Q}$-points. The associated plane-wave states are given by

$$\left|\phi^{\mathrm{M}'_M}_{1,s}(\mathbf{p})\right\rangle = \hat{c}^\dagger_{\mathbf{p}+\mathbf{k}_{\mathrm{M}'_M},\mathbf{Q}_1,s,+}|0\rangle, \quad \left|\phi^{\mathrm{M}'_M}_{2,s}(\mathbf{p})\right\rangle = \hat{c}^\dagger_{\mathbf{p}+\mathbf{k}_{\mathrm{M}'_M},\mathbf{Q}_3,s,-}|0\rangle, \quad \left|\phi^{\mathrm{M}'_M}_{3,s}(\mathbf{p})\right\rangle = \hat{c}^\dagger_{\mathbf{p}+\mathbf{k}_{\mathrm{M}'_M},\mathbf{Q}_5,s,+}|0\rangle, \tag{S10.321}$$

where $\mathbf{Q}_1$, $\mathbf{Q}_3$, and $\mathbf{Q}_5$ were defined in Eq. (S10.304). The corresponding single-particle Hamiltonian can be expressed as

$$H^{\mathrm{M}'_M}_{is,js'}(\mathbf{p}) = \left\langle \phi^{\mathrm{M}'_M}_{i,s}(\mathbf{p})\middle|\mathcal{H}_0^{\mathrm{AA}}\middle|\phi^{\mathrm{M}'_M}_{j,s'}(\mathbf{p})\right\rangle, \tag{S10.322}$$

where

$$H^{\mathrm{M}'_M}(\mathbf{p}) = \begin{pmatrix} \epsilon^0_{\mathbf{p}+M-\mathbf{Q}_1} & iw_1^{\mathrm{AA}} + w_2^{\mathrm{AA}} & 0 \\ -iw_1^{\mathrm{AA}} + w_2^{\mathrm{AA}} & \epsilon^0_{\mathbf{p}+M-\mathbf{Q}_3} & iw_1^{\mathrm{AA}} + w_2^{\mathrm{AA}} \\ 0 & -iw_1^{\mathrm{AA}} + w_2^{\mathrm{AA}} & \epsilon^0_{\mathbf{p}+M-\mathbf{Q}_5} \end{pmatrix} \otimes s_0. \tag{S10.323}$$

At the M'_M point ($\mathbf{p}=\mathbf{0}$), the eigenvalues are

$$E^1_{\mathrm{M}'_M}(\mathbf{0}) = \frac{|\mathbf{q}_0|^2}{4m_x} - \sqrt{\frac{|\mathbf{q}_0|^4}{16m_x^2} + 2\left(\left(w_1^{\mathrm{AA}}\right)^2 + \left(w_2^{\mathrm{AA}}\right)^2\right)},$$
$$E^2_{\mathrm{M}'_M}(\mathbf{0}) = \frac{|\mathbf{q}_0|^2}{2m_x},$$
$$E^3_{\mathrm{M}'_M}(\mathbf{0}) = \frac{|\mathbf{q}_0|^2}{4m_x} + \sqrt{\frac{|\mathbf{q}_0|^4}{16m_x^2} + 2\left(\left(w_1^{\mathrm{AA}}\right)^2 + \left(w_2^{\mathrm{AA}}\right)^2\right)}. \tag{S10.324}$$

and the corresponding eigenvectors are

$$v^1_{\mathrm{M}'_M}(\mathbf{0}) \propto \left(-2\sqrt{2}(iw_1^{\mathrm{AA}} + w_2^{\mathrm{AA}}) \;\; \frac{1}{2}\left(\frac{\mathbf{q}_0^2}{m_x} + \sqrt{\frac{|\mathbf{q}_0|^4}{m_x^2} + 32\left(w_1^{\mathrm{AA}}\right)^2 + 32\left(w_2^{\mathrm{AA}}\right)^2}\right) \;\; 2\sqrt{2}(iw_1^{\mathrm{AA}} - w_2^{\mathrm{AA}})\right),$$
$$v^2_{\mathrm{M}'_M}(\mathbf{0}) \propto \left(\frac{w_1^{\mathrm{AA}} - iw_2^{\mathrm{AA}}}{\sqrt{\left(w_1^{\mathrm{AA}}\right)^2 + \left(w_2^{\mathrm{AA}}\right)^2}} \;\; 0 \;\; \frac{w_1^{\mathrm{AA}} + iw_2^{\mathrm{AA}}}{\sqrt{\left(w_1^{\mathrm{AA}}\right)^2 + \left(w_2^{\mathrm{AA}}\right)^2}}\right),$$
$$v^3_{\mathrm{M}'_M}(\mathbf{0}) \propto \left(2\sqrt{2}(iw_1^{\mathrm{AA}} + w_2^{\mathrm{AA}}) \;\; \frac{1}{2}\left(\frac{\mathbf{q}_0^2}{m_x} - \sqrt{\frac{|\mathbf{q}_0|^4}{m_x^2} + 32\left(w_1^{\mathrm{AA}}\right)^2 + 32\left(w_2^{\mathrm{AA}}\right)^2}\right) \;\; 2\sqrt{2}(iw_1^{\mathrm{AA}} - w_2^{\mathrm{AA}})\right). \tag{S10.325}$$

4. Extending to the two-parameter AB-stacked model

The results derived above can be straightforwardly extended to the two-parameter AB-stacked model introduced in Section VI E 2 by applying the following substitutions

$$w_1^{\mathrm{AA}} \to 0, \quad w_2^{\mathrm{AA}} \to w_2^{\mathrm{AB}}, \quad w_3'^{\mathrm{AA}} \to w_4'^{\mathrm{AB}}. \tag{S10.326}$$

B. Extracting parameters

Before performing a comprehensive parameter extraction or fitting, as detailed in Section XI, it is useful to estimate the values of the moiré potential parameters by comparing the analytical results from Section X A with the numerical *ab initio* results for AA-stacked $\mathrm{SnSe_2}$. Additionally, we will compare the parameters estimated in this section with the fitted parameters provided in Section XI.

1. Energy gaps

We focus on the Γ_M point. The eigenvalues of the analytical Hamiltonian at Γ_M are given in Eq. (S10.310). Considering the *ab initio* spectrum at $\theta = 3.89°$, we restrict our analysis to the lowest three *ab initio* bands, as their weights are primarily located at the four $\mathbf{Q}$-points nearest to the Γ_M point ($\mathbf{Q}_1$, $\mathbf{Q}_2$, $\mathbf{Q}_3$, and $\mathbf{Q}_4$). The corresponding energies are

$$E^1_{\mathrm{DFT},\Gamma_M} = 0\,\mathrm{meV}, \quad E^2_{\mathrm{DFT},\Gamma_M} = 41.3\,\mathrm{meV}, \quad E^3_{\mathrm{DFT},\Gamma_M} = 160.3\,\mathrm{meV}. \tag{S10.327}$$

Inspecting Eq. (S10.310), it is evident that the $w_3'^{\mathrm{AA}}$ parameter can be determined as

$$\left|w_3'^{\mathrm{AA}}\right| = \frac{1}{2}\left(E^2_{\mathrm{DFT},\Gamma_M} - E^1_{\mathrm{DFT},\Gamma_M}\right) = 20.6\,\mathrm{meV} \tag{S10.328}$$

The two lowest-energy states of the simplified model have spinless C_{2x} eigenvalues $-\operatorname{sgn}\left(w_3'^{\mathrm{AA}}\right)$ and $\operatorname{sgn}\left(w_3'^{\mathrm{AA}}\right)$, respectively. From the *ab initio* simulations, we observe that the lowest-energy states have a C_{2x} eigenvalue of $+1$, indicating $w_3'^{\mathrm{AA}} < 0$. Additionally, the current parameters indicate that the band is also approximately flat along the $\hat{\mathbf{y}}$ direction, as derived in Eq. (S10.320), with $|\mathbf{q}_0|^2/\left(3m_y\left|w_3'^{\mathrm{AA}}\right|\right) \approx 0.7$.

In addition, we also have

$$\sqrt{\left(\left(w_1^{\mathrm{AA}}\right)^2 + \left(w_2^{\mathrm{AA}}\right)^2\right)} = \frac{1}{2}\left(E^3_{\Gamma_M} - E^1_{\Gamma_M}\right). \tag{S10.329}$$

which leads to

$$\sqrt{\left(\left(w_1^{\mathrm{AA}}\right)^2+\left(w_2^{\mathrm{AA}}\right)^2\right)}=\frac{1}{2}\left(E^3_{\mathrm{DFT},\Gamma_M}-E^1_{\mathrm{DFT},\Gamma_M}\right)=80.15\,\mathrm{meV}. \tag{S10.330}$$

Thus, we extract the values of $w_3^{\prime\mathrm{AA}}$ and $\sqrt{\left(w_1^{\mathrm{AA}}\right)^2+\left(w_2^{\mathrm{AA}}\right)^2}$ directly from the *ab initio* spectrum. In the next Section X B 2, we determine the ratio $w_1^{\mathrm{AA}}/w_2^{\mathrm{AA}}$ by analyzing the wave functions of the bands obtained from the *ab initio* calculations.

2. *Wave functions*

At the Γ_M point, the eigenvector of the lowest band obtained from *ab initio* simulations *projected* onto the basis defined in Eq. (S10.308) is given by

$$v^1_{\Gamma_M}=\begin{pmatrix}0.45e^{-i0.86\pi} & 0.45e^{-i0.86\pi} & 0.45 & 0.45\end{pmatrix}. \tag{S10.331}$$

We note that the projected eigenvector is not normalized, as it includes only the weight at the four nearest-neighbor $\mathbf{Q}$ points. The analytical wave function of the lowest band is expressed as

$$v^1_{\Gamma_M}=\frac{1}{2}\begin{pmatrix}\frac{-iw_1^{\mathrm{AA}}-w_2^{\mathrm{AA}}}{\sqrt{\left(w_1^{\mathrm{AA}}\right)^2+\left(w_2^{\mathrm{AA}}\right)^2}} & \frac{-iw_1^{\mathrm{AA}}-w_2^{\mathrm{AA}}}{\sqrt{\left(w_1^{\mathrm{AA}}\right)^2+\left(w_2^{\mathrm{AA}}\right)^2}} & 1 & 1\end{pmatrix}. \tag{S10.332}$$

By fitting the phase factor, we obtain

$$\arg\left(iw_1^{\mathrm{AA}}+w_2^{\mathrm{AA}}\right)=0.14\pi. \tag{S10.333}$$

Next, we investigate the K'_M point. The wave function obtained from the *ab initio* calculations, projected onto the basis defined in Eq. (S10.313), is

$$v^1_{\mathrm{K}'_M}=\begin{pmatrix}0.59e^{-i0.72\pi} & 0.59\end{pmatrix}. \tag{S10.334}$$

Analytically, the corresponding wave function is given in Eq. (S10.319). By matching the phase factor in the *ab initio* wave function, we find

$$\arg\left(iw_1^{\mathrm{AA}}+w_2^{\mathrm{AA}}\right)=0.28\pi. \tag{S10.335}$$

Finally, we consider the M'_M point. The *ab initio* wave function of the lowest band, projected onto the basis of Eq. (S10.322), is

$$v^1_{\mathrm{M}'_M}=\begin{pmatrix}0.43e^{-i1.26\pi} & i0.67 & 0.43e^{i0.26\pi}\end{pmatrix}. \tag{S10.336}$$

By comparing the phase factors of the wave functions between *ab initio* and analytical calculations from Eq. (S10.325), we determine

$$\arg\left(iw_1^{\mathrm{AA}}+w_2^{\mathrm{AA}}\right)=0.24\pi. \tag{S10.337}$$

In summary, the phase $\arg\left(iw_1^{\mathrm{AA}}+w_2^{\mathrm{AA}}\right)$ obtained from the Γ_M, K'_M, and M'_M points is 0.14π, 0.28π, and 0.24π, respectively. The deviations may arise due to the simplifications made when deriving the analytical model (*i.e.*, only considering a small set of $\mathbf{Q}$ points). The phase factor of $iw_1^{\mathrm{AA}}+w_2^{\mathrm{AA}}$ is determined by averaging the extracted values from the three high-symmetry points

$$\arg\left(iw_1^{\mathrm{AA}}+w_2^{\mathrm{AA}}\right)=\frac{1}{3}\left(0.14+0.28+0.24\right)\pi\approx 0.22\pi. \tag{S10.338}$$

We adopt the same procedure to estimate parameters at $\theta=3.89°$, $\theta=4.41°$, $\theta=5.09°$, and $\theta=7.34°$. Additionally, we use the masses m_x and m_y obtained from the monolayer *ab initio* calculations in Eq. (S2.16). The results are shown in Table S5, which also includes the minimum overlap between the wave function of the lowest band of the three-parameter model and that obtained in *ab initio* simulations along the Γ_M - M_M - K_M - Γ_M line. The results show that the continuum model can reproduce the lowest band well.

In Fig. S25, we also compare the dispersion obtained from the corresponding three-parameter continuum model and the *ab initio* result. Furthermore, the parameters extracted from the analytical models qualitatively reproduce the fitting results presented in Table S12 for $\theta=3.89°$, $\theta=4.41°$, and $\theta=5.09°$. However, for $\theta=7.34°$, the best three-parameter continuum model is obtained with nonzero w_1^{AA}, w_2^{AA}, and $w_1^{\prime\mathrm{AA}}$ in Table S12, instead of the nonzero w_1^{AA}, w_2^{AA}, and $w_3^{\prime\mathrm{AA}}$ employed here.

θ	$\sqrt{(w_1^{AA})^2 + (w_2^{AA})^2}$/meV	$\arg\left(iw_1^{AA} + w_2^{AA}\right)/\pi$ (Γ_M, M'_M, K'_M)	w_1^{AA}/meV	w_2^{AA}/meV	$w_3'^{AA}$/meV	Overlap
7.34°	86.2	0.22 (0.16, 0.28, 0.24)	54.9	66.4	−13.1	0.98
5.09°	91.7	0.25 (0.15, 0.28, 0.24)	64.8	64.8	−18.7	0.97
4.41°	67.9	0.27 (0.37, 0.23, 0.24)	62.3	27.0	−20.2	0.93
3.89°	80.1	0.22 (0.14, 0.28, 0.24)	51.1	61.7	−20.6	0.96

TABLE S5. Estimated continuum model parameters for AA-stacked $SnSe_2$ based on the simplified analytical models discussed in this section. The third column shows the fitted phase factor $\arg\left(iw_1^{AA} + w_2^{AA}\right)/\pi$ averaged across the Γ_M, M'_M, and K'_M points, along with the individual values at each point in parentheses. The last column provides the minimum overlap between the wave function of the lowest band in the continuum model and the corresponding *ab initio* simulation along the Γ-M_M-K_M-Γ path.

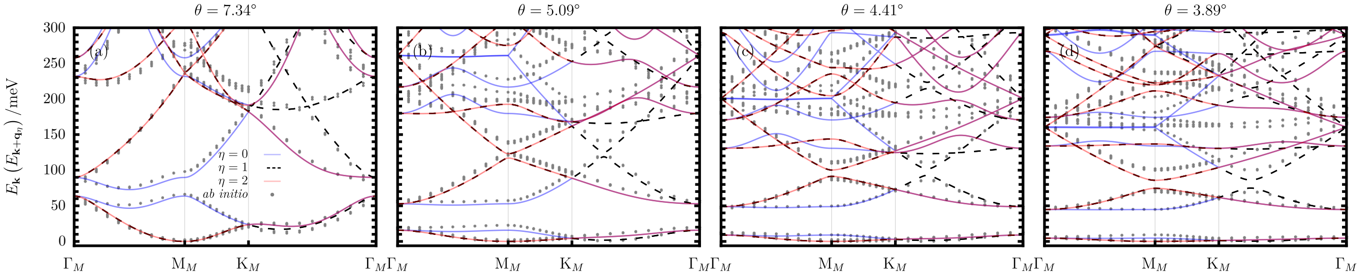

FIG. S25. Dispersion of the three-parameter continuum model with parameters given in Table S5. The blue, dashed, and red lines represent the dispersion of the continuum model, while the gray dots indicate the *ab initio* dispersion. The twist angle is indicated above each panel.

XI. MODELS FITTED TO *AB INITIO* SIMULATIONS

This section presents detailed results on the band structure of twisted $SnSe_2$ and ZrS_2 bilayers. We begin by offering an overview of the key findings in this section, which include the construction of various models and their comparison with the *ab initio* spectra. We also present results on the CDD, Berry curvature, and Wilson loops of the models. Finally, we provide a detailed, point-by-point discussion of the main trends observed in the *ab initio* simulations.

A. Overview of the results

Starting with the *ab initio* band structure computed as detailed in Section III, we derive the plane-wave valley-projected Hamiltonian using the method outlined in Section VIII B. From there, we construct a series of continuum models that *accurately* reproduce the *ab initio* spectrum of the system. The methods described in Section IX are used to construct these continuum Hamiltonians. We then analyze their spectra across the full range of monolayer materials, stacking configurations, and commensurate angles considered in this work. A summary of all the results (detailed below) is provided in Table S6.

1. *Types of models constructed*

We find it useful to construct several types of continuum models with varying levels of complexity. These models include numerically *exact* ones that accurately match both the dispersion and wave functions of the first five bands, with an overlap of more than 0.999 for the first band and over 0.95 for the first five. To gain analytical insight and explore the *emergent* symmetries of the system, we also develop simplified models, beginning with the first moiré harmonic Hamiltonian presented in Eqs. (S5.94) and (S5.95), and progressively reduce the number of parameters. The five types of models we consider, in order of *decreasing* complexity, are:

1. **The full model**: The full model is constructed using the linear-fitting method outlined in Section IX B 2. We first obtain the symmetry-obeying parameterization of the moiré Hamiltonian from Eq. (S7.205), which includes gradient terms and higher-order harmonics in the moiré potential. For the moiré Hamiltonian $h_{\mathbf{Q},\mathbf{Q}'}(\mathbf{k})$, we consider moiré harmonics with $|\mathbf{Q} - \mathbf{Q}'| \leq 4|\mathbf{b}_{M_1}|$, and include gradient terms up to degree $n_x + n_y = 4$

$(n_x + n_y = 10)$ for the $\mathbf{Q} \neq \mathbf{Q}'$ ($\mathbf{Q} = \mathbf{Q}'$) terms. As described in Section IX B 2, the $\mathbf{Q}$-off-diagonal terms are fitted using an energy weighting function of the form given in Eq. (S9.271). The parameters used for the weighting function are chosen to capture the first four or five sets of spinful bands within each valley and are listed in Section XI C. The effective masses of the monolayer are treated as fitting parameters. We reduce the number of parameters in the moiré Hamiltonian, initially exceeding a thousand, by systematically removing the least significant ones using the algorithm outlined in Section IX D. The final model retains the smallest number of parameters, ensuring that the relative error, as defined in Eq. (S9.300), remains below $\varepsilon = 0.02\%, 0.04\%, 0.02\%, 0.015\%$ for AA-stacked $SnSe_2$, AB-stacked $SnSe_2$, AA-stacked ZrS_2, and AB-stacked ZrS_2, respectively.

2. **The full first moiré harmonic model**: This model is constructed using the method outlined in Section IX C and employs the first moiré harmonic Hamiltonian from Eqs. (S5.94) and (S5.95), which includes 16 interlayer hopping parameters. The cost function from Eq. (S9.289) is constructed using the projectors into the first $\mathcal{N}_{\text{Band}} = 2$ ($\mathcal{N}_{\text{Band}} = 1$) sets of spinful bands within each valley for $\theta \leq 6.01°$ ($\theta > 6.01°$). The effective masses of the monolayer are also included as fitting parameters.

3. **The reduced first moiré Harmonic model**: This model is derived from the full first moiré harmonic model by reducing the number of interlayer hopping parameters to just five, using the step-wise reduction method described in Section IX D.

4. **The full first moiré harmonic model with the zero-twist constraints imposed**: This model is similar to the full first moiré harmonic model, but with the zero-twist constraints applied. To prevent issues related to overfitting, the effective masses of the monolayer are not treated as fitting parameters; instead, they are fixed to values extracted from the monolayer band structure in Section II. Additionally, the moiré potential parameters with the largest absolute values, specifically w_1^{AA} and w_2^{AA} (or w_2^{AB} for AB-stacking), are also fixed to their values obtained from the full model, rather than being treated as fitting parameters.

5. **The reduced first moiré harmonic model with the zero-twist constraints imposed**: This model is derived from the full first moiré harmonic model, with the zero-twist constraints applied. The number of interlayer hopping parameters is reduced to just three by using the step-wise reduction method described in Section IX D.

The Hamiltonians corresponding to the analytical models presented here have been deposited electronically in a public repository, as detailed in the main text.

Before analyzing the detailed spectrum of these five models, we establish a consistent notation for the wave functions and energy eigenvalues of the moiré Hamiltonian continuum models. Specifically, we denote by $\epsilon_{n,\eta}(\mathbf{k})$ and $u_{\mathbf{Q},s,l;\eta,n}(\mathbf{k})$ the energy and wave function, respectively, of the n-th conduction band ($n \geq 1$) in valley $0 \leq \eta \leq 2$, such that

$$\sum_{\substack{s',l' \\ \mathbf{Q}' \in \mathcal{Q}_{\text{tot}}}} \left[h_{\mathbf{Q},\mathbf{Q}'}(\mathbf{k})\right]_{sl;s'l'} u_{\mathbf{Q}',s',l';\eta,n}(\mathbf{k}) = \epsilon_{n,\eta}(\mathbf{k})\, u_{\mathbf{Q},s,l;\eta,n}(\mathbf{k}). \tag{S11.339}$$

The bands are indexed by n in the order of increasing energy at every moiré momentum. The corresponding band-basis fermion operators are defined as

$$\hat{c}^\dagger_{\mathbf{k},n,\eta} \equiv \sum_{\substack{s,l \\ \mathbf{Q} \in \mathcal{Q}_{\text{tot}}}} u_{\mathbf{Q},s,l;\eta,n}(\mathbf{k})\, \hat{c}^\dagger_{\mathbf{k},\mathbf{Q},s,l}. \tag{S11.340}$$

Due to the valley symmetry, $u_{\mathbf{Q},s,l;\eta,n}(\mathbf{k})$ is supported on only two of the three plane-wave sublattices, so we have

$$u_{\mathbf{Q},s,l;\eta,n}(\mathbf{k}) = 0, \quad \text{if} \quad \mathbf{Q} \notin \mathcal{Q}_{\eta+l}. \tag{S11.341}$$

Outside the first moiré BZ, the band wave functions are defined by the embedding condition

$$u_{\mathbf{Q},s,l;\eta,n}(\mathbf{k}+\mathbf{G}) = u_{\mathbf{Q}+\mathbf{G},s,l;\eta,n}(\mathbf{k}), \quad \text{for} \quad \mathbf{G} \in \mathcal{Q}. \tag{S11.342}$$

We also introduce the projector onto the m-th set of spinful bands in valley η as

$$P^{m,\eta}_{\mathbf{Q},s,l;\mathbf{Q}',s',l'}(\mathbf{k}) \equiv \sum_{n=2m-1}^{2m} u_{\mathbf{Q},s,l;\eta,n}(\mathbf{k})\, u^*_{\mathbf{Q}',s',l';\eta,n}(\mathbf{k}), \quad \text{for} \quad m \geq 1. \tag{S11.343}$$

Monolayer	Stacking	Models					θ	Spectrum	Additional results	Wilson loops
		1	2	3	4	5				
$SnSe_2$	AA	Table S7	Table S11	Table S12	Table S13	Table S14	9.43°	Fig. S26	Fig. S50	Figs. S86 and S110
							7.34°	Fig. S27	Fig. S51	Figs. S87 and S111
							6.01°	Fig. S28	Fig. S52	Figs. S88 and S112
							5.09°	Fig. S29	Figs. S53 and S54	Figs. S89 and S113
							4.41°	Fig. S30	Figs. S55 and S56	Figs. S90 and S114
							3.89°	Fig. S31	Figs. S57 and S58	Figs. S91 and S115
	AB	Table S8	Table S15	Table S16	Table S17	Table S18	9.43°	Fig. S32	Fig. S59	Figs. S92 and S116
							7.34°	Fig. S33	Fig. S60	Figs. S93 and S117
							6.01°	Fig. S34	Fig. S61	Figs. S94 and S118
							5.09°	Fig. S35	Fig. S62	Figs. S95 and S119
							4.41°	Fig. S36	Figs. S63 and S64	Figs. S96 and S120
							3.89°	Fig. S37	Figs. S65 and S66	Figs. S97 and S121
ZrS_2	AA	Table S9	Table S19	Table S20	Table S21	Table S22	9.43°	Fig. S38	Fig. S67	Figs. S98 and S122
							7.34°	Fig. S39	Fig. S68	Figs. S99 and S123
							6.01°	Fig. S40	Figs. S69 and S70	Figs. S100 and S124
							5.09°	Fig. S41	Figs. S71 and S72	Figs. S101 and S125
							4.41°	Fig. S42	Figs. S73 and S74	Figs. S102 and S126
							3.89°	Fig. S43	Figs. S75 and S76	Figs. S103 and S127
	AB	Table S10	Table S23	Table S24	Table S25	Table S26	9.43°	Fig. S44	Fig. S77	Figs. S104 and S128
							7.34°	Fig. S45	Fig. S78	Figs. S105 and S129
							6.01°	Fig. S46	Fig. S79	Figs. S106 and S130
							5.09°	Fig. S47	Figs. S80 and S81	Figs. S107 and S131
							4.41°	Fig. S48	Figs. S82 and S83	Figs. S108 and S132
							3.89°	Fig. S49	Figs. S84 and S85	Figs. S109 and S133

TABLE S6. Summary of additional results presented in this section. For each monolayer and stacking configuration, we list the tables that detail the corresponding continuum models. Each of the five types of models listed in Section XI A 1 – namely: (1) the full model, (2) the full first moiré harmonic model, (3) the reduced first moiré harmonic model, (4) the full first moiré harmonic model with zero-twist constraints, and (5) the reduced first moiré harmonic model with zero-twist constraints – are summarized in one table. For each commensurate angle, we present the valley-resolved spectra of each continuum model and compare them with the *ab initio* results (referenced in the "Spectrum" column). Additional results are shown for each set of gapped conduction bands of the moiré continuum model in valley $\eta = 0$. Lastly, we compute and plot the Wilson loops for each set of gapped conduction bands along the $\mathbf{b}_{M_1}$ and $\mathbf{b}_{M_2}$ lattice vectors.

Additionally, we use a similar notation for the *ab initio* wave function and projectors, $u^{\text{DFT}}_{\mathbf{Q},s,l;\eta,n}(\mathbf{k})$ and $P^{\text{DFT},m,\eta}_{\mathbf{Q},s,l;\mathbf{Q}',s',l'}(\mathbf{k})$, which are directly obtained from the *ab initio* moiré Hamiltonian defined in Eq. (S8.254).

Finally, we also define the embedding matrix

$$\mathcal{V}_{\mathbf{Q},s,l;\mathbf{Q}',s',l'}(\mathbf{G}) \equiv \delta_{\mathbf{Q}+\mathbf{G},\mathbf{Q}'}\delta_{ss'}\delta_{ll'}, \quad \text{for any} \quad \mathbf{G} \in \mathcal{Q}, \tag{S11.344}$$

such that Eq. (S11.342) becomes equivalent to

$$u_{\mathbf{Q},s,l;\eta,n}(\mathbf{k}+\mathbf{G}) = \sum_{\substack{s',l' \\ \mathbf{Q}' \in \mathcal{Q}_{\text{tot}}}} \mathcal{V}_{\mathbf{Q},s,l;\mathbf{Q}',s',l'}(\mathbf{G})\, u_{\mathbf{Q}',s',l';\eta,n}(\mathbf{k}). \tag{S11.345}$$

2. Overlaps, zero-twist symmetries, SU (2) symmetry

For the continuum models considered here, we assess the goodness of fit by computing the overlap between the fitted and *ab initio* band wave function for the m-th set of bands

$$\mathcal{O}_m = \min_{\substack{0\leq\eta\leq 2\\ \mathbf{k}\in\mathcal{M}}} \sqrt{\frac{\operatorname{Tr}\left(P^{m,\eta}\left(\mathbf{k}\right)P^{\mathrm{DFT},m,\eta}\left(\mathbf{k}\right)\right)}{2}}, \tag{S11.346}$$

where $\mathcal{M}$ are the set of $\mathbf{k}$ point along which the *ab initio* band structure is computed. These overlaps are listed in the tables from Section XI C. Additionally, we compare the energy spectra of the fitted continuum model and the *ab initio* Hamiltonians in Section XI D.

We also evaluate how well the emergent $\tilde{M}_z$ and $\tilde{\mathcal{I}}$ symmetries, which appear in the zero-twist limit of the AA- and AB-stacked moiré Hamiltonians without gradient terms (as detailed in Section VI A), are preserved within the first set of bands of the full model moiré Hamiltonian. If these symmetries were perfectly maintained, the projectors for the first set of bands would satisfy the following conditions

$$P^{\mathrm{AA},1,\eta}_{\mathbf{Q},s,l;\mathbf{Q}',s',l'}\left(\mathbf{k}\right) = P^{\mathrm{AA},1,\eta}_{\mathbf{Q}+\mathbf{q}_\eta,s,-l;\mathbf{Q}'+\mathbf{q}_\eta,s',-l'}\left(\mathbf{k}+\mathbf{q}_\eta\right) \tag{S11.347}$$

$$P^{\mathrm{AB},1,\eta}_{\mathbf{Q},s,l;\mathbf{Q}',s',l'}\left(\mathbf{k}\right) = (-1)^s(-1)^{s'} P^{\mathrm{AB},1,\eta}_{-\mathbf{Q}+\mathbf{q}_\eta,s,-l;-\mathbf{Q}'+\mathbf{q}_\eta,s',-l'}\left(-\mathbf{k}+\mathbf{q}_\eta\right), \tag{S11.348}$$

for the AA- and AB-stacked cases, respectively. These conditions follow directly from Eqs. (S6.138) and (S6.139).

To quantify the degree to which these symmetries are obeyed, we first define the transformed projectors (using the full model continuum Hamiltonian) as

$$P'^{\mathrm{AA},1,\eta}_{\mathbf{Q},s,l;\mathbf{Q}',s',l'}\left(\mathbf{k}\right) \equiv P^{\mathrm{AA},1,\eta}_{\mathbf{Q}+\mathbf{q}_\eta,s,-l;\mathbf{Q}'+\mathbf{q}_\eta,s',-l'}\left(\mathbf{k}+\mathbf{q}_\eta\right) \tag{S11.349}$$

$$P'^{\mathrm{AB},1,\eta}_{\mathbf{Q},s,l;\mathbf{Q}',s',l'}\left(\mathbf{k}\right) \equiv (-1)^s(-1)^{s'} P^{\mathrm{AB},1,\eta}_{-\mathbf{Q}+\mathbf{q}_\eta,s,-l;-\mathbf{Q}'+\mathbf{q}_\eta,s',-l'}\left(-\mathbf{k}+\mathbf{q}_\eta\right), \tag{S11.350}$$

and compute the following overlap metrics

$$\varepsilon_{\tilde{M}_z} \equiv 1 - \sqrt{\frac{1}{N}\sum_{\mathbf{k}\in\mathrm{MBZ}} \frac{\operatorname{Tr}\left(P'^{\mathrm{AA},1,\eta}\left(\mathbf{k}\right)P^{\mathrm{AA},1,\eta}\left(\mathbf{k}\right)\right)}{2}}, \quad \text{for} \quad \eta = 0, \tag{S11.351}$$

$$\varepsilon_{\tilde{\mathcal{I}}} \equiv 1 - \sqrt{\frac{1}{N}\sum_{\mathbf{k}\in\mathrm{MBZ}} \frac{\operatorname{Tr}\left(P'^{\mathrm{AB},1,\eta}\left(\mathbf{k}\right)P^{\mathrm{AB},1,\eta}\left(\mathbf{k}\right)\right)}{2}}, \quad \text{for} \quad \eta = 0, \tag{S11.352}$$

in the AA- and AB-stacked cases, respectively.

Finally, we compute the degree of effective SU (2) symmetry breaking for the first set of bands in a manner similar to that described in Eq. (S2.13). To achieve this, we define the spin-symmetric and spin-antisymmetric components of the band projectors as follows:

$$\begin{aligned} P^{\mathrm{sym},m,\eta}_{\mathbf{Q},s,l;\mathbf{Q}',s',l'}\left(\mathbf{k}\right) &\equiv \frac{\delta_{ss'}}{2}\sum_{s''} P^{m,\eta}_{\mathbf{Q},s'',l;\mathbf{Q}',s'',l'}\left(\mathbf{k}\right), \\ P^{\mathrm{asym},m,\eta}_{\mathbf{Q},s,l;\mathbf{Q}',s',l'}\left(\mathbf{k}\right) &\equiv P^{m,\eta}_{\mathbf{Q},s,l;\mathbf{Q}',s',l'}\left(\mathbf{k}\right) - P^{\mathrm{sym},m,\eta}_{\mathbf{Q},s,l;\mathbf{Q}',s',l'}\left(\mathbf{k}\right). \end{aligned} \tag{S11.353}$$

The effective SU (2) symmetry breaking for the first set of bands is then quantified as

$$\varepsilon_{\mathrm{SU}(2)} \equiv \frac{1}{N}\sum_{\mathbf{k}\in\mathrm{MBZ}} \frac{\left\|P^{\mathrm{asym},1,\eta}\left(\mathbf{k}\right)\right\|}{\left\|P^{1,\eta}\left(\mathbf{k}\right)\right\|}, \quad \text{for} \quad \eta = 0. \tag{S11.354}$$

3. Berry curvature, CDD, and Wilson loops

In Section XI D 2, we present further details on the fitted continuum models. For simplicity, each figure in Section XI D 2 follows the same layout and considers a single set of gapped moiré bands for each of the five continuum models listed in Section XI A 1. For each model, we plot the dispersion of the m-th set of gapped bands across the entire moiré BZ

$$E_{\mathbf{k}} = \frac{\epsilon_{2m-1,\eta}\left(\mathbf{k}\right) + \epsilon_{2m,\eta}\left(\mathbf{k}\right)}{2} - E_0, \quad \text{for} \quad \eta = 0. \tag{S11.355}$$

We average the energies of the two (nearly) spin-degenerate bands and add an offset E_0 such that $\min_{\mathbf{k}\in\text{MBZ}} E_{\mathbf{k}} = 0$. Additionally, we plot the non-abelian Berry curvature [48, 49] for the m-th set of bands

$$\mathcal{F}_{\mathbf{k}} = i\,\text{Tr}\left(P^{m,\eta}(\mathbf{k})\left[\frac{\partial P^{m,\eta}(\mathbf{k})}{\partial k_x}, \frac{\partial P^{m,\eta}(\mathbf{k})}{\partial k_y}\right]\right), \quad \text{for} \quad \eta = 0. \tag{S11.356}$$

We also compute and display the layer-resolved CDD for the m-th set of bands within the model. Specifically, we consider the following many-body state

$$\left|\phi^{\eta,m}\right\rangle = \left(\prod_{\mathbf{k}\in\text{MBZ}} \hat{c}^\dagger_{\mathbf{k},\eta,2m-1}\hat{c}^\dagger_{\mathbf{k},\eta,2m}\right)\left|0\right\rangle, \quad \text{for} \quad m \geq 1, \tag{S11.357}$$

which corresponds to fully filling the m-th set of bands in valley η. The layer-resolved CDD is defined using the continuum real-space operators from Eq. (S4.73) as

$$\begin{aligned}
\rho_{\eta,m,l}(\mathbf{r}) &\equiv \sum_s \left\langle\phi^{\eta,m}\right|\hat{\psi}^\dagger_{\eta,s,l}(\mathbf{r})\,\hat{\psi}_{\eta,s,l}(\mathbf{r})\left|\phi^{\eta,m}\right\rangle \\
&= \frac{1}{N\Omega_0}\sum_s \sum_{\substack{\mathbf{k},\mathbf{k}'\in\text{MBZ}\\ \mathbf{Q},\mathbf{Q}'\in\mathcal{Q}_{\text{tot}}}} \left\langle\phi^{\eta,m}\right|\hat{c}^\dagger_{\mathbf{k},\mathbf{Q},s,l}e^{-i(\mathbf{k}-\mathbf{Q})\cdot\mathbf{r}}\hat{c}_{\mathbf{k}',\mathbf{Q}',s,l}e^{i(\mathbf{k}'-\mathbf{Q}')\cdot\mathbf{r}}\left|\phi^{\eta,m}\right\rangle \\
&= \frac{1}{N\Omega_0}\sum_s \sum_{n=2m-1}^{2m} \sum_{\substack{\mathbf{k}\in\text{MBZ}\\ \mathbf{Q},\mathbf{Q}'\in\mathcal{Q}_{\text{tot}}}} u^*_{\mathbf{Q},s,l;\eta,n}(\mathbf{k})\,u_{\mathbf{Q}',s,l;\eta,n}(\mathbf{k})\,e^{i(\mathbf{Q}-\mathbf{Q}')\cdot\mathbf{r}} \\
&= \frac{1}{N\Omega_0}\sum_s \sum_{n=2m-1}^{2m} \sum_{\substack{\mathbf{k}\in\text{MBZ}\\ \mathbf{Q}\in\mathcal{Q}_{\eta+l}\\ \mathbf{G}\in\mathcal{Q}}} u^*_{\mathbf{Q}+\mathbf{G},s,l;\eta,n}(\mathbf{k})\,u_{\mathbf{Q},s,l;\eta,n}(\mathbf{k})\,e^{i\mathbf{G}\cdot\mathbf{r}}.
\end{aligned} \tag{S11.358}$$

We plot the layer-resolved CDD $\rho_{\eta,m,l}(\mathbf{r})$ in valley $\eta = 0$ for each set of gapped conduction bands.

In Sections XI D 3 and XI D 4, we also plot the Wilson loops for the first one or two sets of gapped moiré conduction bands in valley $\eta = 0$. Two Wilson loops are considered along the reciprocal moiré lattice vectors. Along the $\mathbf{b}_M = \mathbf{b}_{M_1}, \mathbf{b}_{M_2}$ moiré lattice vector, the Wilson loop is defined as

$$\mathcal{W}^m_{\mathbf{b}_M}(\mathbf{k}) = \mathcal{V}(-\mathbf{b}_M)\lim_{N_{\mathcal{W}}\to\infty}\prod_j^{N_{\mathcal{W}}\leftarrow 0} P^{m,\eta}\left(\mathbf{k}+\frac{j}{N_{\mathcal{W}}}\mathbf{b}_M\right), \quad \text{for} \quad \eta = 0 \tag{S11.359}$$

where the ordered product of projectors (in the order indicated by the arrow in $\prod_j^{N_{\mathcal{W}}}$) is given by

$$\prod_j^{N_{\mathcal{W}}\leftarrow 0} P^{m,\eta}\left(\mathbf{k}+\frac{j}{N_{\mathcal{W}}}\mathbf{b}_M\right) \equiv P^{m,\eta}(\mathbf{k}+\mathbf{b}_M)\ldots P^{m,\eta}\left(\mathbf{k}+\frac{2}{N_{\mathcal{W}}}\mathbf{b}_M\right)P^{m,\eta}\left(\mathbf{k}+\frac{1}{N_{\mathcal{W}}}\mathbf{b}_M\right)P^{m,\eta}(\mathbf{k}). \tag{S11.360}$$

The nonzero eigenvalues of the Wilson matrix are phases denoted by $e^{i\theta_W}$. In Section XI D 3 (Section XI D 4), we plot the phase of the Wilson loop $\mathcal{W}^m_{\mathbf{b}_{M_1}}(k_2\mathbf{b}_{M_2})$ [$\mathcal{W}^m_{\mathbf{b}_{M_2}}(k_1\mathbf{b}_{M_1})$] as a function of $0 \leq k_2 \leq 1$ ($0 \leq k_1 \leq 1$).

As shown in Ref. [50], the phases $e^{i\theta_W}$ of the $\mathcal{W}^m_{\mathbf{b}_{M_1}}(k_2\mathbf{b}_{M_2})$ Wilson loop correspond to the eigenvalues of the projected position operator along the $\mathbf{a}_{M_1}$ basis vector. Specifically, the $n = 2m-1$ and $n = 2m$ bands, with wave functions $u_{\mathbf{Q},s,l;\eta,n}(k\mathbf{b}_{M_1} + k_2\mathbf{b}_{M_2})$, can be Wannierized along the $\mathbf{a}_{M_1}$ direction, yielding hybrid Wannier orbitals for every value of $0 \leq k_2 \leq 1$. The corresponding Wannier centers are located at $\frac{\theta_W}{2\pi}\mathbf{a}_{M_1}$ for each eigenvalue $e^{i\theta_W}$ of the $\mathcal{W}^m_{\mathbf{b}_{M_1}}(k_2\mathbf{b}_{M_2})$ Wilson loop. The eigenvalues of the $\mathcal{W}^m_{\mathbf{b}_{M_2}}(k_1\mathbf{b}_{M_1})$ Wilson loop can be interpreted in a similar manner.

B. Discussion

Our comprehensive analysis of the *ab initio* simulations reveals a series of general features, summarized below. In particular, we discuss the relevance and applicability of all the models outlined in Table S3.

- Both the AA- and AB-stacked moiré Hamiltonians exhibit excellent emergent SU (2) symmetry for the lowest gapped group of bands. This arises primarily from the low-energy *effective* SU (2) symmetry of the monolayer materials. As discussed in Section II, any single-spinful-band model describing the bottom of the conduction band in either $SnSe_2$ or ZrS_2 possesses perfect emergent SU (2) symmetry, enforced by $\mathcal{IT}$. In the twisted heterostructure, the moiré potential induces only weak SU (2) symmetry breaking, resulting in an emergent SU (2) symmetry for the lowest gapped group of bands. The superior SU (2) symmetry observed in the AA-stacked case, as shown in Tables S7 and S9, is attributed to the zero-twist symmetries. In the first moiré harmonic model for the AA-stacked case, introduced in Eq. (S5.94), the zero-twist limit *implies* SU (2) symmetry. However, for AB-stacking, the first moiré harmonic model in Eq. (S5.95) does not necessarily feature SU (2) symmetry in the zero-twist limit.

- For all the twist angles considered in this work, the system exhibits at least one gapped set of bands, comprising six bands from the two spin flavors and three valleys. Consequently, the system *cannot* be described by the first monolayer harmonic model discussed in Section IV A and summarized in Table S3, as such a model assumes continuous translational symmetry along the $C_{3z}^{\eta}\hat{\mathbf{y}}$ direction in valley η and results in an overall *gapless* spectrum. This behavior contrasts with TBG, where the first monolayer harmonic model provides an accurate description.

- The lowest gapped set of bands exhibits good zero-twist symmetries. The presence of these symmetries depends on the extent to which the local-stacking approximation holds, as demonstrated in Section VI A. As discussed in Section III B, the in-plane relaxation displacements in twisted ZrS_2 are approximately half as large as those in twisted $SnSe_2$. Consequently, the local-stacking approximation is expected to hold better for ZrS_2. This is reflected in Tables S7 to S10, which show that, at low twist angles, the emerging $\tilde{M}_z$ and $\tilde{\mathcal{I}}$ symmetries are better preserved in ZrS_2 compared to $SnSe_2$.

- The low-energy spectrum of all the moiré heterostructures considered in this work is excellently described by both the full first moiré harmonic model and the reduced first moiré harmonic model, in terms of both dispersion and wave functions. Furthermore, due to the excellent SU (2) symmetry, these models closely approach the SU (2) limit summarized in Table S3.

- The first moiré harmonic model with zero-twist constraints imposed, along with its reduced version, accurately describes all the moiré heterostructures in terms of the wave functions for the lowest gapped set of bands. Among the four possible combinations of stacking configurations and monolayers, the overlap between the *ab initio* wave function and the wave function computed from the continuum model is smallest for AB-stacked $SnSe_2$, due to strong relaxation effects.

  When considering dispersion, the first moiré harmonic model with zero-twist constraints imposed accurately describes the first two bands of AA-stacked twisted $SnSe_2$. However, it does not correctly capture the dispersion of AB-stacked $SnSe_2$ for small twist angles $\theta \leq 5.09°$. On the other hand, the first moiré harmonic model with zero-twist constraints does accurately describe the dispersion of twisted ZrS_2.

- When the monolayer masses are treated as variational parameters, their fitted values agree with the values extracted from the monolayer *ab initio* spectrum to within 10%.

- At small twist angles (*i.e.* $\theta = 3.89°$), all four possible monolayer and stacking configuration combinations are well-described by the respective three-parameter models introduced in Section VI E and summarized in Table S3. Specifically:

  - For the AB-stacked case, Tables S18 and S26 show that $\left|w_3'^{\text{AB}}\right| < \left|w_2^{\text{AB}}\right|, \left|w_4'^{\text{AB}}\right|$, indicating that the corresponding three-parameter model is close to (and adiabatically connected to) the two-parameter limit described at the end of Section VI E 2.
  - For twisted AA-stacked $SnSe_2$, the three-parameter model accurately reproduces both the dispersion *and* the wave function of the first two conduction bands. Since w_1^{AA} is comparable to w_2^{AA}, the model does not lie in the C_{2z} limit but instead features $\tilde{C}_{2z}$ symmetry. The rotation center of $\tilde{C}_{2z}$ does not correspond to the origin of the moiré unit cell but is displaced, as explained in Section VI E 1. The positions of the Wannier orbitals for both the first and second gapped conduction bands can be inferred from Eq. (S6.194). According to *ab initio* results in Table S14, arg $\left(iw_1^{\text{AA}} + w_2^{\text{AB}}\right) \approx 0.2\pi \approx \frac{\pi}{3}$ and $w_3'^{\text{AA}} < 0$, implying from Eq. (S6.194) that the Wannier orbitals in valley $\eta = 0$ are located approximately at $\frac{2}{3}\mathbf{a}_{M_1} + \frac{1}{3}\mathbf{a}_{M_2}$, *i.e.* one of the honeycomb sites, as shown in Figs. S57 and S58.
  - For twisted AB-stacked $SnSe_2$, the three-parameter model reproduces the *ab initio* wave function well but only qualitatively matches the dispersion. The positions of the Wannier orbitals for the first two

gapped conduction bands are qualitatively described by Eq. (S6.196), predicting the Wannier centers to be approximately located at $\frac{1}{2}\mathbf{a}_{M_2}$ in valley $\eta = 0$, *i.e.* one of the three kagome sites, as shown in Figs. S65 and S66.

– For twisted AA-stacked ZrS_2, the three-parameter model accurately reproduces both the dispersion *and* the wave function of the first two conduction bands. Since $\left|w_1^{\mathrm{AA}}\right| \ll \left|w_2^{\mathrm{AA}}\right|$, the model is close to the C_{2z} limit. The positions of the Wannier orbitals for both the first and second gapped conduction bands can be inferred from Eq. (S6.194). According to *ab initio* results in Table S14, $\arg\left(iw_1^{\mathrm{AA}} + w_2^{\mathrm{AB}}\right) \approx 0$ and $w_3'^{\mathrm{AA}} < 0$, implying from Eq. (S6.194) that the Wannier orbitals in valley $\eta = 0$ are located approximately at $\frac{1}{2}\mathbf{a}_{M_2}$, *i.e.* one of the kagome sites, as shown in Figs. S75 and S76.

– For twisted AB-stacked ZrS_2, the three-parameter model accurately reproduces both the dispersion *and* the wave function of the first two conduction bands. The positions of the Wannier orbitals for the first two gapped conduction bands are qualitatively described by Eq. (S6.196), predicting the Wannier centers to be approximately located at the origin, as shown in Figs. S84 and S85.

– The Wannier centers of both the first and second sets of conduction bands are approximately located at the same position. In valley $\eta = 0$, the Wannier orbitals of the first set of conduction bands resemble s-orbitals (elongated along the $\hat{\mathbf{x}}$ direction for ZrS_2), while the Wannier orbitals of the second set of bands resemble distorted p_y-orbitals.

- For AA-stacking, the three-parameter model accurately describes the wave functions of the first and second sets of conduction bands. Since the non-Abelian Berry curvature vanishes in the three-parameter model, as discussed in Section VI E 1, this indicates that the AA-stacked gapped conduction bands are topologically trivial and exhibit very small Berry curvature.

- For AB-stacking, the three-parameter model also accurately describes the wave functions of the first and second sets of conduction bands. Furthermore, the three-parameter AB-stacked model is adiabatically connected to the two-parameter AB-stacked model, as discussed in Section VI E 2. The two-parameter model features vanishing Berry curvature, implying that the corresponding gapped bands are also topologically trivial and exhibit very small Berry curvature.

- The small Berry curvature of the system results in nearly perfectly flat Wilson-loop spectra. Additionally, the Wilson bands reflect the positions of the corresponding Wannier orbitals, as discussed at the end of Section XI A 3.

C. Parameter values

1. *Full models*

θ	Nonzero parameters	Total parameters	ϵ_c/meV	σ/meV	$\mathcal{O}_1$	$\mathcal{O}_2$	$\mathcal{O}_3$	$\mathcal{O}_4$	$\mathcal{O}_5$	$\varepsilon_{\mathrm{SU}(2)}(\%)$	$\varepsilon_{\tilde{M}_z}(\%)$
9.43°	697	2016	500	100	0.9993	0.9821	0.9816	0.9917	0.9778	1.25	1.33
7.34°	298	2016	450	100	0.9992	0.9922	0.9831	0.9832	0.9919	1.02	1.81
6.01°	285	2016	375	75	0.9990	0.9963	0.9898	0.9898	0.9856	0.52	3.20
5.09°	143	3570	300	75	0.9998	0.9994	0.9876	0.9875	0.9909	0.12	4.82
4.41°	193	3570	300	75	0.9997	0.9996	0.9970	0.9957	0.9834	0.67	7.02
3.89°	182	3570	300	75	0.9998	0.9996	0.9962	0.9972	0.9940	0.60	8.94

TABLE S7. Details of the full continuum model for twisted AA-stacked bilayer $SnSe_2$. The total and nonzero parameters used in the fitting procedure are listed, along with the energy weighting function parameters. Also shown are the minimal overlaps for the first five groups of bands and the percentages of SU(2) and $\tilde{M}_z$ symmetry breaking in the first group of bands.

θ	Nonzero parameters	Total parameters	ϵ_c/meV	σ/meV	$\mathcal{O}_1$	$\mathcal{O}_2$	$\mathcal{O}_3$	$\mathcal{O}_4$	$\mathcal{O}_5$	$\varepsilon_{\mathrm{SU}(2)}(\%)$	$\varepsilon_{\tilde{\mathcal{I}}_z}(\%)$
9.43°	358	2010	500	100	0.9993	0.9671	0.8988	0.9017	0.9509	3.02	0.59
7.34°	293	2010	450	100	0.9995	0.9908	0.9897	0.9897	0.9760	3.36	1.58
6.01°	632	2592	375	75	0.9996	0.9605	0.9603	0.9966	0.9964	3.67	3.48
5.09°	171	3558	300	75	0.9996	0.9926	0.9922	0.9901	0.9899	3.82	5.16
4.41°	250	3558	300	75	0.9997	0.9966	0.9832	0.9843	0.9715	3.89	7.29
3.89°	309	3558	300	75	0.9998	0.9982	0.9901	0.9901	0.9827	4.47	8.85

TABLE S8. Details of the full continuum model for twisted AB-stacked bilayer $SnSe_2$. The total and nonzero parameters used in the fitting procedure are listed, along with the energy weighting function parameters. Also shown are the minimal overlaps for the first five groups of bands and the percentages of SU(2) and $\tilde{\mathcal{I}}_z$ symmetry breaking in the first group of bands.

θ	Nonzero parameters	Total parameters	ϵ_c/meV	σ/meV	$\mathcal{O}_1$	$\mathcal{O}_2$	$\mathcal{O}_3$	$\mathcal{O}_4$	$\mathcal{O}_5$	$\varepsilon_{\mathrm{SU}(2)}(\%)$	$\varepsilon_{\tilde{M}_z}(\%)$
9.43°	141	1044	375	75	0.9994	0.9592	0.9592	0.9871	0.9833	0.05	3.08
7.34°	117	2016	338	75	0.9996	0.9904	0.9584	0.9583	0.9797	0.00	2.68
6.01°	197	2016	281	56	0.9996	0.9858	0.9806	0.9889	0.9863	0.12	2.67
5.09°	56	3570	225	56	0.9995	0.9983	0.9221	0.9609	0.9221	0.00	2.02
4.41°	79	3570	225	56	0.9993	0.9994	0.9857	0.9822	0.9842	0.00	1.57
3.89°	69	3570	225	56	0.9998	0.9997	0.9991	0.9988	0.9968	0.00	1.23

TABLE S9. Details of the full continuum model for twisted AA-stacked bilayer ZrS_2. The total and nonzero parameters used in the fitting procedure are listed, along with the energy weighting function parameters. Also shown are the minimal overlaps for the first five groups of bands and the percentages of SU(2) and $\tilde{M}_z$ symmetry breaking in the first group of bands.

θ	Nonzero parameters	Total parameters	ϵ_c/meV	σ/meV	$\mathcal{O}_1$	$\mathcal{O}_2$	$\mathcal{O}_3$	$\mathcal{O}_4$	$\mathcal{O}_5$	$\varepsilon_{\mathrm{SU}(2)}(\%)$	$\varepsilon_{\tilde{\mathcal{I}}_z}(\%)$
9.43°	169	1038	375	75	0.9993	0.9979	0.9786	0.9785	0.9971	2.21	3.49
7.34°	157	2010	338	75	0.9995	0.9884	0.9769	0.9769	0.9824	2.11	3.71
6.01°	171	2010	281	56	0.9991	0.9948	0.9935	0.9933	0.9953	2.17	3.35
5.09°	61	3558	225	56	0.9996	0.9971	0.9902	0.9902	0.9921	2.27	3.11
4.41°	73	3558	225	56	0.9994	0.9974	0.9938	0.9940	0.9954	2.24	3.08
3.89°	63	3558	225	56	0.9996	0.9992	0.9941	0.9939	0.9946	2.22	2.78

TABLE S10. Details of the full continuum model for twisted AB-stacked bilayer ZrS_2. The total and nonzero parameters used in the fitting procedure are listed, along with the energy weighting function parameters. Also shown are the minimal overlaps for the first five groups of bands and the percentages of SU(2) and $\tilde{\mathcal{I}}_z$ symmetry breaking in the first group of bands.

2. *First moiré harmonic models*

θ	m_x	m_y	w_1^{AA}	w_2^{AA}	w_3^{AA}	w_4^{AA}	w_5^{AA}	w_6^{AA}	$w_1'^{\mathrm{AA}}$	$w_2'^{\mathrm{AA}}$	$w_3'^{\mathrm{AA}}$	$w_4'^{\mathrm{AA}}$	$w_5'^{\mathrm{AA}}$	$w_6'^{\mathrm{AA}}$	$w_7'^{\mathrm{AA}}$	$w_8'^{\mathrm{AA}}$	$w_9'^{\mathrm{AA}}$	$w_{10}'^{\mathrm{AA}}$	$\mathcal{O}_1$	$\mathcal{O}_2$
9.43°	0.22	0.67	69.21	69.30	−0.93	1.31	−1.79	−2.44	−5.75	14.50	−10.89	−4.19	28.17	0.64	20.72	−0.73	0.09	14.65	0.9933	0.1505
7.34°	0.22	0.66	56.40	70.11	−0.69	−0.27	−1.39	−0.51	−7.18	21.62	−7.49	1.88	33.60	0.23	23.88	−0.41	−0.12	11.79	0.9964	0.9185
6.01°	0.25	0.75	55.57	71.10	−0.55	−0.42	−0.88	−0.23	−5.21	11.11	−6.73	3.49	26.81	−0.40	13.98	0.21	0.59	10.17	0.9956	0.9825
5.09°	0.21	0.64	63.13	80.70	−0.43	−0.87	−0.70	0.14	−3.86	13.41	−11.58	2.53	27.13	−0.27	19.80	0.12	0.35	18.32	0.9976	0.9891
4.41°	0.23	0.67	57.21	89.38	−0.52	−1.84	−0.66	1.15	−2.78	7.64	−15.79	1.90	33.60	−0.83	25.51	0.58	−0.37	20.36	0.9982	0.9913
3.89°	0.24	0.63	65.29	98.14	−0.58	−2.29	−0.56	1.54	−0.28	−2.08	−18.70	4.69	33.32	−1.01	29.64	0.42	−0.47	28.22	0.9986	0.9944

TABLE S11. Parameter values of the full first moiré harmonic model for twisted AA-stacked bilayer $SnSe_2$. The interlayer hopping parameters are given in units of meV, while the effective masses are given in units of the bare electron mass m_e. The minimal overlaps with the first two groups of bands are listed at the end.

θ	m_x	m_y	w_1^{AA}	w_2^{AA}	$w_1'^{\mathrm{AA}}$	$w_3'^{\mathrm{AA}}$	$w_7'^{\mathrm{AA}}$	$w_{10}'^{\mathrm{AA}}$	$\mathcal{O}_1$	$\mathcal{O}_2$
9.43°	0.20	0.69	84.89	72.39	−14.08	N/A	19.86	16.33	0.9891	0.1527
7.34°	0.20	0.66	88.78	78.32	N/A	−16.21	24.61	18.47	0.9945	0.8733
6.01°	0.27	0.75	71.97	63.54	N/A	−15.43	11.87	13.92	0.9928	0.9645
5.09°	0.25	0.64	79.24	69.33	N/A	−18.53	16.82	23.18	0.9958	0.9860
4.41°	0.28	0.67	74.11	66.00	N/A	−21.13	18.32	26.02	0.9965	0.9912
3.89°	0.27	0.63	77.12	69.40	N/A	−23.98	19.69	34.35	0.9973	0.9928

TABLE S12. Parameter values of the reduced first moiré harmonic model for twisted AA-stacked bilayer $SnSe_2$. The interlayer hopping parameters are given in units of meV, while the effective masses are given in units of the bare electron mass m_e. The minimal overlaps with the first two groups of bands are listed at the end.

θ	m_x	m_y	w_1^{AA}	w_2^{AA}	$w_1'^{\mathrm{AA}}$	$w_2'^{\mathrm{AA}}$	$w_3'^{\mathrm{AA}}$	$w_4'^{\mathrm{AA}}$	$w_5'^{\mathrm{AA}}$	$\mathcal{O}_1$	$\mathcal{O}_2$
9.43°	0.21	0.73	66.22	65.91	−6.95	44.29	−10.74	−11.33	34.62	0.9688	0.0267
7.34°	0.21	0.73	63.73	73.71	−0.55	31.16	−7.92	3.69	30.77	0.9834	0.3233
6.01°	0.21	0.73	65.09	77.81	−1.94	17.10	−9.61	4.56	28.55	0.9835	0.8716
5.09°	0.21	0.73	65.58	81.52	−5.15	14.95	−9.62	2.92	24.88	0.9805	0.9079
4.41°	0.21	0.73	66.85	85.67	−2.94	14.28	−11.06	4.74	26.71	0.9767	0.9226
3.89°	0.21	0.73	66.38	88.80	−4.50	11.04	−7.99	5.71	27.25	0.9722	0.9280

TABLE S13. Parameter values of the first moiré harmonic model with the zero-twist constraints imposed for twisted AA-stacked bilayer $SnSe_2$. The interlayer hopping parameters are given in units of meV, while the effective masses are given in units of the bare electron mass m_e. The minimal overlaps with the first two groups of bands are listed at the end.

θ	m_x	m_y	w_1^{AA}	w_2^{AA}	$w_1'^{\mathrm{AA}}$	$w_3'^{\mathrm{AA}}$	$\mathcal{O}_1$	$\mathcal{O}_2$
9.43°	0.21	0.73	66.22	65.91	−12.22	N/A	0.9762	0.1121
7.34°	0.21	0.73	63.73	73.71	−14.35	N/A	0.9818	0.2888
6.01°	0.21	0.73	65.09	77.81	N/A	−15.34	0.9719	0.8747
5.09°	0.21	0.73	65.58	81.52	N/A	−18.03	0.9667	0.9033
4.41°	0.21	0.73	66.85	85.67	N/A	−18.94	0.9603	0.9101
3.89°	0.21	0.73	66.38	88.80	N/A	−18.94	0.9526	0.9058

TABLE S14. Parameter values of the reduced first moiré harmonic model with the zero-twist constraints imposed for twisted AA-stacked bilayer $SnSe_2$. The interlayer hopping parameters are given in units of meV, while the effective masses are given in units of the bare electron mass m_e. The minimal overlaps with the first two groups of bands are listed at the end.

θ	m_x	m_y	w_1^{AB}	w_2^{AB}	w_3^{AB}	w_4^{AB}	$w_1'^{\mathrm{AB}}$	$w_2'^{\mathrm{AB}}$	$w_3'^{\mathrm{AB}}$	$w_4'^{\mathrm{AB}}$	$w_5'^{\mathrm{AB}}$	$w_6'^{\mathrm{AB}}$	$w_7'^{\mathrm{AB}}$	$w_8'^{\mathrm{AB}}$	$w_9'^{\mathrm{AB}}$	$w_{10}'^{\mathrm{AB}}$	$w_{11}'^{\mathrm{AB}}$	$w_{12}'^{\mathrm{AB}}$	$\mathcal{O}_1$	$\mathcal{O}_2$
9.43°	0.21	0.70	−3.53	−92.77	−1.14	−1.01	−0.32	7.30	−10.85	22.00	0.55	−3.73	−16.22	−0.78	−1.30	1.88	0.28	−8.51	0.9951	0.1228
7.34°	0.21	0.67	−5.32	−81.96	−0.63	−0.52	0.88	7.84	−14.37	30.99	−0.52	−4.01	−29.54	−0.51	−4.36	4.23	0.41	−19.91	0.9981	0.9476
6.01°	0.26	0.77	−4.43	−78.43	−0.50	−0.21	−0.67	9.24	−14.94	35.88	0.37	−7.71	−10.59	−0.36	−4.01	3.94	−0.34	−20.41	0.9984	0.9737
5.09°	0.25	0.69	−3.74	−76.24	−0.38	−0.13	0.04	10.47	−16.87	39.31	−0.06	−7.32	−14.66	−0.82	−6.04	5.22	−0.53	−25.37	0.9990	0.9857
4.41°	0.24	0.77	−3.92	−78.61	−0.09	−0.14	−0.21	12.13	−18.58	46.65	−1.01	−10.52	−17.25	−0.90	−8.31	6.05	−0.95	−33.02	0.9986	0.9918
3.89°	0.23	0.72	−3.65	−84.94	−0.23	−0.01	1.37	14.01	−19.46	57.03	−1.78	−15.89	−20.29	−0.87	−12.01	7.77	−1.27	−40.84	0.9986	0.9933

TABLE S15. Parameter values of the full first moiré harmonic model for twisted AB-stacked bilayer $SnSe_2$. The interlayer hopping parameters are given in units of meV, while the effective masses are given in units of the bare electron mass m_e. The minimal overlaps with the first two groups of bands are listed at the end.

θ	m_x	m_y	w_2^{AB}	$w_2'^{\mathrm{AB}}$	$w_4'^{\mathrm{AB}}$	$w_7'^{\mathrm{AB}}$	$w_9'^{\mathrm{AB}}$	$w_{10}'^{\mathrm{AB}}$	$w_{12}'^{\mathrm{AB}}$	$\mathcal{O}_1$	$\mathcal{O}_2$
9.43°	0.21	0.71	−99.12	7.01	17.85	N/A	N/A	0.95	−8.09	0.9934	0.0347
7.34°	0.21	0.67	−79.41	10.23	26.93	−30.92	N/A	N/A	−21.74	0.9973	0.2354
6.01°	0.26	0.79	−78.22	N/A	25.70	N/A	−9.53	8.85	−18.37	0.9873	0.9576
5.09°	0.25	0.69	−86.65	N/A	29.20	N/A	−11.63	10.71	−24.76	0.9908	0.9687
4.41°	0.25	0.77	−90.78	N/A	31.02	N/A	−14.82	12.95	−31.33	0.9918	0.9820
3.89°	0.24	0.72	−62.10	N/A	31.80	−20.94	N/A	4.11	−35.03	0.9926	0.9844

TABLE S16. Parameter values of the reduced first moiré harmonic model for twisted AB-stacked bilayer $SnSe_2$. The interlayer hopping parameters are given in units of meV, while the effective masses are given in units of the bare electron mass m_e. The minimal overlaps with the first two groups of bands are listed at the end.

θ	m_x	m_y	w_1^{AB}	w_2^{AB}	$w_1'^{\mathrm{AB}}$	$w_2'^{\mathrm{AB}}$	$w_3'^{\mathrm{AB}}$	$w_4'^{\mathrm{AB}}$	$w_5'^{\mathrm{AB}}$	$w_6'^{\mathrm{AB}}$	$w_7'^{\mathrm{AB}}$	$\mathcal{O}_1$	$\mathcal{O}_2$
9.43°	0.21	0.73	−3.34	−81.08	−0.23	5.64	−11.77	18.66	0.59	−2.58	−46.32	0.9873	0.1235
7.34°	0.21	0.73	−5.07	−76.32	0.91	11.93	−16.68	31.30	−0.36	−11.43	−53.45	0.9816	0.4100
6.01°	0.21	0.73	−5.10	−82.56	0.91	8.22	−21.15	44.07	0.59	−13.71	−5.30	0.9791	0.8462
5.09°	0.21	0.73	−4.74	−77.11	−0.51	8.14	−21.77	26.14	0.17	0.76	−12.81	0.9780	0.7112
4.41°	0.21	0.73	−5.27	−82.84	0.09	8.39	−23.13	33.39	−0.92	−3.57	−3.48	0.9725	0.8738
3.89°	0.21	0.73	−4.18	−77.80	1.45	9.45	−24.71	35.10	−2.15	−5.85	−10.28	0.9689	0.8643

TABLE S17. Parameter values of the first moiré harmonic model with the zero-twist constraints imposed for twisted AB-stacked bilayer $SnSe_2$. The interlayer hopping parameters are given in units of meV, while the effective masses are given in units of the bare electron mass m_e. The minimal overlaps with the first two groups of bands are listed at the end.

θ	m_x	m_y	w_2^{AB}	$w_2'^{\mathrm{AB}}$	$w_3'^{\mathrm{AB}}$	$w_4'^{\mathrm{AB}}$	$\mathcal{O}_1$	$\mathcal{O}_2$
9.43°	0.21	0.73	−81.08	5.16	N/A	16.17	0.9847	0.3528
7.34°	0.21	0.73	−76.32	9.30	N/A	17.62	0.9868	0.7436
6.01°	0.21	0.73	−82.56	6.86	N/A	26.48	0.9793	0.7251
5.09°	0.21	0.73	−77.11	6.75	N/A	27.89	0.9725	0.8429
4.41°	0.21	0.73	−82.84	N/A	−23.30	29.10	0.9616	0.8734
3.89°	0.21	0.73	−77.80	N/A	−23.05	27.32	0.9571	0.8644

TABLE S18. Parameter values of the reduced first moiré harmonic model with the zero-twist constraints imposed for twisted AB-stacked bilayer $SnSe_2$. The interlayer hopping parameters are given in units of meV, while the effective masses are given in units of the bare electron mass m_e. The minimal overlaps with the first two groups of bands are listed at the end.

θ	m_x	m_y	w_1^{AA}	w_2^{AA}	w_3^{AA}	w_4^{AA}	w_5^{AA}	w_6^{AA}	$w_1'^{\mathrm{AA}}$	$w_2'^{\mathrm{AA}}$	$w_3'^{\mathrm{AA}}$	$w_4'^{\mathrm{AA}}$	$w_5'^{\mathrm{AA}}$	$w_6'^{\mathrm{AA}}$	$w_7'^{\mathrm{AA}}$	$w_8'^{\mathrm{AA}}$	$w_9'^{\mathrm{AA}}$	$w_{10}'^{\mathrm{AA}}$	$\mathcal{O}_1$	$\mathcal{O}_2$
9.43°	0.27	1.71	−9.99	59.31	0.20	−0.99	0.59	0.16	4.16	−0.01	−28.51	−7.62	−8.33	−0.02	−14.09	0.06	−0.12	10.02	0.9918	0.7264
7.34°	0.26	1.54	−8.58	58.70	0.31	−1.28	0.58	0.57	2.11	−7.45	−30.82	−5.22	−1.75	−0.10	−12.86	−0.06	0.03	6.48	0.9964	0.4586
6.01°	0.27	2.18	−1.11	57.43	0.17	−0.83	0.42	0.28	2.53	−18.40	−25.16	−5.48	3.53	−0.08	−1.82	−0.05	0.01	2.57	0.9910	0.9610
5.09°	0.29	1.93	−4.55	53.26	0.18	−0.89	0.43	0.36	0.54	−13.73	−27.01	−5.61	3.96	−0.10	−0.60	−0.08	0.05	2.06	0.9932	0.9746
4.41°	0.30	1.99	−3.37	47.05	0.09	−0.55	0.33	0.10	1.60	−12.53	−23.00	−3.00	4.59	−0.06	0.24	−0.04	0.04	1.29	0.9942	0.9792
3.89°	0.31	1.88	−5.08	44.80	0.08	−0.52	0.30	0.12	0.99	−10.78	−21.47	−1.56	4.98	−0.05	0.64	−0.03	0.04	0.64	0.9953	0.9821

TABLE S19. Parameter values of the full first moiré harmonic model for twisted AA-stacked bilayer ZrS_2. The interlayer hopping parameters are given in units of meV, while the effective masses are given in units of the bare electron mass m_e. The minimal overlaps with the first two groups of bands are listed at the end.

θ	m_x	m_y	w_1^{AA}	w_2^{AA}	$w_1'^{\mathrm{AA}}$	$w_2'^{\mathrm{AA}}$	$w_3'^{\mathrm{AA}}$	$w_4'^{\mathrm{AA}}$	$w_7'^{\mathrm{AA}}$	$w_{10}'^{\mathrm{AA}}$	$\mathcal{O}_1$	$\mathcal{O}_2$
9.43°	0.27	1.68	−13.23	54.74	N/A	N/A	−23.56	N/A	−13.88	12.41	0.9920	0.5733
7.34°	0.25	1.52	−12.82	56.38	N/A	N/A	−26.49	N/A	−12.76	8.09	0.9967	0.4636
6.01°	0.29	2.21	N/A	46.76	1.97	−19.86	−18.86	N/A	N/A	2.43	0.9880	0.9634
5.09°	0.31	1.93	−4.51	42.32	N/A	−13.45	−20.04	N/A	N/A	1.96	0.9922	0.9750
4.41°	0.37	2.03	−2.54	32.78	1.43	−11.12	−19.24	N/A	N/A	N/A	0.9940	0.9787
3.89°	0.33	1.89	−5.57	37.46	N/A	−10.63	−23.86	−2.90	N/A	N/A	0.9956	0.9816

TABLE S20. Parameter values of the reduced first moiré harmonic model for twisted AA-stacked bilayer ZrS_2. The interlayer hopping parameters are given in units of meV, while the effective masses are given in units of the bare electron mass m_e. The minimal overlaps with the first two groups of bands are listed at the end.

θ	m_x	m_y	w_1^{AA}	w_2^{AA}	$w_1'^{\mathrm{AA}}$	$w_2'^{\mathrm{AA}}$	$w_3'^{\mathrm{AA}}$	$w_4'^{\mathrm{AA}}$	$w_5'^{\mathrm{AA}}$	$\mathcal{O}_1$	$\mathcal{O}_2$
9.43°	0.29	1.86	−3.53	52.58	8.38	0.41	−21.25	−4.59	20.03	0.9603	0.3280
7.34°	0.29	1.86	−6.57	52.53	−0.44	−13.58	−23.08	−2.49	−5.92	0.9797	0.7335
6.01°	0.29	1.86	−10.15	50.51	−0.31	−3.44	−22.68	−3.22	−0.73	0.9866	0.5615
5.09°	0.29	1.86	−8.91	51.29	−0.60	−8.21	−24.06	−2.56	3.63	0.9893	0.8360
4.41°	0.29	1.86	−12.00	50.10	−0.36	−4.32	−24.36	−3.63	2.20	0.9930	0.9341
3.89°	0.29	1.86	−12.35	50.50	−0.59	−5.11	−23.99	−2.78	4.28	0.9948	0.9679

TABLE S21. Parameter values of the first moiré harmonic model with the zero-twist constraints imposed for twisted AA-stacked bilayer ZrS_2. The interlayer hopping parameters are given in units of meV, while the effective masses are given in units of the bare electron mass m_e. The minimal overlaps with the first two groups of bands are listed at the end.

θ	m_x	m_y	w_1^{AA}	w_2^{AA}	$w_3'^{\mathrm{AA}}$	$\mathcal{O}_1$	$\mathcal{O}_2$
9.43°	0.29	1.86	−3.53	52.58	−20.13	0.9612	0.0374
7.34°	0.29	1.86	−6.57	52.53	−20.73	0.9754	0.7307
6.01°	0.29	1.86	−10.15	50.51	−18.86	0.9864	0.5679
5.09°	0.29	1.86	−8.91	51.29	−20.61	0.9856	0.8371
4.41°	0.29	1.86	−12.00	50.10	−19.38	0.9907	0.9340
3.89°	0.29	1.86	−12.35	50.50	−19.83	0.9913	0.9659

TABLE S22. Parameter values of the reduced first moiré harmonic model with the zero-twist constraints imposed for twisted AA-stacked bilayer ZrS_2. The interlayer hopping parameters are given in units of meV, while the effective masses are given in units of the bare electron mass m_e. The minimal overlaps with the first two groups of bands are listed at the end.

θ	m_x	m_y	w_1^{AB}	w_2^{AB}	w_3^{AB}	w_4^{AB}	$w_1'^{\mathrm{AB}}$	$w_2'^{\mathrm{AB}}$	$w_3'^{\mathrm{AB}}$	$w_4'^{\mathrm{AB}}$	$w_5'^{\mathrm{AB}}$	$w_6'^{\mathrm{AB}}$	$w_7'^{\mathrm{AB}}$	$w_8'^{\mathrm{AB}}$	$w_9'^{\mathrm{AB}}$	$w_{10}'^{\mathrm{AB}}$	$w_{11}'^{\mathrm{AB}}$	$w_{12}'^{\mathrm{AB}}$	$\mathcal{O}_1$	$\mathcal{O}_2$
9.43°	0.27	1.79	−2.06	−53.12	0.72	−0.37	−0.76	−4.99	−19.64	−23.74	0.14	6.57	0.73	−0.20	−8.79	1.05	0.25	−3.23	0.9984	0.9832
7.34°	0.26	1.63	−2.13	−50.84	0.62	−0.28	−0.87	−4.13	−14.24	−26.51	0.20	5.52	2.19	−0.15	−7.79	0.51	0.22	−1.29	0.9989	0.8526
6.01°	0.26	2.27	−2.23	−47.30	0.52	−0.24	−0.74	−2.90	−12.45	−23.06	0.55	5.16	1.07	−0.19	−6.42	1.26	0.18	−0.87	0.9994	0.9937
5.09°	0.24	1.93	−2.09	−47.60	0.50	−0.18	−0.88	−2.90	−11.75	−25.31	0.59	6.22	0.23	−0.16	−6.65	1.73	0.23	−0.22	0.9993	0.9983
4.41°	0.25	2.05	−2.06	−44.76	0.40	−0.15	−0.78	−2.55	−10.58	−24.51	0.48	5.72	0.64	−0.11	−5.89	1.38	0.20	0.20	0.9996	0.9986
3.89°	0.26	1.91	−1.74	−38.37	0.38	−0.10	−0.73	−2.27	−9.09	−22.99	0.46	4.20	0.05	−0.08	−5.29	1.10	0.19	0.95	0.9998	0.9989

TABLE S23. Parameter values of the full first moiré harmonic model for twisted AB-stacked bilayer ZrS_2. The interlayer hopping parameters are given in units of meV, while the effective masses are given in units of the bare electron mass m_e. The minimal overlaps with the first two groups of bands are listed at the end.

θ	m_x	m_y	w_2^{AB}	$w_2'^{\mathrm{AB}}$	$w_3'^{\mathrm{AB}}$	$w_4'^{\mathrm{AB}}$	$w_6'^{\mathrm{AB}}$	$w_9'^{\mathrm{AB}}$	$\mathcal{O}_1$	$\mathcal{O}_2$
9.43°	0.27	1.84	−52.19	−4.67	N/A	−23.64	7.33	−7.77	0.9968	0.9756
7.34°	0.26	1.63	−49.34	−4.07	N/A	−26.72	5.42	−7.32	0.9978	0.7689
6.01°	0.27	2.28	−45.42	−2.68	N/A	−22.31	4.29	−4.95	0.9986	0.9921
5.09°	0.25	1.94	−44.99	−2.63	N/A	−23.80	4.83	−4.49	0.9986	0.9965
4.41°	0.24	2.06	−44.25	−2.15	N/A	−23.38	4.76	−4.13	0.9989	0.9970
3.89°	0.24	1.89	−38.95	−1.58	−9.96	−17.26	N/A	−3.94	0.9993	0.9980

TABLE S24. Parameter values of the reduced first moiré harmonic model for twisted AB-stacked bilayer ZrS_2. The interlayer hopping parameters are given in units of meV, while the effective masses are given in units of the bare electron mass m_e. The minimal overlaps with the first two groups of bands are listed at the end.

θ	m_x	m_y	w_1^{AB}	w_2^{AB}	$w_1'^{\mathrm{AB}}$	$w_2'^{\mathrm{AB}}$	$w_3'^{\mathrm{AB}}$	$w_4'^{\mathrm{AB}}$	$w_5'^{\mathrm{AB}}$	$w_6'^{\mathrm{AB}}$	$w_7'^{\mathrm{AB}}$	$\mathcal{O}_1$	$\mathcal{O}_2$
9.43°	0.29	1.86	−2.24	−43.99	1.02	−0.99	−1.68	−14.32	1.25	0.15	3.96	0.9647	0.1384
7.34°	0.29	1.86	−1.48	−41.81	−0.61	−3.66	−24.46	−24.00	0.30	6.21	20.96	0.9782	0.0654
6.01°	0.29	1.86	−1.64	−39.74	−0.85	−3.00	−12.24	−20.71	N/A	3.85	−2.23	0.9829	0.5894
5.09°	0.29	1.86	−1.60	−39.03	−0.83	−3.44	−11.50	−21.69	0.63	3.28	0.33	0.9857	0.7632
4.41°	0.29	1.86	−1.66	−37.32	−0.74	−2.66	−10.08	−22.30	0.46	4.60	0.46	0.9876	0.8792
3.89°	0.29	1.86	−1.57	−35.88	−0.76	−3.33	−9.46	−21.20	0.48	3.41	0.80	0.9876	0.9323

TABLE S25. Parameter values of the first moiré harmonic model with the zero-twist constraints imposed for twisted AB-stacked bilayer ZrS_2. The interlayer hopping parameters are given in units of meV, while the effective masses are given in units of the bare electron mass m_e. The minimal overlaps with the first two groups of bands are listed at the end.

θ	m_x	m_y	w_2^{AB}	$w_3'^{\mathrm{AB}}$	$w_4'^{\mathrm{AB}}$	$w_6'^{\mathrm{AB}}$	$\mathcal{O}_1$	$\mathcal{O}_2$
9.43°	0.29	1.86	−43.99	N/A	−15.04	0.08	0.9613	0.0177
7.34°	0.29	1.86	−41.81	N/A	−26.84	7.98	0.9743	0.0457
6.01°	0.29	1.86	−39.74	−12.11	−16.73	N/A	0.9787	0.4941
5.09°	0.29	1.86	−39.03	−11.46	−18.08	N/A	0.9838	0.7649
4.41°	0.29	1.86	−37.32	−10.29	−16.73	N/A	0.9859	0.8809
3.89°	0.29	1.86	−35.88	−10.05	−17.03	N/A	0.9871	0.9317

TABLE S26. Parameter values of the reduced first moiré harmonic model with the zero-twist constraints imposed for twisted AB-stacked bilayer ZrS_2. The interlayer hopping parameters are given in units of meV, while the effective masses are given in units of the bare electron mass m_e. The minimal overlaps with the first two groups of bands are listed at the end.

D. Numerical results

1. *Band structures along high-symmetry lines*

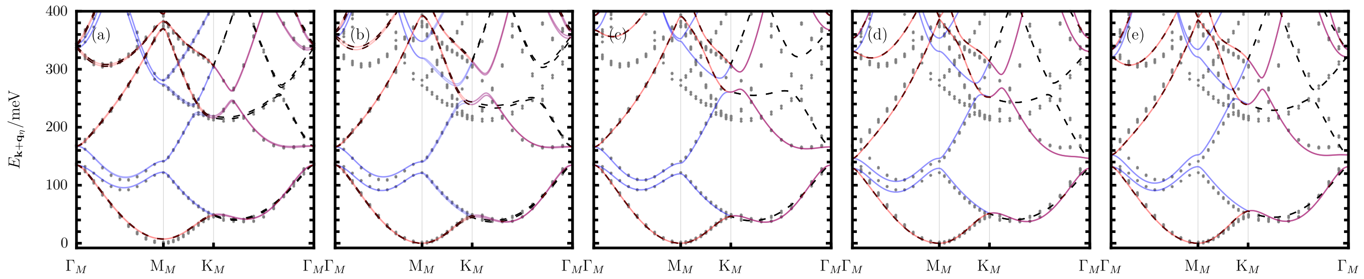

FIG. S26. Model band structures for twisted AA-stacked bilayer $SnSe_2$ at $\theta = 9.43°$. We consider the full continuum model (a), the full first moiré harmonic model (b), the reduced first moiré harmonic model (c), the first moiré harmonic model with the zero-twist constraints imposed (d), and the reduced first moiré harmonic model with the zero-twist constraints imposed (e). The bands of valleys $\eta = 0, 1, 2$ are shown by the blue, dashed black, and red lines, while the *ab initio* band structure is shown by the gray dots.

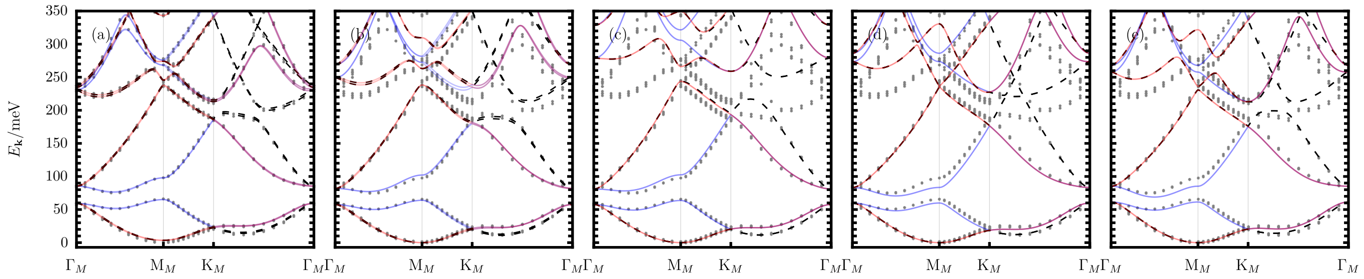

FIG. S27. Model band structures for twisted AA-stacked bilayer $SnSe_2$ at $\theta = 7.34°$. We consider the full continuum model (a), the full first moiré harmonic model (b), the reduced first moiré harmonic model (c), the first moiré harmonic model with the zero-twist constraints imposed (d), and the reduced first moiré harmonic model with the zero-twist constraints imposed (e). The bands of valleys $\eta = 0, 1, 2$ are shown by the blue, dashed black, and red lines, while the *ab initio* band structure is shown by the gray dots.

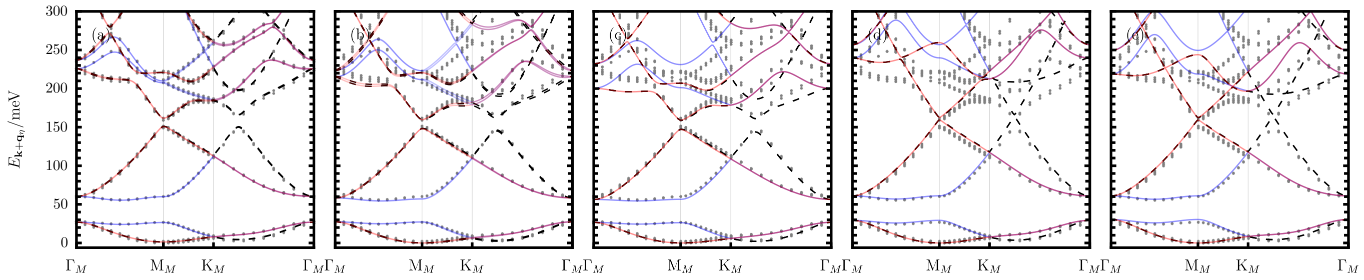

FIG. S28. Model band structures for twisted AA-stacked bilayer $SnSe_2$ at $\theta = 6.01°$. We consider the full continuum model (a), the full first moiré harmonic model (b), the reduced first moiré harmonic model (c), the first moiré harmonic model with the zero-twist constraints imposed (d), and the reduced first moiré harmonic model with the zero-twist constraints imposed (e). The bands of valleys $\eta = 0, 1, 2$ are shown by the blue, dashed black, and red lines, while the *ab initio* band structure is shown by the gray dots.

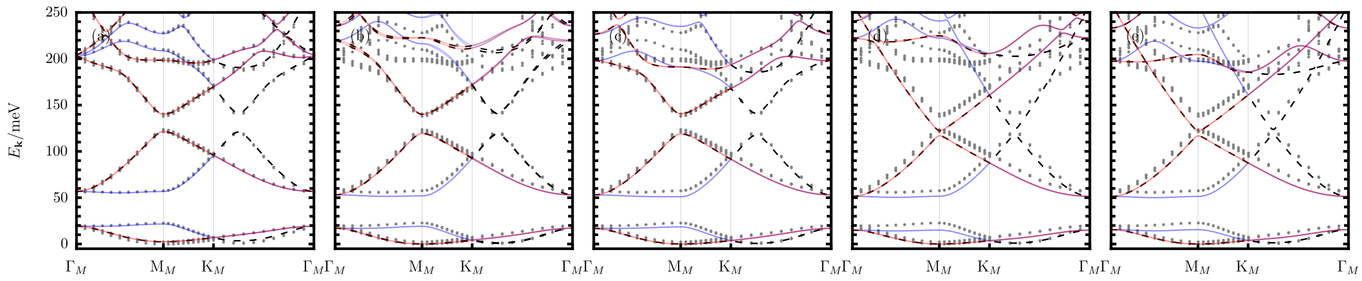

FIG. S29. Model band structures for twisted AA-stacked bilayer $SnSe_2$ at $\theta = 5.09°$. We consider the full continuum model (a), the full first moiré harmonic model (b), the reduced first moiré harmonic model (c), the first moiré harmonic model with the zero-twist constraints imposed (d), and the reduced first moiré harmonic model with the zero-twist constraints imposed (e). The bands of valleys $\eta = 0, 1, 2$ are shown by the blue, dashed black, and red lines, while the *ab initio* band structure is shown by the gray dots.

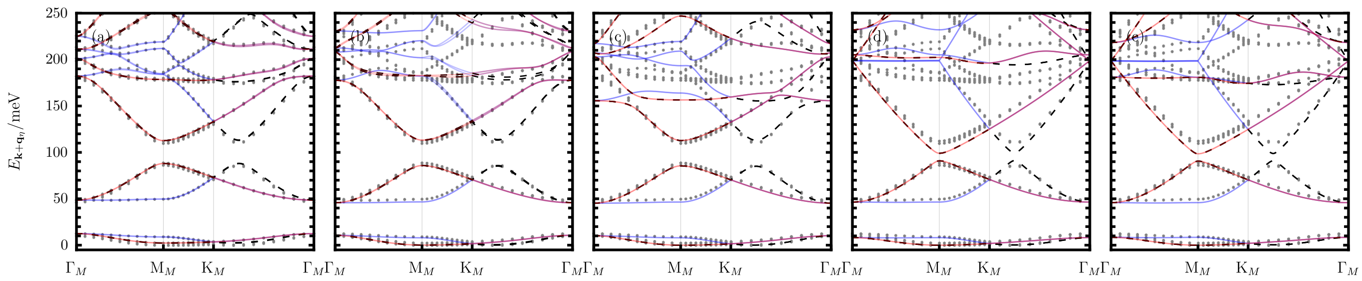

FIG. S30. Model band structures for twisted AA-stacked bilayer $SnSe_2$ at $\theta = 4.41°$. We consider the full continuum model (a), the full first moiré harmonic model (b), the reduced first moiré harmonic model (c), the first moiré harmonic model with the zero-twist constraints imposed (d), and the reduced first moiré harmonic model with the zero-twist constraints imposed (e). The bands of valleys $\eta = 0, 1, 2$ are shown by the blue, dashed black, and red lines, while the *ab initio* band structure is shown by the gray dots.

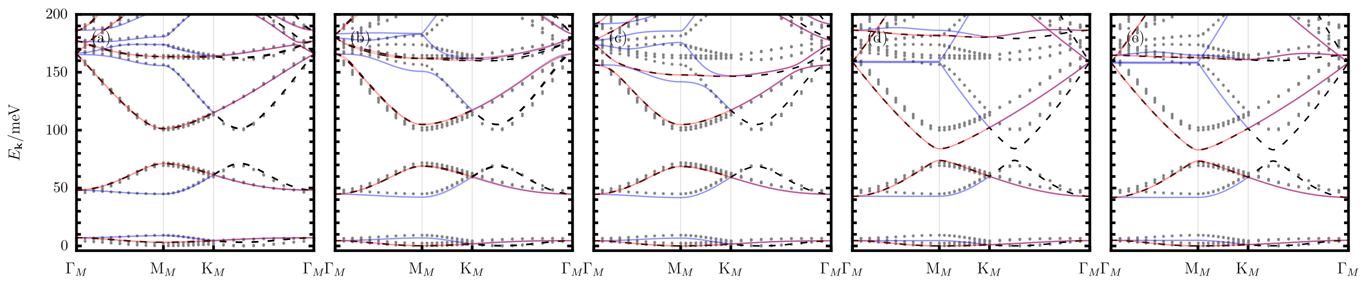

FIG. S31. Model band structures for twisted AA-stacked bilayer $SnSe_2$ at $\theta = 3.89°$. We consider the full continuum model (a), the full first moiré harmonic model (b), the reduced first moiré harmonic model (c), the first moiré harmonic model with the zero-twist constraints imposed (d), and the reduced first moiré harmonic model with the zero-twist constraints imposed (e). The bands of valleys $\eta = 0, 1, 2$ are shown by the blue, dashed black, and red lines, while the *ab initio* band structure is shown by the gray dots.

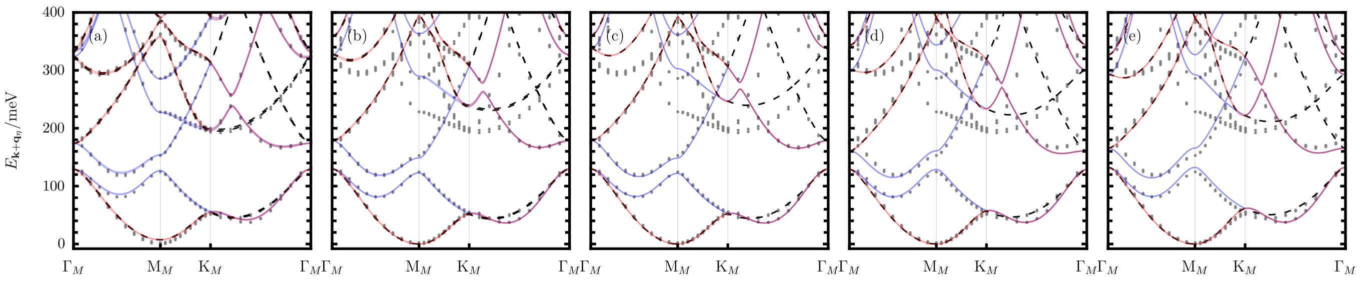

FIG. S32. Model band structures for twisted AB-stacked bilayer $SnSe_2$ at $\theta = 9.43°$. We consider the full continuum model (a), the full first moiré harmonic model (b), the reduced first moiré harmonic model (c), the first moiré harmonic model with the zero-twist constraints imposed (d), and the reduced first moiré harmonic model with the zero-twist constraints imposed (e). The bands of valleys $\eta = 0, 1, 2$ are shown by the blue, dashed black, and red lines, while the *ab initio* band structure is shown by the gray dots.

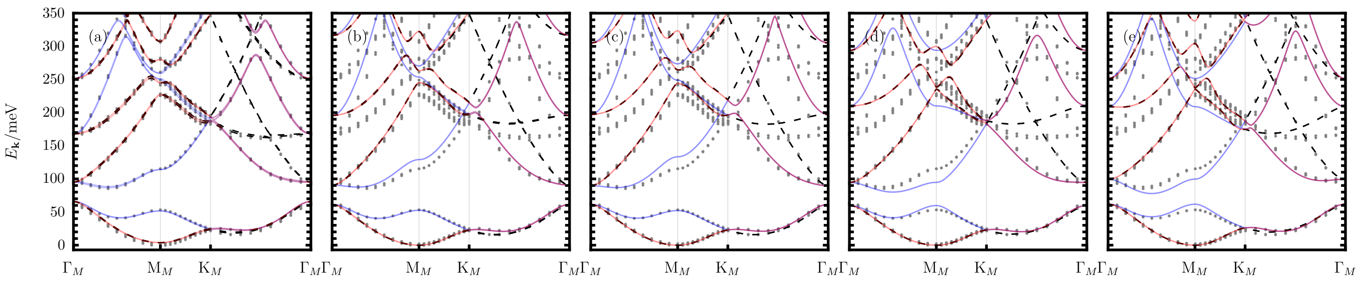

FIG. S33. Model band structures for twisted AB-stacked bilayer $SnSe_2$ at $\theta = 7.34°$. We consider the full continuum model (a), the full first moiré harmonic model (b), the reduced first moiré harmonic model (c), the first moiré harmonic model with the zero-twist constraints imposed (d), and the reduced first moiré harmonic model with the zero-twist constraints imposed (e). The bands of valleys $\eta = 0, 1, 2$ are shown by the blue, dashed black, and red lines, while the *ab initio* band structure is shown by the gray dots.

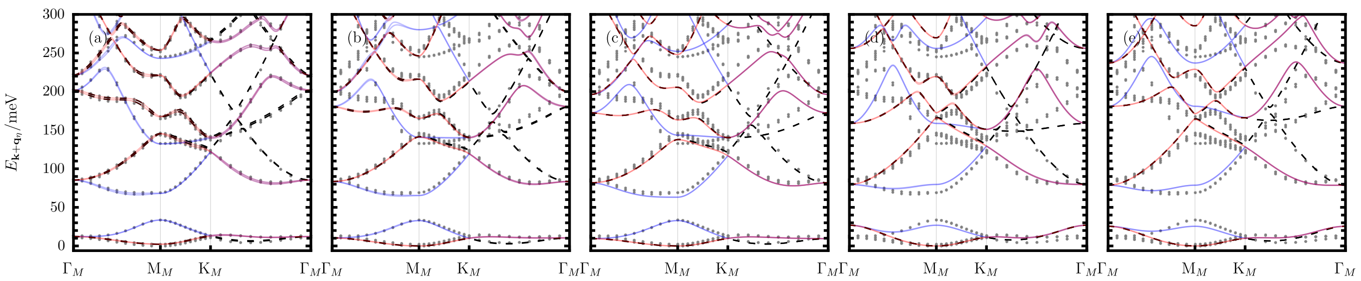

FIG. S34. Model band structures for twisted AB-stacked bilayer $SnSe_2$ at $\theta = 6.01°$. We consider the full continuum model (a), the full first moiré harmonic model (b), the reduced first moiré harmonic model (c), the first moiré harmonic model with the zero-twist constraints imposed (d), and the reduced first moiré harmonic model with the zero-twist constraints imposed (e). The bands of valleys $\eta = 0, 1, 2$ are shown by the blue, dashed black, and red lines, while the *ab initio* band structure is shown by the gray dots.

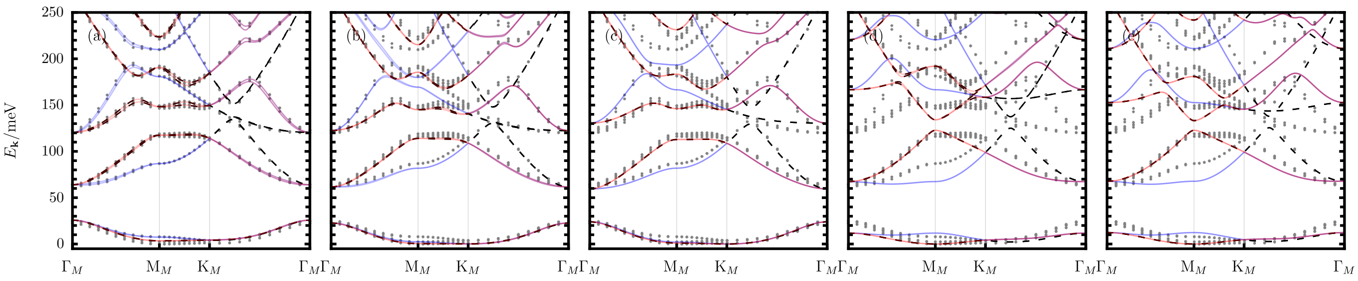

FIG. S35. Model band structures for twisted AB-stacked bilayer $SnSe_2$ at $\theta = 5.09°$. We consider the full continuum model (a), the full first moiré harmonic model (b), the reduced first moiré harmonic model (c), the first moiré harmonic model with the zero-twist constraints imposed (d), and the reduced first moiré harmonic model with the zero-twist constraints imposed (e). The bands of valleys $\eta = 0, 1, 2$ are shown by the blue, dashed black, and red lines, while the *ab initio* band structure is shown by the gray dots.

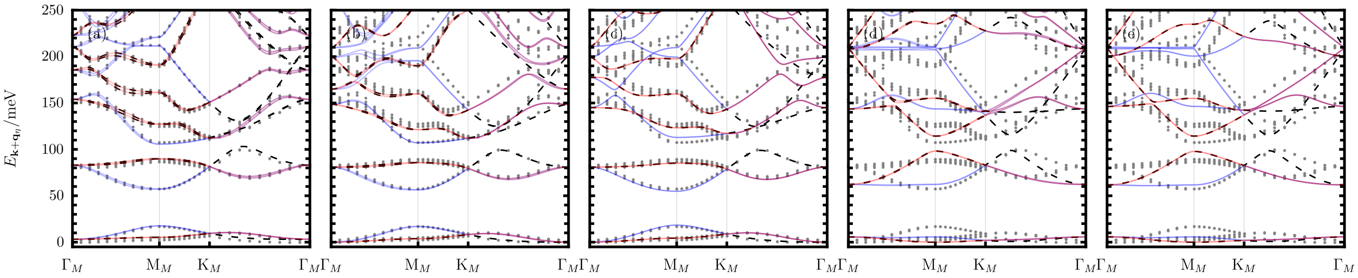

FIG. S36. Model band structures for twisted AB-stacked bilayer $SnSe_2$ at $\theta = 4.41°$. We consider the full continuum model (a), the full first moiré harmonic model (b), the reduced first moiré harmonic model (c), the first moiré harmonic model with the zero-twist constraints imposed (d), and the reduced first moiré harmonic model with the zero-twist constraints imposed (e). The bands of valleys $\eta = 0, 1, 2$ are shown by the blue, dashed black, and red lines, while the *ab initio* band structure is shown by the gray dots.

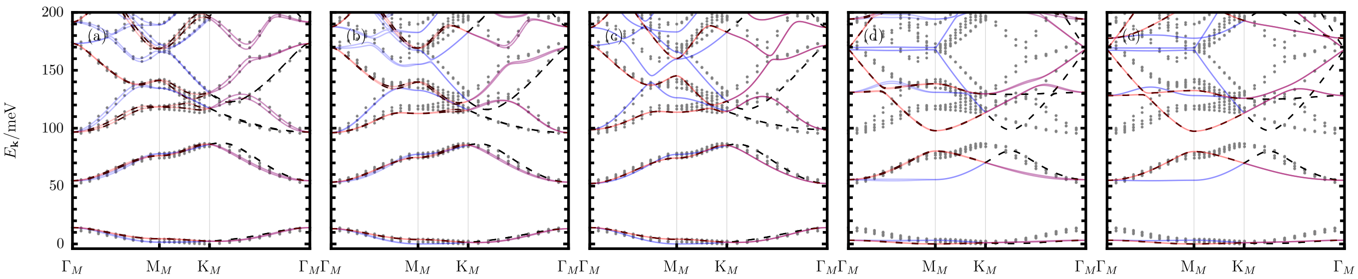

FIG. S37. Model band structures for twisted AB-stacked bilayer $SnSe_2$ at $\theta = 3.89°$. We consider the full continuum model (a), the full first moiré harmonic model (b), the reduced first moiré harmonic model (c), the first moiré harmonic model with the zero-twist constraints imposed (d), and the reduced first moiré harmonic model with the zero-twist constraints imposed (e). The bands of valleys $\eta = 0, 1, 2$ are shown by the blue, dashed black, and red lines, while the *ab initio* band structure is shown by the gray dots.

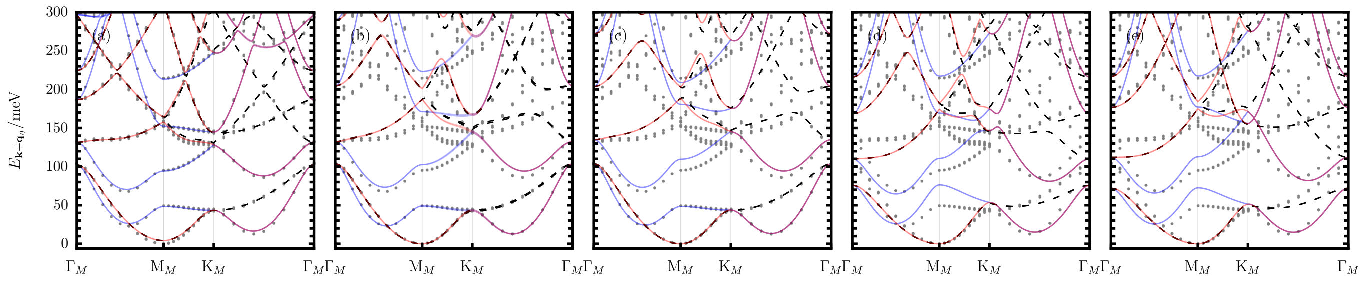

FIG. S38. Model band structures for twisted AA-stacked bilayer ZrS_2 at $\theta = 9.43°$. We consider the full continuum model (a), the full first moiré harmonic model (b), the reduced first moiré harmonic model (c), the first moiré harmonic model with the zero-twist constraints imposed (d), and the reduced first moiré harmonic model with the zero-twist constraints imposed (e). The bands of valleys $\eta = 0, 1, 2$ are shown by the blue, dashed black, and red lines, while the *ab initio* band structure is shown by the gray dots.

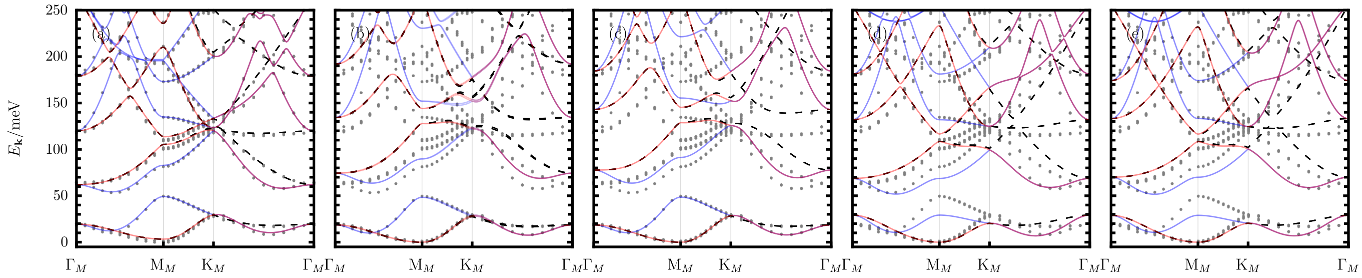

FIG. S39. Model band structures for twisted AA-stacked bilayer ZrS_2 at $\theta = 7.34°$. We consider the full continuum model (a), the full first moiré harmonic model (b), the reduced first moiré harmonic model (c), the first moiré harmonic model with the zero-twist constraints imposed (d), and the reduced first moiré harmonic model with the zero-twist constraints imposed (e). The bands of valleys $\eta = 0, 1, 2$ are shown by the blue, dashed black, and red lines, while the *ab initio* band structure is shown by the gray dots.

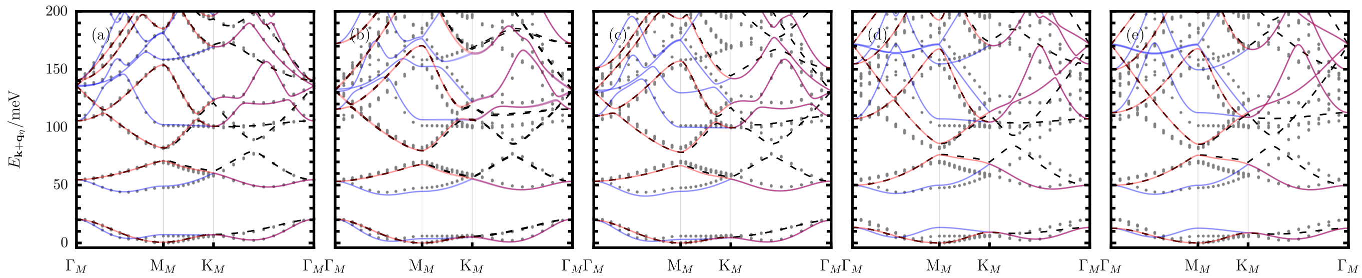

FIG. S40. Model band structures for twisted AA-stacked bilayer ZrS_2 at $\theta = 6.01°$. We consider the full continuum model (a), the full first moiré harmonic model (b), the reduced first moiré harmonic model (c), the first moiré harmonic model with the zero-twist constraints imposed (d), and the reduced first moiré harmonic model with the zero-twist constraints imposed (e). The bands of valleys $\eta = 0, 1, 2$ are shown by the blue, dashed black, and red lines, while the *ab initio* band structure is shown by the gray dots.

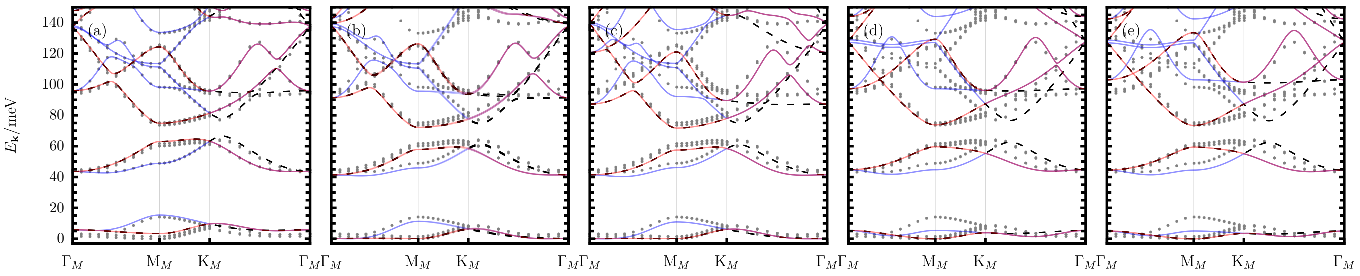

FIG. S41. Model band structures for twisted AA-stacked bilayer ZrS_2 at $\theta = 5.09°$. We consider the full continuum model (a), the full first moiré harmonic model (b), the reduced first moiré harmonic model (c), the first moiré harmonic model with the zero-twist constraints imposed (d), and the reduced first moiré harmonic model with the zero-twist constraints imposed (e). The bands of valleys $\eta = 0, 1, 2$ are shown by the blue, dashed black, and red lines, while the *ab initio* band structure is shown by the gray dots.

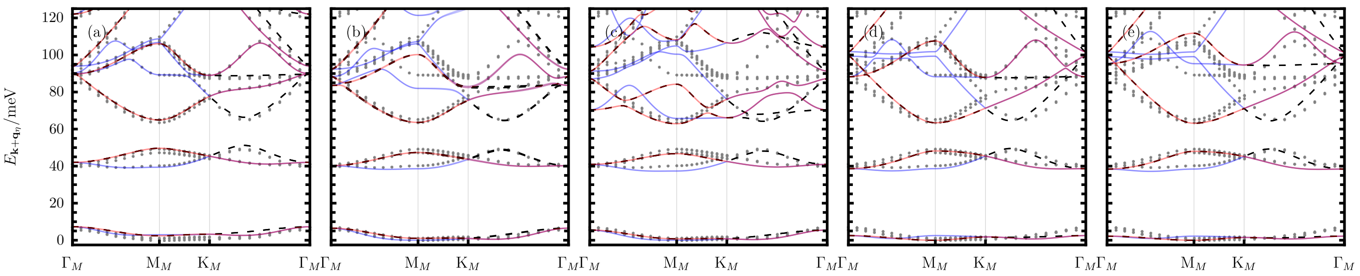

FIG. S42. Model band structures for twisted AA-stacked bilayer ZrS_2 at $\theta = 4.41°$. We consider the full continuum model (a), the full first moiré harmonic model (b), the reduced first moiré harmonic model (c), the first moiré harmonic model with the zero-twist constraints imposed (d), and the reduced first moiré harmonic model with the zero-twist constraints imposed (e). The bands of valleys $\eta = 0, 1, 2$ are shown by the blue, dashed black, and red lines, while the *ab initio* band structure is shown by the gray dots.

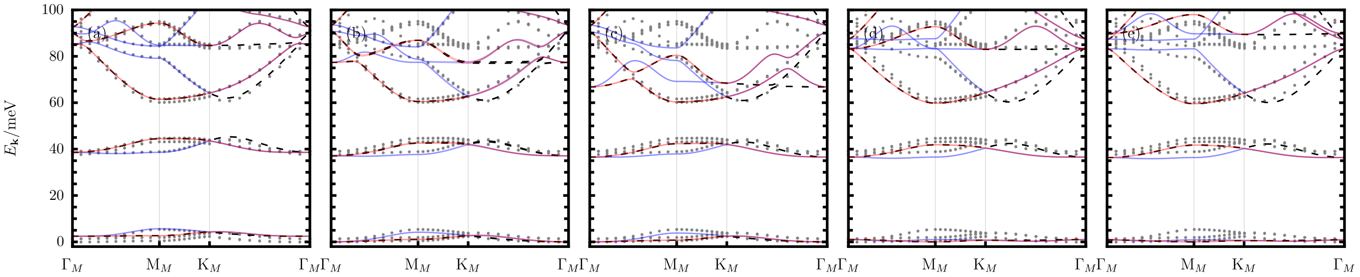

FIG. S43. Model band structures for twisted AA-stacked bilayer ZrS_2 at $\theta = 3.89°$. We consider the full continuum model (a), the full first moiré harmonic model (b), the reduced first moiré harmonic model (c), the first moiré harmonic model with the zero-twist constraints imposed (d), and the reduced first moiré harmonic model with the zero-twist constraints imposed (e). The bands of valleys $\eta = 0, 1, 2$ are shown by the blue, dashed black, and red lines, while the *ab initio* band structure is shown by the gray dots.

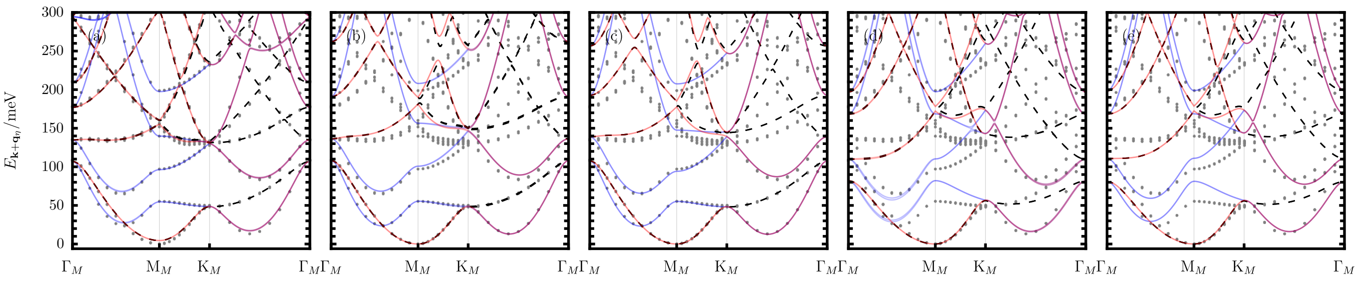

FIG. S44. Model band structures for twisted AB-stacked bilayer ZrS_2 at $\theta = 9.43°$. We consider the full continuum model (a), the full first moiré harmonic model (b), the reduced first moiré harmonic model (c), the first moiré harmonic model with the zero-twist constraints imposed (d), and the reduced first moiré harmonic model with the zero-twist constraints imposed (e). The bands of valleys $\eta = 0, 1, 2$ are shown by the blue, dashed black, and red lines, while the *ab initio* band structure is shown by the gray dots.

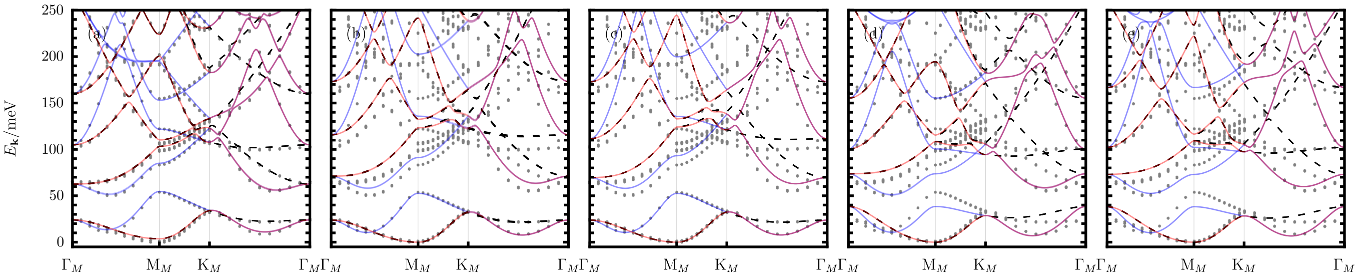

FIG. S45. Model band structures for twisted AB-stacked bilayer ZrS_2 at $\theta = 7.34°$. We consider the full continuum model (a), the full first moiré harmonic model (b), the reduced first moiré harmonic model (c), the first moiré harmonic model with the zero-twist constraints imposed (d), and the reduced first moiré harmonic model with the zero-twist constraints imposed (e). The bands of valleys $\eta = 0, 1, 2$ are shown by the blue, dashed black, and red lines, while the *ab initio* band structure is shown by the gray dots.

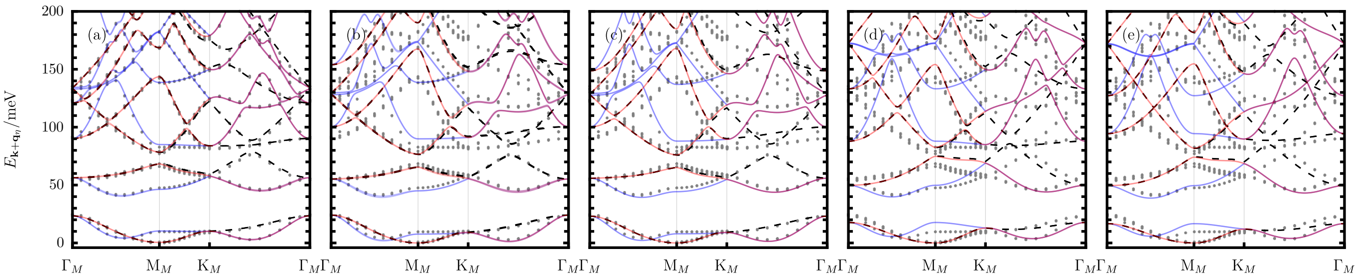

FIG. S46. Model band structures for twisted AB-stacked bilayer ZrS_2 at $\theta = 6.01°$. We consider the full continuum model (a), the full first moiré harmonic model (b), the reduced first moiré harmonic model (c), the first moiré harmonic model with the zero-twist constraints imposed (d), and the reduced first moiré harmonic model with the zero-twist constraints imposed (e). The bands of valleys $\eta = 0, 1, 2$ are shown by the blue, dashed black, and red lines, while the *ab initio* band structure is shown by the gray dots.

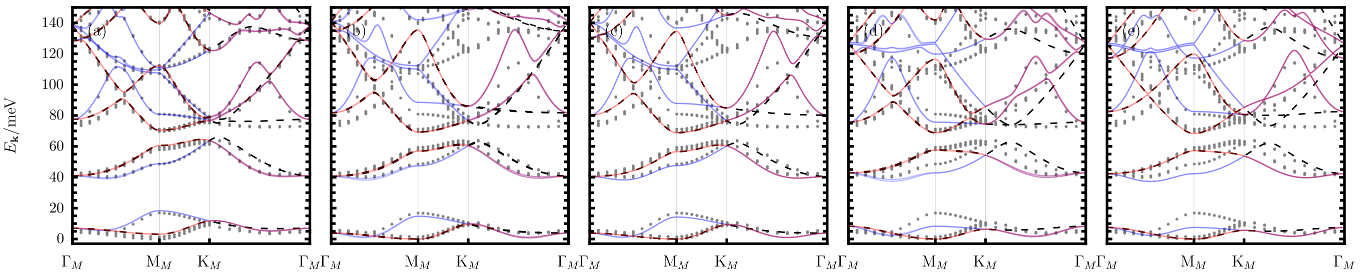

FIG. S47. Model band structures for twisted AB-stacked bilayer ZrS_2 at $\theta = 5.09°$. We consider the full continuum model (a), the full first moiré harmonic model (b), the reduced first moiré harmonic model (c), the first moiré harmonic model with the zero-twist constraints imposed (d), and the reduced first moiré harmonic model with the zero-twist constraints imposed (e). The bands of valleys $\eta = 0, 1, 2$ are shown by the blue, dashed black, and red lines, while the *ab initio* band structure is shown by the gray dots.

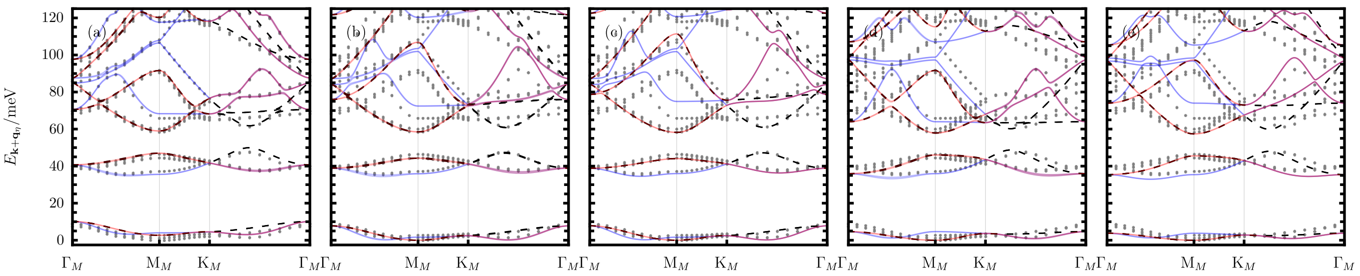

FIG. S48. Model band structures for twisted AB-stacked bilayer ZrS_2 at $\theta = 4.41°$. We consider the full continuum model (a), the full first moiré harmonic model (b), the reduced first moiré harmonic model (c), the first moiré harmonic model with the zero-twist constraints imposed (d), and the reduced first moiré harmonic model with the zero-twist constraints imposed (e). The bands of valleys $\eta = 0, 1, 2$ are shown by the blue, dashed black, and red lines, while the *ab initio* band structure is shown by the gray dots.

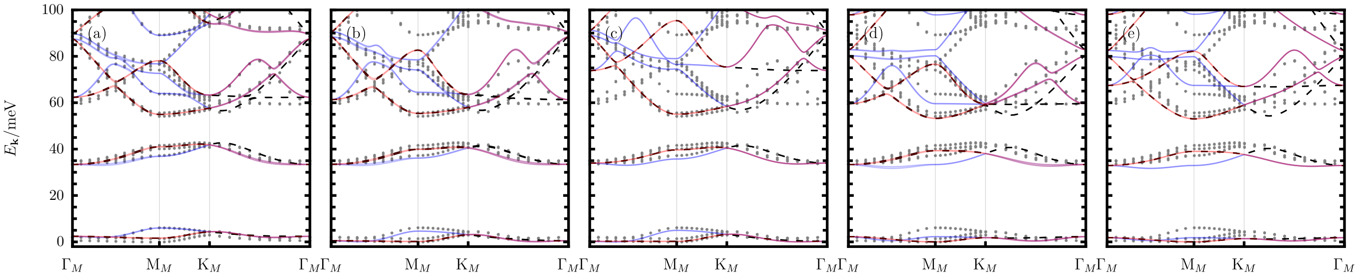

FIG. S49. Model band structures for twisted AB-stacked bilayer ZrS_2 at $\theta = 3.89°$. We consider the full continuum model (a), the full first moiré harmonic model (b), the reduced first moiré harmonic model (c), the first moiré harmonic model with the zero-twist constraints imposed (d), and the reduced first moiré harmonic model with the zero-twist constraints imposed (e). The bands of valleys $\eta = 0, 1, 2$ are shown by the blue, dashed black, and red lines, while the *ab initio* band structure is shown by the gray dots.

2. Spectra of the first gapped conduction bands

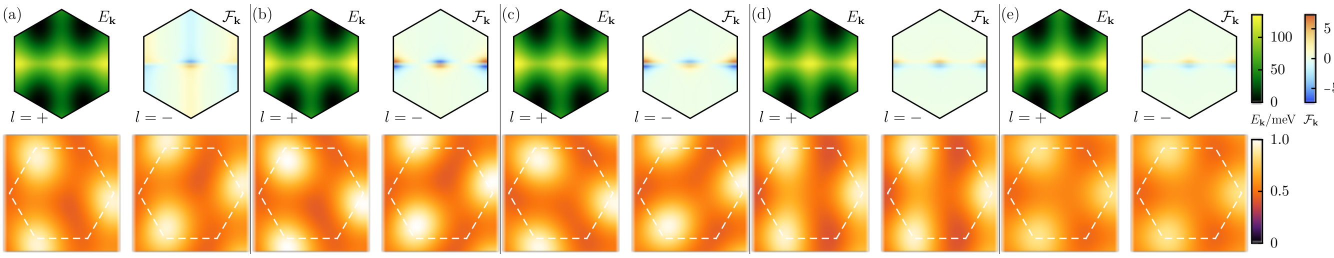

FIG. S50. The first group of conduction bands of twisted AA-stacked bilayer $SnSe_2$ at $\theta = 9.43°$. We consider the full continuum model (a), the full first moiré harmonic model (b), the reduced first moiré harmonic model (c), the first moiré harmonic model with the zero-twist constraints imposed (d), and the reduced first moiré harmonic model with the zero-twist constraints imposed (e). Within each panel, we plot the dispersion of the (approximately or exactly) degenerate group of bands ($E_{\mathbf{k}}$) and their non-abelian Berry curvature ($\mathcal{F}_{\mathbf{k}}$) throughout the first moiré BZ (black hexagon). Additionally, we plot the real space CDD in layer $l = \pm$ within the moiré unit cell (dashed hexagon).

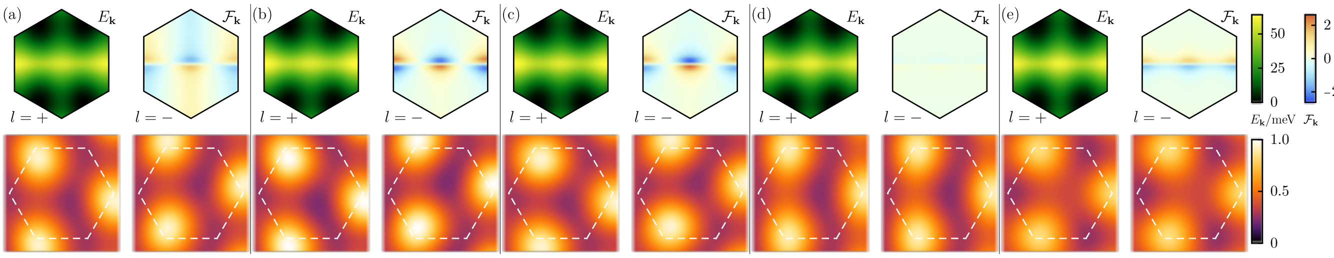

FIG. S51. The first group of conduction bands of twisted AA-stacked bilayer $SnSe_2$ at $\theta = 7.34°$. We consider the full continuum model (a), the full first moiré harmonic model (b), the reduced first moiré harmonic model (c), the first moiré harmonic model with the zero-twist constraints imposed (d), and the reduced first moiré harmonic model with the zero-twist constraints imposed (e). Within each panel, we plot the dispersion of the (approximately or exactly) degenerate group of bands ($E_{\mathbf{k}}$) and their non-abelian Berry curvature ($\mathcal{F}_{\mathbf{k}}$) throughout the first moiré BZ (black hexagon). Additionally, we plot the real space CDD in layer $l = \pm$ within the moiré unit cell (dashed hexagon).

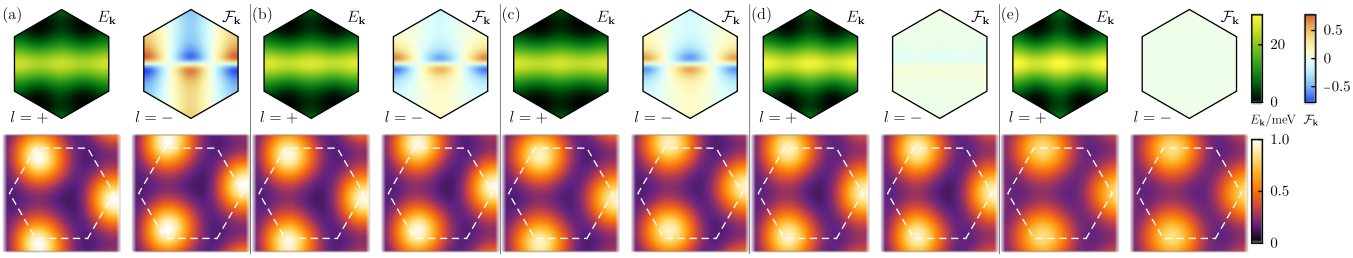

FIG. S52. The first group of conduction bands of twisted AA-stacked bilayer $SnSe_2$ at $\theta = 6.01°$. We consider the full continuum model (a), the full first moiré harmonic model (b), the reduced first moiré harmonic model (c), the first moiré harmonic model with the zero-twist constraints imposed (d), and the reduced first moiré harmonic model with the zero-twist constraints imposed (e). Within each panel, we plot the dispersion of the (approximately or exactly) degenerate group of bands ($E_{\mathbf{k}}$) and their non-abelian Berry curvature ($\mathcal{F}_{\mathbf{k}}$) throughout the first moiré BZ (black hexagon). Additionally, we plot the real space CDD in layer $l = \pm$ within the moiré unit cell (dashed hexagon).

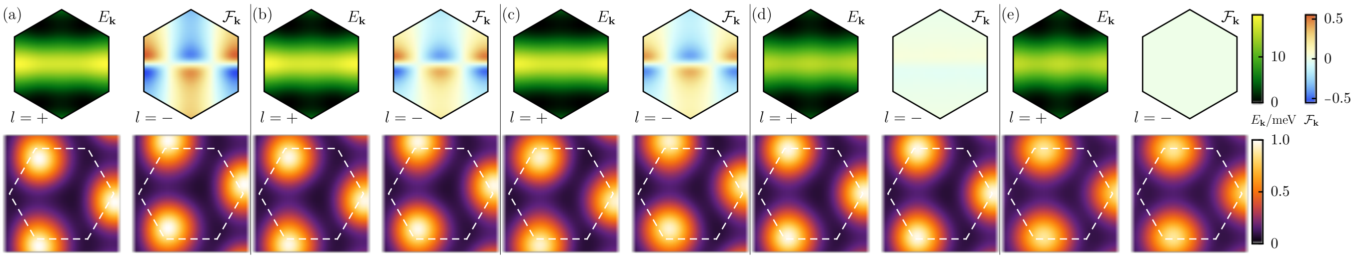

FIG. S53. The first group of conduction bands of twisted AA-stacked bilayer $SnSe_2$ at $\theta = 5.09^\circ$. We consider the full continuum model (a), the full first moiré harmonic model (b), the reduced first moiré harmonic model (c), the first moiré harmonic model with the zero-twist constraints imposed (d), and the reduced first moiré harmonic model with the zero-twist constraints imposed (e). Within each panel, we plot the dispersion of the (approximately or exactly) degenerate group of bands ($E_{\mathbf{k}}$) and their non-abelian Berry curvature ($\mathcal{F}_{\mathbf{k}}$) throughout the first moiré BZ (black hexagon). Additionally, we plot the real space CDD in layer $l = \pm$ within the moiré unit cell (dashed hexagon).

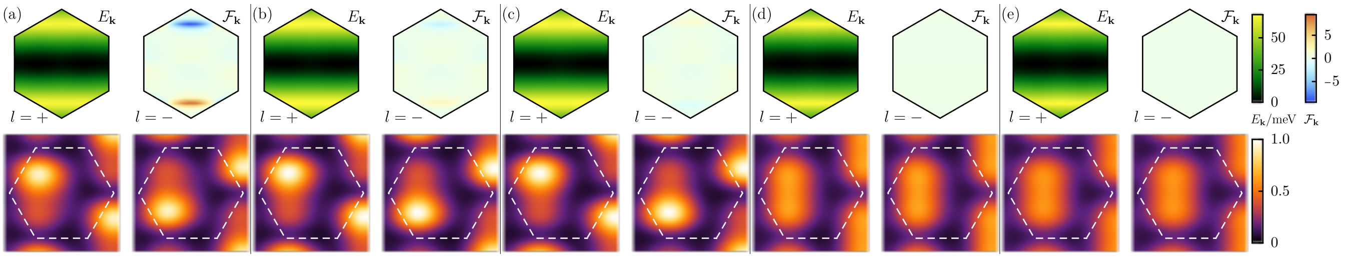

FIG. S54. The second group of conduction bands of twisted AA-stacked bilayer $SnSe_2$ at $\theta = 5.09^\circ$. We consider the full continuum model (a), the full first moiré harmonic model (b), the reduced first moiré harmonic model (c), the first moiré harmonic model with the zero-twist constraints imposed (d), and the reduced first moiré harmonic model with the zero-twist constraints imposed (e). Within each panel, we plot the dispersion of the (approximately or exactly) degenerate group of bands ($E_{\mathbf{k}}$) and their non-abelian Berry curvature ($\mathcal{F}_{\mathbf{k}}$) throughout the first moiré BZ (black hexagon). Additionally, we plot the real space CDD in layer $l = \pm$ within the moiré unit cell (dashed hexagon).

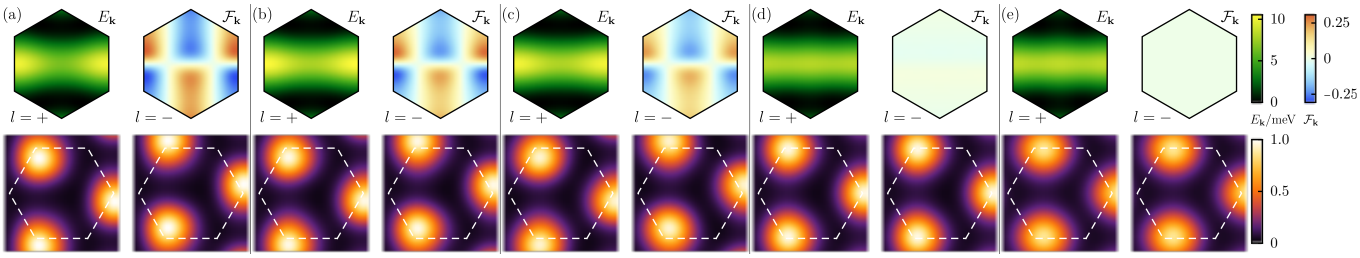

FIG. S55. The first group of conduction bands of twisted AA-stacked bilayer $SnSe_2$ at $\theta = 4.41^\circ$. We consider the full continuum model (a), the full first moiré harmonic model (b), the reduced first moiré harmonic model (c), the first moiré harmonic model with the zero-twist constraints imposed (d), and the reduced first moiré harmonic model with the zero-twist constraints imposed (e). Within each panel, we plot the dispersion of the (approximately or exactly) degenerate group of bands ($E_{\mathbf{k}}$) and their non-abelian Berry curvature ($\mathcal{F}_{\mathbf{k}}$) throughout the first moiré BZ (black hexagon). Additionally, we plot the real space CDD in layer $l = \pm$ within the moiré unit cell (dashed hexagon).

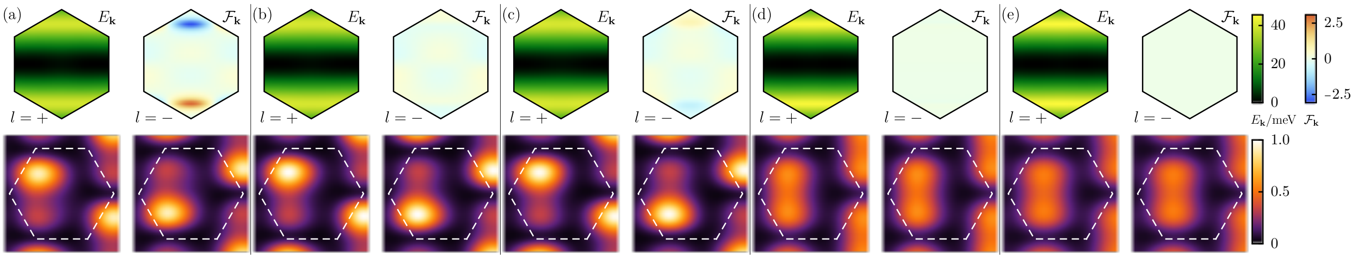

FIG. S56. The second group of conduction bands of twisted AA-stacked bilayer $SnSe_2$ at $\theta = 4.41°$. We consider the full continuum model (a), the full first moiré harmonic model (b), the reduced first moiré harmonic model (c), the first moiré harmonic model with the zero-twist constraints imposed (d), and the reduced first moiré harmonic model with the zero-twist constraints imposed (e). Within each panel, we plot the dispersion of the (approximately or exactly) degenerate group of bands ($E_{\mathbf{k}}$) and their non-abelian Berry curvature ($\mathcal{F}_{\mathbf{k}}$) throughout the first moiré BZ (black hexagon). Additionally, we plot the real space CDD in layer $l = \pm$ within the moiré unit cell (dashed hexagon).

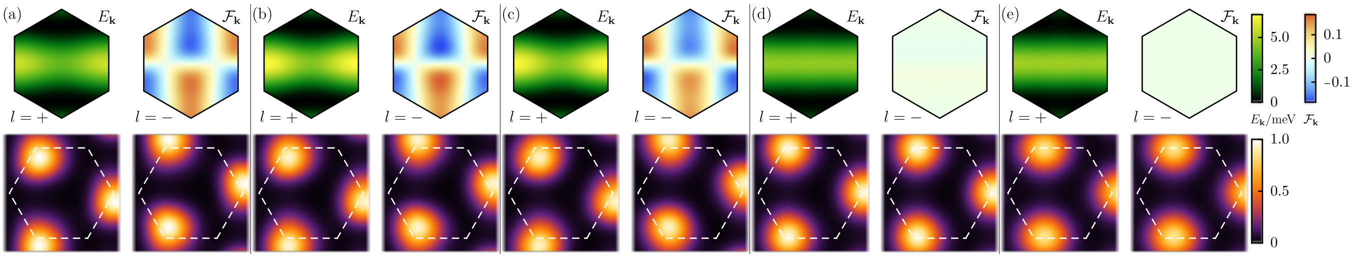

FIG. S57. The first group of conduction bands of twisted AA-stacked bilayer $SnSe_2$ at $\theta = 3.89°$. We consider the full continuum model (a), the full first moiré harmonic model (b), the reduced first moiré harmonic model (c), the first moiré harmonic model with the zero-twist constraints imposed (d), and the reduced first moiré harmonic model with the zero-twist constraints imposed (e). Within each panel, we plot the dispersion of the (approximately or exactly) degenerate group of bands ($E_{\mathbf{k}}$) and their non-abelian Berry curvature ($\mathcal{F}_{\mathbf{k}}$) throughout the first moiré BZ (black hexagon). Additionally, we plot the real space CDD in layer $l = \pm$ within the moiré unit cell (dashed hexagon).

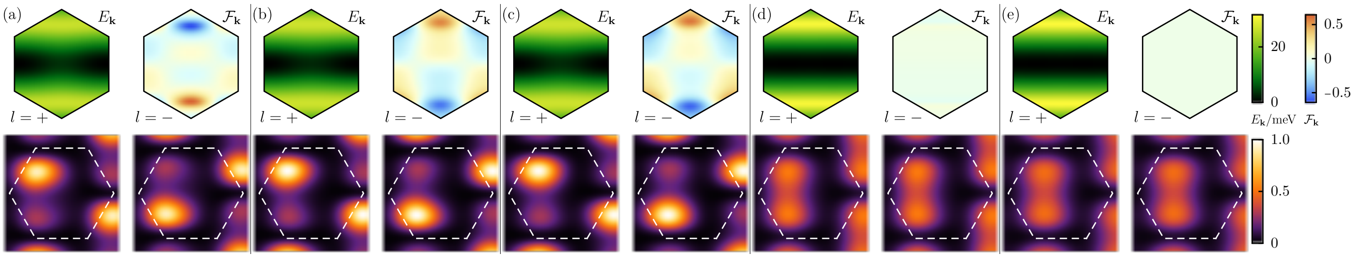

FIG. S58. The second group of conduction bands of twisted AA-stacked bilayer $SnSe_2$ at $\theta = 3.89°$. We consider the full continuum model (a), the full first moiré harmonic model (b), the reduced first moiré harmonic model (c), the first moiré harmonic model with the zero-twist constraints imposed (d), and the reduced first moiré harmonic model with the zero-twist constraints imposed (e). Within each panel, we plot the dispersion of the (approximately or exactly) degenerate group of bands ($E_{\mathbf{k}}$) and their non-abelian Berry curvature ($\mathcal{F}_{\mathbf{k}}$) throughout the first moiré BZ (black hexagon). Additionally, we plot the real space CDD in layer $l = \pm$ within the moiré unit cell (dashed hexagon).

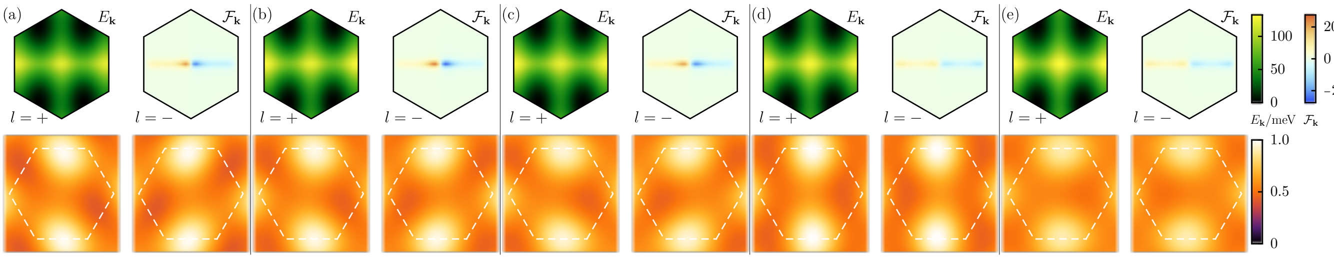

FIG. S59. The first group of conduction bands of twisted AB-stacked bilayer $SnSe_2$ at $\theta = 9.43°$. We consider the full continuum model (a), the full first moiré harmonic model (b), the reduced first moiré harmonic model (c), the first moiré harmonic model with the zero-twist constraints imposed (d), and the reduced first moiré harmonic model with the zero-twist constraints imposed (e). Within each panel, we plot the dispersion of the (approximately or exactly) degenerate group of bands ($E_{\mathbf{k}}$) and their non-abelian Berry curvature ($\mathcal{F}_{\mathbf{k}}$) throughout the first moiré BZ (black hexagon). Additionally, we plot the real space CDD in layer $l = \pm$ within the moiré unit cell (dashed hexagon).

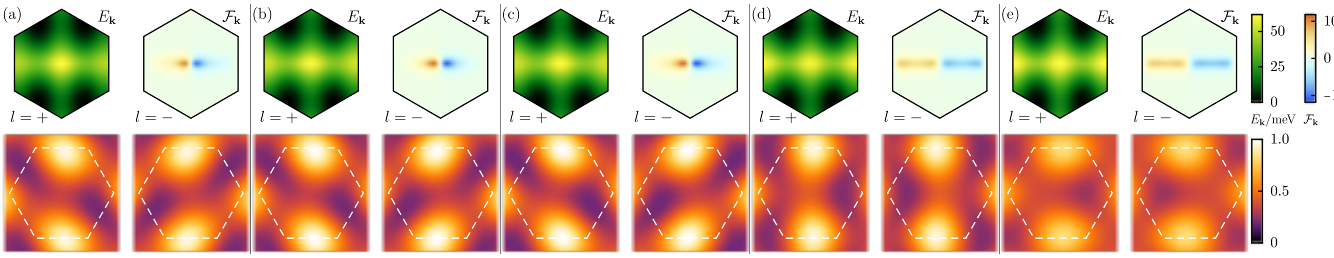

FIG. S60. The first group of conduction bands of twisted AB-stacked bilayer $SnSe_2$ at $\theta = 7.34°$. We consider the full continuum model (a), the full first moiré harmonic model (b), the reduced first moiré harmonic model (c), the first moiré harmonic model with the zero-twist constraints imposed (d), and the reduced first moiré harmonic model with the zero-twist constraints imposed (e). Within each panel, we plot the dispersion of the (approximately or exactly) degenerate group of bands ($E_{\mathbf{k}}$) and their non-abelian Berry curvature ($\mathcal{F}_{\mathbf{k}}$) throughout the first moiré BZ (black hexagon). Additionally, we plot the real space CDD in layer $l = \pm$ within the moiré unit cell (dashed hexagon).

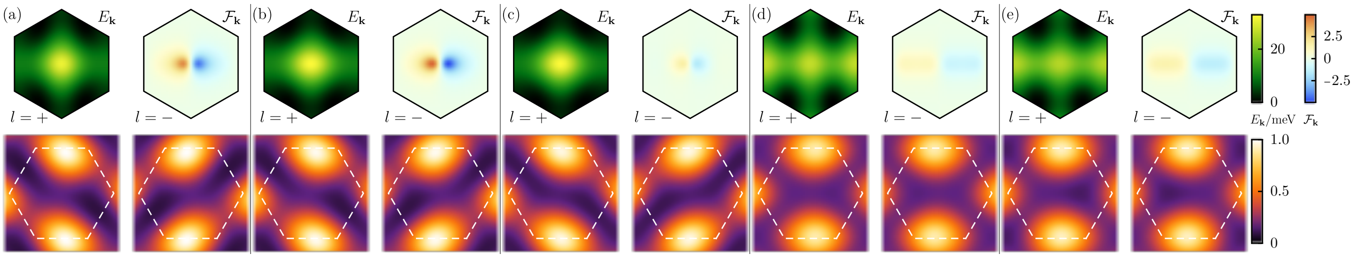

FIG. S61. The first group of conduction bands of twisted AB-stacked bilayer $SnSe_2$ at $\theta = 6.01°$. We consider the full continuum model (a), the full first moiré harmonic model (b), the reduced first moiré harmonic model (c), the first moiré harmonic model with the zero-twist constraints imposed (d), and the reduced first moiré harmonic model with the zero-twist constraints imposed (e). Within each panel, we plot the dispersion of the (approximately or exactly) degenerate group of bands ($E_{\mathbf{k}}$) and their non-abelian Berry curvature ($\mathcal{F}_{\mathbf{k}}$) throughout the first moiré BZ (black hexagon). Additionally, we plot the real space CDD in layer $l = \pm$ within the moiré unit cell (dashed hexagon).

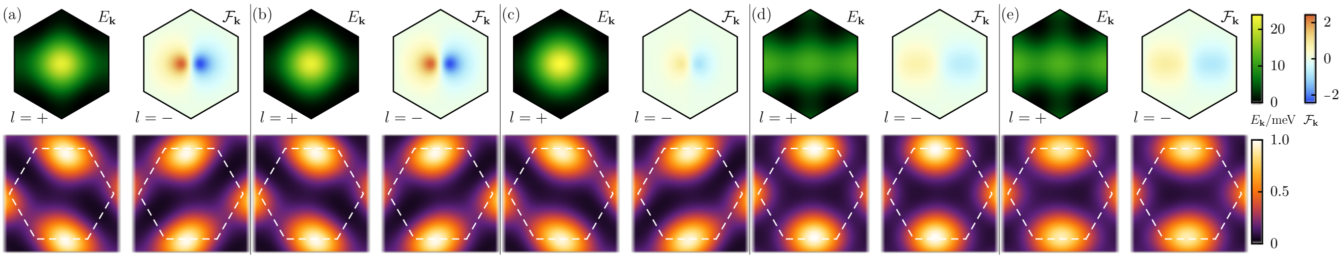

FIG. S62. The first group of conduction bands of twisted AB-stacked bilayer $SnSe_2$ at $\theta = 5.09°$. We consider the full continuum model (a), the full first moiré harmonic model (b), the reduced first moiré harmonic model (c), the first moiré harmonic model with the zero-twist constraints imposed (d), and the reduced first moiré harmonic model with the zero-twist constraints imposed (e). Within each panel, we plot the dispersion of the (approximately or exactly) degenerate group of bands ($E_{\mathbf{k}}$) and their non-abelian Berry curvature ($\mathcal{F}_{\mathbf{k}}$) throughout the first moiré BZ (black hexagon). Additionally, we plot the real space CDD in layer $l = \pm$ within the moiré unit cell (dashed hexagon).

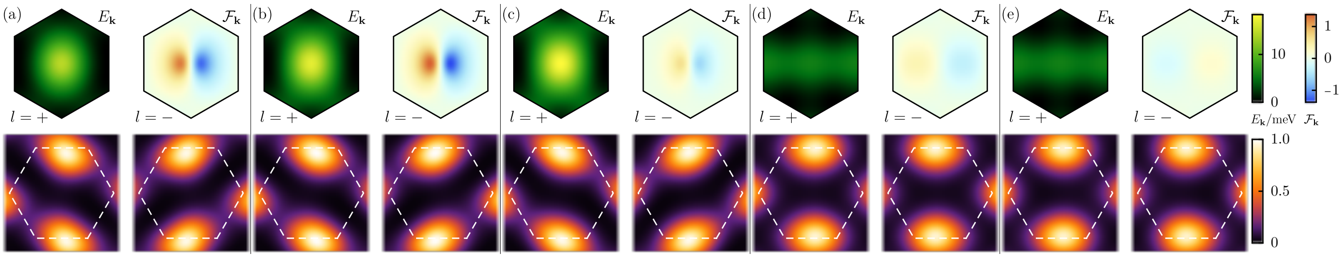

FIG. S63. The first group of conduction bands of twisted AB-stacked bilayer $SnSe_2$ at $\theta = 4.41°$. We consider the full continuum model (a), the full first moiré harmonic model (b), the reduced first moiré harmonic model (c), the first moiré harmonic model with the zero-twist constraints imposed (d), and the reduced first moiré harmonic model with the zero-twist constraints imposed (e). Within each panel, we plot the dispersion of the (approximately or exactly) degenerate group of bands ($E_{\mathbf{k}}$) and their non-abelian Berry curvature ($\mathcal{F}_{\mathbf{k}}$) throughout the first moiré BZ (black hexagon). Additionally, we plot the real space CDD in layer $l = \pm$ within the moiré unit cell (dashed hexagon).

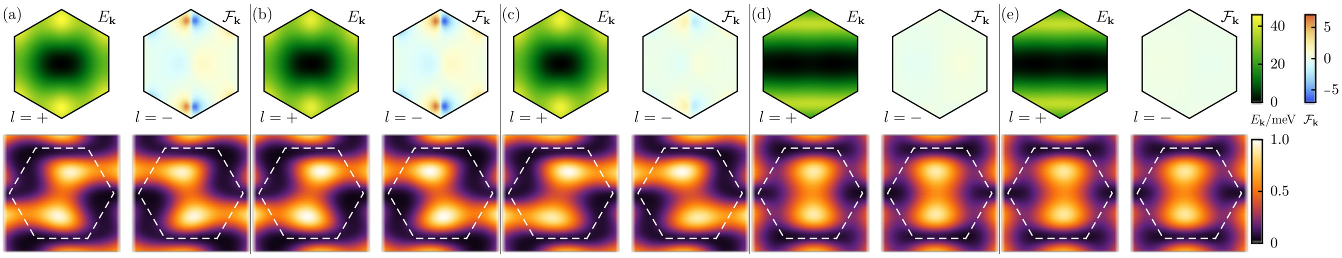

FIG. S64. The second group of conduction bands of twisted AB-stacked bilayer $SnSe_2$ at $\theta = 4.41°$. We consider the full continuum model (a), the full first moiré harmonic model (b), the reduced first moiré harmonic model (c), the first moiré harmonic model with the zero-twist constraints imposed (d), and the reduced first moiré harmonic model with the zero-twist constraints imposed (e). Within each panel, we plot the dispersion of the (approximately or exactly) degenerate group of bands ($E_{\mathbf{k}}$) and their non-abelian Berry curvature ($\mathcal{F}_{\mathbf{k}}$) throughout the first moiré BZ (black hexagon). Additionally, we plot the real space CDD in layer $l = \pm$ within the moiré unit cell (dashed hexagon).

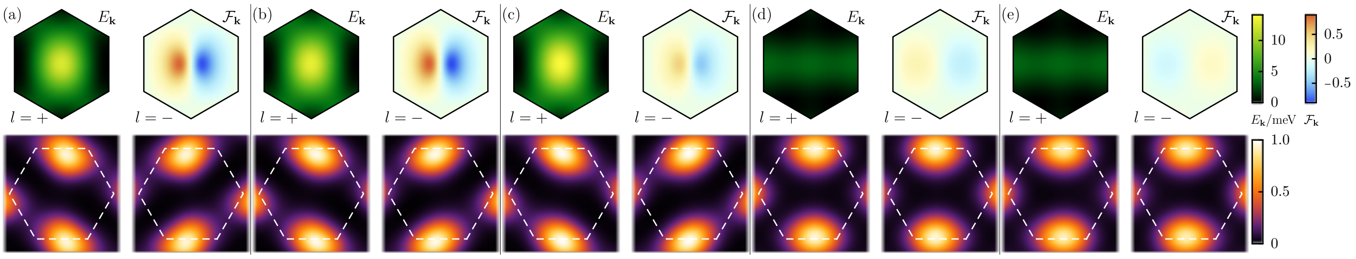

FIG. S65. The first group of conduction bands of twisted AB-stacked bilayer $SnSe_2$ at $\theta = 3.89°$. We consider the full continuum model (a), the full first moiré harmonic model (b), the reduced first moiré harmonic model (c), the first moiré harmonic model with the zero-twist constraints imposed (d), and the reduced first moiré harmonic model with the zero-twist constraints imposed (e). Within each panel, we plot the dispersion of the (approximately or exactly) degenerate group of bands ($E_{\mathbf{k}}$) and their non-abelian Berry curvature ($\mathcal{F}_{\mathbf{k}}$) throughout the first moiré BZ (black hexagon). Additionally, we plot the real space CDD in layer $l = \pm$ within the moiré unit cell (dashed hexagon).

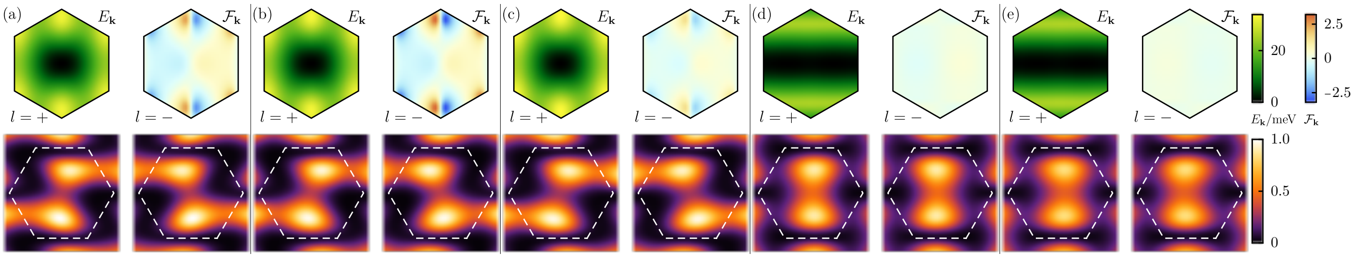

FIG. S66. The second group of conduction bands of twisted AB-stacked bilayer $SnSe_2$ at $\theta = 3.89°$. We consider the full continuum model (a), the full first moiré harmonic model (b), the reduced first moiré harmonic model (c), the first moiré harmonic model with the zero-twist constraints imposed (d), and the reduced first moiré harmonic model with the zero-twist constraints imposed (e). Within each panel, we plot the dispersion of the (approximately or exactly) degenerate group of bands ($E_{\mathbf{k}}$) and their non-abelian Berry curvature ($\mathcal{F}_{\mathbf{k}}$) throughout the first moiré BZ (black hexagon). Additionally, we plot the real space CDD in layer $l = \pm$ within the moiré unit cell (dashed hexagon).

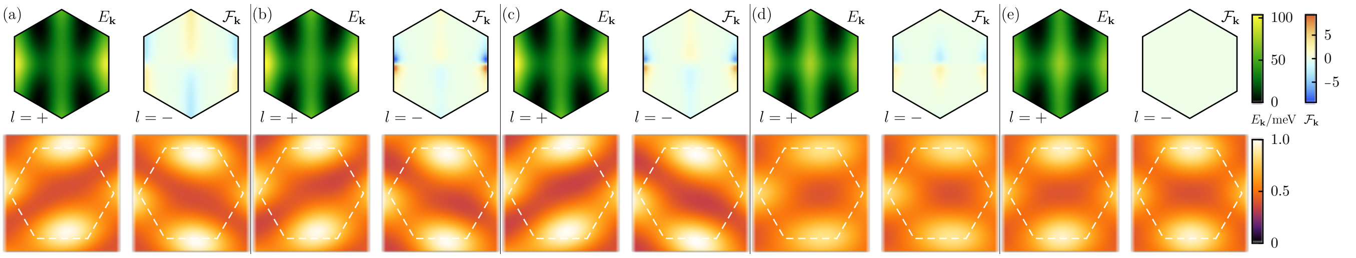

FIG. S67. The first group of conduction bands of twisted AA-stacked bilayer ZrS_2 at $\theta = 9.43°$. We consider the full continuum model (a), the full first moiré harmonic model (b), the reduced first moiré harmonic model (c), the first moiré harmonic model with the zero-twist constraints imposed (d), and the reduced first moiré harmonic model with the zero-twist constraints imposed (e). Within each panel, we plot the dispersion of the (approximately or exactly) degenerate group of bands ($E_{\mathbf{k}}$) and their non-abelian Berry curvature ($\mathcal{F}_{\mathbf{k}}$) throughout the first moiré BZ (black hexagon). Additionally, we plot the real space CDD in layer $l = \pm$ within the moiré unit cell (dashed hexagon).

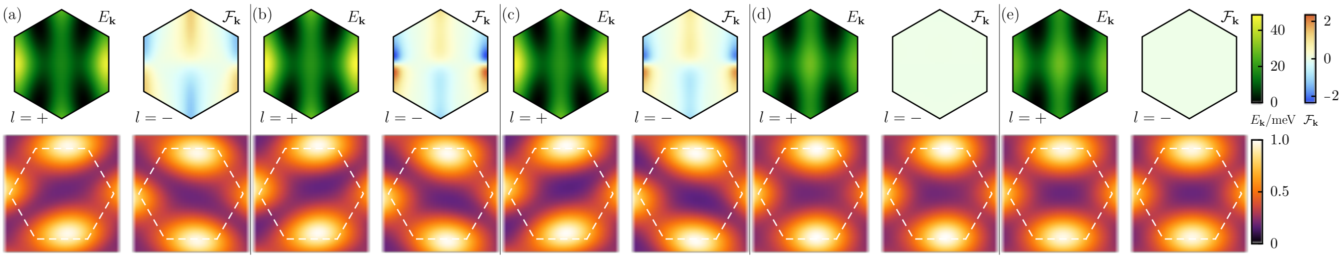

FIG. S68. The first group of conduction bands of twisted AA-stacked bilayer ZrS_2 at $\theta = 7.34^\circ$. We consider the full continuum model (a), the full first moiré harmonic model (b), the reduced first moiré harmonic model (c), the first moiré harmonic model with the zero-twist constraints imposed (d), and the reduced first moiré harmonic model with the zero-twist constraints imposed (e). Within each panel, we plot the dispersion of the (approximately or exactly) degenerate group of bands ($E_{\mathbf{k}}$) and their non-abelian Berry curvature ($\mathcal{F}_{\mathbf{k}}$) throughout the first moiré BZ (black hexagon). Additionally, we plot the real space CDD in layer $l = \pm$ within the moiré unit cell (dashed hexagon).

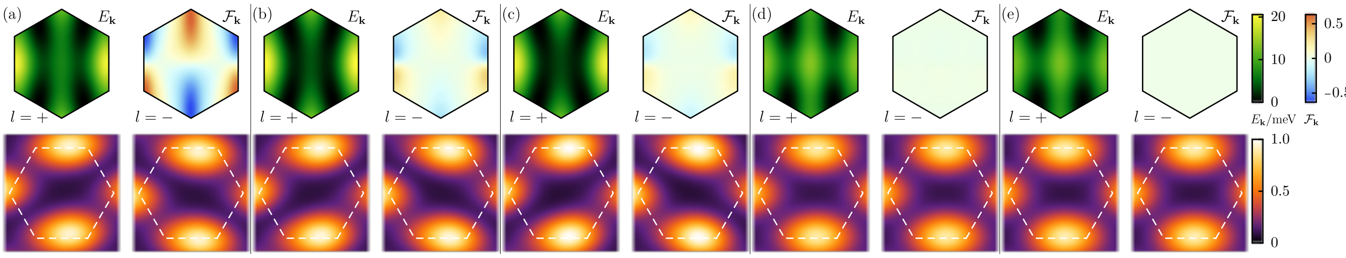

FIG. S69. The first group of conduction bands of twisted AA-stacked bilayer ZrS_2 at $\theta = 6.01^\circ$. We consider the full continuum model (a), the full first moiré harmonic model (b), the reduced first moiré harmonic model (c), the first moiré harmonic model with the zero-twist constraints imposed (d), and the reduced first moiré harmonic model with the zero-twist constraints imposed (e). Within each panel, we plot the dispersion of the (approximately or exactly) degenerate group of bands ($E_{\mathbf{k}}$) and their non-abelian Berry curvature ($\mathcal{F}_{\mathbf{k}}$) throughout the first moiré BZ (black hexagon). Additionally, we plot the real space CDD in layer $l = \pm$ within the moiré unit cell (dashed hexagon).

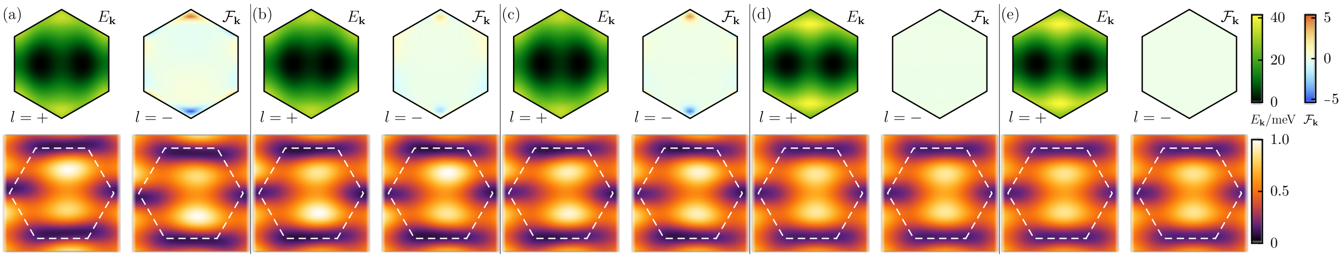

FIG. S70. The second group of conduction bands of twisted AA-stacked bilayer ZrS_2 at $\theta = 6.01^\circ$. We consider the full continuum model (a), the full first moiré harmonic model (b), the reduced first moiré harmonic model (c), the first moiré harmonic model with the zero-twist constraints imposed (d), and the reduced first moiré harmonic model with the zero-twist constraints imposed (e). Within each panel, we plot the dispersion of the (approximately or exactly) degenerate group of bands ($E_{\mathbf{k}}$) and their non-abelian Berry curvature ($\mathcal{F}_{\mathbf{k}}$) throughout the first moiré BZ (black hexagon). Additionally, we plot the real space CDD in layer $l = \pm$ within the moiré unit cell (dashed hexagon).

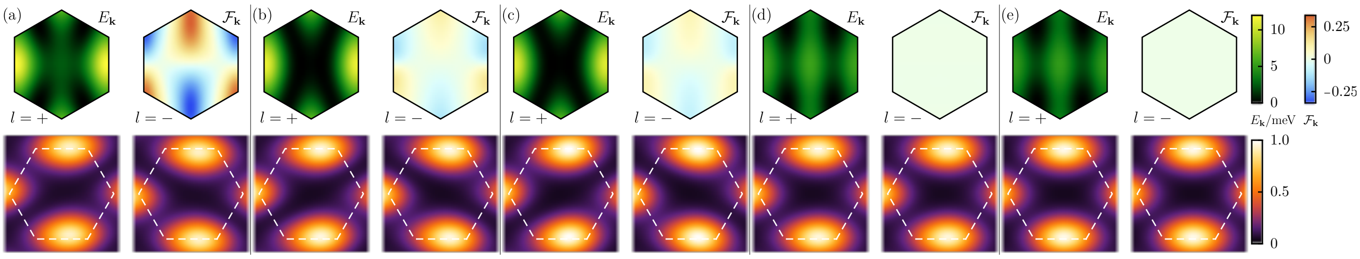

FIG. S71. The first group of conduction bands of twisted AA-stacked bilayer ZrS_2 at $\theta = 5.09°$. We consider the full continuum model (a), the full first moiré harmonic model (b), the reduced first moiré harmonic model (c), the first moiré harmonic model with the zero-twist constraints imposed (d), and the reduced first moiré harmonic model with the zero-twist constraints imposed (e). Within each panel, we plot the dispersion of the (approximately or exactly) degenerate group of bands ($E_{\mathbf{k}}$) and their non-abelian Berry curvature ($\mathcal{F}_{\mathbf{k}}$) throughout the first moiré BZ (black hexagon). Additionally, we plot the real space CDD in layer $l = \pm$ within the moiré unit cell (dashed hexagon).

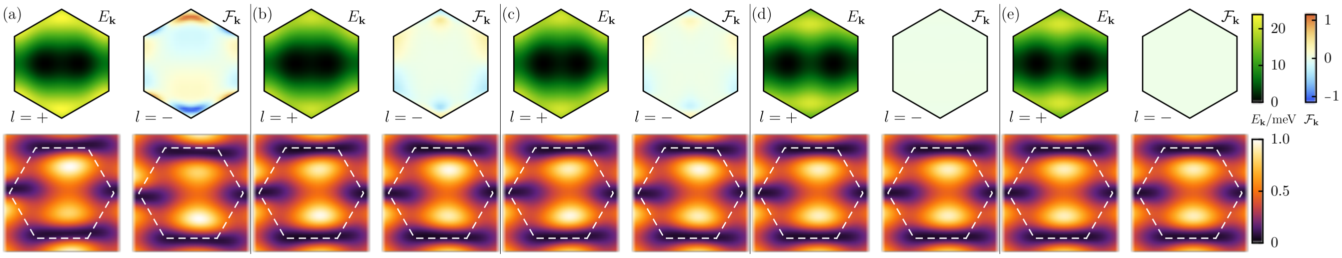

FIG. S72. The second group of conduction bands of twisted AA-stacked bilayer ZrS_2 at $\theta = 5.09°$. We consider the full continuum model (a), the full first moiré harmonic model (b), the reduced first moiré harmonic model (c), the first moiré harmonic model with the zero-twist constraints imposed (d), and the reduced first moiré harmonic model with the zero-twist constraints imposed (e). Within each panel, we plot the dispersion of the (approximately or exactly) degenerate group of bands ($E_{\mathbf{k}}$) and their non-abelian Berry curvature ($\mathcal{F}_{\mathbf{k}}$) throughout the first moiré BZ (black hexagon). Additionally, we plot the real space CDD in layer $l = \pm$ within the moiré unit cell (dashed hexagon).

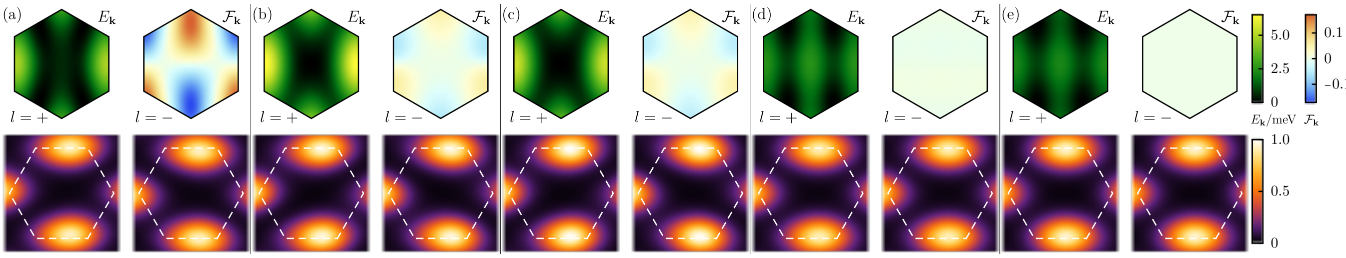

FIG. S73. The first group of conduction bands of twisted AA-stacked bilayer ZrS_2 at $\theta = 4.41°$. We consider the full continuum model (a), the full first moiré harmonic model (b), the reduced first moiré harmonic model (c), the first moiré harmonic model with the zero-twist constraints imposed (d), and the reduced first moiré harmonic model with the zero-twist constraints imposed (e). Within each panel, we plot the dispersion of the (approximately or exactly) degenerate group of bands ($E_{\mathbf{k}}$) and their non-abelian Berry curvature ($\mathcal{F}_{\mathbf{k}}$) throughout the first moiré BZ (black hexagon). Additionally, we plot the real space CDD in layer $l = \pm$ within the moiré unit cell (dashed hexagon).

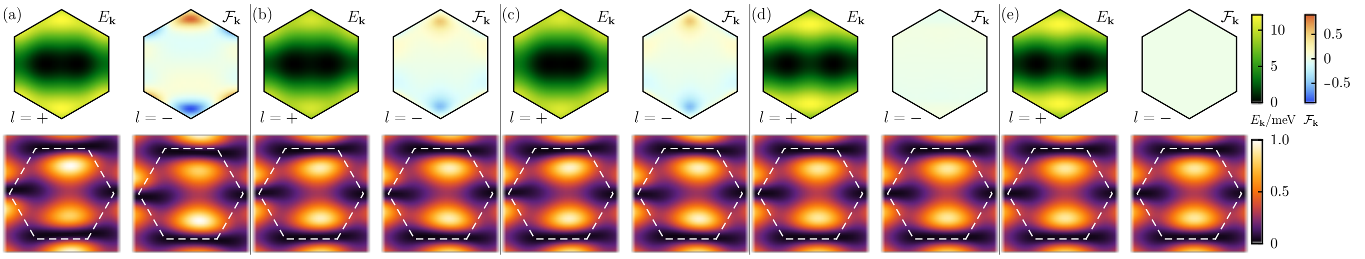

FIG. S74. The second group of conduction bands of twisted AA-stacked bilayer ZrS_2 at $\theta = 4.41°$. We consider the full continuum model (a), the full first moiré harmonic model (b), the reduced first moiré harmonic model (c), the first moiré harmonic model with the zero-twist constraints imposed (d), and the reduced first moiré harmonic model with the zero-twist constraints imposed (e). Within each panel, we plot the dispersion of the (approximately or exactly) degenerate group of bands ($E_{\mathbf{k}}$) and their non-abelian Berry curvature ($\mathcal{F}_{\mathbf{k}}$) throughout the first moiré BZ (black hexagon). Additionally, we plot the real space CDD in layer $l = \pm$ within the moiré unit cell (dashed hexagon).

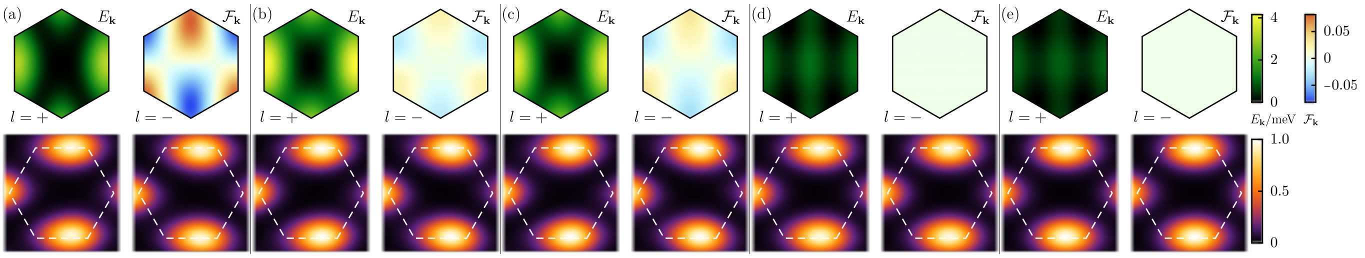

FIG. S75. The first group of conduction bands of twisted AA-stacked bilayer ZrS_2 at $\theta = 3.89°$. We consider the full continuum model (a), the full first moiré harmonic model (b), the reduced first moiré harmonic model (c), the first moiré harmonic model with the zero-twist constraints imposed (d), and the reduced first moiré harmonic model with the zero-twist constraints imposed (e). Within each panel, we plot the dispersion of the (approximately or exactly) degenerate group of bands ($E_{\mathbf{k}}$) and their non-abelian Berry curvature ($\mathcal{F}_{\mathbf{k}}$) throughout the first moiré BZ (black hexagon). Additionally, we plot the real space CDD in layer $l = \pm$ within the moiré unit cell (dashed hexagon).

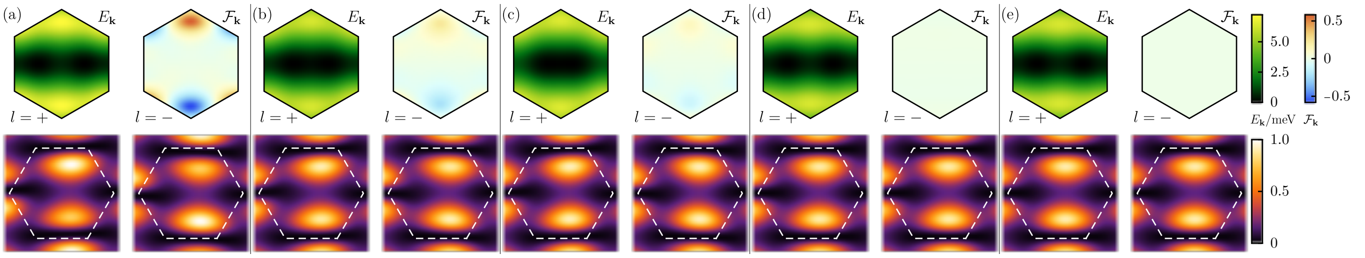

FIG. S76. The second group of conduction bands of twisted AA-stacked bilayer ZrS_2 at $\theta = 3.89°$. We consider the full continuum model (a), the full first moiré harmonic model (b), the reduced first moiré harmonic model (c), the first moiré harmonic model with the zero-twist constraints imposed (d), and the reduced first moiré harmonic model with the zero-twist constraints imposed (e). Within each panel, we plot the dispersion of the (approximately or exactly) degenerate group of bands ($E_{\mathbf{k}}$) and their non-abelian Berry curvature ($\mathcal{F}_{\mathbf{k}}$) throughout the first moiré BZ (black hexagon). Additionally, we plot the real space CDD in layer $l = \pm$ within the moiré unit cell (dashed hexagon).

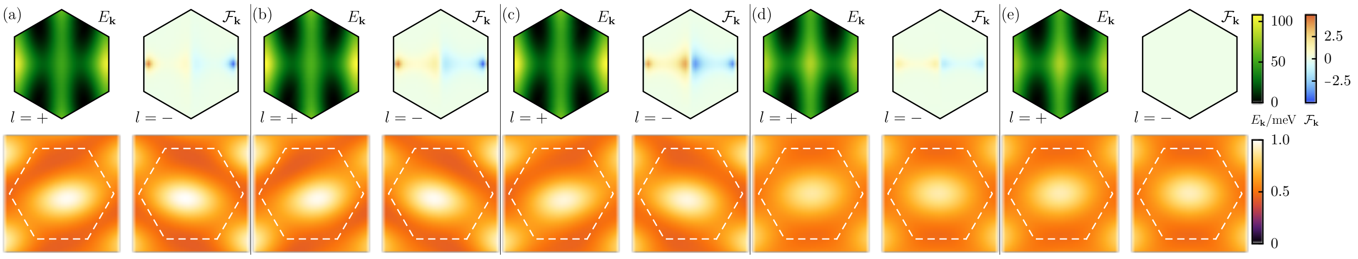

FIG. S77. The first group of conduction bands of twisted AB-stacked bilayer ZrS_2 at $\theta = 9.43°$. We consider the full continuum model (a), the full first moiré harmonic model (b), the reduced first moiré harmonic model (c), the first moiré harmonic model with the zero-twist constraints imposed (d), and the reduced first moiré harmonic model with the zero-twist constraints imposed (e). Within each panel, we plot the dispersion of the (approximately or exactly) degenerate group of bands ($E_{\mathbf{k}}$) and their non-abelian Berry curvature ($\mathcal{F}_{\mathbf{k}}$) throughout the first moiré BZ (black hexagon). Additionally, we plot the real space CDD in layer $l = \pm$ within the moiré unit cell (dashed hexagon).

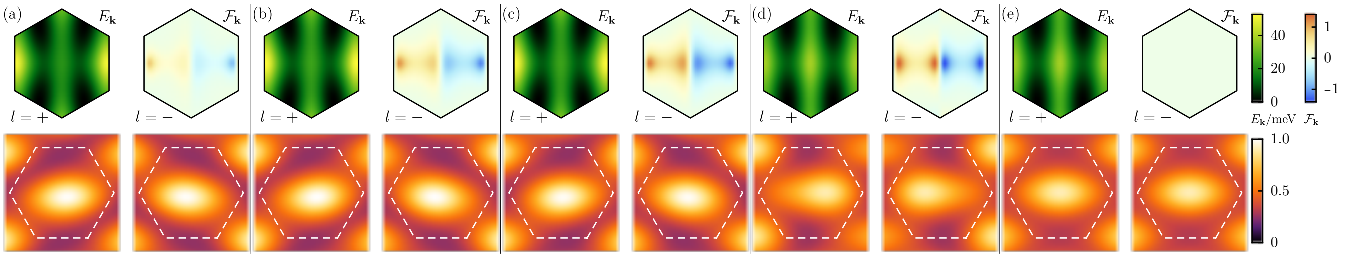

FIG. S78. The first group of conduction bands of twisted AB-stacked bilayer ZrS_2 at $\theta = 7.34°$. We consider the full continuum model (a), the full first moiré harmonic model (b), the reduced first moiré harmonic model (c), the first moiré harmonic model with the zero-twist constraints imposed (d), and the reduced first moiré harmonic model with the zero-twist constraints imposed (e). Within each panel, we plot the dispersion of the (approximately or exactly) degenerate group of bands ($E_{\mathbf{k}}$) and their non-abelian Berry curvature ($\mathcal{F}_{\mathbf{k}}$) throughout the first moiré BZ (black hexagon). Additionally, we plot the real space CDD in layer $l = \pm$ within the moiré unit cell (dashed hexagon).

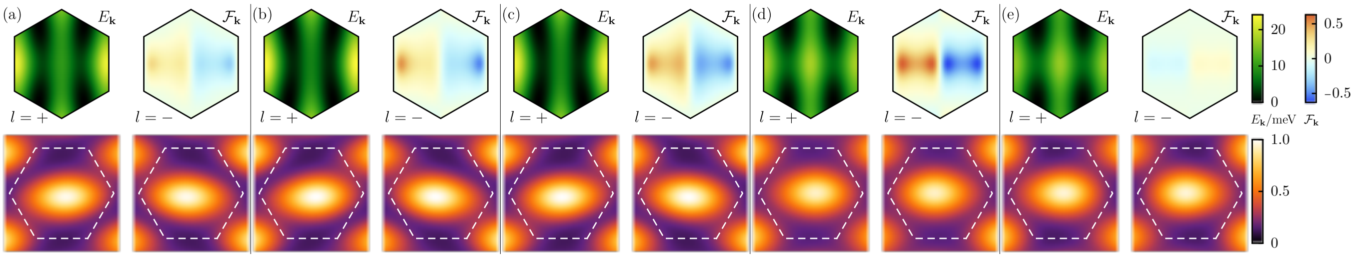

FIG. S79. The first group of conduction bands of twisted AB-stacked bilayer ZrS_2 at $\theta = 6.01°$. We consider the full continuum model (a), the full first moiré harmonic model (b), the reduced first moiré harmonic model (c), the first moiré harmonic model with the zero-twist constraints imposed (d), and the reduced first moiré harmonic model with the zero-twist constraints imposed (e). Within each panel, we plot the dispersion of the (approximately or exactly) degenerate group of bands ($E_{\mathbf{k}}$) and their non-abelian Berry curvature ($\mathcal{F}_{\mathbf{k}}$) throughout the first moiré BZ (black hexagon). Additionally, we plot the real space CDD in layer $l = \pm$ within the moiré unit cell (dashed hexagon).

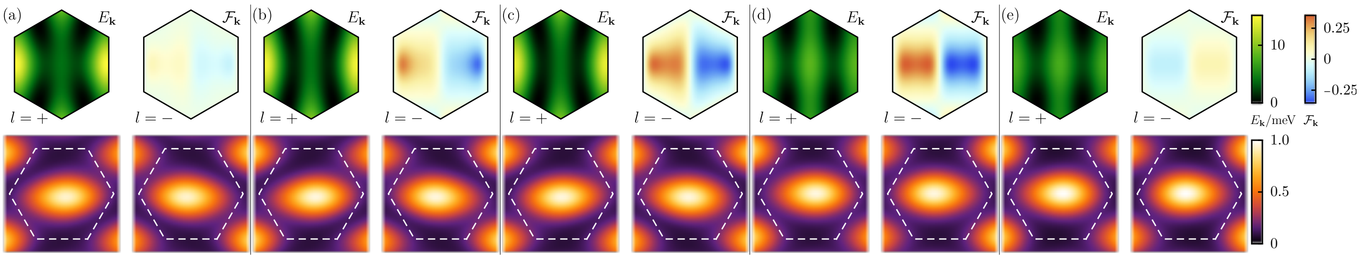

FIG. S80. The first group of conduction bands of twisted AB-stacked bilayer ZrS_2 at $\theta = 5.09°$. We consider the full continuum model (a), the full first moiré harmonic model (b), the reduced first moiré harmonic model (c), the first moiré harmonic model with the zero-twist constraints imposed (d), and the reduced first moiré harmonic model with the zero-twist constraints imposed (e). Within each panel, we plot the dispersion of the (approximately or exactly) degenerate group of bands ($E_{\mathbf{k}}$) and their non-abelian Berry curvature ($\mathcal{F}_{\mathbf{k}}$) throughout the first moiré BZ (black hexagon). Additionally, we plot the real space CDD in layer $l = \pm$ within the moiré unit cell (dashed hexagon).

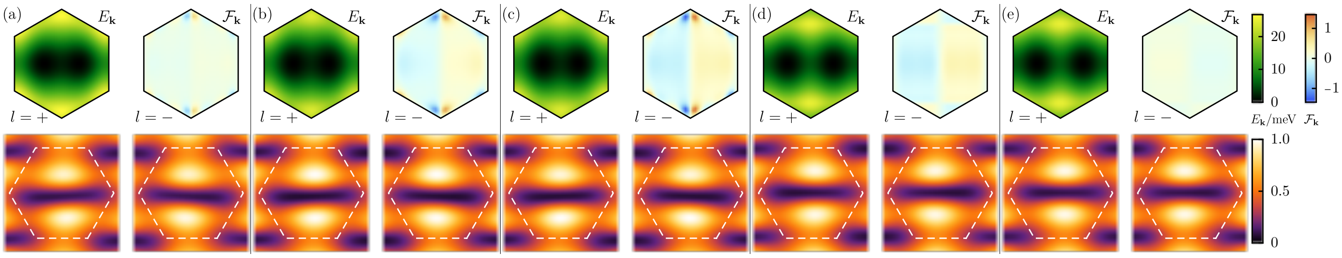

FIG. S81. The second group of conduction bands of twisted AB-stacked bilayer ZrS_2 at $\theta = 5.09°$. We consider the full continuum model (a), the full first moiré harmonic model (b), the reduced first moiré harmonic model (c), the first moiré harmonic model with the zero-twist constraints imposed (d), and the reduced first moiré harmonic model with the zero-twist constraints imposed (e). Within each panel, we plot the dispersion of the (approximately or exactly) degenerate group of bands ($E_{\mathbf{k}}$) and their non-abelian Berry curvature ($\mathcal{F}_{\mathbf{k}}$) throughout the first moiré BZ (black hexagon). Additionally, we plot the real space CDD in layer $l = \pm$ within the moiré unit cell (dashed hexagon).

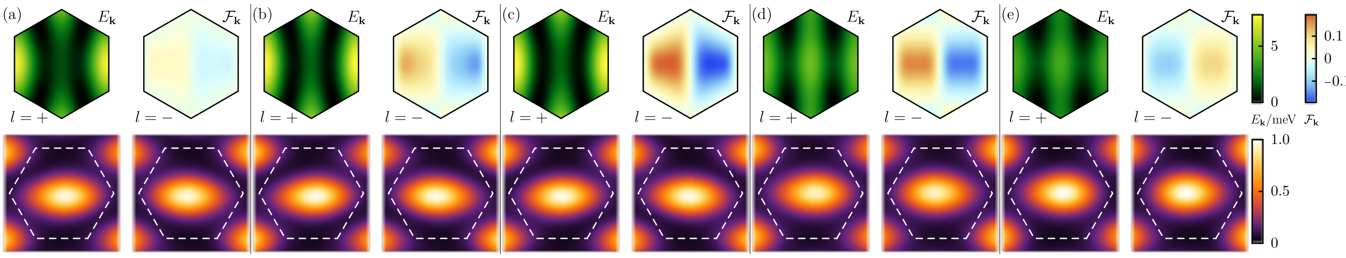

FIG. S82. The first group of conduction bands of twisted AB-stacked bilayer ZrS_2 at $\theta = 4.41°$. We consider the full continuum model (a), the full first moiré harmonic model (b), the reduced first moiré harmonic model (c), the first moiré harmonic model with the zero-twist constraints imposed (d), and the reduced first moiré harmonic model with the zero-twist constraints imposed (e). Within each panel, we plot the dispersion of the (approximately or exactly) degenerate group of bands ($E_{\mathbf{k}}$) and their non-abelian Berry curvature ($\mathcal{F}_{\mathbf{k}}$) throughout the first moiré BZ (black hexagon). Additionally, we plot the real space CDD in layer $l = \pm$ within the moiré unit cell (dashed hexagon).

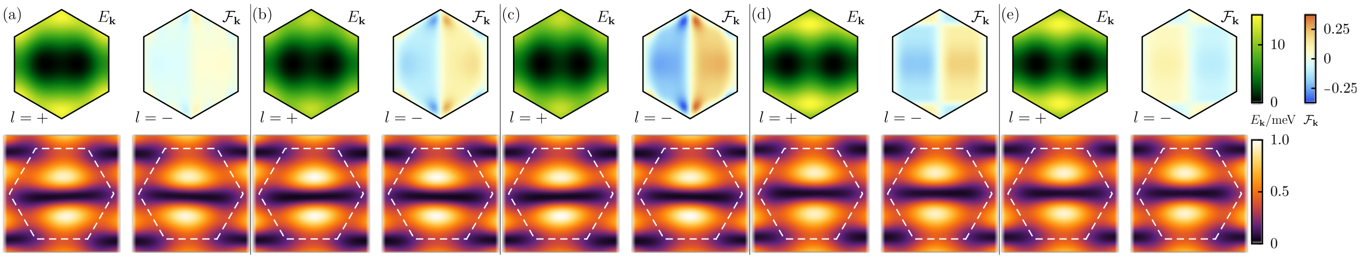

FIG. S83. The second group of conduction bands of twisted AB-stacked bilayer ZrS_2 at $\theta = 4.41°$. We consider the full continuum model (a), the full first moiré harmonic model (b), the reduced first moiré harmonic model (c), the first moiré harmonic model with the zero-twist constraints imposed (d), and the reduced first moiré harmonic model with the zero-twist constraints imposed (e). Within each panel, we plot the dispersion of the (approximately or exactly) degenerate group of bands ($E_{\mathbf{k}}$) and their non-abelian Berry curvature ($\mathcal{F}_{\mathbf{k}}$) throughout the first moiré BZ (black hexagon). Additionally, we plot the real space CDD in layer $l = \pm$ within the moiré unit cell (dashed hexagon).

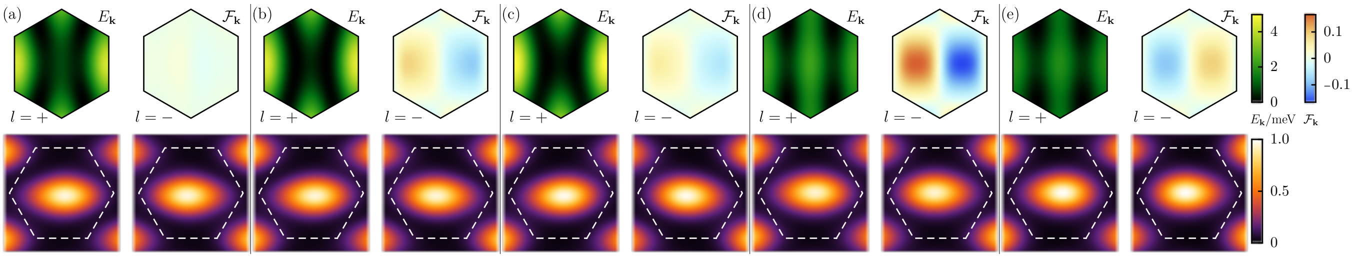

FIG. S84. The first group of conduction bands of twisted AB-stacked bilayer ZrS_2 at $\theta = 3.89°$. We consider the full continuum model (a), the full first moiré harmonic model (b), the reduced first moiré harmonic model (c), the first moiré harmonic model with the zero-twist constraints imposed (d), and the reduced first moiré harmonic model with the zero-twist constraints imposed (e). Within each panel, we plot the dispersion of the (approximately or exactly) degenerate group of bands ($E_{\mathbf{k}}$) and their non-abelian Berry curvature ($\mathcal{F}_{\mathbf{k}}$) throughout the first moiré BZ (black hexagon). Additionally, we plot the real space CDD in layer $l = \pm$ within the moiré unit cell (dashed hexagon).

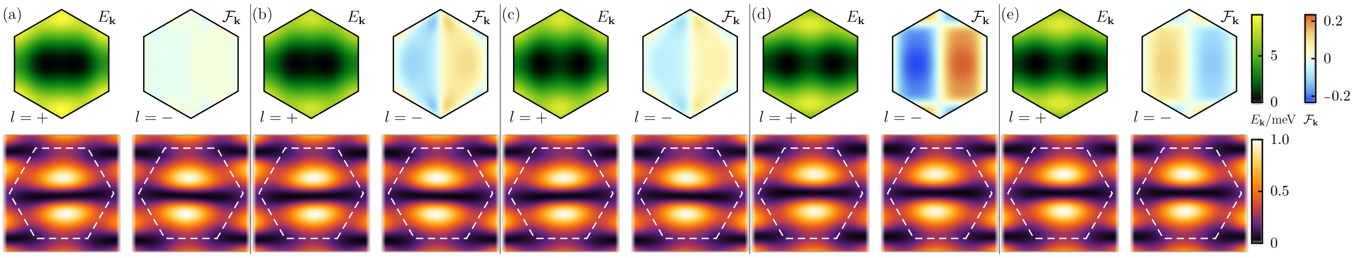

FIG. S85. The second group of conduction bands of twisted AB-stacked bilayer ZrS_2 at $\theta = 3.89°$. We consider the full continuum model (a), the full first moiré harmonic model (b), the reduced first moiré harmonic model (c), the first moiré harmonic model with the zero-twist constraints imposed (d), and the reduced first moiré harmonic model with the zero-twist constraints imposed (e). Within each panel, we plot the dispersion of the (approximately or exactly) degenerate group of bands ($E_{\mathbf{k}}$) and their non-abelian Berry curvature ($\mathcal{F}_{\mathbf{k}}$) throughout the first moiré BZ (black hexagon). Additionally, we plot the real space CDD in layer $l = \pm$ within the moiré unit cell (dashed hexagon).

3. *Wilson loops of the first gapped conduction bands along* $\mathbf{b}_{M_1}$

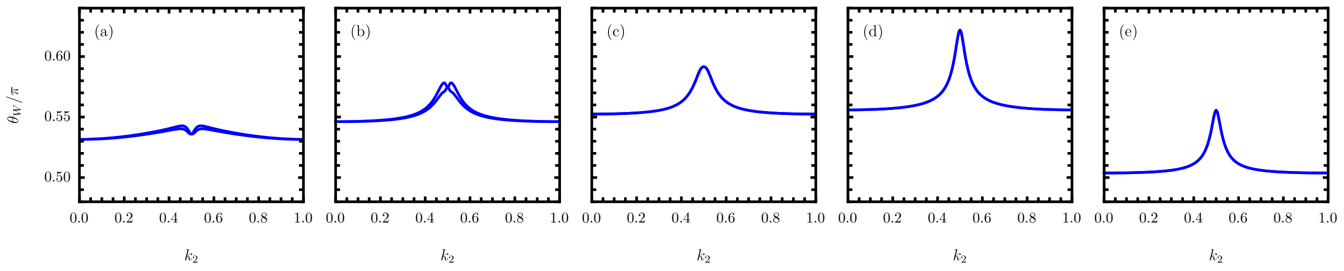

FIG. S86. Wilson loops for the first set of conduction bands of twisted AA-stacked bilayer $SnSe_2$ at $\theta = 9.43°$. The Wilson loop is computed along $\mathbf{b}_{M_1}$ We consider the full continuum model (a), the full first moiré harmonic model (b), the reduced first moiré harmonic model (c), the first moiré harmonic model with the zero-twist constraints imposed (d), and the reduced first moiré harmonic model with the zero-twist constraints imposed (e).

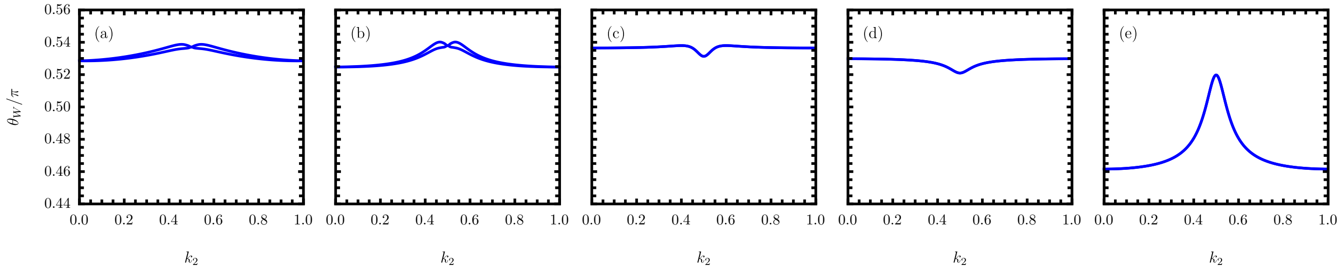

FIG. S87. Wilson loops for the first set of conduction bands of twisted AA-stacked bilayer $SnSe_2$ at $\theta = 7.34°$. The Wilson loop is computed along $\mathbf{b}_{M_1}$ We consider the full continuum model (a), the full first moiré harmonic model (b), the reduced first moiré harmonic model (c), the first moiré harmonic model with the zero-twist constraints imposed (d), and the reduced first moiré harmonic model with the zero-twist constraints imposed (e).

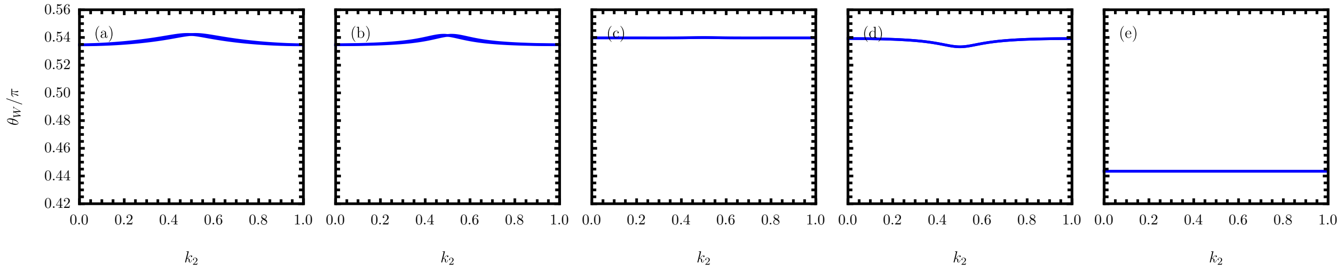

FIG. S88. Wilson loops for the first set of conduction bands of twisted AA-stacked bilayer $SnSe_2$ at $\theta = 6.01°$. The Wilson loop is computed along $\mathbf{b}_{M_1}$ We consider the full continuum model (a), the full first moiré harmonic model (b), the reduced first moiré harmonic model (c), the first moiré harmonic model with the zero-twist constraints imposed (d), and the reduced first moiré harmonic model with the zero-twist constraints imposed (e).

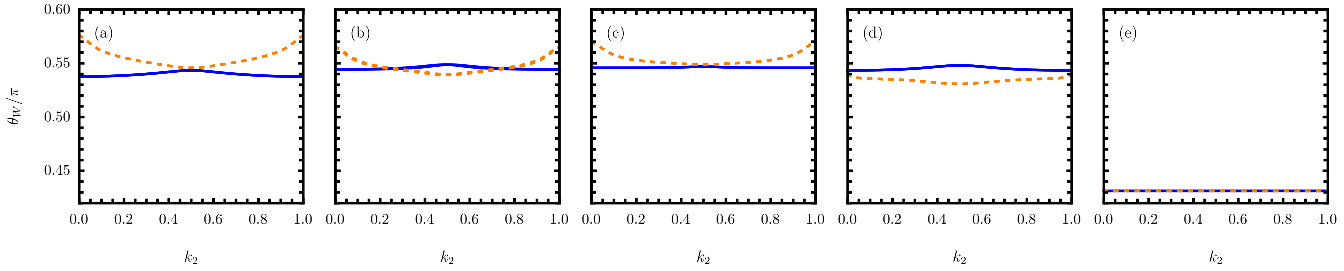

FIG. S89. Wilson loops for the first two sets of conduction bands of twisted AA-stacked bilayer $SnSe_2$ at $\theta = 5.09°$. The Wilson loop is computed along $\mathbf{b}_{M_1}$ We consider the full continuum model (a), the full first moiré harmonic model (b), the reduced first moiré harmonic model (c), the first moiré harmonic model with the zero-twist constraints imposed (d), and the reduced first moiré harmonic model with the zero-twist constraints imposed (e). The blue (dashed orange) lines correspond to the first (second) set of conduction bands.

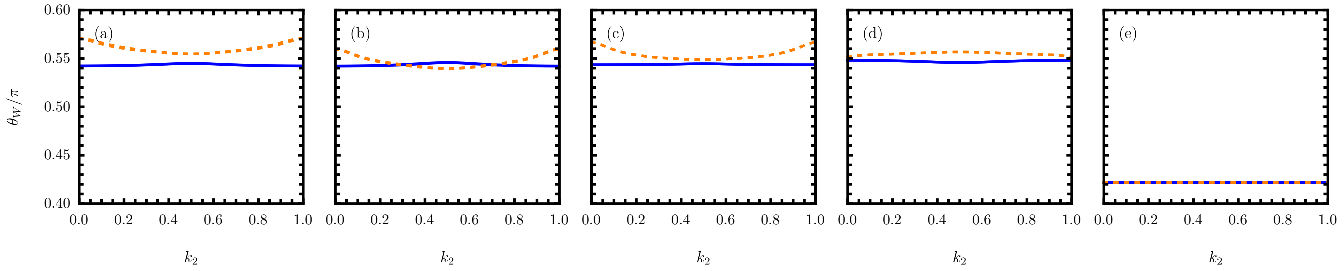

FIG. S90. Wilson loops for the first two sets of conduction bands of twisted AA-stacked bilayer $SnSe_2$ at $\theta = 4.41°$. The Wilson loop is computed along $\mathbf{b}_{M_1}$ We consider the full continuum model (a), the full first moiré harmonic model (b), the reduced first moiré harmonic model (c), the first moiré harmonic model with the zero-twist constraints imposed (d), and the reduced first moiré harmonic model with the zero-twist constraints imposed (e). The blue (dashed orange) lines correspond to the first (second) set of conduction bands.

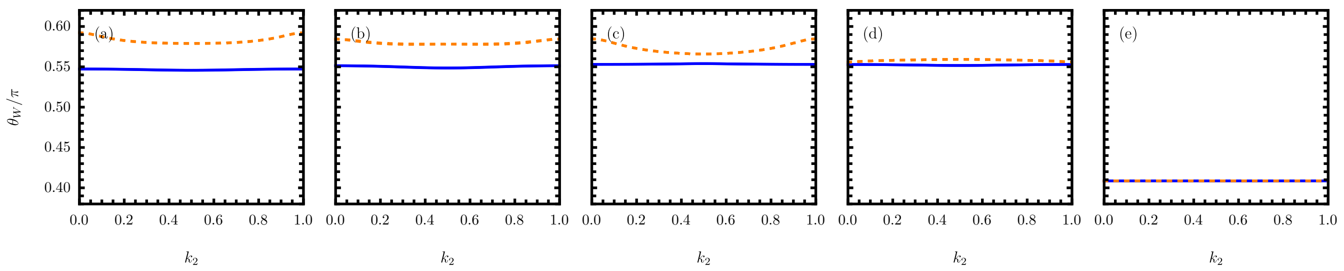

FIG. S91. Wilson loops for the first two sets of conduction bands of twisted AA-stacked bilayer $SnSe_2$ at $\theta = 3.89°$. The Wilson loop is computed along $\mathbf{b}_{M_1}$ We consider the full continuum model (a), the full first moiré harmonic model (b), the reduced first moiré harmonic model (c), the first moiré harmonic model with the zero-twist constraints imposed (d), and the reduced first moiré harmonic model with the zero-twist constraints imposed (e). The blue (dashed orange) lines correspond to the first (second) set of conduction bands.

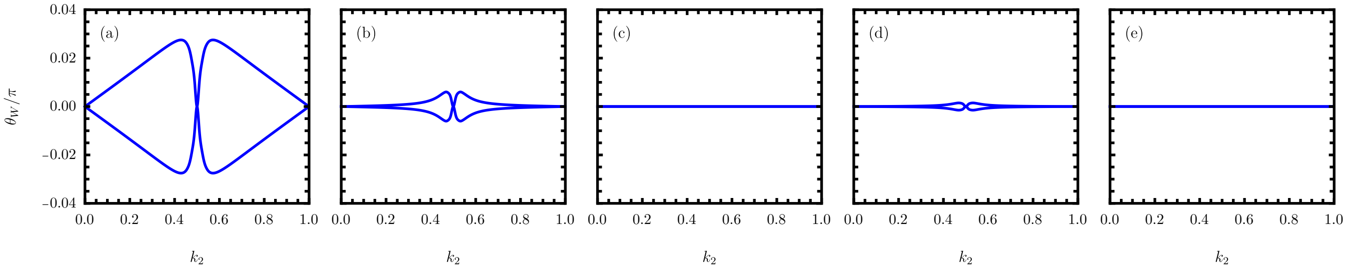

FIG. S92. Wilson loops for the first set of conduction bands of twisted AB-stacked bilayer $SnSe_2$ at $\theta = 9.43°$. The Wilson loop is computed along $\mathbf{b}_{M_1}$ We consider the full continuum model (a), the full first moiré harmonic model (b), the reduced first moiré harmonic model (c), the first moiré harmonic model with the zero-twist constraints imposed (d), and the reduced first moiré harmonic model with the zero-twist constraints imposed (e).

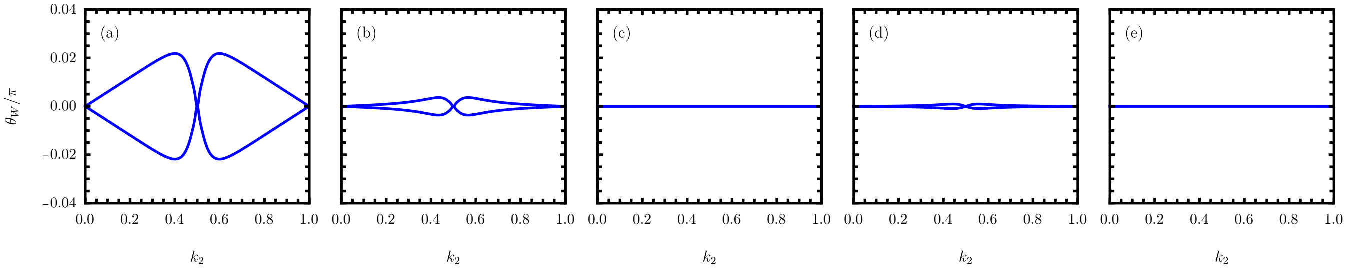

FIG. S93. Wilson loops for the first set of conduction bands of twisted AB-stacked bilayer $SnSe_2$ at $\theta = 7.34°$. The Wilson loop is computed along $\mathbf{b}_{M_1}$ We consider the full continuum model (a), the full first moiré harmonic model (b), the reduced first moiré harmonic model (c), the first moiré harmonic model with the zero-twist constraints imposed (d), and the reduced first moiré harmonic model with the zero-twist constraints imposed (e).

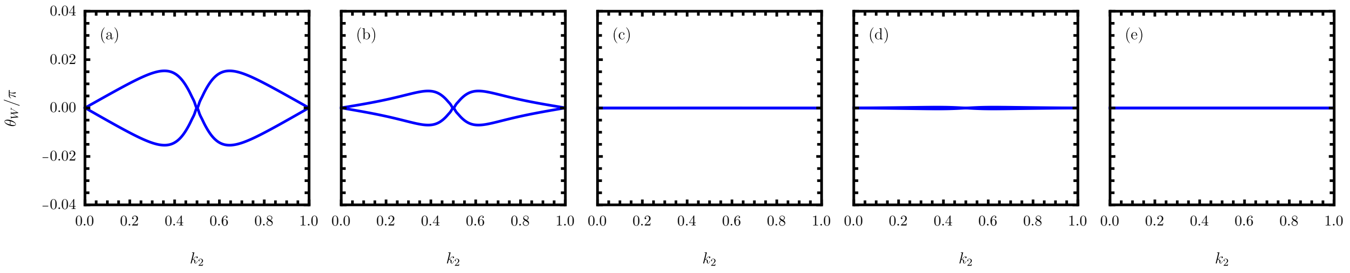

FIG. S94. Wilson loops for the first set of conduction bands of twisted AB-stacked bilayer $SnSe_2$ at $\theta = 6.01°$. The Wilson loop is computed along $\mathbf{b}_{M_1}$ We consider the full continuum model (a), the full first moiré harmonic model (b), the reduced first moiré harmonic model (c), the first moiré harmonic model with the zero-twist constraints imposed (d), and the reduced first moiré harmonic model with the zero-twist constraints imposed (e).

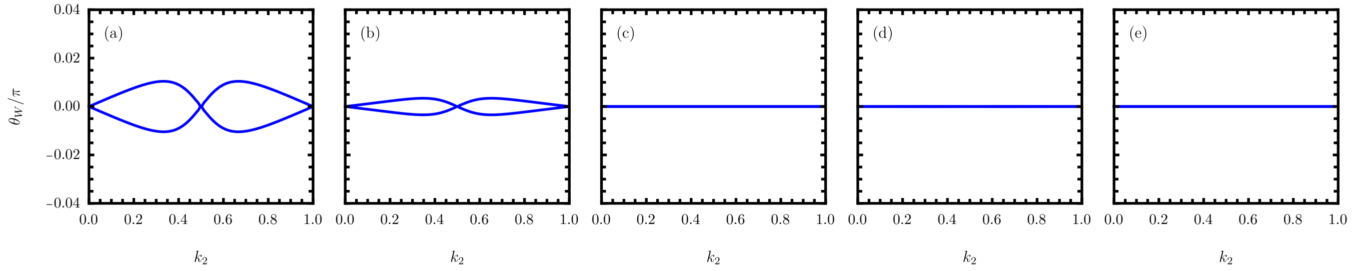

FIG. S95. Wilson loops for the first set of conduction bands of twisted AB-stacked bilayer $SnSe_2$ at $\theta = 5.09°$. The Wilson loop is computed along $\mathbf{b}_{M_1}$ We consider the full continuum model (a), the full first moiré harmonic model (b), the reduced first moiré harmonic model (c), the first moiré harmonic model with the zero-twist constraints imposed (d), and the reduced first moiré harmonic model with the zero-twist constraints imposed (e).

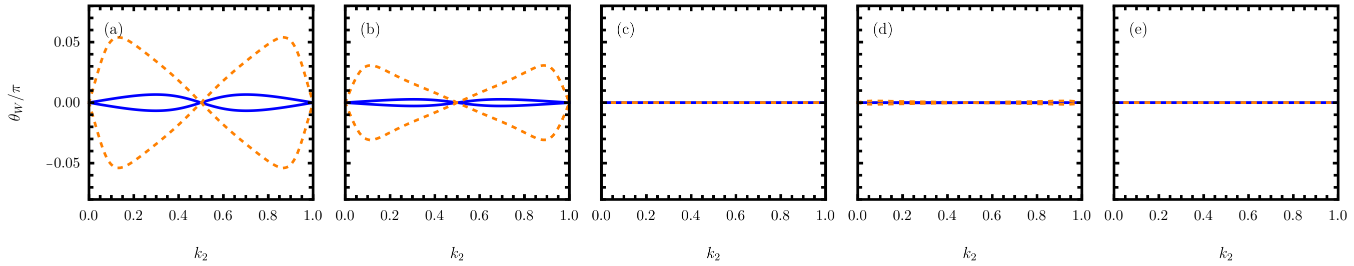

FIG. S96. Wilson loops for the first two sets of conduction bands of twisted AB-stacked bilayer $SnSe_2$ at $\theta = 4.41°$. The Wilson loop is computed along $\mathbf{b}_{M_1}$ We consider the full continuum model (a), the full first moiré harmonic model (b), the reduced first moiré harmonic model (c), the first moiré harmonic model with the zero-twist constraints imposed (d), and the reduced first moiré harmonic model with the zero-twist constraints imposed (e). The blue (dashed orange) lines correspond to the first (second) set of conduction bands.

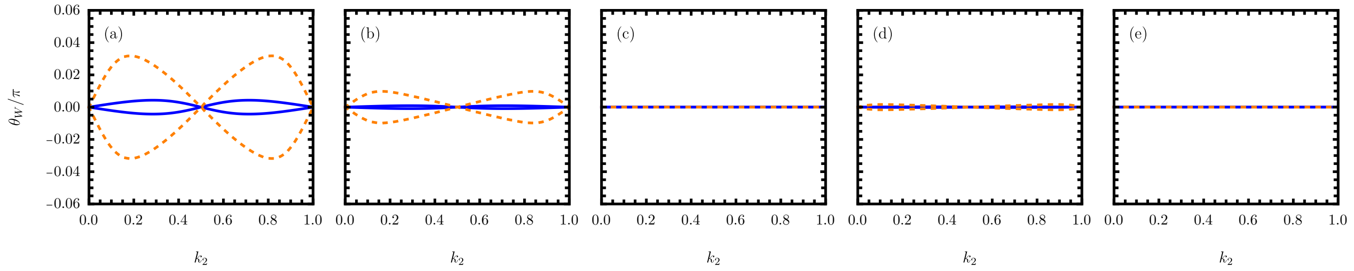

FIG. S97. Wilson loops for the first two sets of conduction bands of twisted AB-stacked bilayer $SnSe_2$ at $\theta = 3.89°$. The Wilson loop is computed along $\mathbf{b}_{M_1}$ We consider the full continuum model (a), the full first moiré harmonic model (b), the reduced first moiré harmonic model (c), the first moiré harmonic model with the zero-twist constraints imposed (d), and the reduced first moiré harmonic model with the zero-twist constraints imposed (e). The blue (dashed orange) lines correspond to the first (second) set of conduction bands.

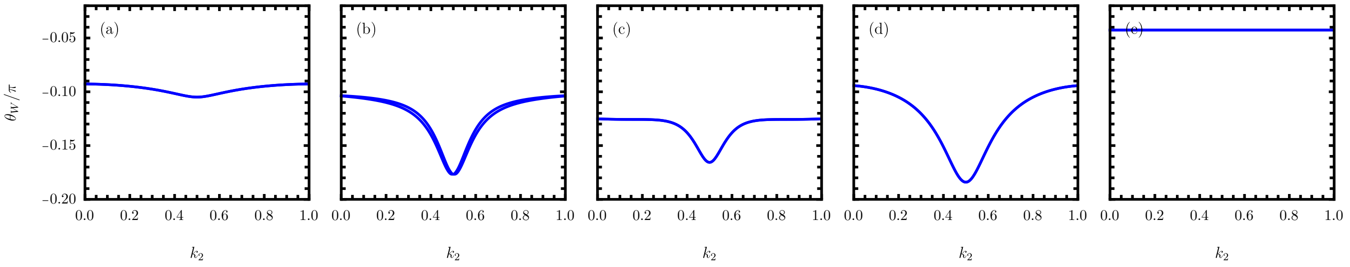

FIG. S98. Wilson loops for the first set of conduction bands of twisted AA-stacked bilayer ZrS_2 at $\theta = 9.43°$. The Wilson loop is computed along $\mathbf{b}_{M_1}$ We consider the full continuum model (a), the full first moiré harmonic model (b), the reduced first moiré harmonic model (c), the first moiré harmonic model with the zero-twist constraints imposed (d), and the reduced first moiré harmonic model with the zero-twist constraints imposed (e).

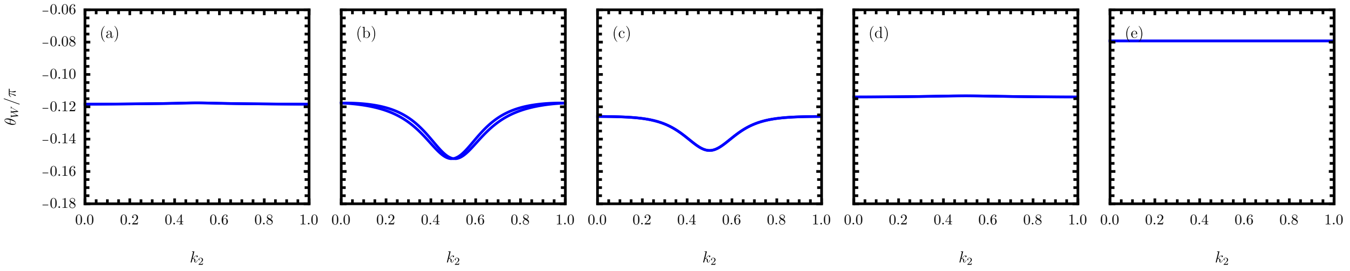

FIG. S99. Wilson loops for the first set of conduction bands of twisted AA-stacked bilayer ZrS_2 at $\theta = 7.34°$. The Wilson loop is computed along $\mathbf{b}_{M_1}$ We consider the full continuum model (a), the full first moiré harmonic model (b), the reduced first moiré harmonic model (c), the first moiré harmonic model with the zero-twist constraints imposed (d), and the reduced first moiré harmonic model with the zero-twist constraints imposed (e).

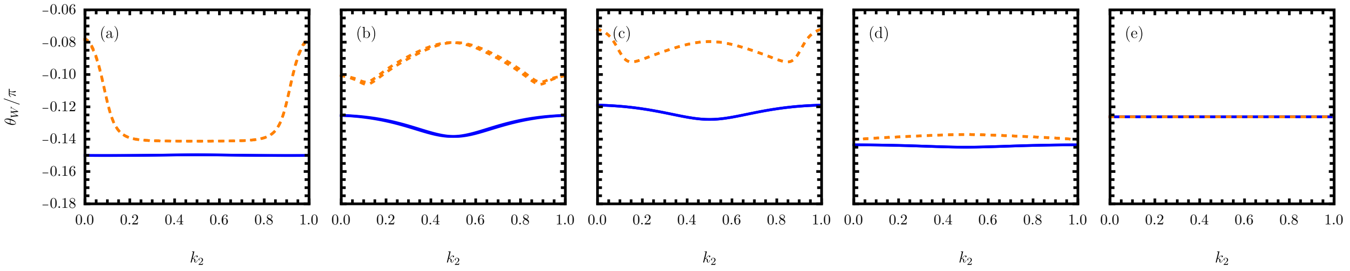

FIG. S100. Wilson loops for the first two sets of conduction bands of twisted AA-stacked bilayer ZrS_2 at $\theta = 6.01°$. The Wilson loop is computed along $\mathbf{b}_{M_1}$ We consider the full continuum model (a), the full first moiré harmonic model (b), the reduced first moiré harmonic model (c), the first moiré harmonic model with the zero-twist constraints imposed (d), and the reduced first moiré harmonic model with the zero-twist constraints imposed (e). The blue (dashed orange) lines correspond to the first (second) set of conduction bands.

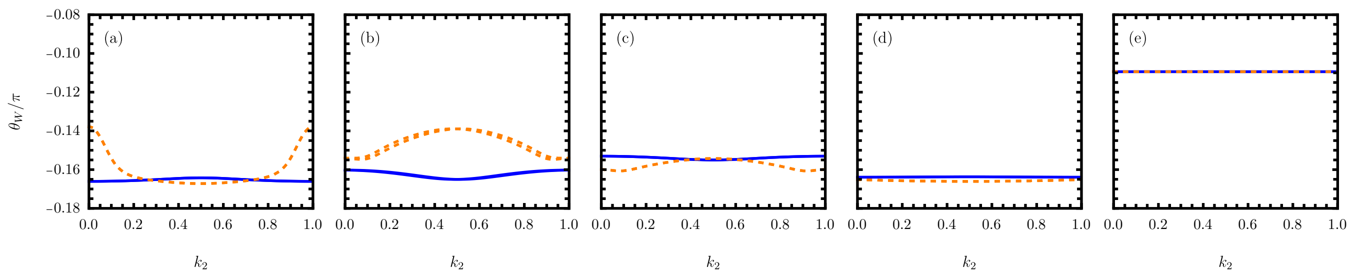

FIG. S101. Wilson loops for the first two sets of conduction bands of twisted AA-stacked bilayer ZrS_2 at $\theta = 5.09°$. The Wilson loop is computed along $\mathbf{b}_{M_1}$ We consider the full continuum model (a), the full first moiré harmonic model (b), the reduced first moiré harmonic model (c), the first moiré harmonic model with the zero-twist constraints imposed (d), and the reduced first moiré harmonic model with the zero-twist constraints imposed (e). The blue (dashed orange) lines correspond to the first (second) set of conduction bands.

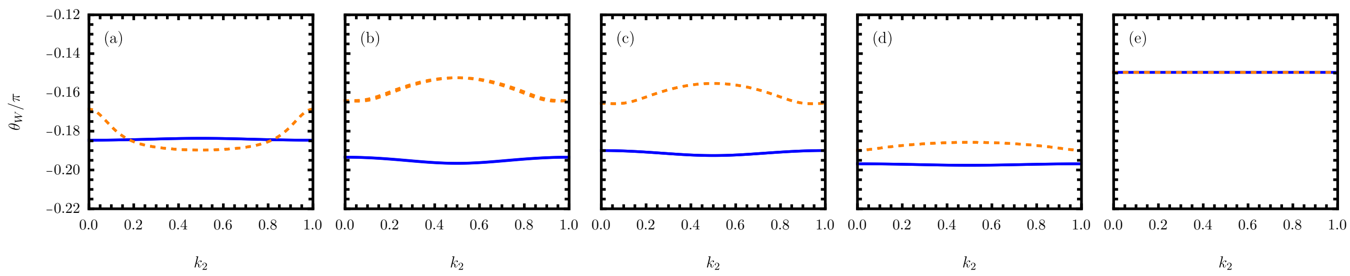

FIG. S102. Wilson loops for the first two sets of conduction bands of twisted AA-stacked bilayer ZrS_2 at $\theta = 4.41°$. The Wilson loop is computed along $\mathbf{b}_{M_1}$ We consider the full continuum model (a), the full first moiré harmonic model (b), the reduced first moiré harmonic model (c), the first moiré harmonic model with the zero-twist constraints imposed (d), and the reduced first moiré harmonic model with the zero-twist constraints imposed (e). The blue (dashed orange) lines correspond to the first (second) set of conduction bands.

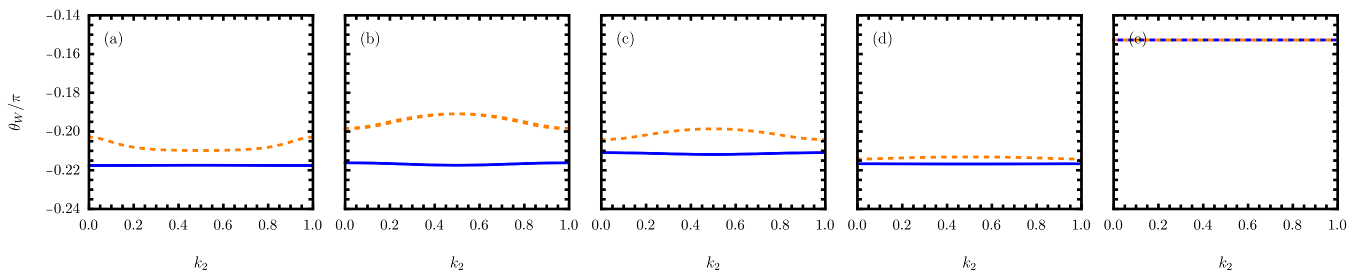

FIG. S103. Wilson loops for the first two sets of conduction bands of twisted AA-stacked bilayer ZrS_2 at $\theta = 3.89°$. The Wilson loop is computed along $\mathbf{b}_{M_1}$ We consider the full continuum model (a), the full first moiré harmonic model (b), the reduced first moiré harmonic model (c), the first moiré harmonic model with the zero-twist constraints imposed (d), and the reduced first moiré harmonic model with the zero-twist constraints imposed (e). The blue (dashed orange) lines correspond to the first (second) set of conduction bands.

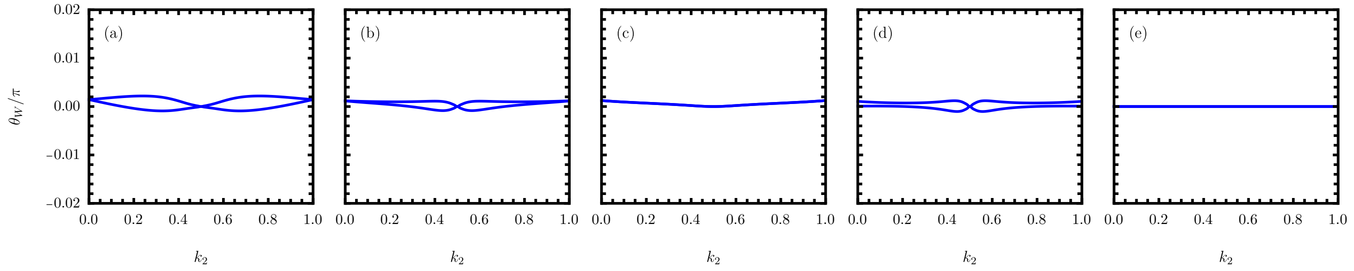

FIG. S104. Wilson loops for the first set of conduction bands of twisted AB-stacked bilayer ZrS_2 at $\theta = 9.43°$. The Wilson loop is computed along $\mathbf{b}_{M_1}$ We consider the full continuum model (a), the full first moiré harmonic model (b), the reduced first moiré harmonic model (c), the first moiré harmonic model with the zero-twist constraints imposed (d), and the reduced first moiré harmonic model with the zero-twist constraints imposed (e).

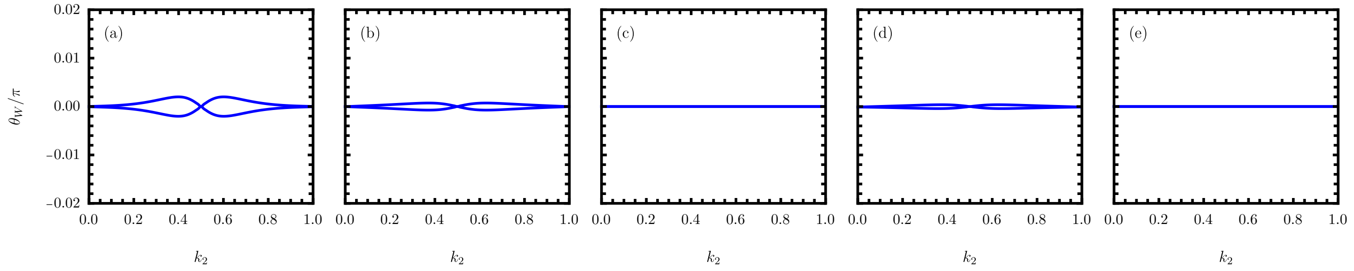

FIG. S105. Wilson loops for the first set of conduction bands of twisted AB-stacked bilayer ZrS_2 at $\theta = 7.34°$. The Wilson loop is computed along $\mathbf{b}_{M_1}$ We consider the full continuum model (a), the full first moiré harmonic model (b), the reduced first moiré harmonic model (c), the first moiré harmonic model with the zero-twist constraints imposed (d), and the reduced first moiré harmonic model with the zero-twist constraints imposed (e).

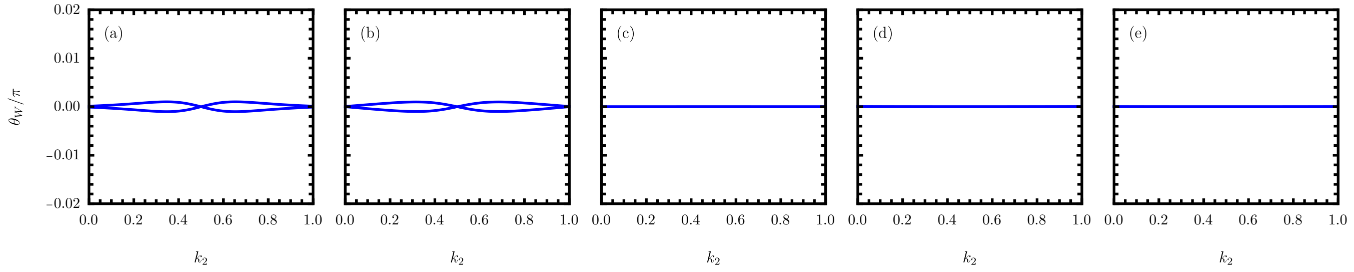

FIG. S106. Wilson loops for the first set of conduction bands of twisted AB-stacked bilayer ZrS_2 at $\theta = 6.01°$. The Wilson loop is computed along $\mathbf{b}_{M_1}$ We consider the full continuum model (a), the full first moiré harmonic model (b), the reduced first moiré harmonic model (c), the first moiré harmonic model with the zero-twist constraints imposed (d), and the reduced first moiré harmonic model with the zero-twist constraints imposed (e).

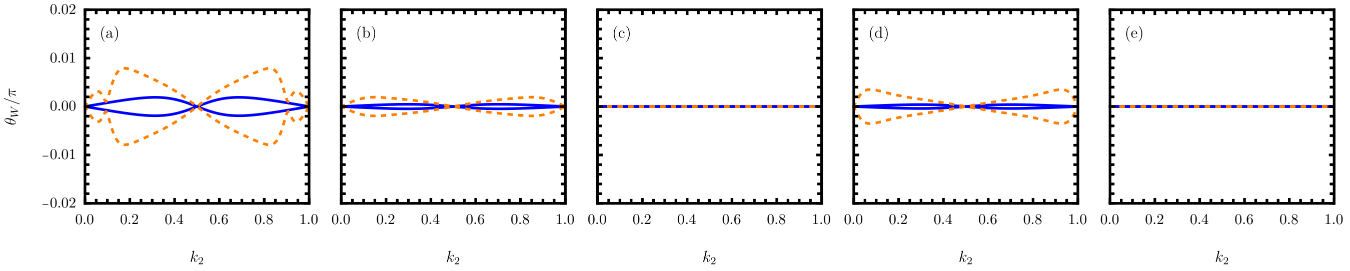

FIG. S107. Wilson loops for the first two sets of conduction bands of twisted AB-stacked bilayer ZrS_2 at $\theta = 5.09°$. The Wilson loop is computed along $\mathbf{b}_{M_1}$ We consider the full continuum model (a), the full first moiré harmonic model (b), the reduced first moiré harmonic model (c), the first moiré harmonic model with the zero-twist constraints imposed (d), and the reduced first moiré harmonic model with the zero-twist constraints imposed (e). The blue (dashed orange) lines correspond to the first (second) set of conduction bands.

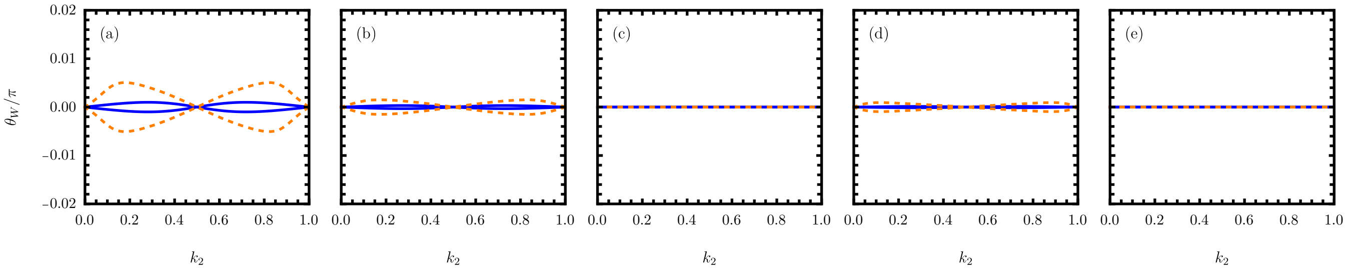

FIG. S108. Wilson loops for the first two sets of conduction bands of twisted AB-stacked bilayer ZrS_2 at $\theta = 4.41°$. The Wilson loop is computed along $\mathbf{b}_{M_1}$ We consider the full continuum model (a), the full first moiré harmonic model (b), the reduced first moiré harmonic model (c), the first moiré harmonic model with the zero-twist constraints imposed (d), and the reduced first moiré harmonic model with the zero-twist constraints imposed (e). The blue (dashed orange) lines correspond to the first (second) set of conduction bands.

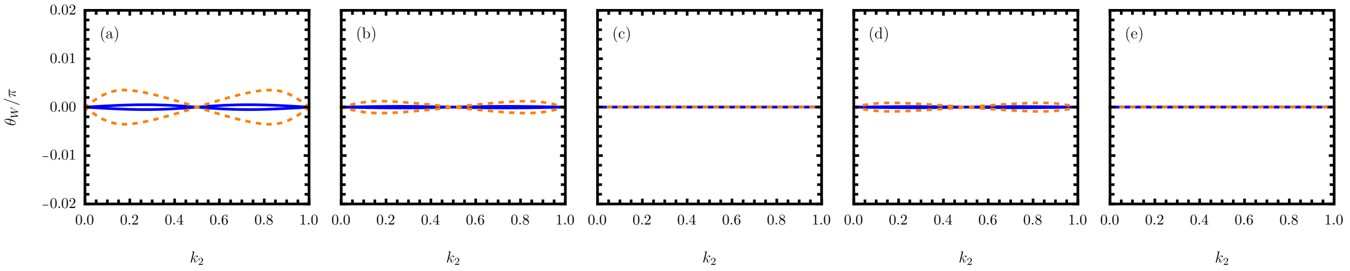

FIG. S109. Wilson loops for the first two sets of conduction bands of twisted AB-stacked bilayer ZrS_2 at $\theta = 3.89°$. The Wilson loop is computed along $\mathbf{b}_{M_1}$ We consider the full continuum model (a), the full first moiré harmonic model (b), the reduced first moiré harmonic model (c), the first moiré harmonic model with the zero-twist constraints imposed (d), and the reduced first moiré harmonic model with the zero-twist constraints imposed (e). The blue (dashed orange) lines correspond to the first (second) set of conduction bands.

4. *Wilson loops of the first gapped conduction bands along* $\mathbf{b}_{M_2}$

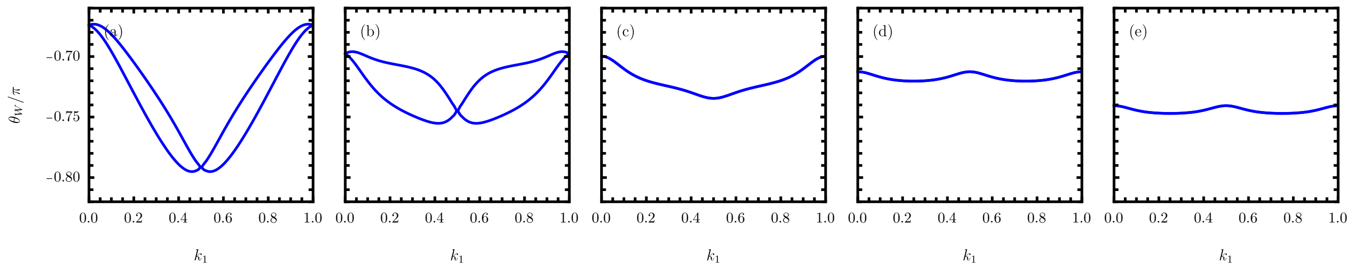

FIG. S110. Wilson loops for the first set of conduction bands of twisted AA-stacked bilayer $SnSe_2$ at $\theta = 9.43°$. The Wilson loop is computed along $\mathbf{b}_{M_2}$ We consider the full continuum model (a), the full first moiré harmonic model (b), the reduced first moiré harmonic model (c), the first moiré harmonic model with the zero-twist constraints imposed (d), and the reduced first moiré harmonic model with the zero-twist constraints imposed (e).

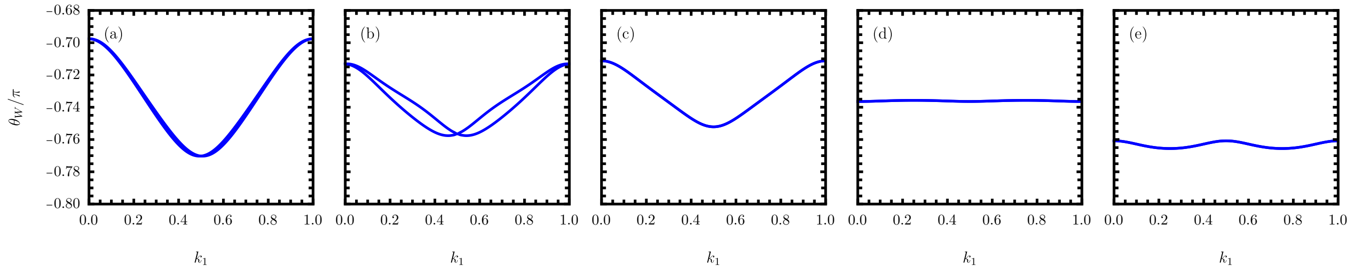

FIG. S111. Wilson loops for the first set of conduction bands of twisted AA-stacked bilayer $SnSe_2$ at $\theta = 7.34°$. The Wilson loop is computed along $\mathbf{b}_{M_2}$ We consider the full continuum model (a), the full first moiré harmonic model (b), the reduced first moiré harmonic model (c), the first moiré harmonic model with the zero-twist constraints imposed (d), and the reduced first moiré harmonic model with the zero-twist constraints imposed (e).

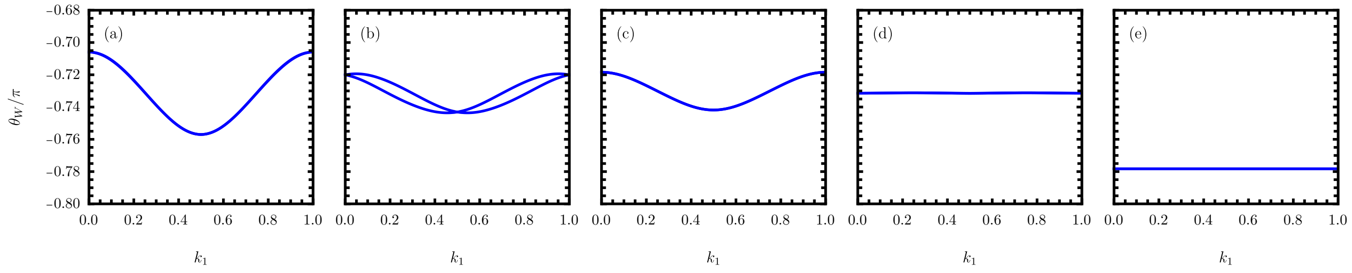

FIG. S112. Wilson loops for the first set of conduction bands of twisted AA-stacked bilayer $SnSe_2$ at $\theta = 6.01°$. The Wilson loop is computed along $\mathbf{b}_{M_2}$ We consider the full continuum model (a), the full first moiré harmonic model (b), the reduced first moiré harmonic model (c), the first moiré harmonic model with the zero-twist constraints imposed (d), and the reduced first moiré harmonic model with the zero-twist constraints imposed (e).

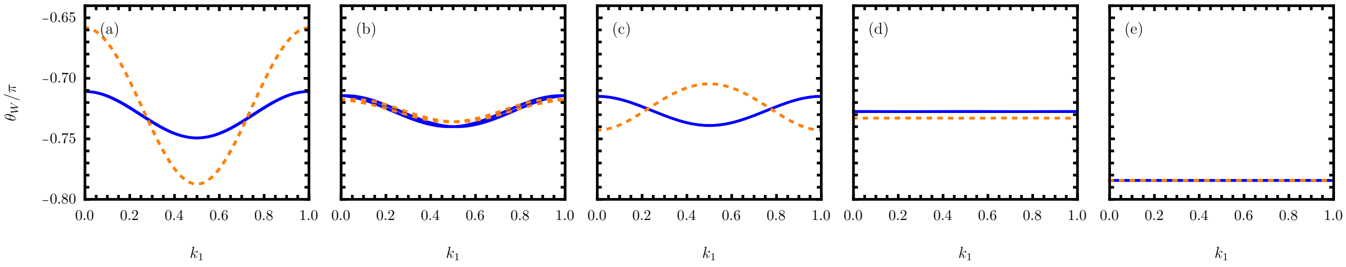

FIG. S113. Wilson loops for the first two sets of conduction bands of twisted AA-stacked bilayer $SnSe_2$ at $\theta = 5.09°$. The Wilson loop is computed along $\mathbf{b}_{M_2}$ We consider the full continuum model (a), the full first moiré harmonic model (b), the reduced first moiré harmonic model (c), the first moiré harmonic model with the zero-twist constraints imposed (d), and the reduced first moiré harmonic model with the zero-twist constraints imposed (e). The blue (dashed orange) lines correspond to the first (second) set of conduction bands.

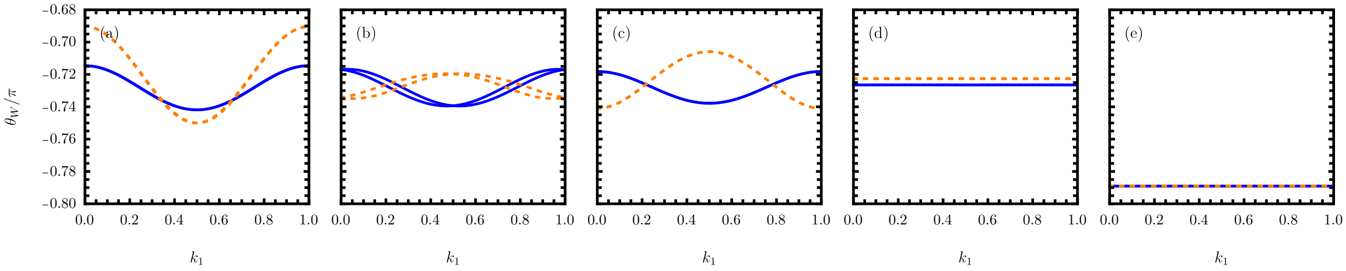

FIG. S114. Wilson loops for the first two sets of conduction bands of twisted AA-stacked bilayer $SnSe_2$ at $\theta = 4.41°$. The Wilson loop is computed along $\mathbf{b}_{M_2}$ We consider the full continuum model (a), the full first moiré harmonic model (b), the reduced first moiré harmonic model (c), the first moiré harmonic model with the zero-twist constraints imposed (d), and the reduced first moiré harmonic model with the zero-twist constraints imposed (e). The blue (dashed orange) lines correspond to the first (second) set of conduction bands.

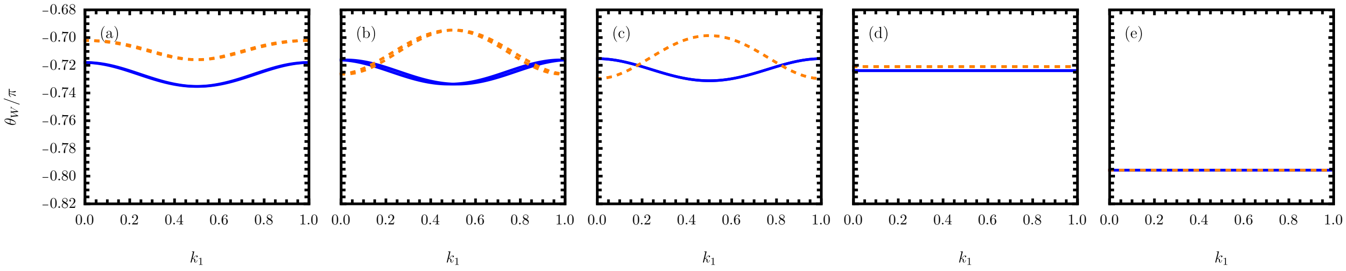

FIG. S115. Wilson loops for the first two sets of conduction bands of twisted AA-stacked bilayer $SnSe_2$ at $\theta = 3.89°$. The Wilson loop is computed along $\mathbf{b}_{M_2}$ We consider the full continuum model (a), the full first moiré harmonic model (b), the reduced first moiré harmonic model (c), the first moiré harmonic model with the zero-twist constraints imposed (d), and the reduced first moiré harmonic model with the zero-twist constraints imposed (e). The blue (dashed orange) lines correspond to the first (second) set of conduction bands.

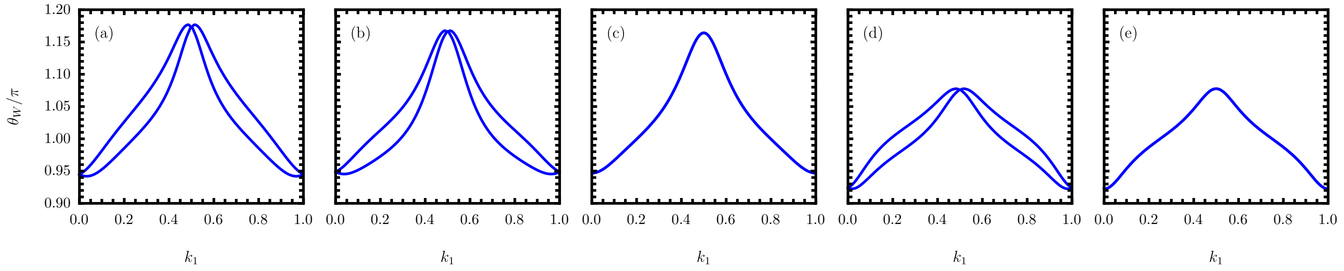

FIG. S116. Wilson loops for the first set of conduction bands of twisted AB-stacked bilayer $SnSe_2$ at $\theta = 9.43°$. The Wilson loop is computed along $\mathbf{b}_{M_2}$ We consider the full continuum model (a), the full first moiré harmonic model (b), the reduced first moiré harmonic model (c), the first moiré harmonic model with the zero-twist constraints imposed (d), and the reduced first moiré harmonic model with the zero-twist constraints imposed (e).

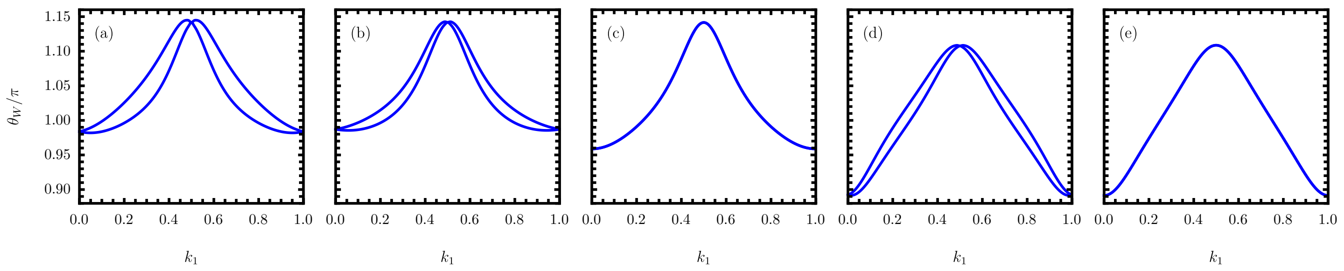

FIG. S117. Wilson loops for the first set of conduction bands of twisted AB-stacked bilayer $SnSe_2$ at $\theta = 7.34°$. The Wilson loop is computed along $\mathbf{b}_{M_2}$ We consider the full continuum model (a), the full first moiré harmonic model (b), the reduced first moiré harmonic model (c), the first moiré harmonic model with the zero-twist constraints imposed (d), and the reduced first moiré harmonic model with the zero-twist constraints imposed (e).

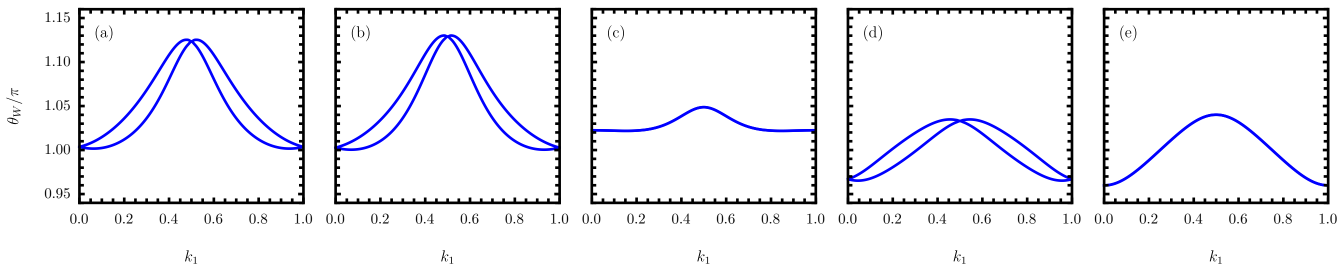

FIG. S118. Wilson loops for the first set of conduction bands of twisted AB-stacked bilayer $SnSe_2$ at $\theta = 6.01°$. The Wilson loop is computed along $\mathbf{b}_{M_2}$ We consider the full continuum model (a), the full first moiré harmonic model (b), the reduced first moiré harmonic model (c), the first moiré harmonic model with the zero-twist constraints imposed (d), and the reduced first moiré harmonic model with the zero-twist constraints imposed (e).

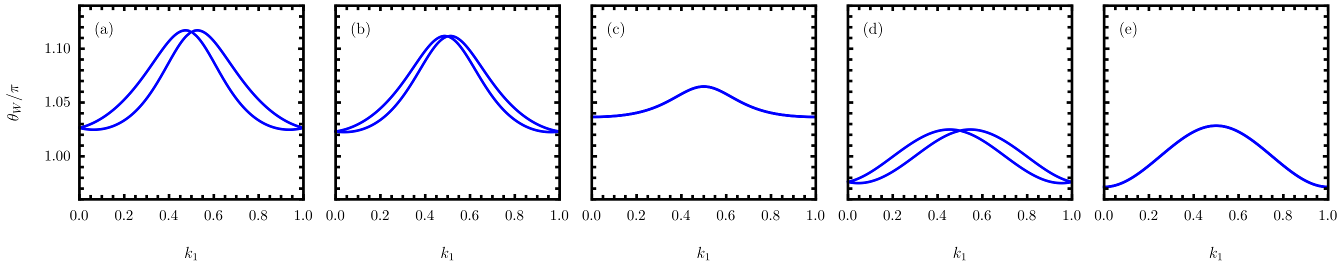

FIG. S119. Wilson loops for the first set of conduction bands of twisted AB-stacked bilayer $SnSe_2$ at $\theta = 5.09°$. The Wilson loop is computed along $\mathbf{b}_{M_2}$ We consider the full continuum model (a), the full first moiré harmonic model (b), the reduced first moiré harmonic model (c), the first moiré harmonic model with the zero-twist constraints imposed (d), and the reduced first moiré harmonic model with the zero-twist constraints imposed (e).

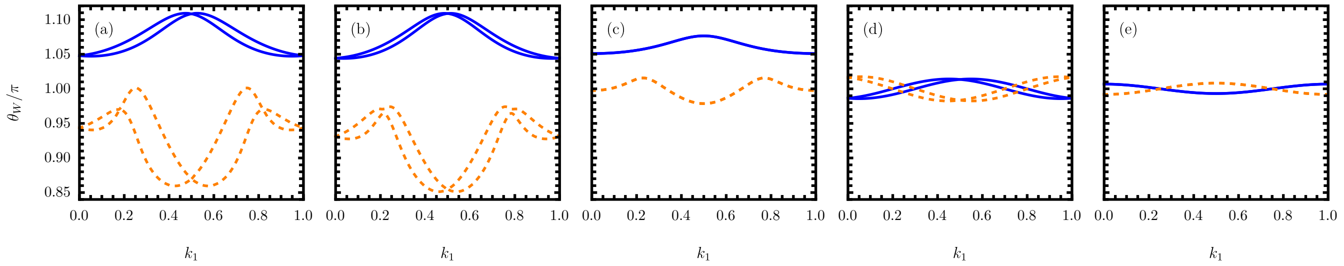

FIG. S120. Wilson loops for the first two sets of conduction bands of twisted AB-stacked bilayer $SnSe_2$ at $\theta = 4.41°$. The Wilson loop is computed along $\mathbf{b}_{M_2}$ We consider the full continuum model (a), the full first moiré harmonic model (b), the reduced first moiré harmonic model (c), the first moiré harmonic model with the zero-twist constraints imposed (d), and the reduced first moiré harmonic model with the zero-twist constraints imposed (e). The blue (dashed orange) lines correspond to the first (second) set of conduction bands.

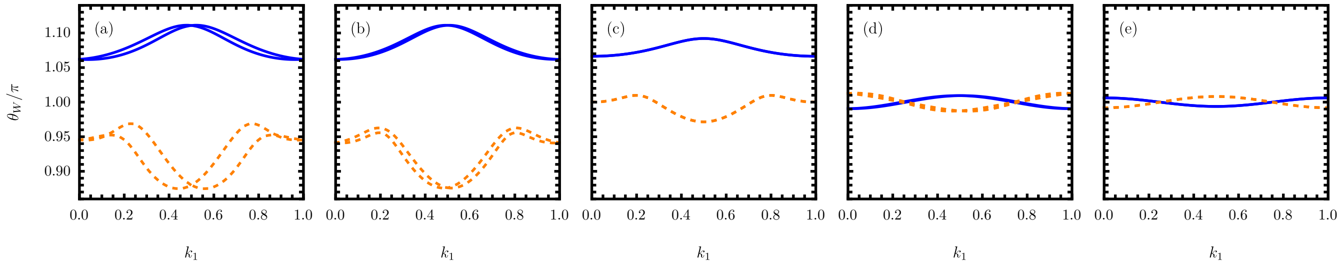

FIG. S121. Wilson loops for the first two sets of conduction bands of twisted AB-stacked bilayer $SnSe_2$ at $\theta = 3.89°$. The Wilson loop is computed along $\mathbf{b}_{M_2}$ We consider the full continuum model (a), the full first moiré harmonic model (b), the reduced first moiré harmonic model (c), the first moiré harmonic model with the zero-twist constraints imposed (d), and the reduced first moiré harmonic model with the zero-twist constraints imposed (e). The blue (dashed orange) lines correspond to the first (second) set of conduction bands.

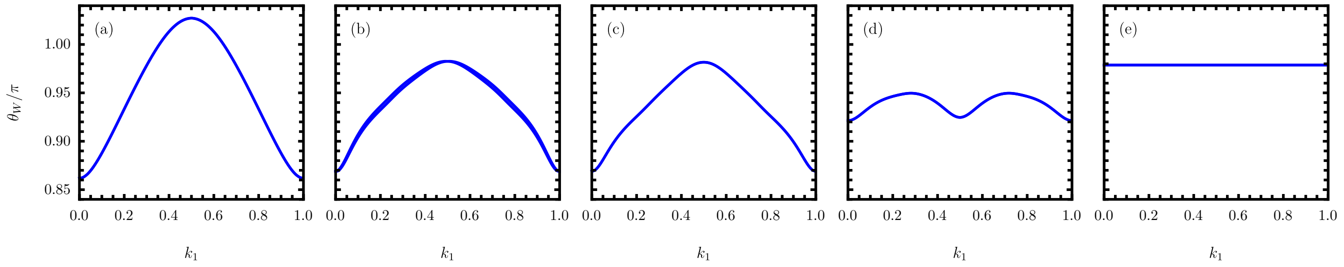

FIG. S122. Wilson loops for the first set of conduction bands of twisted AA-stacked bilayer ZrS_2 at $\theta = 9.43°$. The Wilson loop is computed along $\mathbf{b}_{M_2}$ We consider the full continuum model (a), the full first moiré harmonic model (b), the reduced first moiré harmonic model (c), the first moiré harmonic model with the zero-twist constraints imposed (d), and the reduced first moiré harmonic model with the zero-twist constraints imposed (e).

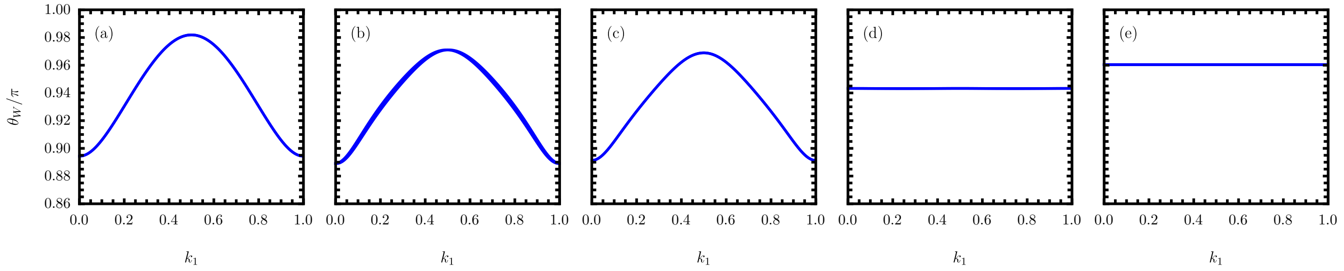

FIG. S123. Wilson loops for the first set of conduction bands of twisted AA-stacked bilayer ZrS_2 at $\theta = 7.34°$. The Wilson loop is computed along $\mathbf{b}_{M_2}$ We consider the full continuum model (a), the full first moiré harmonic model (b), the reduced first moiré harmonic model (c), the first moiré harmonic model with the zero-twist constraints imposed (d), and the reduced first moiré harmonic model with the zero-twist constraints imposed (e).

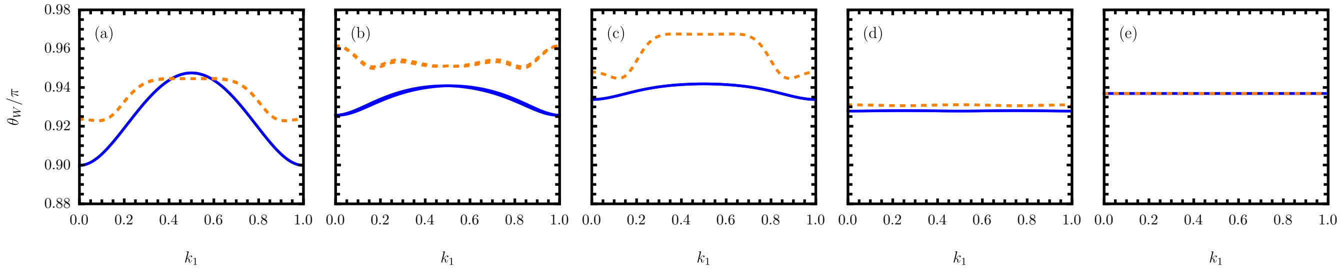

FIG. S124. Wilson loops for the first two sets of conduction bands of twisted AA-stacked bilayer ZrS_2 at $\theta = 6.01°$. The Wilson loop is computed along $\mathbf{b}_{M_2}$ We consider the full continuum model (a), the full first moiré harmonic model (b), the reduced first moiré harmonic model (c), the first moiré harmonic model with the zero-twist constraints imposed (d), and the reduced first moiré harmonic model with the zero-twist constraints imposed (e). The blue (dashed orange) lines correspond to the first (second) set of conduction bands.

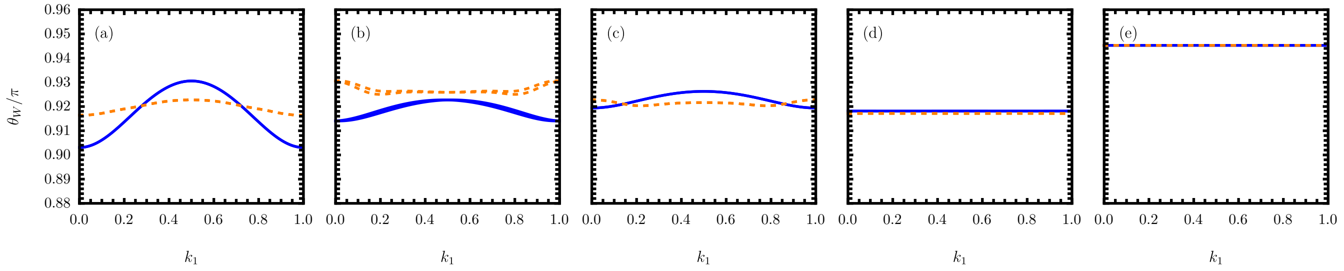

FIG. S125. Wilson loops for the first two sets of conduction bands of twisted AA-stacked bilayer ZrS_2 at $\theta = 5.09°$. The Wilson loop is computed along $\mathbf{b}_{M_2}$ We consider the full continuum model (a), the full first moiré harmonic model (b), the reduced first moiré harmonic model (c), the first moiré harmonic model with the zero-twist constraints imposed (d), and the reduced first moiré harmonic model with the zero-twist constraints imposed (e). The blue (dashed orange) lines correspond to the first (second) set of conduction bands.

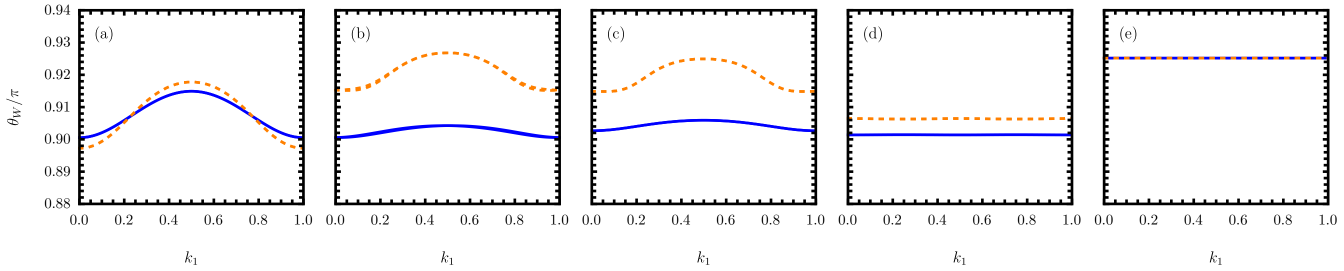

FIG. S126. Wilson loops for the first two sets of conduction bands of twisted AA-stacked bilayer ZrS_2 at $\theta = 4.41°$. The Wilson loop is computed along $\mathbf{b}_{M_2}$ We consider the full continuum model (a), the full first moiré harmonic model (b), the reduced first moiré harmonic model (c), the first moiré harmonic model with the zero-twist constraints imposed (d), and the reduced first moiré harmonic model with the zero-twist constraints imposed (e). The blue (dashed orange) lines correspond to the first (second) set of conduction bands.

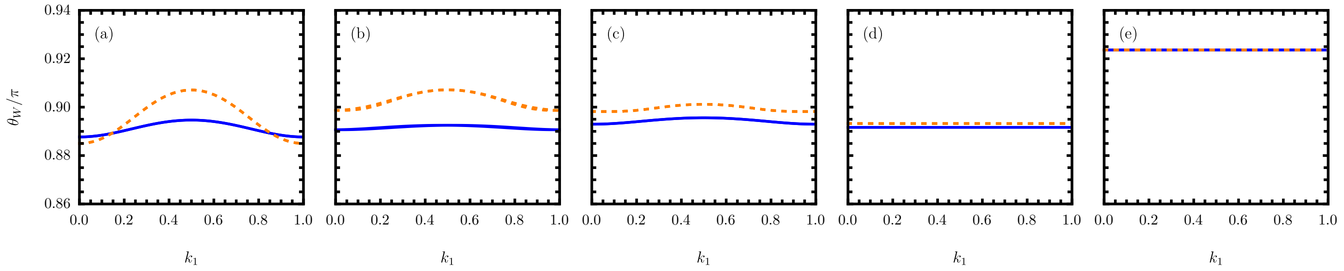

FIG. S127. Wilson loops for the first two sets of conduction bands of twisted AA-stacked bilayer ZrS_2 at $\theta = 3.89°$. The Wilson loop is computed along $\mathbf{b}_{M_2}$ We consider the full continuum model (a), the full first moiré harmonic model (b), the reduced first moiré harmonic model (c), the first moiré harmonic model with the zero-twist constraints imposed (d), and the reduced first moiré harmonic model with the zero-twist constraints imposed (e). The blue (dashed orange) lines correspond to the first (second) set of conduction bands.

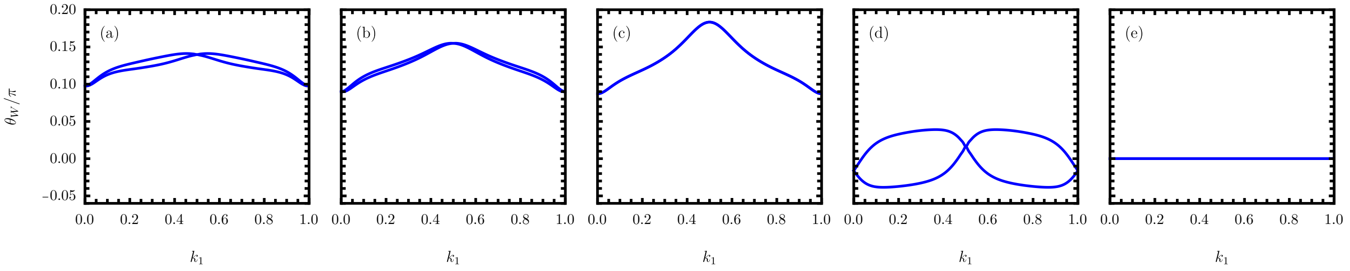

FIG. S128. Wilson loops for the first set of conduction bands of twisted AB-stacked bilayer ZrS_2 at $\theta = 9.43°$. The Wilson loop is computed along $\mathbf{b}_{M_2}$ We consider the full continuum model (a), the full first moiré harmonic model (b), the reduced first moiré harmonic model (c), the first moiré harmonic model with the zero-twist constraints imposed (d), and the reduced first moiré harmonic model with the zero-twist constraints imposed (e).

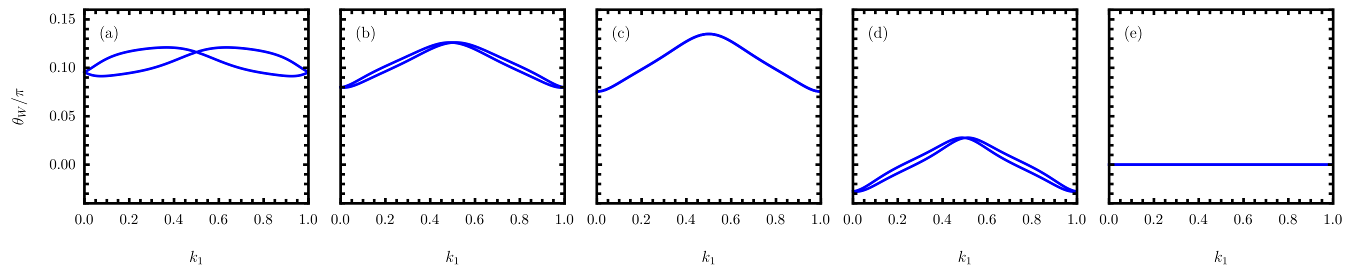

FIG. S129. Wilson loops for the first set of conduction bands of twisted AB-stacked bilayer ZrS_2 at $\theta = 7.34°$. The Wilson loop is computed along $\mathbf{b}_{M_2}$ We consider the full continuum model (a), the full first moiré harmonic model (b), the reduced first moiré harmonic model (c), the first moiré harmonic model with the zero-twist constraints imposed (d), and the reduced first moiré harmonic model with the zero-twist constraints imposed (e).

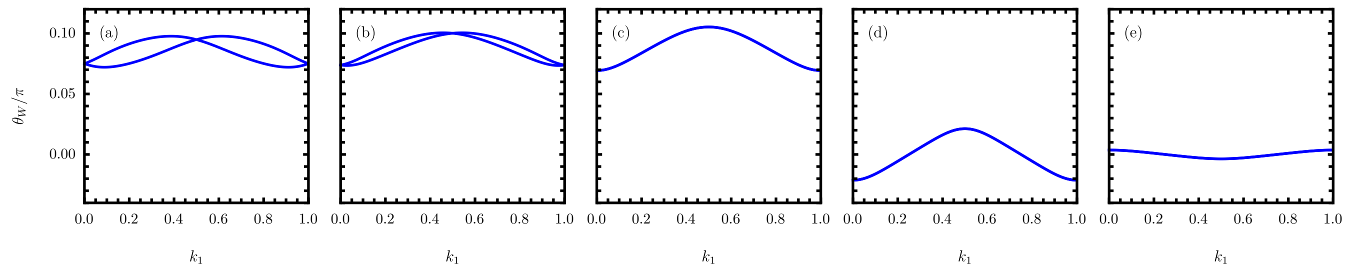

FIG. S130. Wilson loops for the first set of conduction bands of twisted AB-stacked bilayer ZrS_2 at $\theta = 6.01°$. The Wilson loop is computed along $\mathbf{b}_{M_2}$ We consider the full continuum model (a), the full first moiré harmonic model (b), the reduced first moiré harmonic model (c), the first moiré harmonic model with the zero-twist constraints imposed (d), and the reduced first moiré harmonic model with the zero-twist constraints imposed (e).

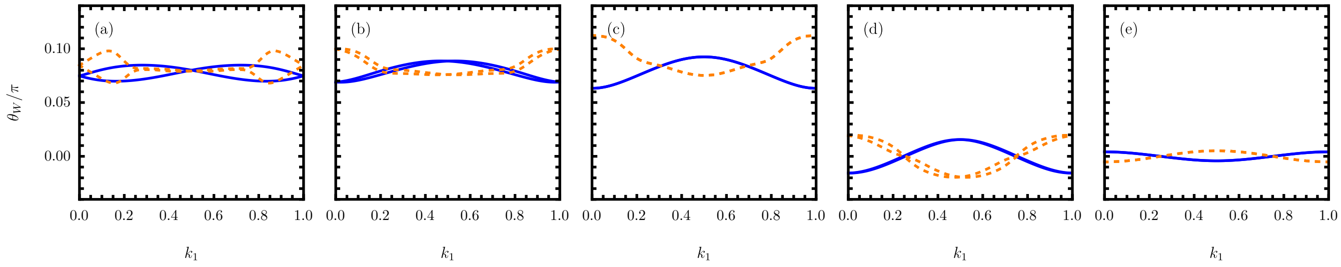

FIG. S131. Wilson loops for the first two sets of conduction bands of twisted AB-stacked bilayer ZrS_2 at $\theta = 5.09°$. The Wilson loop is computed along $\mathbf{b}_{M_2}$ We consider the full continuum model (a), the full first moiré harmonic model (b), the reduced first moiré harmonic model (c), the first moiré harmonic model with the zero-twist constraints imposed (d), and the reduced first moiré harmonic model with the zero-twist constraints imposed (e). The blue (dashed orange) lines correspond to the first (second) set of conduction bands.

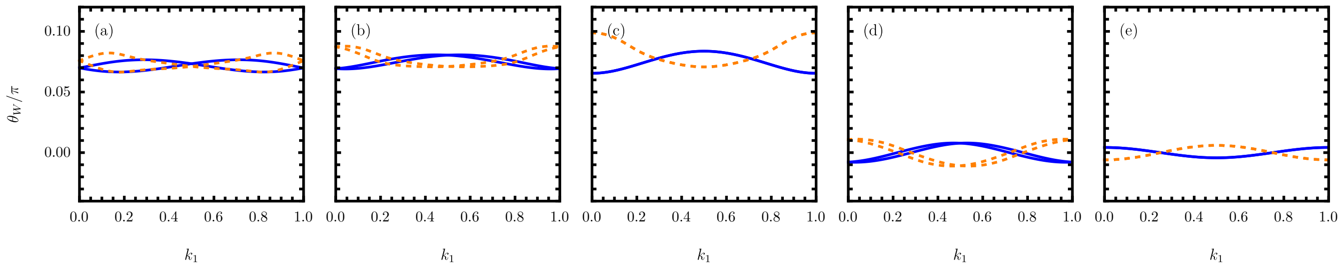

FIG. S132. Wilson loops for the first two sets of conduction bands of twisted AB-stacked bilayer ZrS_2 at $\theta = 4.41°$. The Wilson loop is computed along $\mathbf{b}_{M_2}$ We consider the full continuum model (a), the full first moiré harmonic model (b), the reduced first moiré harmonic model (c), the first moiré harmonic model with the zero-twist constraints imposed (d), and the reduced first moiré harmonic model with the zero-twist constraints imposed (e). The blue (dashed orange) lines correspond to the first (second) set of conduction bands.

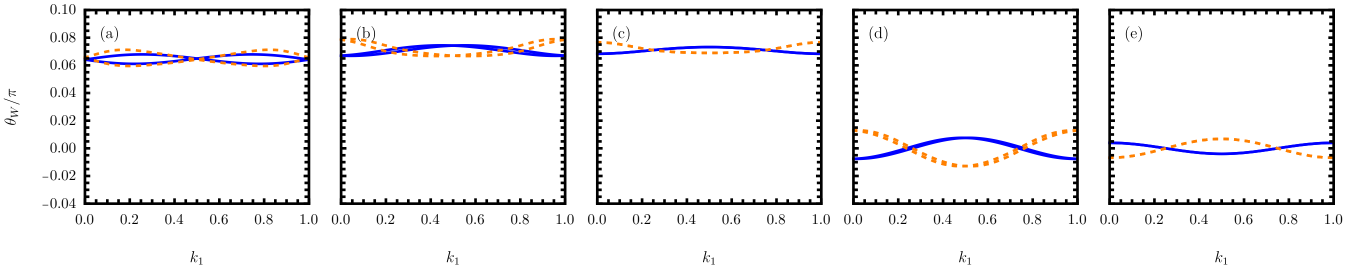

FIG. S133. Wilson loops for the first two sets of conduction bands of twisted AB-stacked bilayer ZrS_2 at $\theta = 3.89°$. The Wilson loop is computed along $\mathbf{b}_{M_2}$ We consider the full continuum model (a), the full first moiré harmonic model (b), the reduced first moiré harmonic model (c), the first moiré harmonic model with the zero-twist constraints imposed (d), and the reduced first moiré harmonic model with the zero-twist constraints imposed (e). The blue (dashed orange) lines correspond to the first (second) set of conduction bands.

[1] R. Bistritzer and A. H. MacDonald, PNAS **108**, 12233 (2011).
[2] J. Jung, A. Raoux, Z. Qiao, and A. H. MacDonald, Phys. Rev. B **89**, 205414 (2014).

[3] H. Hu, Y. Jiang, D. Călugăru, X. Feng, D. Subires, M. G. Vergniory, C. Felser, S. Blanco-Canosa, and B. A. Bernevig, Phys. Rev. B **111**, 054113 (2025).
[4] Y. Jiang, H. Hu, D. Călugăru, C. Felser, S. Blanco-Canosa, H. Weng, Y. Xu, and B. A. Bernevig, Phys. Rev. B **111**, 125163 (2025).
[5] Y. Zhang, H. Pi, J. Liu, W. Miao, Z. Qi, N. Regnault, H. Weng, X. Dai, B. A. Bernevig, Q. Wu, and J. Yu, arXiv:2411.08108 [cond-mat] (2024), arXiv:2411.08108 [cond-mat].
[6] B. A. Bernevig, Z.-D. Song, N. Regnault, and B. Lian, Phys. Rev. B **103**, 205411 (2021).
[7] H. Wu, S. Li, M. Susner, S. Kwon, M. Kim, T. Haugan, and B. Lv, 2D Mater. **6**, 045048 (2019).
[8] B. Bradlyn, L. Elcoro, J. Cano, M. G. Vergniory, Z. Wang, C. Felser, M. I. Aroyo, and B. A. Bernevig, Nature **547**, 298 (2017).
[9] H. C. Po, A. Vishwanath, and H. Watanabe, Nat. Commun. **8**, 50 (2017).
[10] J. Kruthoff, J. de Boer, J. van Wezel, C. L. Kane, and R.-J. Slager, Phys. Rev. X **7**, 041069 (2017).
[11] F. A. S. Al-Alamy, A. A. Balchin, and M. White, J. Mater. Sci. **12**, 2037 (1977).
[12] C. Busch, C. Fröhlich, and F. Hulliger, Helv. Phys. Acta **34**, 359 (1961).
[13] S. Grimme, J. Comput. Chem. **27**, 1787 (2006).
[14] J. Yu, J. Herzog-Arbeitman, M. Wang, O. Vafek, B. A. Bernevig, and N. Regnault, Phys. Rev. B **109**, 045147 (2024).
[15] Y. Jia, J. Yu, J. Liu, J. Herzog-Arbeitman, Z. Qi, H. Pi, N. Regnault, H. Weng, B. A. Bernevig, and Q. Wu, Phys. Rev. B **109**, 205121 (2024).
[16] S. Batzner, A. Musaelian, L. Sun, M. Geiger, J. P. Mailoa, M. Kornbluth, N. Molinari, T. E. Smidt, and B. Kozinsky, Nat. Commun. **13**, 2453 (2022).
[17] J. Liu, Z. Fang, H. Weng, and Q. Wu, DPmoire: A tool for constructing accurate machine learning force fields in moiré systems (2025), arXiv:2412.19333 [cond-mat].
[18] G. Kresse and J. Furthmüller, Comput. Mater. Sci. **6**, 15 (1996).
[19] G. Kresse and J. Hafner, Phys. Rev. B **48**, 13115 (1993).
[20] G. Kresse and J. Hafner, Phys. Rev. B **47**, 558 (1993).
[21] G. Kresse and J. Hafner, Phys. Rev. B **49**, 14251 (1994).
[22] G. Kresse and J. Furthmüller, Phys. Rev. B **54**, 11169 (1996).
[23] S. Grimme, J. Antony, S. Ehrlich, and H. Krieg, J. Chem. Phys. **132**, 154104 (2010).
[24] F. Xie, A. Cowsik, Z.-D. Song, B. Lian, B. A. Bernevig, and N. Regnault, Phys. Rev. B **103**, 205416 (2021).
[25] B. A. Bernevig, B. Lian, A. Cowsik, F. Xie, N. Regnault, and Z.-D. Song, Phys. Rev. B **103**, 205415 (2021).
[26] B. Lian, Z.-D. Song, N. Regnault, D. K. Efetov, A. Yazdani, and B. A. Bernevig, Phys. Rev. B **103**, 205414 (2021).
[27] B. A. Bernevig, Z.-D. Song, N. Regnault, and B. Lian, Phys. Rev. B **103**, 205413 (2021).
[28] Z.-D. Song, B. Lian, N. Regnault, and B. A. Bernevig, Phys. Rev. B **103**, 205412 (2021).
[29] D. Călugăru, F. Xie, Z.-D. Song, B. Lian, N. Regnault, and B. A. Bernevig, Phys. Rev. B **103**, 195411 (2021).
[30] F. Xie, N. Regnault, D. Călugăru, B. A. Bernevig, and B. Lian, Phys. Rev. B **104**, 115167 (2021).
[31] Z. Song, Z. Wang, W. Shi, G. Li, C. Fang, and B. A. Bernevig, Phys. Rev. Lett. **123**, 036401 (2019).
[32] S. Carr, D. Massatt, S. B. Torrisi, P. Cazeaux, M. Luskin, and E. Kaxiras, Phys. Rev. B **98**, 224102 (2018).
[33] M. G. Scheer, K. Gu, and B. Lian, Phys. Rev. B **106**, 115418 (2022).
[34] L. Zou, H. C. Po, A. Vishwanath, and T. Senthil, Phys. Rev. B **98**, 085435 (2018).
[35] Z. Y. Chen, S. A. Yang, and Y. X. Zhao, Nat. Commun. **13**, 2215 (2022).
[36] C. Zhang, Z. Y. Chen, Z. Zhang, and Y. X. Zhao, Phys. Rev. Lett. **130**, 256601 (2023).
[37] T. Ozaki, Phys. Rev. B **67**, 155108 (2003).
[38] T. Ozaki and H. Kino, Phys. Rev. B **69**, 195113 (2004).
[39] T. Ozaki and H. Kino, Phys. Rev. B **72**, 045121 (2005).
[40] K. Lejaeghere, G. Bihlmayer, T. Björkman, P. Blaha, S. Blügel, V. Blum, D. Caliste, I. E. Castelli, S. J. Clark, A. Dal Corso, S. de Gironcoli, T. Deutsch, J. K. Dewhurst, I. Di Marco, C. Draxl, M. Dułak, O. Eriksson, J. A. Flores-Livas, K. F. Garrity, L. Genovese, P. Giannozzi, M. Giantomassi, S. Goedecker, X. Gonze, O. Grånäs, E. K. U. Gross, A. Gulans, F. Gygi, D. R. Hamann, P. J. Hasnip, N. A. W. Holzwarth, D. Iuşan, D. B. Jochym, F. Jollet, D. Jones, G. Kresse, K. Koepernik, E. Küçükbenli, Y. O. Kvashnin, I. L. M. Locht, S. Lubeck, M. Marsman, N. Marzari, U. Nitzsche, L. Nordström, T. Ozaki, L. Paulatto, C. J. Pickard, W. Poelmans, M. I. J. Probert, K. Refson, M. Richter, G.-M. Rignanese, S. Saha, M. Scheffler, M. Schlipf, K. Schwarz, S. Sharma, F. Tavazza, P. Thunström, A. Tkatchenko, M. Torrent, D. Vanderbilt, M. J. van Setten, V. Van Speybroeck, J. M. Wills, J. R. Yates, G.-X. Zhang, and S. Cottenier, Science **351**, aad3000 (2016).
[41] P.-O. Löwdin, J. Chem. Phys. **18**, 365 (1950).
[42] J. D. Cloizeaux, Phys. Rev. **135**, A685 (1964).
[43] N. Marzari and D. Vanderbilt, Phys. Rev. B **56**, 12847 (1997).
[44] N. Marzari, A. A. Mostofi, J. R. Yates, I. Souza, and D. Vanderbilt, Rev. Mod. Phys. **84**, 1419 (2012).
[45] E. Polizzi, Phys. Rev. B **79**, 115112 (2009).
[46] W. Miao, C. Li, X. Han, D. Pan, and X. Dai, Phys. Rev. B **107**, 125112 (2023).
[47] J. M. B. Lopes dos Santos, N. M. R. Peres, and A. H. Castro Neto, Phys. Rev. Lett. **99**, 256802 (2007).
[48] T. Fukui, Y. Hatsugai, and H. Suzuki, J. Phys. Soc. Jpn. **74**, 1674 (2005).
[49] C. Brouder, G. Panati, M. Calandra, C. Mourougane, and N. Marzari, Phys. Rev. Lett. **98**, 046402 (2007).
[50] A. Alexandradinata, X. Dai, and B. A. Bernevig, Phys. Rev. B **89**, 155114 (2014).
